# B(C_6_F_5_)_3_-Catalyzed Diastereoselective and Divergent Reactions of Vinyldiazo Esters with Nitrones: Synthesis of Highly Functionalized Diazo Compounds

**DOI:** 10.1021/acs.orglett.2c04198

**Published:** 2023-01-12

**Authors:** Katarina Stefkova, Michael G. Guerzoni, Yara van Ingen, Emma Richards, Rebecca L. Melen

**Affiliations:** Cardiff Catalysis Institute, School of Chemistry, Cardiff University, Main Building, Park Place, Cardiff, CF10 3AT, Cymru/Wales, United Kingdom

## Abstract

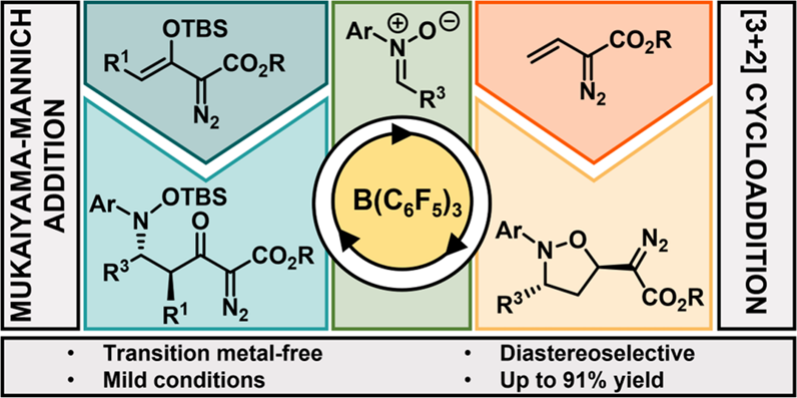

Herein we report a mild, transition-metal-free, highly diastereoselective Lewis acid catalyzed methodology toward the synthesis of isoxazolidine-based diazo compounds from the reaction between vinyldiazo esters and nitrones. Interestingly, the isoxazolidine products were identified to have contrasting diastereoselectivity to previously reported metal-catalyzed reactions. Furthermore, the same catalyst can be used with enol diazo esters, prompting the formation of Mukaiyama–Mannich products. These diazo products can then be further functionalized to afford benzo[*b*]azepine and pyrrolidinone derivatives.

Over the past decade, alkenyldiazo compounds have been shown to be versatile reagents for the preparation of a wide variety of hetero- and carbocycles. The reactivity of alkenyldiazo compounds has been found to be influenced by the substitutions on the vinyl moiety or the choice of a catalyst used for the desired transformation.^1^ Vinyldiazo compounds have been mainly used in the presence of transition metals (i.e., Rh, Ag, Au, Cu) to form electrophilic metal carbene intermediates, which then undergo [3+*n*] (*n* = 2–4) cycloaddition reactions with dienophiles, including nitrones (Scheme 1).^2^ Though not as prevalent, and again mostly in the presence of transition metals, there have been several reports on utilizing alkenyldiazo reagents for their nucleophilic character, originating from the vinyl functionality, to form acyclic and cyclic compounds, where the diazo functionality remains intact.^3^ The remaining diazo group can then be used in further synthetic transformations (Scheme 1c,e).

**Scheme 1:**
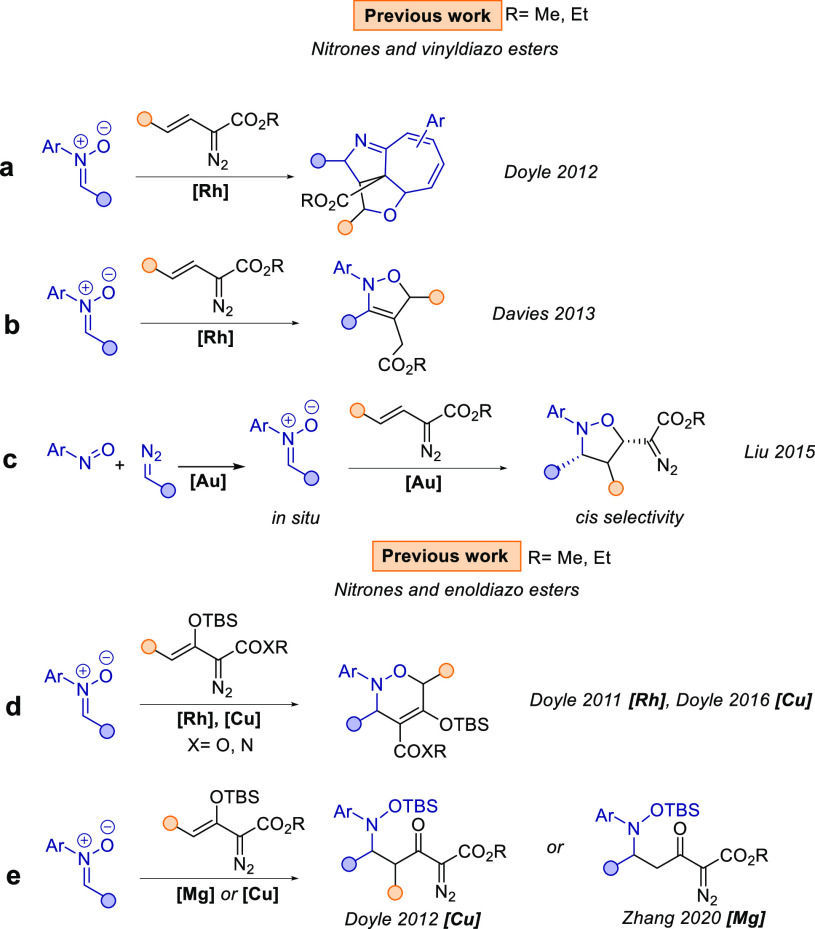
Previous Work

In the past few years, we and others have shown that the boron Lewis acid, B(C_6_F_5_)_3_, activates α-aryl α-diazo esters in a similar fashion to metal catalysts, and this has been utilized for transition-metal-free cyclization, alkenylation, or X–H insertion reactions. Moreover, these transformations are highly diastereoselective, which has been attributed to the steric hindrance around the boron center.^4^ In our studies we have observed that the activation of the diazo moiety strongly depends on the nature of the diazo compound, so there are cases where the diazo functionality could remain intact and be activated at later stages. With this in mind, we were curious whether borane catalysts can be employed for cycloaddition reactions in the presence of vinyldiazo esters to access heterocyclic, highly functionalized diazo compounds (Scheme 2).

**Scheme 2:**
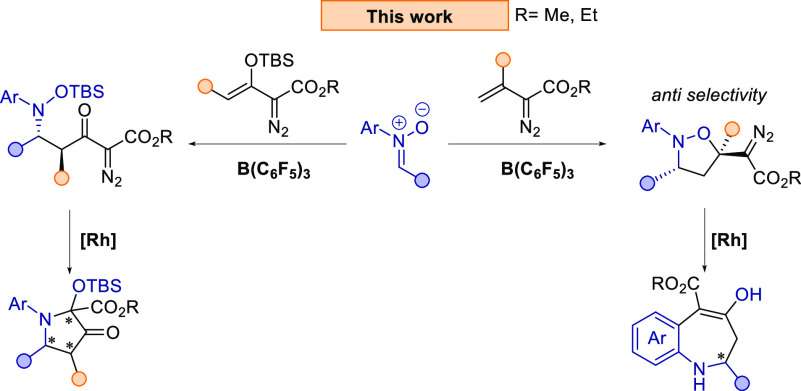
Work Presented in This Manuscript

Accessing isoxazolidine-derived diazo reagents has caught our interest due to the heterocycle’s labile N–O bond, which can undergo ring-opening reactions to access novel bioactive heterocycles, including pyrrolidines and benzoazepines.^5^ Moreover, fused bicyclic isoxazolidines have been found in natural products and can possess cytotoxic, antifungal, or anti-inflammatory activities.^6^ The most straightforward synthetic strategy to access isoxazolidine-derived scaffolds is via a thermal 1,3-dipolar cycloaddition of nitrones and alkenes, which often results in poor diastereoselective and regioselective control.^7^ Furthermore, applying high temperatures is not compatible with the unstable alkenyldiazo reagents, which can readily undergo decomposition.^8^ As such, accessing isoxazolidine-derived diazo esters under mild conditions and in high diastereoselectivities remains challenging and, to the best of our knowledge, such methodology has only been reported in the presence of a gold catalyst (Scheme 1a).^3b^

To this end we initiated our studies by exploring the optimal reaction conditions for the cycloaddition of nitrone **1a** with alkenyldiazo ester **2a** as our model substrates (Table 1). The initial control reactions, carried out in the absence of a catalyst at room temperature and at 80 °C in 1,2-dichloroethane (1,2-C_2_H_4_Cl_2_), showed no product formation after 24 h, and diazo decomposition was observed (entries 1 and 2). However, when the same reaction was carried out at 40 °C for 36 h, the formation of isoxazolidine **3a** was observed in good diastereoselectivity (dr 9:91), though in poor yield (18%) (entry 3). Utilizing 20 mol % BF_3_·OEt_2_ did not improve the yield or the diastereoselectivity (entry 4). However, when 20 mol % B(C_6_F_5_)_3_ was used as a catalyst, not only was the yield of **3a** improved to 60% but also the diastereoselectivity was inversed (dr 83:17) (entry 5). Other aryl fluorinated boranes, such as B(3,4,5-F_3_C_6_H_2_)_3_ [B(3,4,5-Ar^F^)_3_] and B(2,4,6-F_3_C_6_H_2_)[B(2,4,6-Ar^F^)_3_], failed to catalyze the cycloaddition reaction efficiently giving **3a** in just 28% and 23% yield and in poor diastereoselectivities (38:62 and 48:52) (entries 6 and 7). When BPh_3_ was screened as a catalyst, less than 5% of product formation was observed (entry 8), and Brønsted acidic TfOH failed to catalyze the reaction completely (entry 9). Decreasing the catalytic loading of B(C_6_F_5_)_3_ from 20 to 10 and 5 mol % resulted in both poorer diastereoselectivities and yields (entries 10, 11, and 14). Lastly, different solvents were screened for the cycloaddition reaction (entries 12–19). When the reaction was carried out in dichloromethane (CH_2_Cl_2_), the dr remained unchanged, and the yield improved slightly to 63% (entry 16). Both trifluorotoluene (C_6_H_5_CF_3_) and hexane improved the diastereoselectivities to 87:13 and 89:11, though the overall yields were decreased to 56% and 45%, respectively (entries 17 and 18). Coordinating solvents such as acetonitrile (MeCN) and tetrahydrofuran (THF) either completely failed to form the isoxazolidine product (entry 19) or **3a** was obtained in a lower yield of 32% and in poor diastereoselectivity (65:35) (entry 13). This is presumably due to the deactivation of the borane catalyst. The most favorable reaction conditions were obtained when the reaction was carried out in toluene at either room temperature or 40 °C (entries 12 and 13). Not only was the yield of **3a** improved to 72–74% but an excellent diastereoselectivity (91:9) was also observed. We decided to use the conditions in entry 12 as the optimized conditions for the scope investigation.

**Table 1:**
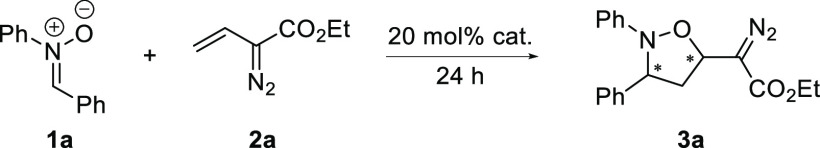
Reaction Optimization for the Cycloaddition Reaction

Entry	Catalyst	Solvent	Temp (°C)	dr^a^ (anti/syn)	Yield^b^ of **3a** (%)
1		C_2_H_4_Cl_2_	rt		
2		C_2_H_4_Cl_2_	80		
3^c^		C_2_H_4_Cl_2_	40	9:91	18
4	BF_3_·OEt_2_	C_2_H_4_Cl_2_	40	18:82	15
5	B(C_6_F_5_)_3_	C_2_H_4_Cl_2_	40	83:17	60
6	B(3,4,5-Ar^F^)_3_	C_2_H_4_Cl_2_	40	38:62	28
7	B(2,4,6-Ar^F^)_3_	C_2_H_4_Cl_2_	40	48:52	23
8	BPh_3_	C_2_H_4_Cl_2_	40		<5
9	TfOH	C_2_H_4_Cl_2_	40		
10^d^	B(C_6_F_5_)_3_	C_2_H_4_Cl_2_	40	71:29	44
11^e^	B(C_6_F_5_)_3_	C_2_H_4_Cl_2_	40	57:43	26
12	B(C_6_F_5_)_3_	Toluene	40	91:9	74
13	B(C_6_F_5_)_3_	Toluene	RT	91:9	72
14^d^	B(C_6_F_5_)_3_	Toluene	RT	80:20	42
15	B(C_6_F_5_)_3_	THF	40	65:35	32
16	B(C_6_F_5_)_3_	CH_2_Cl_2_	40	83:17	63
17	B(C_6_F_5_)_3_	C_6_H_5_CF_3_	40	87:13	56
18	B(C_6_F_5_)_3_	Hexane	40	89:11	45
19	B(C_6_F_5_)_3_	MeCN	40		

a Determined from ^1^H NMR spectra of the crude reaction mixture.

b Isolated yields of the diastereomeric mixture.

c Reaction ran for 36 h.

d 10 mol % B(C_6_F_5_)_3_.

e 5 mol % B(C_6_F_5_)_3_.

With the optimized conditions in hand, we explored a substrate scope for the reaction (Scheme 3). First, nitrones **1a**–**1i** and **1p** (see Figure S1) with different substitutions at the R^1^ position were explored. In general, *para*-substituted halogen groups (*p*-F and *p*-Cl) on the phenyl ring resulted in excellent yields [82% (**3e**) and 84% (**3f**), respectively] and excellent diastereoselectivities (92:8 and 91:9, respectively). Notably, when the *p*-CF_3_ group was present, the isoxazolidine **3d** was formed as a single diastereoisomer in a good yield of 76%. Furthermore, both 2-naphthyl and 1-naphthyl substitutions were also tolerated as **3b** and **3c** were produced in excellent yields of 85% and 83%, respectively, though **3c** was formed in poorer diastereoselectivities (68:32) than **3b** (89:11). Moderate diastereoselectivity and yield were also observed with *o*-Br substitution, in which **3g** was isolated in 65% yield (dr 69:31). An electron-donating *p*-OMe group was tolerated, and **3h** was obtained in 68% yield and good diastereoselectivity (85:15). However, no formation of the desired product was observed with mesityl nitrone (**1p**). Lastly, when a phenyl ring was replaced with a cyclohexyl moiety, the isoxazolidine-derivative **3i** was formed in excellent yield (87%) and in good diastereoselectivity (82:18). Subsequently, nitrones with different *N*-aryl substitutions (**1j**–**1o**, see Figure S1) were explored. In general, good to excellent yields (82–91%) and diastereoselectivities (up to 89:11) were obtained with halogen substitutions at the *para*-position (**3m**–**3o**). Moreover, the isoxazolidine products **3j** (*p*-Me), **3k** (*o*-Me), and **3l** (*o*-Et) with electron-donating alkyl groups were formed in 74–77% yields and in excellent diastereoselectivities. However, when a nitrone with a stronger electron-donating *p*-OMe group (**1q**) was employed, the reaction resulted in less than 5% of the desired product. Additionally, nitrones with two naphthyl substitutions (**1r**) or an *N*-alkyl group (**1s**) failed to react. Additionally, we screened internal alkenyldiazo esters bearing methyl (**2b**) and phenyl (**2c**) substitutions; however, these did not react with the nitrone **1a**. Alkenyldiazo ester **2d** afforded the desired isoxazolidine **3p** bearing a quaternary stereocenter, but a lower temperature and longer reaction time was required. Lastly, this methodology also proved applicable on a larger scale. Reaction of nitrone **1e** with the alkenyldiazo ester **2a** on a 1.0 mmol scale formed the isoxazolidine **3f** in 85% yield (92:8).

**Scheme 3:**
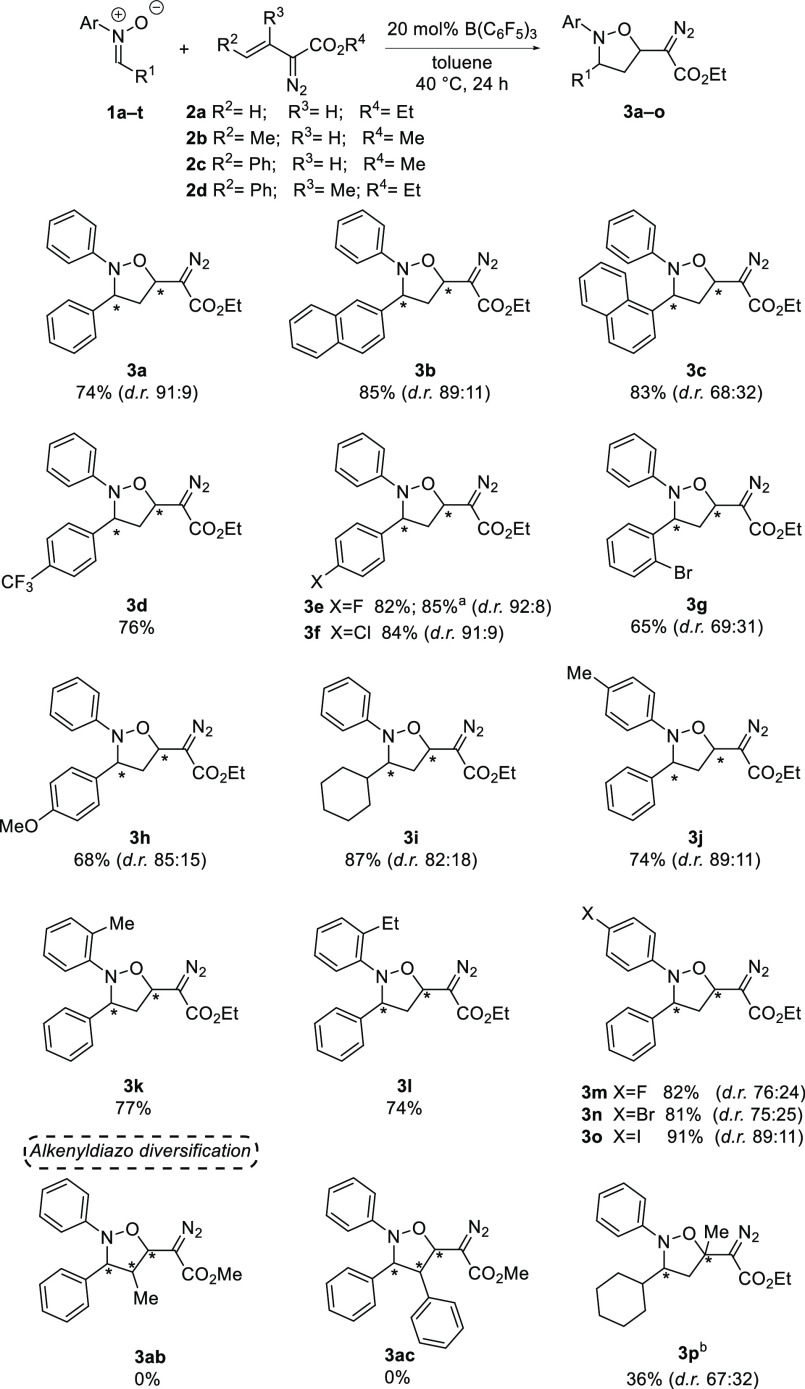
Substrate Scope of the B(C_6_F_5_)_3_-Catalyzed Cycloaddition Reaction of Vinyldiazo Esters with Nitrones Reaction carried out on a 1.0 mmol scale. Reaction carried out at room temperature for 3 days.

Crystals of product **3i** were grown by slow evaporation from CH_2_Cl_2_, whose structure was elucidated by single-crystal X-ray diffraction. The obtained crystal structure (Figure 1, left) revealed (*R*,*R*) stereochemistry, rendering *anti*-**3i** to be the major diastereoisomer. In the previously reported gold-catalyzed isoxazolidine formation by Liu et al., the *syn* diastereoisomer was obtained.^3b^ We propose that the alternate selectivity observed in our study is likely due to the large steric demand of the borane catalyst as seen in other B(C_6_F_5_)_3_-catalyzed cycloaddition reactions.^9^

**Figure 1:**
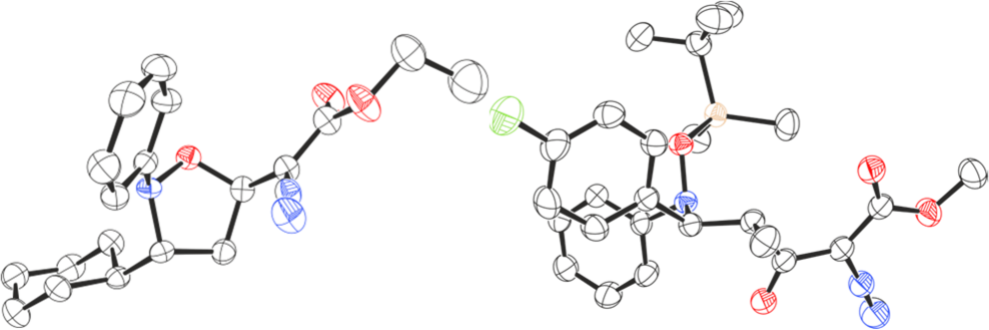
Solid-state structures of **3i** (left) and **5d** (right). Carbon: black; oxygen: red; nitrogen: blue; fluorine: green; silicon; beige. H atoms omitted for clarity. Thermal ellipsoids drawn at 50% probability.

Interestingly, when enol diazoacetate (**4a**) was reacted with nitrone **1e**, the expected [3 + 2] cycloaddition product was not observed and instead the Mukaiyama–Mannich product **5l** was obtained in 90% yield (Scheme 4). Similar results have previously been reported in the presence of Cu- and Mg-based Lewis acids.^3a,3d,3e^ These observations led us to investigate the Mukaiyama–Mannich addition reaction. First, the optimized reaction conditions for the cycloaddition reaction (Table 1, entry 12) were tested for the Mukaiyama–Mannich addition with enol diazoacetate **4b** (R^2^ = Me) and nitrone **1e**. Product **5d** was obtained in an excellent yield of 83% and in moderate diastereoselectivity (73:27). Lower temperatures (0 °C–room temperature) gave almost identical yields and diastereoselectivities (83% and 74:26 respectively). A lower catalytic loading of 10 mol % was also tested, and product **5d** was obtained in equivalent yield (83%).

The optimized reaction conditions for the Mukaiyama–Mannich reaction were set to 0 °C to room temperature with 10 mol % catalyst loading, and a scope was explored (Scheme 4). The product **5d** was isolated as a white crystalline solid, and its solid-state structure was elucidated by single-crystal X-ray diffraction (Figure 1, right) to reveal the *anti*-diastereoisomer (*anti*-**5d**) as the major product.

**Scheme 4:**
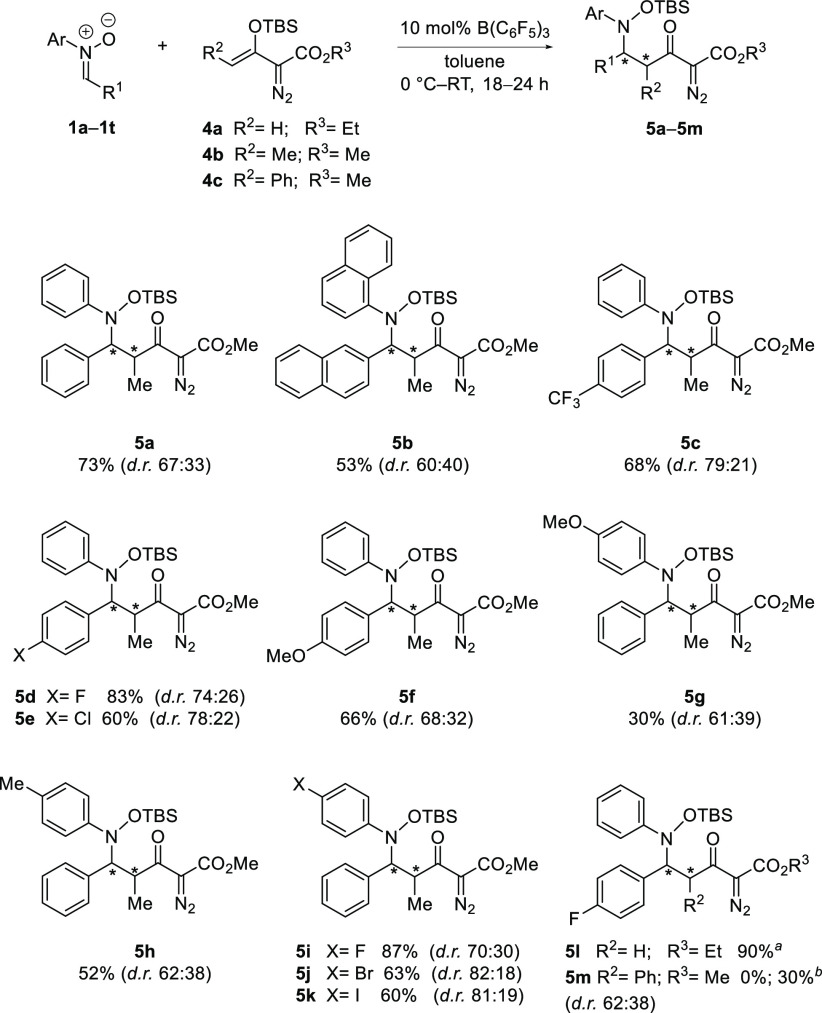
Substrate Scope for the B(C_6_F_5_)_3_-Catalyzed Mukaiyama–Mannich Addition Reactions 20 mol % B(C_6_F_5_)_3_, 40 °C. Reaction carried out in 1,2-dichloroethane.

Investigation of the reaction scope revealed products bearing electron-withdrawing groups at the *para* position of R^1^ = aryl (**5c**–**5e**) were formed in good to excellent yields (60–83%), with the best dr observed for *p*-CF_3_ [79:21 (**5c**)] substitution. Mukaiyama–Mannich addition products with neutral (**5a**) and electron-donating *p*-OMe (**5f**) groups were obtained in yields of 73% and 66%, respectively, in moderate diastereoselectivities [67:33 (**5a**)] and [68:32 (**5f**)]. Similar observations were noticed with varying *N*-aryl substitution. Products with *p*-F (**5i**), *p*-Br (**5j**), and *p*-I (**5k**) substitutions were formed in up to 87% yield and with very good diastereoselectivities (up to 82:18).

A more moderate yield of 52% and diastereoselectivity (62:38) was observed with a *p*-Me moiety (**5h**). Notably, the previously limiting substrates for the [3 + 2] cycloaddition reactions, *p*-OMe (**1q**) and dinaphthyl nitrone (**1r**), reacted with the enoldiazo ester **4b** giving **5g** (30%) and **5b** (53%). On the other hand, cyclohexyl (**1i**), mesityl (**1p**), and *N*-alkyl (**1s**) nitrones failed to react. Lastly, when the more sterically hindered enoldiazo ester (**4c**) was reacted with nitrone (**1e**), no product formation was observed. However, by changing the solvent to 1,2-dichloroethane, product (**5m**) was obtained, though in poor yield (30%).

Using our methodology both the isoxazolidine and Mukaiyama–Mannich addition products maintain the diazo functionality intact, which could be used for further functionalization. As a proof of concept, we took inspiration from the works of Doyle^3e^ and Liu,^3b^ and we subjected our substrates to the metal-catalyzed decomposition of the diazo functionality (Scheme 5).

**Scheme 5:**
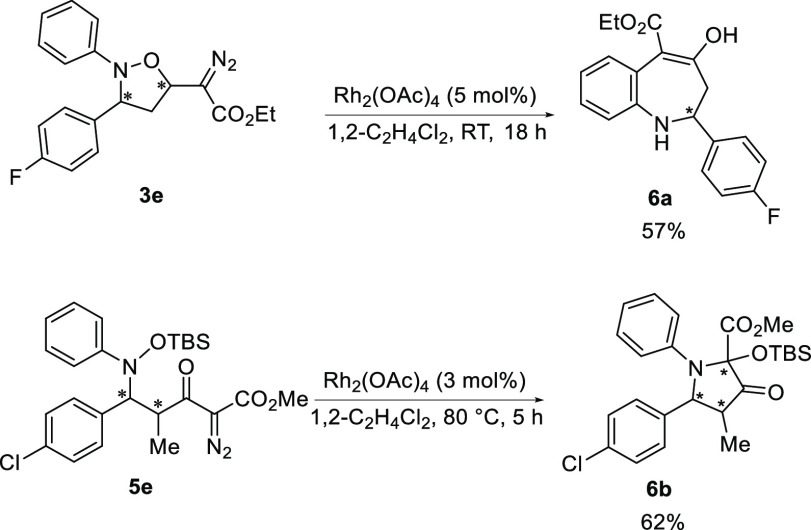
Further Functionalization of the Isoxazolidine and Mukaiyama–Mannich Addition Products

Rh_2_(OAc)_4_ (5 mol %) proved to be a good catalyst for the synthesis of benzo[*b*]azepine cores from **3a** generating **6a** in 57% yield. Interestingly, by using B(C_6_F_5_)_3_ (10 mol %) only 10% **6a** was formed. Rh_2_(OAc)_4_ (3 mol %) could also catalyze the formation of pyrrolidinone **6b** from the Mukayiama–Mannich product **5e**.

In summary, we have developed a transition-metal-free, Lewis acid catalyzed diastereoselective method toward highly functionalized isoxazolidine-derived diazo compounds in yields up to 91%. Interestingly, the diastereoselectivity observed in these reactions is opposite to that observed with similar gold-catalyzed transformations offering complementarity to transition-metal-catalyzed processes. Moreover, we have demonstrated and utilized the divergent reactivities of vinyldiazo compounds with nitrones through the substitution pattern present in the alkenyldiazo ester. To this end, we obtained Mukaiyama–Mannich addition diazo products in up to 90% yield. As such, a diverse pool of highly functionalized diazo compounds has been presented. Their further transformation toward medicinally relevant scaffolds has also been demonstrated.

## Data Availability

The data underlying this study are available in the published article and its Supporting Information. Information about the data that underpins the results presented in this article can be found in the Cardiff University data catalogue at 10.17035/d.2023.0236343549.

## Supporting Information Available

Experimental procedures, full characterization of products, copies of ^1^H, ^13^C, ^19^F NMR spectra, copies of mass spectra, and the X-ray crystallographic structures of compounds **3i** and **5e** (PDF)
Elemental composition data (ZIP)
