# Correction to “B(C_6_F_5_)_3_-Catalyzed Diastereoselective and Divergent Reactions of Vinyldiazo Esters with Nitrones: Synthesis of Highly Functionalized Diazo Compounds”

**DOI:** 10.1021/acs.orglett.4c02477

**Published:** 2024-07-11

**Authors:** Katarina Stefkova, Michael G. Guerzoni, Yara van Ingen, Emma Richards, Rebecca L. Melen

The authors regret that a citation related to a previous publication on the activation of nitrones by boranes is missing in the original article. The full reference is presented herein as ref (1).

The authors apologize for this error and any consequent inconvenience to readers.
