# Supplementary material for: B(C6F5)3-Catalyzed Diastereoselective and Divergent Reactions of Vinyldiazo Esters with Nitrones: Synthesis of Highly Functionalized Diazo Compounds
Source: Org Lett. 2023 Jan 12;25(3):500–5. doi: 10.1021/acs.orglett.2c04198 (PMC9887602; doi:10.1021/acs.orglett.2c04198)
Supplement: Supplementary file 1 — ol2c04198_si_001.pdf [file ol2c04198_si_001.pdf]

Supporting Information

$B(C_6F_5)_3$ Catalyzed Diastereoselective and Divergent Reactions of Vinyldiazo Esters with Nitrones: Synthesis of Highly Functionalized Diazo Compounds

Katarina Stefkova,‡ Michael G. Guerzoni,‡ Yara van Ingen, Emma Richards,* Rebecca L. Melen*

Cardiff Catalysis Institute, School of Chemistry, Cardiff University, Main Building, Park Place, Cardiff, CF10 3AT, Cymru/Wales, United Kingdom
*E-mail: RichardsE10@cardiff.ac.uk , MelenR@cardiff.ac.uk

Table of Content

1.0	Experimental		3
	1.1	General Experimental	3
1.2	Synthesis of Starting Materials		4
	1.2.1	Synthesis and Spectral Characterization of Nitrones [General procedure a]	4
	1.2.2	Synthesis of Vinyldiazo Esters [General procedure b]	6
	1.2.3	Synthesis of Enoldiazo Esters [General procedure c]	7
2.0	Synthesis of Products	[General procedures d, e]	9
	2.1	Synthesis and Spectral Characterization of Isoxazolidine-Derived Diazo Products	10
	2.2	Synthesis and Spectral Characterization of Mukaiyama-Mannich Addition Diazo Products	19
	2.3	Further functionalization and Spectral Characterization of Isoxazolidine and Mukaiyama-Mannich Addition Diazo Products	29
3.0	NMR Spectra		30
	3.1	2D-NMR Spectra	112
4.0	Crystallographic Data		122
	4.1	Single Crystal X-Ray Diffraction	122
	4.2	Solid-State Structures	123
	4.3	X-Ray Refinement Data	125
5.0	References		127

1.0 Experimental

1.1 General Experimental

Except for the nitrone starting materials, all reactions and manipulations were carried out under an atmosphere of dry, O_2-free nitrogen using standard double-manifold techniques with a rotary oil pump. A nitrogen-filled glove box (MBraun) was used to manipulate solids including the storage of starting materials, ambient temperature reactions, product recovery and sample preparation for analysis. For reactions requiring heating, an oil bath has been used. All solvents (dichloromethane, hexane, acetonitrile, toluene) were dried by employing a Grubbs-type column system (Innovative Technology) or a solvent purification system MB SPS-800 and stored under a nitrogen atmosphere. Anhydrous (with Sure/Seal) 1,2-dichloroethane (1,2-DCE) and α, α, α-Trifluorotoluene (TFT) were purchased from Merck and dried over molecular sieves before use. Deuterated solvents were distilled and/or dried over molecular sieves before use. Chemicals were purchased from commercial suppliers and used as received. All the triarylfluoroboranes were prepared as per the standard literature report.[1] Thin-layer chromatography (TLC) was performed on pre-coated aluminum sheets of Merck silica gel 60 F254 (0.20 mm). ^{1}H, ^{13}C, and ^{19}F NMR spectra were recorded on a Bruker Avance II 300 or 400 or Bruker Avance 500 spectrometers. All coupling constants are absolute values and are expressed in Hertz (Hz). ^{13}C NMR was measured as ^{1}H decoupled. Yields are given as isolated yields. Yields reported are the combined isolated yields of major and minor diastereoisomers. Unless stated otherwise, all characterization reported is that of the major diastereoisomer. Chemical shifts are expressed as parts per million (ppm, δ) downfield of tetramethylsilane (TMS) and are referenced to $CDCl_3$ (7.26/77.16 ppm) as internal standard. The description of signals includes s = singlet, d = doublet, t = triplet, q = quartet, qd = quartet of doublets, and m = multiplet, br. s = broad singlet. Structural assignments were made with additional information from gCOSY, gHSQC, and gHMBC experiments. All spectra were analyzed assuming a first order approximation. IR-Spectra were measured on a Shimadzu IRAffinity-1 photo-spectrometer. Mass spectra were measured on a Waters LCT Premier/XE or a Waters GCT Premier spectrometer. Ions were generated by Electrospray (ES). The molecular ion peaks values are quoted for molecular ion plus hydrogen ($M+H^+$). Moreover, we carried out the cycloaddition reactions in vessels wrapped in aluminum foil and used 2 equiv. of the vinyldiazo ester **2a**, as **2a** readily undergoes thermal decomposition at room temperature, as well as photodecomposition in the presence of visible light.[2]

1.2 Synthesis of Starting Materials

1.2.1 Synthesis and Spectral Characterization of Nitrones

General procedure a: nitrobenzene (1 equiv.), benzaldehyde (1.1 equiv.), and NH_4Cl (1.2 equiv.) were added to a 1:1 mixture of ethanol:water (2 mL/mmol) and the resulting mixture was stirred for 5 minutes at room temperature. The mixture was then cooled to 0 °C, and Zn dust (2 equiv.) was added portion-wise over 30 minutes. Subsequently, the reaction was slowly warmed to room temperature and stirred overnight. The resulting mixture was then filtered through a pad of celite, and the organics were extracted using CH_2Cl_2 (3 × 40 mL), washed with brine (1 × 40 mL), dried over Na_2SO_4, and concentrated *in vacuo*. The crude compound was purified by recrystallization using ethanol or ethyl acetate. The nitrones **1a**–**1s**, isolated in the yield range of 45–83% are known (except for **1l**, **1o**, **1r**) and their obtained spectroscopic data matched with the literature.[3-9]

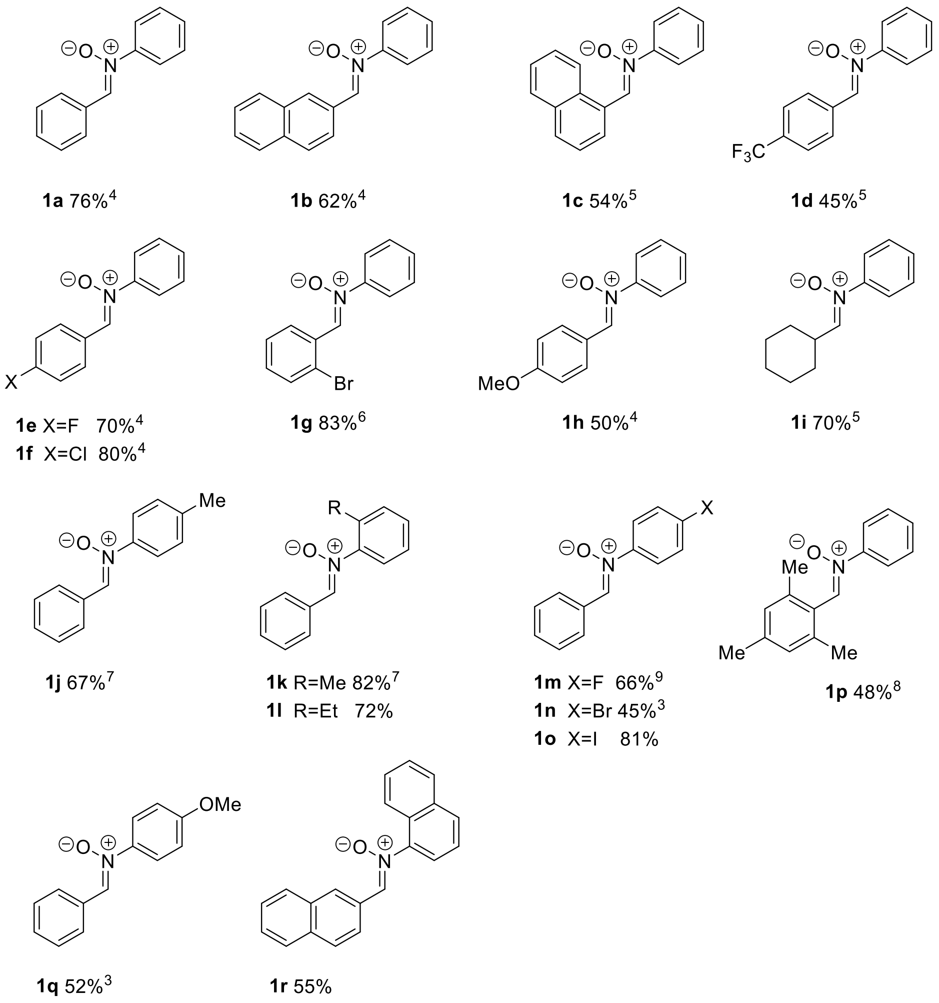

Figure S1. Synthesized nitrones **1a** to **1r**.

*Synthesis of N-(2-ethylphenyl)-1-phenylmethanimine oxide (***1l***)*

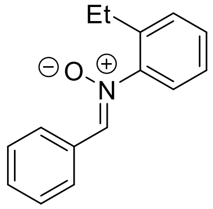

Synthesized in accordance with *General procedure a*, using 1-Ethyl-2-nitrobenzene (1.1 mL, 8 mmol), benzaldehyde (0.9 mL, 8.8 mmol), NH_4Cl (513 mg, 9.6 mmol), Zn dust (1.1 g, 16 mmol). The crude compound was recrystallized from ethanol to afford the product (**1l**) as a white solid. Yield: 1.3 g, 5.8 mmol, 72%.

^{1}H NMR (500 MHz, $CDCl_3$, 298 K) δ: 8.29–8.27 (m, 2H, Ar–CH), 7.51 (s, 1H, CH), 7.41–7.40 (m, 3H, Ar–CH), 7.33–7.27 (m, 3H, Ar–CH), 7.21–7.18 (m, 1H, Ar–CH), 2.72 (q, *J* = 7.6 Hz, 2H, CH_2), 1.20 (t, *J* = 7.6 Hz, 3H, CH_3); ^{13}C NMR (126 MHz, $CDCl_3$, 298 K) δ: 148.4, 137.8, 137.7, 130.9, 130.5, 129.8, 129.7, 128.8, 128.7, 126.7, 123.6, 23.8 (CH_2), 15.1 (CH_3); IR ν_{max} (cm^{-1}): 2978, 1570, 1487, 1440, 1402, 1301, 1184; HRMS (ES+) $[M+H]^+$ calculated for $[C_{15}H_{16}NO]^+$: 226.1232, found 226.1228.

*Synthesis of N-(4-iodophenyl)-1-phenylmethanimine oxide (***1o***)*

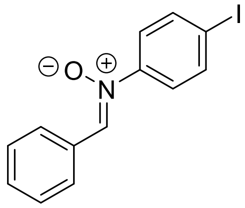

Synthesized in accordance with *General procedure a*, using 1-Iodo-4-nitrobenzene (2 g, 8 mmol), benzaldehyde (0.9 mL, 8.8 mmol), NH_4Cl (513 mg, 9.6 mmol), Zn dust (1.1 g, 16 mmol). The crude compound was recrystallized from ethanol to afford the product (**1o**) as a purple solid. Yield: 2.1 g, 6.5 mmol, 81%.

^{1}H NMR (400 MHz, $CDCl_3$, 298 K) δ: 8.37 (d, *J* = 3.6 Hz, 2H, Ar–CH), 7.90 (s, 1H, CH), 7.79 (d, *J* = 8.2 Hz, 2H, Ar–CH), 7.54–7.46 (m, 5H, Ar–CH); ^{13}C NMR (101 MHz, $CDCl_3$, 298 K) δ: 148.6, 138.3, 134.7, 131.3, 130.4, 129.2, 128.8, 123.5, 95.6; IR ν_{max} (cm^{-1}): 3053, 2981, 1583, 1546, 1477, 1444, 1413, 1190, 1066; HRMS (ES+) $[M+H]^+$ calculated for $[C_{13}H_{11}NOI]^+$: 323.9885, found 323.9888.

*Synthesis of N-(naphthalen-1-yl)-1-(naphthalen-2-yl)methanimine oxide (***1r***)*

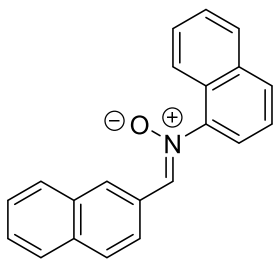

Synthesized in accordance with *General procedure a*, using 1-Nitronaphtalene (1.4 g, 8 mmol), 2- Naphthaldehyde (1.4 g, 8.8 mmol), NH_4Cl (513 mg, 9.6 mmol), Zn dust (1.1 g, 16 mmol). The crude compound was recrystallized from ethyl acetate to afford the product (**1r**) as a yellow solid. Yield: 1.3 g, 4.4 mmol, 55%.

^{1}H NMR (500 MHz, $CDCl_3$, 298 K) δ: 9.52 (s, 1H, Ar–CH), 8.20–8.19 (m, 1H, Ar–CH), 8.03–7.87 (m, 7H, Ar–CH and CH), 7.67 (d, *J* = 7.2 Hz, 1H, Ar–CH), 7.59–7.52 (m, 5H, Ar–CH);

^{13}C NMR (126 MHz, $CDCl_3$, 298 K) δ: 146.2, 138.9, 134.6, 134.4, 133.3, 130.1, 129.6, 129.2, 128.3, 128.2, 127.9, 127.84, 127.82, 127.7, 127.2, 127.1, 126.8, 126.2, 125.0, 122.9, 120.5; IR ν_{max} (cm^{-1}): 3057, 1693, 1598, 1556, 1506, 1402, 1388, 1379, 1361, 1350, 1267, 1228, 1217, 1170, 1163, 1128, 1116, 1060, 1022; HRMS (ES+) $[M+H]^+$ calculated for $[C_{21}H_{16}NO]^+$: 298.1232; found 298.1227.

*Synthesis of N-methyl-1-phenylmethanimine oxide (**1s**)*[10]

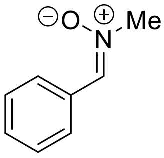

Benzaldehyde (1.9 mL, 18.8 mmol, 1 equiv.) was added at room temperature into a solution of N-methyl hydroxylamine hydrochloride (1.7 g, 20.7 mmol, 1.1 equiv.) in [0.6 M] aqueous NaOH (903 mg, 22.6 mmol, 1.2 equiv.) and the reaction was stirred for additional 1 hour. Subsequently, the organics were extracted by CH_2Cl_2 (3 × 20 mL), washed with brine (1 × 20 mL), dried over $MgSO_4$, and concentrated in vacuo. The crude compound was recrystallized from ethyl acetate/hexane to afford the product (**1s**) as a white solid. Yield: 2 g, 14.8 mmol, 78%. The obtained spectroscopic data matched with literature.

1.2.2 Synthesis of Vinyldiazo Esters

General procedure b: α-diazo-β-hydroxy ester (1 equiv.) was dissolved in 3 mL/mmol of anhydrous triethylamine. The reaction mixture was cooled to 0 °C and trifluoroacetic anhydride (1.4 equiv.) was added dropwise. The reaction was allowed to warm to room temperature, and was stirred for 24 hours. After completion, the reaction was diluted with ethyl acetate (30 mL), washed with water (3 × 25 mL), and dried over Na_2SO_4. The solvent was evaporated at room temperature using compressed air and the crude product was purified *via* column chromatography using silica gel (Merck, 60 Å, 230–400 mesh particle size) and hexane/ethyl acetate as eluent.

*Synthesis of ethyl 2-diazobut-3-enoate (**2a**)*[11]

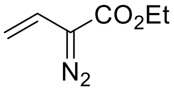

Synthesized in accordance with *General procedure b*, using ethyl 2-diazo-3-hydroxybutanoate (950 mg, 6 mmol, 1 equiv.), trifluoroacetic anhydride (1.2 mL, 8.4 mmol, 1.4 equiv.) and 20 mL of dry triethylamine. The crude compound was purified via column chromatography using hexane/ethyl acetate (90:10 v/v) as eluent. The compound (**2a**) was obtained as a red oil. Yield: 520 mg, 3.7 mmol, 62%. The obtained spectroscopic data matched with literature.

*Synthesis of methyl (E)-2-diazopent-3-enoate (***2b***)*[12]

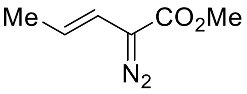

Synthesized in accordance with *General procedure b*, using methyl 2-diazo-3-hydroxypentanoate (1.0 g, 6 mmol, 1 equiv.), trifluoroacetic anhydride (1.2 mL, 8.4 mmol, 1.4 equiv.) and 20 mL of dry triethylamine. The crude compound was purified via column chromatography using hexane/ethyl acetate (90:10 v/v) as eluent. The compound (**2b**) was obtained as a red oil. Yield: 650 mg, 4.2 mmol, 70%. The obtained spectroscopic data matched with literature.

*Synthesis of methyl (E)-2-diazo-4-phenylbut-3-enoate (***2c***)*[12]

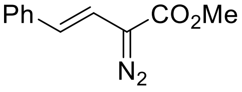

1,8-Diazabicyclo[5.4.0]undec-7-ene (1.1 mL, 7.2 mmol, 1.2 equiv.) was added dropwise into a solution of (3*E*)-4-phenylbut-3-enoic acid methyl ester (1.0 mL, 6 mmol., 1 equiv.), and 4-acetamidobenzenesulfonyl azide (2.0 g, 8.3 mmol, 1.4 equiv.) in 40 mL of anhydrous acetonitrile at 0 °C. Upon complete addition, the reaction mixture was stirred for an additional 2 hours at 0 °C and subsequently quenched with saturated NH_4Cl (25 mL). The organics were extracted with diethyl ether (3 × 30 mL), washed with brine (1 × 40 mL), dried over $MgSO_4$, and concentrated *in vacuo*. The crude product was purified *via* column chromatography using silica gel (Merck, 60 Å, 230–400 mesh particle size) and hexane/ethyl acetate (92:8 v/v) as eluent. The compound (**2c**) was obtained as a red solid. Yield: 920 mg, 4.5 mmol, 76%. The obtained spectroscopic data matched with literature.

1.2.3 Synthesis of Enoldiazo Esters

General procedure c: Following a slightly modified literature reported procedure,[13] α-diazo-β-keto ester (1 equiv.) and anhydrous triethylamine (1.2 equiv.) were dissolved in 5 mL/mmol of anhydrous dichloromethane. The reaction mixture was cooled to 0 °C and *tert*-butyldimethylsilyl trifluoromethanesulfonate (1.1 equiv.) was added dropwise. The reaction was allowed to warm to room temperature and was stirred for up to 24 hours. After completion (monitored by TLC), the reaction was diluted with ethyl acetate (30 mL) and quenched with saturated $NaHCO_3$. The organic layer was washed with water (2 × 40 mL), brine (1 × 40 mL), dried over $MgSO_4$, and concentrated *in vacuo*. The crude compound was purified *via* a silica plug using silica gel (Merck, 60 Å, 230–400 mesh particle size) and hexane/ethyl acetate as eluent.

*Synthesis of ethyl 3-((tert-butyldimethylsilyl)oxy)-2-diazobut-3-enoate (**4a**)*[14]

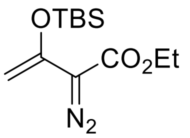

Synthesized in accordance with *General procedure c*, using methyl 2-diazo-3-oxobutanoate (1.4 g, 10 mmol, 1 equiv.), triethylamine (1.7 mL, 12 mmol, 1.2 equiv.), and *tert*-butyldimethylsilyl trifluoromethanesulfonate (2.5 mL, 11 mmol, 1.1 equiv.) in 50 mL of dry dichloromethane. The crude compound was purified *via* a silica plug using hexane/ethyl acetate (95:5 v/v) as eluent. The compound (**4a**) was obtained as a red oil. Yield: 2.4 g, 8.8 mmol, 88%. The obtained spectroscopic data matched with literature.

*Synthesis of methyl (Z)-3-((tert-butyldimethylsilyl)oxy)-2-diazopent-3-enoate (**4b**)*[15]

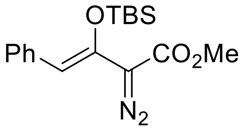

Synthesized in accordance with *General procedure c*, using methyl 2-diazo-3-oxopentanoate (0.8 g, 4.9 mmol, 1 equiv.), triethylamine (0.8 mL, 5.9 mmol, 1.2 equiv.), and *tert*-butyldimethylsilyl trifluoromethanesulfonate (1.2 mL, 5.4 mmol, 1.1 equiv.) in 20 mL of dry dichloromethane. The crude compound was purified *via* a silica plug using hexane/ethyl acetate (98:2 v/v) as eluent. The compound (**4b**) was obtained as a red oil. Yield: 1.0 g, 3.7 mmol, 75%. The obtained spectroscopic data matched with literature.

*Synthesis of methyl (Z)-3-((tert-butyldimethylsilyl)oxy)-2-diazo-4-phenylbut-3-enoate (**4c**)*[13]

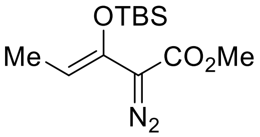

Synthesized in accordance with *General procedure c*, methyl 2-diazo-3-oxo-4-phenylbutanoate (1.6 g, 7.5 mmol, 1 equiv.), triethylamine (1.3 mL, 9.0 mmol, 1.2 equiv.), and *tert*-butyldimethylsilyl trifluoromethanesulfonate (1.9 mL, 8.3 mmol, 1.1 equiv.) in 35 mL of dry dichloromethane. The crude compound was purified via column chromatography using hexane/ethyl acetate (98:2 v/v) as eluent. The compound (**4c**) was obtained as an orange solid. Yield: 2.4 g, 7.2 mmol, 96%. The obtained spectroscopic data matched with literature.

*Synthesis of methyl ethyl 2-diazo-3-methylbut-3-enoate (**2d**)*[13]

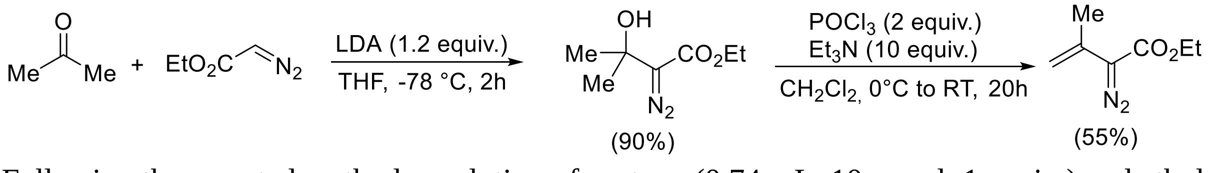

Following the reported method, a solution of acetone (0.74 mL, 10 mmol, 1 equiv.) and ethyl diazo acetate (15% in toluene, 8.5 mL, 10 mmol, 1 equiv.) in THF (20 mL) was cooled to -78 °C. To this solution, lithium diisopropylamide (1M in THF, 12 ml, 12 mmol, 1.2 equiv.) was added dropwise. The resulting orange solution was quenched with water after stirring at -78 °C for 2 hours. The crude reaction mixture was extracted with EtOAc (3 × 20 mL) after which the

organic layers were combined, treated with brine, and dried over $MgSO_4$. The solvent was then evaporated using a stream of compressed air giving 1.58 g of ethyl 2-diazo-3-hydroxy-3-methylbutanoate as yellow oil (92%). The intermediate was stored at -50 °C until needed for the second step.

A solution of ethyl 2-diazo-3-hydroxy-3-methylbutanoate (0.34 g, 2 mmol, 1 equiv.,) and triethylamine (2.8 mL, 20 mmol, 10 equiv.) in CH_2Cl_2 (20 mL) was cooled to 0 °C. Separately, $POCl_3$ (0.37 mL, 4 mmol, 2 equiv.) was dissolved in 8 mL of CH_2Cl_2 which was then added dropwise to the reaction mixture. The reaction was stirred at 0 °C for an additional 5 min before letting it warm to room temperature. The progress of the reaction was checked by TLC (~20 hours). After completion, the reaction mixture was filtered and the solvent removed. The crude product was purified by flash chromatography using 9:1 Hexane:EtOAc to give ethyl 2-diazo-3-methylbut-3-enoate as a yellow oil (170 mg, 55%). The product was immediately used for the cycloaddition reaction due to its reported instability.

2.0 Synthesis of Products

General procedure d: Tris(pentafluorophenyl)borane [$B(C_6F_5)_3$] (20 mol%), nitrone (1 equiv.) and 4 Å MS were charged in a microwave vial and then dissolved in 0.5 mL of anhydrous toluene. The mixture was stirred for 5 minutes at room temperature. Subsequently, vinyldiazo ester (2 equiv.), dissolved in 0.8 mL of anhydrous toluene, was added *via* syringe pump for 0.5 hour into the reaction mixture. After complete addition, the reaction was heated to 40 °C and stirred in the dark for up to 24 hours. After completion, all volatiles were evaporated, and the crude compound was purified *via* preparative thin layer chromatography using hexane/ethyl acetate as eluent.

General procedure e: Tris(pentafluorophenyl)borane [$B(C_6F_5)_3$] (10 mol%), nitrone (1 equiv.) and 4 Å MS were charged in a microwave vial and then dissolved in 0.5 mL of anhydrous toluene. The mixture was stirred for 5 minutes at 0°C. Subsequently, enoldiazo ester (2 equiv.) was dissolved in 0.8 mL of anhydrous toluene and was added *via* syringe pump for 0.5 hour into the reaction mixture. After complete addition, the reaction was allowed to reach room temperature over the course of 3 hours and stirred for up to 24 hours. After completion, all volatiles were evaporated, and the crude compound was purified *via* preparative thin layer chromatography using hexane/ethyl acetate as eluent.

2.1 Synthesis and Spectral Characterization of Isoxazolidine-Derived Diazo Products

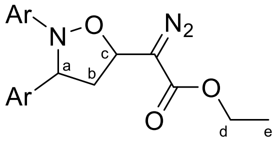

Figure S2. Labelling of isoxazolidine-derived diazo products

The characteristic protons and carbons were assigned according to the labelling in the general structure in Figure S2. Peak assignment has been inferred from 2D-NMR analysis on compounds **3p**.

*Synthesis of ethyl 2-diazo-2-(2,3-diphenylisoxazolidin-5-yl)acetate (**3a**)*

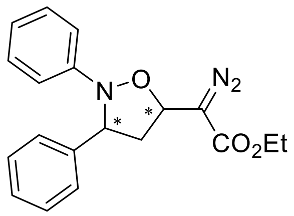

Synthesized in accordance with *General procedure d*, using $B(C_6F_5)_3$ (10 mg, 0.02 mmol), nitrone **1a** (20 mg, 0.1 mmol), vinyldiazo ester **2a** (28 mg, 0.2 mmol) in toluene to afford **3a**. All volatiles were removed *in vacuo* and the crude compound was purified *via* preparative thin layer chromatography using silica gel and hexane/ethyl acetate (90:10 v/v) as eluent. The desired product (**3a**) was obtained as an inseparable mixture of diastereoisomers (91:9) as a yellow oil. Yield: 25 mg, 0.07 mmol, 74 %.

^{1}H NMR (500 MHz, $CDCl_3$, 298 K) δ: 7.50 –7.47 (m, Ar–CH), 7.41–7.37 (m, Ar–CH), 7.32–7.25 (m, Ar–CH), 7.22–7.19 (m, Ar–CH), 7.05–7.03 (m, Ar–CH), 6.98–6.92 (m, Ar–CH), 5.36 (t, *J* = 7.3 Hz, 1H, CH^c, major isomer), 5.23 (dd, *J* = 8.8, 6.8 Hz, CH^c, minor isomer), 4.87–4.84 (m, CH^a, minor isomer), 4.69 (dd, *J* = 8.3, 5.5 Hz, 1H, CH^a, major isomer), 4.30–4.24 (m, $CH_2{}^d$), 3.08 (ddd, *J* = 12.5, 8.2, 6.8 Hz, CH^b, minor isomer), 2.71–2.60 (m, 2H, CH^b, major isomer), 2.26 (ddd, *J* = 12.5, 8.8, 6.6 Hz, CH^b, minor isomer), 1.30 (t, *J* = 7.1 Hz, $CH_3{}^e$); ^{13}C NMR (126 MHz, $CDCl_3$, 298 K) δ: 165.3 (C=O), 151.6, 150.7, 141.9, 141.2, 129.1, 129.06, 129.03, 128.8, 127.8, 127.7, 126.7, 126.3, 122.3, 122.2, 115.6, 114.8, 72.9, 72.7, 70.9, 69.0, 61.3, 42.9, 41.7, 14.6; IR ν_{max} (cm^{-1}): 2981, 2935, 2094 ($C=N_2$), 1691 (C=O), 1597, 1489, 1450, 1371, 1298, 1259, 1244, 1109, 1026; HRMS (ES+) $[M+H]^+$ calculated for $[C_{19}H_{20}N_3O_3]^+$: 338.1505, found: 338.1504.

*Synthesis of ethyl 2-diazo-2-(3-(naphthalen-2-yl)-2-phenylisoxazolidin-5-yl)acetate (**3b**)*

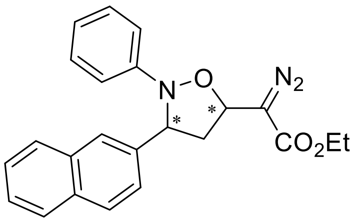

Synthesized in accordance with *General procedure d*, using $B(C_6F_5)_3$ (10 mg, 0.02 mmol), nitrone **1b** (25 mg, 0.1 mmol), vinyldiazo ester **2a** (28 mg, 0.2 mmol) in toluene to afford **3b**. All volatiles were removed *in vacuo* and the crude compound was purified *via* preparative thin layer chromatography using silica gel and hexane/ethyl acetate (90:10 v/v) as eluent. The desired product (**3b**) was obtained as a yellow oil. Yield: 33 mg, 0.08 mmol, 85 %.

^{1}H NMR (500 MHz, $CDCl_3$, 298 K) δ: 7.96 (s, 1H, Ar–CH), 7.89–7.83 (m, 3H, Ar–CH), 7.61 (dd, *J* = 8.5, 1.8 Hz, 1H, Ar–CH), 7.52–7.48 (m, 2H, Ar–CH), 7.22–7.18 (m, 2H, Ar–CH), 7.01–6.99 (m, 2H, Ar–CH), 6.95– 6.92 (m, 1H, Ar–CH), 5.42 (t, *J* = 7.3 Hz, 1H, CH^c), 4.84 (dd, *J* = 8.5, 5.7 Hz, 1H, CH^a), 4.28 (qd, *J* = 7.1, 1.6 Hz, 2H, CH_2^d), 2.78–2.68 (m, 2H, CH^b), 1.30 (t, *J* = 7.1 Hz, 3H, CH_3^e); ^{13}C NMR (126 MHz, $CDCl_3$, 298 K) δ: 150.7, 138.6, 133.5, 133.1, 129.0, 128.8, 128.1, 127.8, 126.5, 126.2, 125.7, 124.6, 122.3, 115.6, 72.9 (CH^c), 69.3 (CH^a), 61.4 (CH_2^d), 41.8 (CH_2^b), 14.6 (CH_3^e); IR ν_{max} (cm^{-1}): 3059, 2983, 2096 ($C{=}N_2$), 1693 (C=O), 1597, 1489, 1373, 1300, 1242, 1109, 1018; HRMS (ES+) $[M+H]^+$ calculated for $[C_{23}H_{22}N_3O_3]^+$: 388.1661, found 388.1660.

*Synthesis of ethyl 2-diazo-2-(3-(naphthalen-1-yl)-2-phenylisoxazolidin-5-yl)acetate (**3c**)*

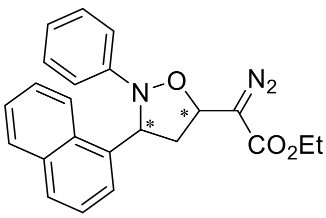

Synthesized in accordance with *General procedure d*, using $B(C_6F_5)_3$ (10 mg, 0.02 mmol), nitrone **1c** (25 mg, 0.1 mmol), vinyldiazo ester **2a** (28 mg, 0.2 mmol) in toluene to afford **3c**. All volatiles were removed *in vacuo* and the crude compound was purified *via* preparative thin layer chromatography using silica gel and hexane/ethyl acetate (90:10 v/v) as eluent. The desired product (**3c**) was obtained as an inseparable mixture of diastereoisomers (68:32) as a yellow oil. Yield: 32 mg, 0.08 mg, 83%.

^{1}H NMR (500 MHz, $CDCl_3$, 298 K) δ: 8.07 (d, *J* = 8.6 Hz, Ar–CH), 8.02 (d, *J* = 8.6 Hz, Ar–CH), 7.98–7.94 (m, Ar–CH), 7.85–7.83 (m, Ar–CH), 7.59–7.49 (m, Ar–CH), 7.28 (dd, *J* = 8.8, 7.3 Hz, Ar–CH), 7.21 (dd, *J* = 8.8, 7.3 Hz, Ar–CH), 7.09 (dd, *J* = 8.8, 1.1 Hz, Ar–CH), 6.99– 6.92 (m, Ar–CH), 5.54 (dd, *J* = 8.5, 6.3 Hz, CH^c, minor isomer), 5.42 (dd, *J* = 8.9, 4.7 Hz, 1H, CH^c, major isomer), 5.37 (dd, *J* = 8.1, 6.7 Hz, CH^a), 4.28 (qd, *J* = 7.1, 2.4 Hz, CH_2^d), 3.33 (ddd, *J* = 12.6, 8.5, 7.0 Hz, CH^b, minor isomer), 2.91 (dt, *J* = 12.5, 8.5 Hz, 1H, CH^b, major isomer), 2.62 (ddd, *J* = 12.5, 6.8, 4.7 Hz, 1H, CH^b, major isomer), 2.26 (ddd, *J* = 12.6, 8.7, 6.3

Hz, CH[b], minor isomer), 1.31 (t, J = 7.1 Hz, CH_3[e]); ^{13}C NMR (126 MHz, $CDCl_3$, 298 K) δ: 165.3 (C=O), 151.8, 150.9, 137.2, 136.4, 134.19, 134.16, 130.16, 130.10, 129.38, 129.34, 129.2, 128.8, 128.29, 128.28, 126.5, 126.4, 126.09, 126.06, 125.8, 125.7, 124.3, 123.7, 122.97, 122.90, 122.1, 122.0, 115.2, 114.6, 73.0, 72.9, 68.6, 66.4, 61.3, 42.1, 40.4, 14.6; IR ν_{max} (cm^{-1}): 3059, 2980, 2096 (C=N_2), 1689 (C=O), 1597, 1489, 1396, 1373, 1300, 1259, 1244, 1114; HRMS (ES+) $[M+H]^+$ calculated for $[C_{23}H_{22}N_3O_3]^+$: 388.1661; found 388.1656.

*Synthesis of ethyl 2-diazo-2-(2-phenyl-3-(4-(trifluoromethyl)phenyl)isoxazolidin-5-yl)acetate (**3d**)*

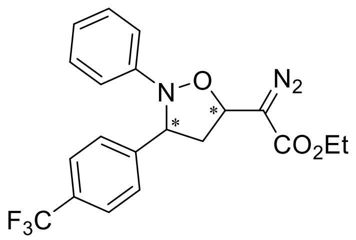

Synthesized in accordance with *General procedure d*, using $B(C_6F_5)_3$ (10 mg, 0.02 mmol), nitrone **1d** (27 mg, 0.1 mmol), vinyldiazo ester **2a** (28 mg, 0.2 mmol) in toluene to afford **3d**. All volatiles were removed *in vacuo* and the crude compound was purified *via* preparative thin layer chromatography using silica gel and hexane/ethyl acetate (90:10 v/v) as eluent. The desired product (**3d**) was obtained as a yellow oil. Yield: 31 mg, 0.07 mmol, 76%,

^{1}H NMR (500 MHz, $CDCl_3$, 298 K) δ: 7.65–7.61 (m, 4H, Ar–CH), 7.23 (dd, J = 8.7, 7.4 Hz, 2H, Ar–CH), 6.98–6.92 (m, 3H, Ar–CH), 5.34 (t, J = 7.4 Hz, 1H, CH[c]), 4.78 (dd, J = 8.6, 5.0 Hz, 1H, CH[a]), 4.27 (qd, J = 7.1, 1.2 Hz, 2H, CH_2[d]), 2.73 (dt, J = 12.8, 8.2 Hz, 1H, CH[b]), 2.59 (ddd, J = 12.6, 7.3, 5.1 Hz, 1H, CH[b]), 1.29 (t, J = 7.1 Hz, 3H, CH_3[e]); ^{13}C NMR (126 MHz, $CDCl_3$, 298 K) δ: 165.2 (C=O), 150.3, 145.3, 145.3, 130.2 (q, $J_{C–F}$ = 32.5 Hz), 128.9, 127.1, 126.0 (q, $J_{C–F}$ = 3.8 Hz), 122.6, 115.5, 73.1 (CH[c]), 68.5 (CH[a]), 61.4 (CH_2[d]), 41.3 (CH_2[b]), 14.6 (CH_3[e]); ^{19}F NMR (471 MHz, $CDCl_3$, 298 K) δ: -62.51 (Ar–CF_3); IR ν_{max} (cm^{-1}): 2987, 2939, 2096 (C=N_2), 1691 (C=O), 1597, 1489, 1373, 1323, 1301, 1165, 1120, 1109, 1066, 1016; HRMS (ES+) $[M+H]^+$ calculated for $[C_{20}H_{19}N_3O_3F_3]^+$: 406.1379, found 406.1375.

*Synthesis of ethyl 2-diazo-2-(3-(4-fluorophenyl)-2-phenylisoxazolidin-5-yl)acetate (**3e**)*

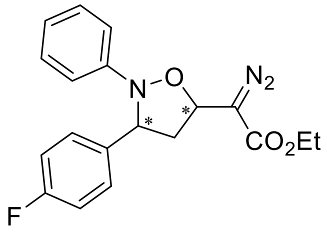

Synthesized in accordance with *General procedure d*, using $B(C_6F_5)_3$ (10 mg, 0.02 mmol), nitrone **1e** (22 mg, 0.1 mmol), vinyldiazo ester **2a** (28 mg, 0.2 mmol) in toluene to afford **3e**. All volatiles were removed *in vacuo* and the crude compound was purified *via* preparative thin layer chromatography using silica gel and hexane/ethyl acetate (90:10 v/v) as eluent. The desired product (**3e**) was obtained as a yellow oil. Yield: 29 mg, 0.08 mmol, 82%.

^{1}H NMR (400 MHz, $CDCl_3$, 298 K) δ: 7.47–7.44 (m, 2H, Ar–CH), 7.23–7.19 (m, 2H, Ar–CH), 7.06–7.04 (m, 2H, Ar–CH), 6.97–6.93 (m, 3H, Ar–CH), 5.34 (t, J = 7.4 Hz, 1H, CH^c), 4.67 (dd, J = 8.3, 5.3 Hz, 1H, CH^a), 4.27 (qd, J = 7.1, 1.0 Hz, 2H, $CH_2{}^d$), 2.70–2.55 (m, 2H, $CH_2{}^b$), 1.29 (t, J = 7.1 Hz, 3H, $CH_3{}^e$); ^{13}C NMR (101 MHz, $CDCl_3$, 298 K) δ: 162.4 (d, $J_{C\text{–}F}$ = 246.2 Hz), 150.4, 136.8 (d, $J_{C\text{–}F}$ = 3.2 Hz), 128.8, 128.4 (d, $J_{C\text{–}F}$ = 8.1 Hz), 122.5, 116.0, 115.7, 72.9 (CH^a), 68.4 (CH^c), 61.4 ($CH_2{}^d$), 41.6 ($CH_2{}^b$), 14.6 ($CH_3{}^e$); ^{19}F NMR (376 MHz, $CDCl_3$, 298 K) δ: -114.67; IR ν_{max} (cm^{-1}): 2987, 2931, 2096 ($C{=}N_2$), 1693 (C=O), 1598, 1508, 1489, 1373, 1300, 1261, 1224, 1174, 1157, 1112, 1097, 1014; HRMS (ES+) $[M+H]^+$ calculated for $[C_{19}H_{19}N_3O_3F]^+$: 356.1410, found 356.1411.

*Synthesis of ethyl 2-diazo-2-(3-(4-fluorophenyl)-2-phenylisoxazolidin-5-yl)acetate (**3f**)*

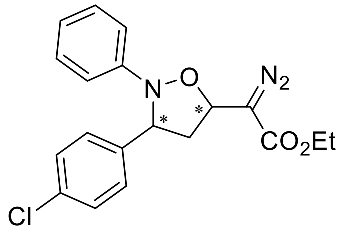

Synthesized in accordance with *General procedure d*, using $B(C_6F_5)_3$ (10 mg, 0.02 mmol), nitrone **1f** (23 mg, 0.1 mmol), vinyldiazo ester **2a** (28 mg, 0.2 mmol) in toluene to afford **3f**. All volatiles were removed *in vacuo* and the crude compound was purified *via* preparative thin layer chromatography using silica gel and hexane/ethyl acetate (90:10 v/v) as eluent. The desired product (**3f**) was obtained as a yellow oil. Yield: 30 mg, 0.08 mmol, 84%.

^{1}H NMR (400 MHz, $CDCl_3$, 298 K) δ: 7.43 (d, J = 8.2 Hz, 2H, Ar–CH), 7.35 (d, J = 8.6 Hz, 2H, Ar–CH), 7.23–7.19 (m, 2H, Ar–CH), 6.97–6.92 (m, 3H, Ar–CH), 5.33 (t, J = 7.4 Hz, 1H, CH^c), 4.67 (dd, J = 8.4, 5.2 Hz, 1H, CH^a), 4.26 (q, J = 7.1 Hz, 2H, $CH_2{}^d$), 2.71–2.53 (m, 2H, $CH_2{}^b$), 1.29 (t, J = 7.1 Hz, 3H, $CH_3{}^e$); ^{13}C NMR (101 MHz, $CDCl_3$, 298 K) δ: 150.3, 139.6, 133.6, 129.1, 128.9, 128.2, 122.5, 115.7, 72.9 (CH^c), 68.4 (CH^a), 61.4 ($CH_2{}^d$), 41.5 ($CH_2{}^b$), 14.6 ($CH_3{}^e$); IR ν_{max} (cm^{-1}): 2985, 2937, 2096 ($C{=}N_2$), 1693 (C=O), 1597, 1489, 1398, 1373, 1300, 1261, 1209, 1172, 1111, 1089, 1056, 1014; HRMS (ES+) $[M+H]^+$ calculated for $[C_{19}H_{19}N_3O_3Cl]^+$: 372.1115, found 372.1115.

*Synthesis of ethyl 2-(3-(2-bromophenyl)-2-phenylisoxazolidin-5-yl)-2-diazoacetate (**3g**)*

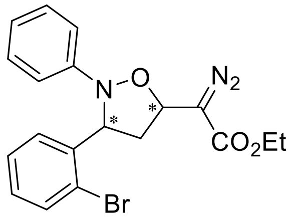

Synthesized in accordance with *General procedure d*, using $B(C_6F_5)_3$ (10 mg, 0.02 mmol), nitrone **1g** (28 mg, 0.1 mmol), vinyldiazo ester **2a** (28 mg, 0.2 mmol) in toluene to afford **3g**. All volatiles were removed *in vacuo* and the crude compound was purified *via* preparative thin layer chromatography using silica gel and hexane/ethyl acetate (90:10 v/v) as eluent. The desired product (**3g**) was obtained as a yellow oil. Yield: 27, mg, 0.06 mmol, 65%.

^{1}H NMR (400 MHz, $CDCl_3$, 298 K) δ: 7.81 (d, *J* = 7.8 Hz, 1H, Ar–CH), 7.60 (d, *J* = 8.0 Hz, 1H, Ar–CH), 7.35 (t, *J* = 7.6 Hz, 1H, Ar–CH), 7.24–7.16 (m, 3H, Ar–CH), 6.96–6.92 (m, 3H, Ar–CH), 5.31 (t, *J* = 7.5 Hz, 1H, CH^c), 5.10 (dd, *J* = 8.6, 4.3 Hz, 1H, CH^a), 4.27 (q, *J* = 7.1 Hz, 2H, $CH_2{}^d$), 2.79–2.72 (m, 1H, CH^b), 2.54–2.48 (m, 1H, CH^b), 1.29 (t, *J* = 7.1 Hz, 3H, $CH_3{}^e$); ^{13}C NMR (101 MHz, $CDCl_3$, 298 K) δ: 150.6, 140.3, 132.9, 129.2, 128.9, 128.6, 128.2, 122.16, 122.10, 114.9, 73.1 (CH^c), 68.3 (CH^a), 61.4 ($CH_2{}^d$), 39.7 ($CH_2{}^b$), 14.6 ($CH_3{}^e$); IR ν_{max} (cm^{-1}): 2094 ($C{=}N_2$), 1693 (C=O), 1597, 1489, 1257, 1244, 1022; HRMS (ES+) $[M+H]^+$ calculated for $[C_{19}H_{19}N_3O_3Br]^+$: 416.0610, found 416.0610.

*Synthesis of ethyl 2-diazo-2-(3-(4-methoxyphenyl)-2-phenylisoxazolidin-5-yl)acetate (**3h**)*

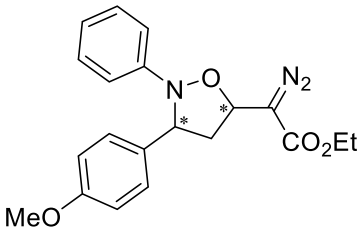

Synthesized in accordance with *General procedure d*, using $B(C_6F_5)_3$ (10 mg, 0.02 mmol), nitrone **1h** (23 mg, 0.1 mmol), vinyldiazo ester **2a** (28 mg, 0.2 mmol) in toluene to afford **3h**. All volatiles were removed *in vacuo* and the crude compound was purified *via* preparative thin layer chromatography using silica gel and hexane/ethyl acetate (90:10 v/v) as eluent. The desired product (**3h**) was obtained as an off-white solid. Yield: 25 mg, 0.06 mmol, 68%.

^{1}H NMR (500 MHz, $CDCl_3$, 298 K) δ: 7.39 (d, *J* = 8.9 Hz, 2H, Ar–CH), 7.21–7.18 (m, 2H, Ar–CH), 6.96–6.90 (m, 5H, Ar–CH), 5.35 (t, *J* = 7.3 Hz, 1H, CH^c), 4.61 (dd, *J* = 8.1, 5.7 Hz, 1H, CH^a), 4.29–4.25 (m, 2H, $CH_2{}^d$), 3.81 (s, 3H, Ar–OCH_3), 2.67–2.57 (m, 2H, $CH_2{}^b$), 1.30 (t, *J* = 7.1 Hz, 3H, $CH_3{}^e$); ^{13}C NMR (126 MHz, $CDCl_3$, 298 K) δ: 165.4 (C=O), 159.2, 150.6, 133.0, 128.7, 128.0, 122.4, 115.8, 114.3, 72.7(CH^c), 68.7 (CH^a), 61.3 ($CH_2{}^d$), 55.4 (Ar–OCH_3), 41.8 ($CH_2{}^b$), 14.6 ($CH_3{}^e$); IR ν_{max} (cm^{-1}): 2983, 2929, 2096 ($C{=}N_2$), 1693 (C=O), 1612, 1598, 1512, 1373, 1300, 1246, 1174, 1111, 1033; HRMS (ES+) $[M+H]^+$ calculated for $[C_{20}H_{22}N_3O_4]^+$: 368.1610, found 368.1609.

*Synthesis of ethyl 2-(3-cyclohexyl-2-phenylisoxazolidin-5-yl)-2-diazoacetate (**3i**)*

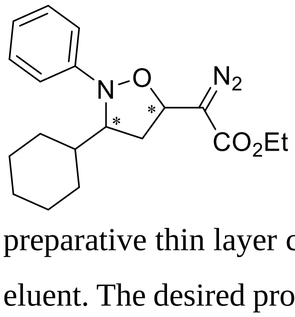

Synthesized in accordance with *General procedure d*, using $B(C_6F_5)_3$ (10 mg, 0.02 mmol), nitrone **1i** (20 mg, 0.1 mmol), vinyldiazo ester **2a** (28 mg, 0.2 mmol) in toluene to afford **3i**. All volatiles were removed *in vacuo* and the crude compound was purified *via* preparative thin layer chromatography using silica gel and hexane/ethyl acetate (90:10 v/v) as eluent. The desired product (**3i**) was obtained as a yellow solid. Yield: 30 mg, 0.08 mmol, 87%

^{1}H NMR (500 MHz, $CDCl_3$, 298 K) δ: 7.25–7.22 (m, 2H, Ar–CH), 6.99– 6.97 (m, 2H, Ar–CH), 6.91–6.88 (m, 1H, Ar–CH), 5.31 (t, *J* = 7.3 Hz, 1H, CH^c), 4.25 (q, *J* = 7.1 Hz, 2H, CH_2^d), 3.56 (td, *J* = 8.0, 3.2 Hz, 1H, CH^a), 2.48 (ddd, *J* = 13.1, 7.6, 3.3 Hz, 1H, CH), 2.07–1.98 (m, 2H, CH_2), 1.82–1.78 (m, 3H, CH), 1.73– 1.70 (m, 1H, CH), 1.59– 1.55 (m, 2H, CH), 1.28 (t, *J* = 7.1 Hz, 3H, CH_3^e), 1.26–1.05 (m, 4H, CH); ^{13}C NMR (126 MHz, $CDCl_3$, 298 K) δ: 165.6 (C=O), 152.6, 128.9, 121.3, 114.2, 74.9, 70.6, 61.3, 41.1, 33.4, 30.8, 29.8, 26.5, 26.2, 26.2, 14.6 (CH_3^e); IR ν_{max} (cm^{-1}): 2924, 2852, 2092 ($C=N_2$), 1693 (C=O), 1597, 1485, 1448, 1251; HRMS (ES+) $[M+H]^+$ calculated for $[C_{19}H_{26}N_3O_3]^+$: 344.1974, found 344.1975.

*Synthesis of ethyl 2-diazo-2-(3-phenyl-2-(p-tolyl)isoxazolidin-5-yl)acetate (**3j**)*

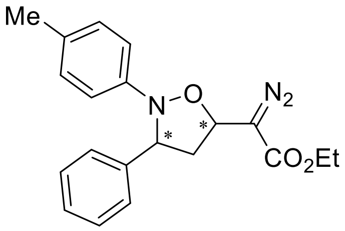

Synthesized in accordance with *General procedure d*, using $B(C_6F_5)_3$ (10 mg, 0.02 mmol), nitrone **1j** (21 mg, 0.1 mmol), vinyldiazo ester **2a** (28 mg, 0.2 mmol) in toluene to afford **3j**. All volatiles were removed *in vacuo* and the crude compound was purified *via* preparative thin layer chromatography using silica gel and hexane/ethyl acetate (90:10 v/v) as eluent. The desired product (**3j**) was obtained as a yellow oil. Yield: 26 mg, 0.07 mmol, 74%.

^{1}H NMR (500 MHz, $CDCl_3$, 298 K) δ: 7.48 (d, *J* = 7.1 Hz, 2H, Ar–CH), 7.37 (t, *J* = 7.5 Hz, 2H, Ar–CH), 7.31–7.28 (m, 1H, Ar–CH), 7.01 (d, *J* = 8.2 Hz, 2H, Ar–CH), 6.87 (d, *J* = 8.5 Hz, 2H, Ar–CH), 5.34 (t, *J* = 7.4 Hz, 1H, CH^c), 4.61 (dd, *J* = 8.1, 5.9 Hz, 1H, CH^a), 4.27 (qd, *J* = 7.1, 1.3 Hz, 2H, CH_2^d), 2.69–2.59 (m, 2H, CH_2^b), 2.25 (s, Ar–CH_3)1.30 (t, *J* = 7.1 Hz, 3H, CH_3^e); ^{13}C NMR (126 MHz, $CDCl_3$, 298 K) δ: 148.1, 141.0, 132.1, 129.3, 128.9, 127.8, 126.9, 116.3, 72.6 (CH_2^c), 69.4 (CH_2^a), 61.3 (CH_2^d), 41.8 (CH_2^b), 20.7 (Ar–CH_3), 14.6 (CH_3^e); IR ν_{max} (cm^{-1}): 2980, 2924, 2094 ($C=N_2$), 1693 (C=O), 1506, 1450, 1373, 1298, 1259, 1109; HRMS (ES+) $[M+H]^+$ calculated for $[C_{20}H_{22}N_3O_3]^+$: 352.1661, found 352.1661.

*Synthesis of ethyl 2-diazo-2-(3-phenyl-2-(o-tolyl)isoxazolidin-5-yl)acetate (**3k**)*

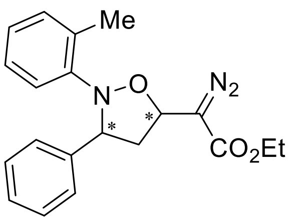

Synthesized in accordance with *General procedure d*, using $B(C_6F_5)_3$ (10 mg, 0.02 mmol), nitrone **1k** (21 mg, 0.1 mmol), vinyldiazo ester **2a** (28 mg, 0.2 mmol) in toluene to afford **3k**. All volatiles were removed *in vacuo* and the crude compound was purified *via* preparative thin layer chromatography using silica gel and hexane/ethyl acetate (90:10 v/v) as eluent. The desired product (**3k**) was obtained as a yellow oil. Yield: 27 mg, 0.07 mmol, 77%.

^{1}H NMR (500 MHz, $CDCl_3$, 298 K) δ: 7.41–7.39 (m, 2H, Ar–CH), 7.30 (t, J = 7.4 Hz, 2H, Ar–CH), 7.25–7.23 (m, 2H, Ar–CH), 7.12–7.08 (m, 2H, Ar–CH), 7.02– 6.98 (m, 1H, Ar–CH), 5.31 (t, J = 7.8 Hz, 1H, CH^c), 4.62 (t, J = 6.9 Hz, 1H, CH^a), 4.28 (qd, J = 7.2, 1.6 Hz, 2H, CH_2^d), 2.70 (t, J = 7.2 Hz, 2H, CH_2^b), 2.17 (s, 3H, Ar–CH_3), 1.31 (t, J = 7.1 Hz, 3H, CH_3^e); ^{13}C NMR (126 MHz, $CDCl_3$, 298 K) δ: 146.6, 139.1, 133.0, 130.8, 128.6, 127.8, 126.2, 125.5, 119.4, 72.2 (CH^c), 68.2 (CH^a), 61.3 (CH_2^d), 41.2 (CH_2^b), 18.53 (Ar–CH_3), 14.6 (CH_3^e); IR ν_{max} (cm^{-1}): 2983, 2929, 2092 (C=N_2), 1693 (C=O), 1600, 1479, 1450, 1375, 1300, 1265, 1111; HRMS (ES+) $[M+H]^+$ calculated for $[C_{20}H_{22}N_3O_3]^+$: 352.1661, found 352.1659.

*Synthesis of ethyl 2-diazo-2-(2-(2-ethylphenyl)-3-phenylisoxazolidin-5-yl)acetate (**3l**)*

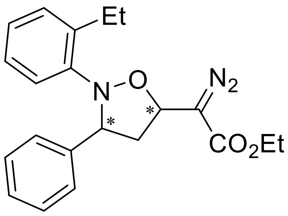

Synthesized in accordance with *General procedure d*, using $B(C_6F_5)_3$ (10 mg, 0.02 mmol), nitrone **1l** (23 mg, 0.1 mmol), vinyldiazo ester **2a** (28 mg, 0.2 mmol) in toluene to afford **3l**. All volatiles were removed *in vacuo* and the crude compound was purified *via* preparative thin layer chromatography using silica gel and hexane/ethyl acetate (90:10 v/v) as eluent. The desired product (**3l**) was obtained as a yellow oil. Yield: 27 mg, 0.07 mg, 74%.

^{1}H NMR (500 MHz, $CDCl_3$, 298 K) δ: 7.40–7.38 (m, 2H, Ar–CH), 7.31–7.28 (m, 3H, Ar–CH), 7.25–7.22 (m, 1H, Ar–CH), 7.16–7.06 (m, 3H, Ar–CH), 5.31 (t, J = 7.8 Hz, 1H, CH^c), 4.61 (t, J = 7.2 Hz, 1H, CH^a), 4.27 (qd, J = 7.1, 1.7 Hz, 2H, CH_2^d), 2.75–2.54 (m, 4H), 1.30 (t, J = 7.1 Hz, 3H, CH_3), 1.10 (t, J = 7.5 Hz, 3H, CH_3); ^{13}C NMR (126 MHz, $CDCl_3$, 298 K) δ: 145.5, 140.0, 139.0, 128.9, 128.6, 127.8, 127.6, 126.3, 126.2, 120.1, 72.0 (CH^c), 68.4 (CH^a), 61.2 (CH_2^d), 41.7 (CH_2^b), 24.0, 14.7, 14.6; IR ν_{max} (cm^{-1}): 2964, 2931, 2092 (C=N_2), 1693 (C=O), 1489, 1450, 1375, 1300, 1263, 1111, 1028; HRMS (ES+) $[M+H]^+$ calculated for $[C_{21}H_{24}N_3O_3]^+$: 366.1818, 366.1818.

*Synthesis of ethyl 2-diazo-2-(2-(4-fluorophenyl)-3-phenylisoxazolidin-5-yl)acetate (**3m**)*

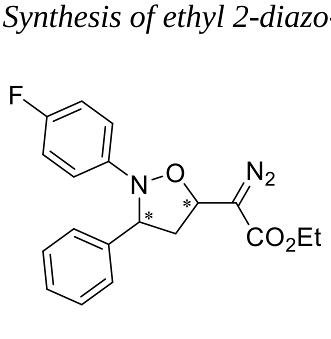

Synthesized in accordance with *General procedure d*, using $B(C_6F_5)_3$ (10 mg, 0.02 mmol), nitrone **1m** (22 mg, 0.1 mmol), vinyldiazo ester **2a** (28 mg, 0.2 mmol) in toluene to afford **3m**. All volatiles were removed *in vacuo* and the crude compound was purified *via* preparative thin layer chromatography using silica gel and hexane/ethyl acetate (90:10 v/v) as eluent. The desired product (**3m**) was obtained as a yellow oil. Yield: 29 mg, 0.08 mmol, 82%.

^{1}H NMR (400 MHz, $CDCl_3$, 298 K) δ: 7.46–7.44 (m, 2H, Ar–CH), 7.39–7.29 (m, 3H, Ar–CH), 6.94–6.86 (m, 4H, , Ar–CH), 5.35 (t, *J* = 7.4 Hz, 1H, CH^c), 4.52 (dd, *J* = 8.3, 6.3 Hz, 1H, , CH^a), 4.27 (q, *J* = 7.4, 6.7 Hz, 2H, CH_2^d), 2.74–2.61 (m, 2H, CH_2^b), 1.30 (t, *J* = 7.1 Hz, 3H, CH_3^e); ^{13}C NMR (101 MHz, $CDCl_3$, 298 K) δ: 158.9 (d, $J_{C–F}$ = 241.2 Hz), 146.4 (d, $J_{C–F}$ = 2.5 Hz), 140.5, 129.1, 128.1, 127.1, 118.1 (d, $J_{C–F}$ = 7.9 Hz), 115.4 (d, $J_{C–F}$ = 22.5 Hz), 72.7 (CH^c), 69.9 (CH^a), 61.4 (CH_2^d), 42.0 (CH_2^b), 14.6 (CH_3^e); ^{19}F NMR (376 MHz, $CDCl_3$, 298 K) δ: -121.11; IR ν_{max} (cm^{-1}): 2098 ($C=N_2$), 1693 (C=O), 1502, 1300, 1226, 1112, 1028; HRMS (ES+) $[M+H]^+$ calculated for $[C_{19}H_{19}N_3O_3F]^+$: 356.1410, found 356.1411.

*Synthesis of ethyl 2-((3R,5R)-2-(4-bromophenyl)-3-phenylisoxazolidin-5-yl)-2-diazoacetate (anti-**3n**)*

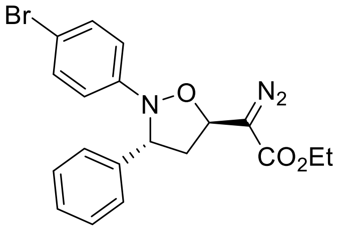

Synthesized in accordance with *General procedure d*, using $B(C_6F_5)_3$ (10 mg, 0.02 mmol), nitrone **1n** (28 mg, 0.1 mmol), vinyldiazo ester **2a** (28 mg, 0.2 mmol) in toluene to afford *anti*-**3n**. All volatiles were removed *in vacuo* and the crude compound was purified *via* preparative thin layer chromatography using silica gel and hexane/ethyl acetate (90:10 v/v) as eluent. The desired product (*anti*-**3n**) was obtained as yellow oil. Yield: 28 mg, 0.06 mmol, 67%.

^{1}H NMR (500 MHz, $CDCl_3$, 298 K) δ: 7.46–7.45 (m, 2H, Ar–CH), 7.39–7.36 (m, 2H, Ar–CH), 7.33–7.28 (m, 3H, Ar–CH), 6.82–6.79 (m, 2H, Ar–CH), 5.36 (t, *J* = 7.2 Hz, 1H, CH^c), 4.60 (dd, *J* = 8.5, 5.6 Hz, 1H, CH^a), 4.27 (q, *J* = 7.1 Hz, 2H, CH_2^d), 2.74–2.60 (m, 2H, CH_2^b), 1.30 (t, *J* = 7.1 Hz, 3H, CH_3^e); ^{13}C NMR (126 MHz, $CDCl_3$, 298 K) δ: 149.6, 140.7, 131.6, 129.1, 128.0, 126.7, 117.3, 114.8, 73.0 (CH^c), 69.1 (CH^a), 61.4 (CH_2^d), 41.8 (CH_2^b), 14.6 (CH_3^e); IR ν_{max} (cm^{-1}): 2098 ($C=N_2$), 1693 (C=O), 1485, 1267, 1244; HRMS (ES+) $[M+H]^+$ calculated for $[C_{19}H_{19}N_3O_3Br]^+$: 416.0610, found 416.0609.

*Synthesis of ethyl 2-((3R,5S)-2-(4-bromophenyl)-3-phenylisoxazolidin-5-yl)-2-diazoacetate (syn-**3n**)*

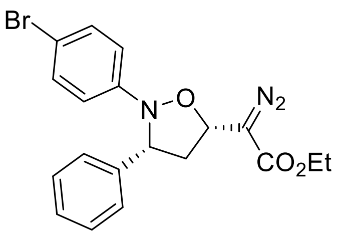

Synthesized in accordance with *General procedure d*, using $B(C_6F_5)_3$ (10 mg, 0.02 mmol), nitrone **1n** (28 mg, 0.1 mmol), vinyldiazo ester **2a** (28 mg, 0.2 mmol) in toluene to afford *syn*-**3n**. All volatiles were removed *in vacuo* and the crude compound was purified *via* preparative thin layer chromatography using silica gel

and hexane/ethyl acetate (90:10 v/v) as eluent. The desired product (*syn*-**3n**) was obtained as yellow oil. Yield: 6 mg, 0.01 mmol, 14%.

^{1}H NMR (500 MHz, $CDCl_3$, 298 K) δ: 7.44 (d, *J* = 7.7 Hz, 2H, Ar–CH), 7.39 (t, *J* = 7.5 Hz, 2H, Ar–CH), 7.37–7.30 (m, 3H, Ar–CH), 6.90 (d, *J* = 8.7 Hz, 2H, Ar–CH), 5.19 (t, *J* = 7.8 Hz, 1H, CH^c), 4.77 (t, *J* = 7.6 Hz, 1H, CH^a), 4.28 (q, *J* = 7.1 Hz, 2H, $CH_2{}^d$), 3.11–3.05 (m, 1H, CH^b), 2.29–2.23 (m, 1H, CH^b), 1.31 (t, *J* = 7.1 Hz, 3H, $CH_3{}^e$); ^{13}C NMR (126 MHz, $CDCl_3$, 298 K) δ: 150.7, 141.4, 132.0, 129.1, 127.9, 126.3, 116.6, 114.7, 72.9 (CH^c), 71.0 (CH^a), 61.4 ($CH_2{}^d$), 42.9 ($CH_2{}^b$), 14.6 ($CH_3{}^e$); IR ν_{max} (cm^{-1}): 2926, 2852, 2098 ($C=N_2$), 1737, 1693 (C=O), 1483, 1398, 1377, 1300, 1242, 1174, 1028; HRMS (ES+) $[M-N_2+H]^+$ calculated for $[C_{19}H_{19}NO_3Br]^+$: 388.0548, found 388.0541.

*Synthesis of ethyl 2-diazo-2-(2-(4-iodophenyl)-3-phenylisoxazolidin-5-yl)acetate (**3o**)*

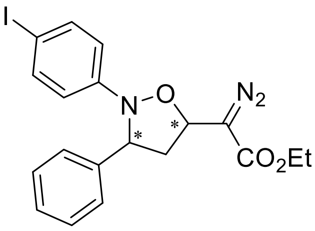

Synthesized in accordance with *General procedure d*, using $B(C_6F_5)_3$ (10 mg, 0.02 mmol), nitrone **1o** (31 mg, 0.1 mmol), vinyldiazo ester **2a** (28 mg, 0.2 mmol) in toluene to afford **3o**. All volatiles were removed *in vacuo* and the crude compound was purified *via* preparative thin layer chromatography using silica gel and hexane/ethyl acetate (90:10 v/v) as eluent. The desired product (**3o**) was obtained as a yellow oil. Yield: 42 mg, 0.09 mg, 91%.

^{1}H NMR (500 MHz, $CDCl_3$, 298 K) δ: 7.48–7.44 (m, 4H, Ar–CH), 7.38 (t, *J* = 7.5 Hz, 2H, Ar–CH), 7.33–7.29 (m, 1H, Ar–CH), 6.69 (d, *J* = 9.0 Hz, 2H, Ar–CH), 5.36 (t, *J* = 7.2 Hz, 1H, CH^c), 4.61 (dd, *J* = 8.5, 5.5 Hz, 1H, CH^a), 4.26 (q, *J* = 7.1 Hz, 2H, $CH_2{}^d$), 2.74–2.59 (m, 2H, $CH_2{}^b$), 1.29 (t, *J* = 7.1 Hz, 3H, $CH_3{}^e$); ^{13}C NMR (126 MHz, $CDCl_3$, 298 K) δ: 150.3, 140.8, 137.5, 129.1, 128.0, 126.7, 117.5, 84.8 (CH^b), 73.1 (CH^a), 68.9 (CH^b), 61.4 ($CH_2{}^d$), 41.8 ($CH_2{}^b$), 14.6 ($CH_3{}^e$); IR ν_{max} (cm^{-1}): 2981, 2096 ($C=N_2$), 1693 (C=O), 1583, 1481, 1373, 1300, 1261, 1111; HRMS (ES+) $[M+H]^+$ calculated for $[C_{19}H_{19}N_3O_3I]^+$: 464.0471, found 464.0468.

*Synthesis of ethyl 2-(3-cyclohexyl-5-methyl-2-phenylisoxazolidin-5-yl)-2-diazoacetate (**3p**)*

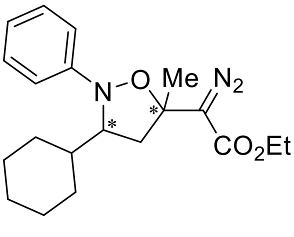

Synthesized in accordance with a slightly modified *General procedure d* (room temperature instead of 40 °C and for 3 days instead of 24 hours), using $B(C_6F_5)_3$ (10 mg, 0.02 mmol), nitrone **1i** (20 mg, 0.1 mmol), vinyldiazo ester **2d** (31 mg, 0.2 mmol) in toluene to afford **3p**. All volatiles were removed *in vacuo* and the crude compound was purified *via* preparative thin layer chromatography using silica gel and

hexane/ethyl acetate (95:5 v/v) as eluent. The desired product (**3p**) was as a yellow oil. Yield: 13 mg, 0.04 mmol, 36%.

^{1}H NMR (500 MHz, $CDCl_3$, 298 K) δ: 7.31–7.26 (m, 2H, Ar–CH), 7.14–7.08 (m, 2H, Ar–CH), 7.01 (tt, *J* = 7.3, 1.1 Hz, 1H, Ar–CH), 4.32–4.18 (m, 2H, CH_2^d), 3.50 (dt, *J* = 8.9, 6.0 Hz, 1H, CH_2^a), 2.81 (dd, *J* = 12.9, 5.9 Hz, 1H, CH_2^b), 2.26 (dd, *J* = 12.9, 8.9 Hz, 1H, CH_2^b), 1.88–1.65 (m, 5H, CH), 1.64–1.57 (m, 1H, CH), 1.56 (s, 3H, CH_3), 1.30 (t, *J* = 7.1 Hz, 3H, CH_3^e), 1.28 – 1.13 (m, 3H, CH), 1.08–0.95 (m, 2H, CH); ^{13}C NMR (101 MHz, $CDCl_3$, 298 K) δ: 165.9 (C=O), 151.2, 128.8, 123.0, 117.2 , 78.1 , 70.4 (CH^a), 60.6 (CH^d), 42.6 (CH^b), 40.7 (CH), 31.4 (CH_2), 27.6 (CH_2), 26.7 (CH_2), 26.6 (CH_2), 26.2 (CH_2), 25.8 (CH_3), 14.7 (CH_3^e); IR ν_{max} (cm^{-1}): 2980, 2926, 2853, 2099 ($C{=}N_2$), 1688 (C=O), 1599, 1489, 1451, 1370, 1310, 1258, 1180, 1068; HRMS (ES+) $[M\text{-}N_2\text{+}H]^+$ calculated for $[C_{20}H_{28}NO_3]^+$: 330.2069, found 330.2079.

2.2 Synthesis and Spectral Characterization of Mukaiyama-Mannich Addition Diazo Products

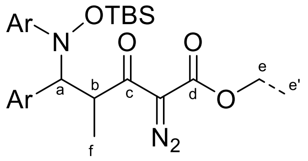

Figure S3. General labelling of Mukaiyama-Mannich addition products

The characteristic protons and carbons were assigned according to the labelling in the general structure in Figure S3. Peak assignment has been inferred from 2D-NMR analysis on compounds *anti*-**5d**.

*Synthesis of methyl 5-(((tert-butyldimethylsilyl)oxy)(phenyl)amino)-2-diazo-4-methyl-3-oxo-5-phenylpentanoate (**5a**)*

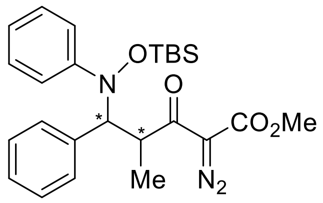

Synthesized in accordance with *General procedure e*, using $B(C_6F_5)_3$ (5 mg, 0.01 mmol), nitrone **1a** (19.7 mg, 0.1 mmol), enoldiazo ester **4b** (54.1 mg, 0.2 mmol) in toluene to afford **5a**. All volatiles were removed *in vacuo* and the crude compound was purified *via* preparative thin layer chromatography using silica gel and hexane/ethyl acetate (95:5 v/v) as eluent. The desired product (**5a**) was obtained as a white solid. Yield: 34 mg, 0.07 mmol, 73 %.

^{1}H NMR (500 MHz, $CDCl_3$, 298 K) δ: 7.25–7.15 (m, 3H, Ar–CH), 7.13–7.08 (m, 2H, Ar–CH), 7.01 (d, *J* = 7.2 Hz, 2H, Ar–CH), 6.98–6.90 (m, 3H, Ar–CH), 4.76 (d, *J* = 11.1 Hz, 1H, CH^a), 4.41–4.33 (m, 1H, CH^b), 3.89 (s, 3H, CH_3^e), 0.90 (s, 9H, OTBS), 0.85 (d, *J* = 7.1 Hz, 3H, CH_3^f),

-0.04 (br. s, 3H, OTBS), -0.36 (br. s, 3H, OTBS); ^{13}C NMR (126 MHz, $CDCl_3$, 298 K) δ: 194.8 (C=O[c]), 161.8 (C=O[d]), 153.5, 134.6, 131.3, 127.8, 127.5, 127.1, 123.9, 121.5, 76.1 (CH[a]), 52.3 (CH[e]), 43.4 (CH[b]), 26.3 (OTBS), 18.1 (OTBS), 16.8 (CH[f]), -4.5 (OTBS), -5.2 (OTBS); IR ν_{max} (cm^{-1}): 2955, 2930, 2886, 2857, 2141 ($C=N_2$), 1724 (C=O[d]), 1659 (C=O[c]), 1595, 1485, 1452, 1437, 1375, 1327, 1304, 1256, 1206, 1123, 1078, 1009; HRMS (ES+) $[M+H]^+$ calculated for $[C_{25}H_{34}N_3O_4Si]^+$: 468.2319, found 468.2318.

*Synthesis of methyl 5-(((tert-butyldimethylsilyl)oxy)(naphthalen-1-yl)amino)-2-diazo-4-methyl-5-(naphthalen-2-yl)-3-oxopentanoate (**5b**)*

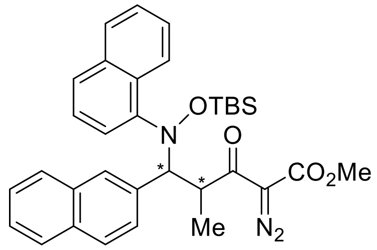

Synthesized in accordance with *General procedure e*, using $B(C_6F_5)_3$ (5 mg, 0.01 mmol), nitrone **1r** (29.7 mg, 0.1 mmol), enoldiazo ester **4b** (54.1 mg, 0.2 mmol) in toluene to afford **5b**. All volatiles were removed *in vacuo* and the crude compound was purified *via* preparative thin layer chromatography using silica gel and hexane/ethyl acetate (90:10 v/v) as eluent. The desired product (**5b**) was obtained as a yellow oil. Yield: 30 mg, 0.05 mmol, 53 %.

^{1}H NMR (500 MHz, $CDCl_3$, 298 K) δ: 8.44 (d, J = 7.8 Hz, 1H, Ar–CH), 7.85 (d, J = 7.9 Hz, 1H, Ar–CH), 7.75 (d, J = 8.1 Hz, 2H, Ar–CH), 7.58–7.53 (m, 2H, Ar–CH), 7.53–7.48 (m, 3H, Ar–CH), 7.41 (t, J = 7.3 Hz, 3H, Ar–CH), 6.93 (t, J = 7.8 Hz, 1H, Ar–CH), 6.48 (d, J = 7.6 Hz, 1H, Ar–CH), 5.09 (dq, J = 13.0, 6.6 Hz, 1H, CH[b]), 4.47 (d, J = 10.9 Hz, 1H, CH[a]), 3.91 (s, 3H, CH[e]), 1.90 (d, J = 6.7 Hz, 3H, CH[f]), 0.98 (s, 9H, OTBS), 0.48 (s, 3H, OTBS), -0.37 (s, 3H, OTBS); ^{13}C NMR (126 MHz, $CDCl_3$, 298 K) δ: 195.1 (C=O[c]), 161.5 (C=O[d]), 148.0, 134.1, 132.9, 128.4, 128.3, 127.0, 125.8, 125.6, 125.5, 124.9, 124.8, 122.8, 120.2, 76.4, 74.4, 52.4 (CH[e]), 26.4 (OTBS), 18.2 (OTBS), 17.7 (CH[f]), -3.7 (OTBS), -4.8 (OTBS); IR ν_{max} (cm^{-1}): 3051, 2953, 2928, 2855, 2137 ($C=N_2$), 1721 (C=O[d]), 1657(C=O[c]), 1593, 1574, 1506, 1435, 1379, 1362, 1306, 1256, 1202, 1128, 1005; HRMS (ES+) $[M+H]^+$ calculated for $[C_{33}H_{38}N_3O_4Si]^+$: 568.2632, found 568.2634.

*Synthesis of methyl 5-(((tert-butyldimethylsilyl)oxy)(phenyl)amino)-2-diazo-4-methyl-3-oxo-5-(4-(trifluoromethyl)phenyl)pentanoate (**5c**)*

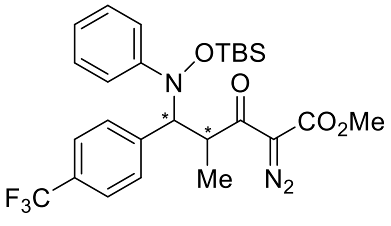

Synthesized in accordance with *General procedure e*, using $B(C_6F_5)_3$ (5 mg, 0.01 mmol), nitrone **1d** (26.5 mg, 0.1 mmol), enoldiazo ester **4b** (54.1 mg, 0.2 mmol) in toluene to afford **5c**. All volatiles were removed *in vacuo* and the crude compound was purified *via* preparative

thin layer chromatography using silica gel and hexane/ethyl acetate (95:5 v/v) as eluent. The desired product (**5c**) was obtained as a yellow oil. Yield: 36 mg, 0.07 mmol, 68 %.

^{1}H NMR (500 MHz, $CDCl_3$, 298 K) δ: 7.45 (d, *J* = 8.8 Hz, 2H, Ar–CH), 7.14–7.09 (m, 4H, Ar–CH), 7.00–6.95 (m, 1H, Ar–CH), 6.91 (d, *J* = 8.8 Hz, 2H, Ar–CH), 4.83 (d, *J* = 11.1 Hz, 1H, CH^a), 4.37 (dq, *J* = 11.1, 7.1 Hz, 1H, CH^b), 3.89 (s, 3H, CH_3^e), 0.90 (s, 9H, OTBS), 0.83 (d, *J* = 7.2 Hz, 3H, CH_3^f), -0.02 (br. s, 3H, OTBS), -0.36 (br. s, 3H, OTBS); ^{13}C NMR (126 MHz, $CDCl_3$, 298 K) δ: 194.3 ($C{=}O^c$), 161.7 ($C{=}O^d$), 153.0, 138.5, 131.5, 129.7 (q, $J_{C–F}$ = 32.2 Hz), 128.0, 125.5, 124.4 (q, $J_{C–F}$ = 272.0 Hz), 124.1 (q, $J_{C–F}$= 3.8 Hz), 121.4, 76.1, 75.6 (CH^a), 52.4 (CH^e), 43.2 (CH^b), 26.3 (OTBS), 18.1 (OTBS), 16.7 (CH^f), -4.5 (OTBS), -5.3 (OTBS). ^{19}F NMR (376 MHz, $CDCl_3$, 298 K) δ: -62.36 (Ar–CF_3); IR ν_{max} (cm^{-1}): 2957, 2930, 2893, 2859, 2141 ($C{=}N_2$), 1721 ($C{=}O^d$), 1655($C{=}O^c$), 1618, 1595, 1485, 1452, 1437, 1377, 1321, 1308, 1300, 1252, 1204, 1163, 1123, 1105, 1069, 1018, 1007; HRMS (ES+) $[M+H]^+$ calculated for $[C_{26}H_{33}N_3O_4F_3Si]^+$: 536.2192, found 536.2192.

*Synthesis of methyl (4S,5R)-5-(((tert-butyldimethylsilyl)oxy)(phenyl)amino)-2-diazo-5-(4-fluorophenyl)-4-methyl-3-oxopentanoate (anti-**5d**)*

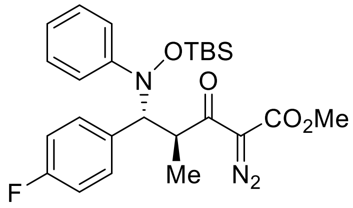

Synthesized in accordance with *General procedure e,* using $B(C_6F_5)_3$ (5 mg, 0.01 mmol), nitrone **1e** (21.5 mg, 0.1 mmol), enoldiazo ester **4b** (54.1 mg, 0.2 mmol) in toluene to afford **5d**. All volatiles were removed *in vacuo* and the crude compound was purified *via* preparative thin layer chromatography using silica gel and hexane/ethyl acetate (95:5 v/v) as eluent. The desired product (*anti*-**5d**) was obtained as a white solid. Yield: 26 mg, 0.05 mmol, 53 %.

^{1}H NMR (500 MHz, $CDCl_3$, 298 K) δ: 7.11 (dt, *J* = 8.4, 7.3 Hz, 2H, Ar–CH), 7.00–6.84 (m, 7H, Ar–CH), 4.74 (d, *J* = 11.1 Hz, 1H, CH^a), 4.32 (dq, *J* = 11.0, 7.1 Hz, 1H, CH^b), 3.89 (s, 3H, CH_3^e), 0.90 (s, 9H, OTBS), 0.84 (d, *J* = 7.2 Hz, 3H, CH_3^f), -0.03 (br. s, 3H, OTBS), -0.36 (br. s, 3H, OTBS); ^{13}C NMR (126 MHz, $CDCl_3$, 298 K) δ: 194.6 ($C{=}O^c$), 162.4 (d, $J_{C–F}$ = 245.3 Hz), 161.7 ($C{=}O^d$), 153.3, 132.7 (d, $J_{C–F}$ = 7.8 Hz), 130.2 127.8, 124.1, 121.4, 114.0 (d, $J_{C–F}$ = 21.0 Hz), 76.0, 75.3 (CH^a), 52.4 (CH^e), 43.5 (CH^b), 26.3 (OTBS), 18.1 (OTBS), 16.7 (CH^f), -4.5 (OTBS), -5.2 (OTBS); IR ν_{max} (cm^{-1}): 2955, 2930, 2893, 2857, 2141 ($C{=}N_2$), 1721 ($C{=}O^d$), 1655 ($C{=}O^c$), 1603, 1595, 1508, 1485, 1437, 1377, 1317, 1298, 1250, 1223, 1202, 1159, 1140, 1123, 1094, 1005; HRMS (ES+) $[M+H]^+$ calculated for $[C_{25}H_{33}N_3O_4FSi]^+$: 486.2224, found 486.2224.

*Synthesis of methyl (4S,5s)-5-(((tert-butyldimethylsilyl)oxy)(phenyl)amino)-2-diazo-5-(4-fluorophenyl)-4-methyl-3-oxopentanoate (syn-**5d**)*

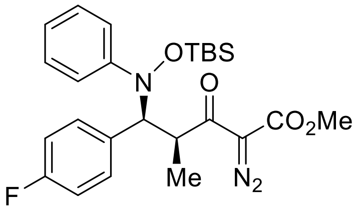

Synthesized in accordance with *General procedure e*, using $B(C_6F_5)_3$ (5 mg, 0.01 mmol), nitrone **1e** (21.5 mg, 0.1 mmol), enoldiazo ester **4b** (54.1 mg, 0.2 mmol) in toluene to afford **5d**. All volatiles were removed *in vacuo* and the crude compound was purified *via* preparative thin layer chromatography using silica gel and hexane/ethyl acetate (95:5 v/v) as eluent. The desired product (*syn*-**5d**) was obtained as a white solid. Yield: 15 mg, 0.03 mmol, 30 %.

^{1}H NMR (500 MHz, $CDCl_3$, 298 K) δ: 7.17–7.12 (m, 2H, Ar–CH), 7.01–6.96 (m, 1H, Ar–CH), 6.84–6.74 (m, 6H, Ar–CH), 4.62 (br. s, 1H, CH^a), 4.30 (br. s, 1H, CH^b), 3.87 (s, 3H, CH_3^e), 1.62 (d, J = 6.6 Hz, 3H, CH_3^f), 0.93 (s, 9H, OTBS), 0.28 (br. s, 3H, OTBS), -0.17 (br. s, 3H, OTBS); ^{13}C NMR (126 MHz, $CDCl_3$, 298 K) δ: 195.2 (C=O^c), 162.3 (d, $J_{C–F}$ = 245.1 Hz) 161.4 (C=O^d), 131.7 (d, $J_{C–F}$ = 7.9 Hz), 131.2, 128.0, 123.9, 121.0, 113.8 (d, $J_{C–F}$ = 20.9 Hz), 76.4, 60.5, 52.4 (CH^e), 43.3 (CH^b) , 26.4 (OTBS), 18.2 (OTBS), 17.7 (CH^f), -4.1 (OTBS), -4.7 (OTBS); IR ν_{max} (cm^{-1}): 2957, 2930, 2857, 2141 (C=N_2), 1719 (C=O^d), 1655 (C=O^c), 1595, 1508, 1485, 1437, 1370, 1362, 1304, 1258, 1221, 1209, 1202, 1161, 1125, 1096; HRMS (ES+) $[M+H]^+$ calculated for $[C_{25}H_{33}N_3O_4FSi]^+$: 486.2224, found 486.2227.

*Synthesis of methyl 5-(((tert-butyldimethylsilyl)oxy)(phenyl)amino)-5-(4-chlorophenyl)-2-diazo-4-methyl-3-oxopentanoate (**5e**)*

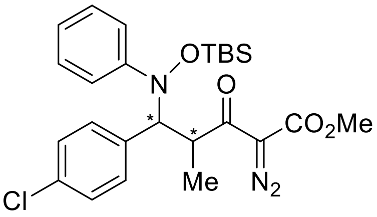

Synthesized in accordance with *General procedure e*, using $B(C_6F_5)_3$ (5 mg, 0.01 mmol), nitrone **1f** (23.1 mg, 0.1 mmol), enoldiazo ester **4b** (54.1 mg, 0.2 mmol) in toluene to afford **5e**. All volatiles were removed *in vacuo* and the crude compound was purified *via* preparative thin layer chromatography using silica gel and hexane/ethyl acetate (90:10 v/v) as eluent. The desired product (**5e**) was obtained as an off-white solid. Yield: 30 mg, 0.06 mmol, 60 %.

^{1}H NMR (500 MHz, $CDCl_3$, 298 K) δ: 7.17–7.14 (m, 2H, Ar–CH), 7.12 (dd, J = 8.5, 7.3 Hz, 2H, Ar–CH), 7.00–6.94 (m, 1H, Ar–CH), 6.93–6.90 (m, 4H, Ar–CH), 4.73 (d, J = 11.1 Hz, 1H, CH^a), 4.32 (dq, J = 11.1, 7.1 Hz, 1H, CH^b), 3.89 (s, 3H, CH^e), 0.90 (s, 9H, OTBS), 0.83 (d, J = 7.2 Hz, 3H, CH^f), -0.02 (br. s, 3H, OTBS), -0.36 (br. s, 3H, OTBS); ^{13}C NMR (126 MHz, $CDCl_3$, 298 K) δ: 194.5 (C=O^c), 161.7 (C=O^d), 153.2, 133.4, 132.9, 132.5, 127.9, 127.3, 124.1, 121.4, 76.0, 75.4 (CH^a), 52.4 (CH^e), 43.4 (CH^b), 26.3 (OTBS), 18.1 (OTBS), 16.7(CH^f), -4.5

(OTBS), -5.3 (OTBS); IR ν_{max} (cm^{-1}): 2955, 2930, 2884, 2857, 2139 ($C=N_2$), 1721 ($C=O^d$), 1655 ($C=O^c$), 1593, 1485, 1452, 1437, 1408, 1375, 1315, 1300, 1256, 1250, 1204, 1123, 1090, 1005; HRMS (ES+) $[M+H]^+$ calculated for $[C_{25}H_{33}N_3O_4SiCl]^+$: 502.1929, found 502.1931.

*Synthesis of methyl 5-(((tert-butyldimethylsilyl)oxy)(phenyl)amino)-2-diazo-5-(4-methoxyphenyl)-4-methyl-3-oxopentanoate (**5f**)*

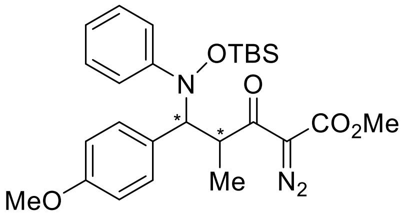

Synthesized in accordance with *General procedure e*, using $B(C_6F_5)_3$ (5 mg, 0.01 mmol), nitrone **1h** (22.7 mg, 0.1 mmol), enoldiazo ester **4b** (54.1 mg, 0.2 mmol) in toluene to afford **5f**. All volatiles were removed *in vacuo* and the crude compound was purified *via* preparative thin layer chromatography using silica gel and hexane/ethyl acetate (90:10 v/v) as eluent. The desired product (**5f**) was obtained as a yellow oil. Yield: 33 mg, 0.06 mmol, 66 %.

^{1}H NMR (500 MHz, $CDCl_3$, 298 K) δ: 7.11 (dd, J = 8.4, 7.2 Hz, 2H, Ar–CH), 6.98–6.87 (m, 5H, Ar–CH), 6.72 (d, J = 9.0 Hz, 2H, Ar–CH), 4.70 (d, J = 11.1 Hz, 1H, CH^a), 4.32 (dq, J = 11.1, 7.1 Hz, 1H, CH^b), 3.89 (s, 3H, CH^e), 3.77 (s, 3H, Ar–OCH_3), 0.91 (s, 9H, OTBS), 0.84 (d, J = 7.1 Hz, 3H, CH^f), -0.03 (br. s, 3H, OTBS), -0.36 (br. s, 3H, OTBS); ^{13}C NMR (126 MHz, $CDCl_3$, 298 K) δ: 194.9 ($C=O^c$), 161.8 ($C=O^d$), 159.0, 153.6, 132.3, 127.7, 126.7, 123.8, 121.5, 112.5, 76.0, 75.5 (CH^a), 55.2 (Ar–OCH_3), 52.3 (CH^e), 43.6 (CH^b), 26.3(OTBS), 18.1 (OTBS), 16.8 (CH^f), -4.5 (OTBS), -5.2 (OTBS); IR ν_{max} (cm^{-1}): 2930, 2857, 2139 ($C=N_2$), 1721 ($C=O^d$), 1655 ($C=O^c$), 1611, 1595, 1586, 1512, 1487, 1472, 1462, 1452, 1435, 1375, 1362, 1321, 1300, 1250, 1202, 1179, 1142, 1123, 1107, 1078, 1061, 1036, 1005; HRMS (ES+) $[M+H]^+$ calculated for $[C_{26}H_{36}N_3O_5Si]^+$: 498.2424, found 498.2423.

*Synthesis of methyl 5-(((tert-butyldimethylsilyl)oxy)(4-methoxyphenyl)amino)-2-diazo-4-methyl-3-oxo-5-phenylpentanoate (**5g**)*

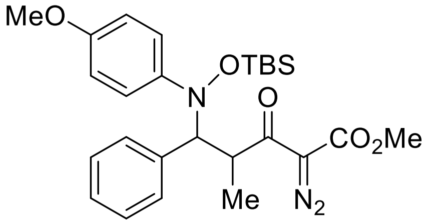

Synthesized in accordance with *General procedure e*, using $B(C_6F_5)_3$ (5 mg, 0.01 mmol), nitrone **1q** (22.7 mg, 0.1 mmol), enoldiazo ester **4b** (54.1 mg, 0.2 mmol) in toluene to afford **5g**. All volatiles were removed *in vacuo* and the crude compound was purified *via* preparative thin layer chromatography using silica gel and hexane/ethyl acetate (90:10 v/v) as eluent. The desired product (**5g**) was obtained as a yellow oil. Yield: 15 mg, 0.03 mmol, 30 %.

^{1}H NMR (500 MHz, $CDCl_3$, 298 K) δ: 7.23–7.17 (m, 3H, Ar–CH), 7.03 (d, *J* = 7.0 Hz, 2H, Ar–CH), 6.82 (d, *J* = 8.8 Hz, 2H, Ar–CH), 6.64 (d, *J* = 9.1 Hz, 2H, Ar–CH), 4.66 (d, *J* = 10.9 Hz, 1H, CH^a), 4.28 (dq, *J* = 11.0, 7.1 Hz, 1H, CH^b), 3.88 (s, 3H, CH^e), 3.74 (s, 3H, Ar–OCH_3), 0.86 (s, 9H, OTBS), 0.81 (d, *J* = 7.1 Hz, 3H, CH^f), -0.09 (br. s, 3H, OTBS), -0.34 (br. s, 3H, OTBS); ^{13}C NMR (126 MHz, $CDCl_3$, 298 K) δ: 194.9 (C=O^c), 161.8 (C=O^d), 156.5, 146.3, 135.1, 131.4, 127.5, 127.2, 123.5, 112.8, 76.1, 75.9 (CH^a), 55.4 (Ar–OCH_3), 52.3 (CH^e), 43.5 (CH^b), 26.2 (OTBS), 18.1 (OTBS), 16.8 (CH^f), -4.6 (OTBS), -5.2 (OTBS); IR ν_{max} (cm^{-1}): 2955, 2928, 2855, 2139 (C=N_2), 1724 (C=O^d), 1659 (C=O^c), 1503, 1456, 1437, 1375, 1325, 1304, 1246, 1206, 1123, 1036, 1009; HRMS (ES+) $[M+H]^+$ calculated for $[C_{26}H_{36}N_3O_5Si]^+$: 498.2424, found 498.2426.

*Synthesis of methyl 5-(((tert-butyldimethylsilyl)oxy)(p-tolyl)amino)-2-diazo-4-methyl-3-oxo-5-phenylpentanoate (**5h**)*

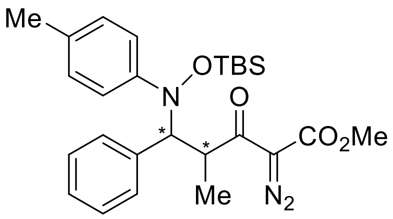

Synthesized in accordance with *General procedure e*, using $B(C_6F_5)_3$ (5 mg, 0.01 mmol), nitrone **1b** (21.1 mg, 0.1 mmol), enoldiazo ester **4b** (54.1 mg, 0.2 mmol) in toluene to afford **5h**. All volatiles were removed *in vacuo* and the crude compound was purified *via* preparative thin layer chromatography using silica gel and hexane/ethyl acetate (90:10 v/v) as eluent. The desired product (**5h**) was obtained as a yellow oil. Yield: 25 mg, 0.05 mmol, 52 %.

^{1}H NMR (500 MHz, $CDCl_3$, 298 K) δ: 7.23–7.16 (m, 3H, Ar–CH), 7.03 (d, *J* = 7.1 Hz, 2H, Ar–CH), 6.89 (d, *J* = 7.5 Hz, 2H, Ar–CH), 6.80 (d, *J* = 8.2 Hz, 2H, Ar–CH), 4.71 (d, *J* = 11.0 Hz, 1H, CH^a), 4.32 (dq, *J* = 11.0, 7.1 Hz, 1H, CH^b), 3.89 (s, 3H, CH^e), 2.25 (s, 3H, Ar-CH_3), 0.88 (s, 9H, OTBS), 0.82 (d, *J* = 7.1 Hz, 3H, CH^f), -0.08 (br. s, 3H, OTBS), -0.35 (br. s, 3H, OTBS); ^{13}C NMR (126 MHz, $CDCl_3$, 298 K) δ: 194.8 (C=O^c), 161.8 (C=O^d), 150.7, 135.1, 133.5, 131.3, 128.3, 127.4, 127.1, 121.8, 76.04 (CH^a), 75.96, 52.3 (CH^e), 43.4 (CH^b), 26.3 (OTBS), 21.0 (Ar-CH_3), 18.1 (OTBS), 16.8 (CH^f), -4.5 (OTBS), -5.2 (OTBS); IR ν_{max} (cm^{-1}): 3030, 2955, 2928, 2884, 2857, 2139 (C=N_2), 1721 (C=O^d), 1655 (C=O^c), 1505, 1472, 1452, 1435, 1375, 1323, 1302, 1256, 1250, 1204, 1123, 1007; HRMS (ES+) $[M+H]^+$ calculated for $[C_{26}H_{36}N_3O_4Si]^+$: 482.2475, found 482.2475.

*Synthesis of methyl 5-(((tert-butyldimethylsilyl)oxy)(4-fluorophenyl)amino)-2-diazo-4-methyl-3-oxo-5-phenylpentanoate (**5i**)*

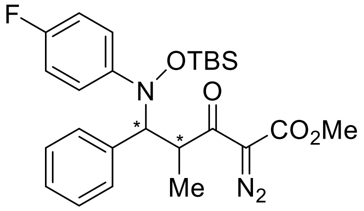

Synthesized in accordance with *General procedure e*, using $B(C_6F_5)_3$ (5 mg, 0.01 mmol), nitrone **1m** (21.5 mg, 0.1 mmol), enoldiazo ester **4b** (54.1 mg, 0.2 mmol) in toluene to afford **5i**. All volatiles were removed *in vacuo* and the crude compound was purified *via* preparative thin layer chromatography using silica gel and hexane/ethyl acetate (90:10 v/v) as eluent. The desired product (**5i**) was obtained as a yellow oil. Yield: 42 mg, 0.08 mmol, 87 %.

^{1}H NMR (500 MHz, $CDCl_3$, 298 K) δ: 7.24–7.16 (m, 3H, Ar–CH), 6.99 (d, J = 7.3 Hz, 2H, Ar–CH), 6.88–6.83 (m, 2H, Ar–CH), 6.82–6.76 (m, 2H, Ar–CH), 4.66 (d, J = 11.1 Hz, 1H, CH^a), 4.32 (dq, J = 11.0, 7.1 Hz, 1H, CH^b), 3.89 (s, 3H, CH^e), 0.88 (s, 9H, OTBS), 0.84 (d, J = 7.1 Hz, 3H, CH^f), -0.03 (br. s, 3H, OTBS), -0.34 (br. s, 3H, OTBS); ^{13}C NMR (126 MHz, $CDCl_3$, 298 K) δ: 194.8 (C=O^c), 161.7 (C=O^c), 159.7 (d, $J_{C–F}$ = 241.9 Hz), 149.4, 134.2, 131.4, 127.7, 127.2, 123.2, 114.4 (d, $J_{C–F}$ = 22.3 Hz), 76.2 (CH^a), 76.0, 52.4 (CH^e), 43.5 (CH^b), 26.2 (OTBS), 18.1 (OTBS), 16.7 (CH^f), -4.5 (OTBS), -5.3 (OTBS); IR ν_{max} (cm^{-1}): 2955, 2930, 2886, 2857, 2139 (C=N_2), 1721 (C=O^d), 1655 (C=O^c), 1601, 1499, 1472, 1454, 1437, 1376, 1325, 1304, 1256, 1250, 1225, 1202, 1148, 1123, 1094, 1007, 1001; HRMS (ES+) $[M+H]^+$ calculated for $[C_{25}H_{33}N_3O_4FSi]^+$: 486.2224, found 486.2224.

*Synthesis of methyl 5-((4-bromophenyl)((tert-butyldimethylsilyl)oxy)amino)-2-diazo-4-methyl-3-oxo-5-phenylpentanoate (**5j**)*

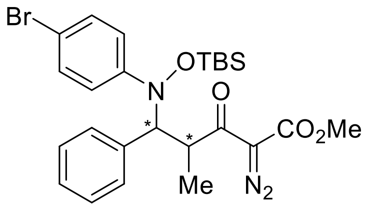

Synthesized in accordance with *General procedure e*, using $B(C_6F_5)_3$ (5 mg, 0.01 mmol), nitrone **1n** (27.6 mg, 0.1 mmol), enoldiazo ester **4b** (54.1 mg, 0.2 mmol) in toluene to afford **5j**. All volatiles were removed *in vacuo* and the crude compound was purified *via* preparative thin layer chromatography using silica gel and hexane/ethyl acetate (90:10 v/v) as eluent. The desired product (**5j**) was obtained as a yellow oil. Yield: 34 mg, 0.06 mmol, 63 %.

^{1}H NMR (500 MHz, $CDCl_3$, 298 K) δ: 7.24–7.17 (m, 5H, Ar–CH), 6.99 (d, J = 6.7 Hz, 2H, Ar–CH), 6.81 (d, J = 8.5 Hz, 2H, Ar–CH), 4.70 (d, J = 11.0 Hz, 1H, CH^a), 4.34 (dq, J = 11.1, 7.1 Hz, 1H, CH^b), 3.89 (s, 3H, CH^e), 0.90 (s, 9H, OTBS), 0.85 (d, J = 7.1 Hz, 3H, CH^f), -0.02 (br. s, 3H, OTBS), -0.35 (br. s, 3H, OTBS); ^{13}C NMR (126 MHz, $CDCl_3$, 298 K) δ: 194.8

(C=O[c]), 161.7 (C=O[d]), 152.8, 133.8, 131.3, 130.8, 127.7, 127.3, 123.2, 116.8, 76.1 (CH[a]), 52.4 (CH[e]), 43.4 (CH[b]), 26.2 (OTBS), 18.1 (OTBS), 16.7 (CH[f]), -4.4 (OTBS), -5.2 (OTBS);
IR ν_{max} (cm^{-1}): 2955, 2928, 2857, 2141(C=N_2), 1719 (C=O[d]), 1655 (C=O[c]), 1479, 1435, 1327, 1302, 1256, 1250, 1204, 1123, 1007; HRMS (ES+) $[M+H]^+$ calculated for $[C_{25}H_{33}N_3O_4SiBr]^+$: 546.1424, found 546.1425.

*Synthesis methyl 5-(((tert-butyldimethylsilyl)oxy)(4-iodophenyl)amino)-2-diazo-4-methyl-3-oxo-5-phenylpentanoate (**5k**)*

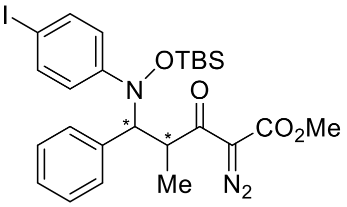

Synthesized in accordance with *General procedure e*, using $B(C_6F_5)_3$ (5 mg, 0.01 mmol), nitrone **1o** (32.3 mg, 0.1 mmol), enoldiazo ester **4b** (54.1 mg, 0.2 mmol) in toluene to afford **5k**. All volatiles were removed *in vacuo* and the crude compound was purified *via* preparative thin layer chromatography using silica gel and hexane/ethyl acetate (90:10 v/v) as eluent. The desired product (**5k**) was obtained as a yellow oil. Yield: 35 mg, 0.06 mmol, 60 %.

^{1}H NMR (500 MHz, $CDCl_3$, 298 K) δ: 7.40 (d, *J* = 8.8 Hz, 2H, Ar–CH), 7.24–7.17 (m, 3H, Ar–CH), 6.99 (d, *J* = 7.0 Hz, 2H, Ar–CH), 6.69 (d, *J* = 8.4 Hz, 2H, Ar–CH), 4.71 (d, *J* = 11.1 Hz, 1H, CH[a]), 4.34 (dq, *J* = 11.1, 7.1 Hz, 1H, CH[b]), 3.89 (s, 3H, CH[e]), 0.89 (s, 9H, OTBS), 0.84 (d, *J* = 7.1 Hz, 3H, CH[f]), -0.03 (s, 3H, OTBS), -0.35 (s, 3H, OTBS); ^{13}C NMR (126 MHz, $CDCl_3$, 298 K) δ: 194.7 (C=O[c]), 161.7 (C=O[d]), 153.6, 136.8, 133.9, 131.3, 127.7, 127.3, 123.5, 87.7, 76.1 (CH[a]), 52.4 (CH[e]), 43.3 (CH[b]), 26.2 (OTBS), 18.1 (OTBS), 16.7 (CH[f]), -4.4 (OTBS), -5.2 (OTBS); IR ν_{max} (cm^{-1}): 2953, 2928, 2884, 2857, 2139 (C=N_2), 1719 (C=O[d]), 1655 (C=O[c]), 1582, 1478, 1460, 1454, 1435, 1391, 1375, 1362, 1327, 1300, 1256, 1250, 1204, 1142, 1123, 1061; HRMS (ES+) $[M+H]^+$ calculated for $[C_{25}H_{33}N_3O_4Si^{127}I]^+$: 594.1285, found 594.1288.

*Synthesis ethyl 5-(((tert-butyldimethylsilyl)oxy)(phenyl)amino)-2-diazo-5-(4-fluorophenyl)-3-oxopentanoate (**5l**)*

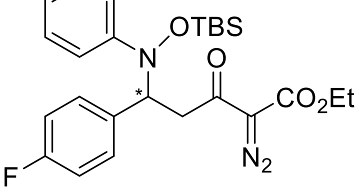

Synthesized in accordance with *General procedure d*, using $B(C_6F_5)_3$ (10 mg, 0.02 mmol), nitrone **1e** (21.5 mg, 0.1 mmol), enoldiazo ester **4b** (54.1 mg, 0.2 mmol) in toluene to afford **5l**. All volatiles were removed *in vacuo* and the crude compound was purified *via* preparative thin layer chromatography using silica gel and hexane/ethyl acetate (90:10 v/v) as eluent. The desired product (**5l**) was obtained as a yellow oil. Yield: 42 mg, 0.09 mmol, 90 %.

^{1}H NMR (500 MHz, $CDCl_3$, 298 K) δ: 7.21–7.15 (m, 2H, Ar–CH), 7.16–7.11 (m, 2H, Ar–CH), 7.04 (d, J = 7.9 Hz, 2H, Ar–CH), 6.99 (t, J = 7.3 Hz, 1H, Ar–CH), 6.90 (t, J = 8.7 Hz, 2H, Ar–CH), 4.91 (dd, J = 8.5, 5.5 Hz, 1H, CH^a), 4.32 (qd, J = 7.1, 1.2 Hz, 2H, CH^e), 3.72 (dd, J = 17.5, 8.6 Hz, 1H, CH^b), 3.53–3.44 (m, 1H, CH^b), 1.34 (t, J = 7.1 Hz, 3H, $CH^{e'}$), 0.93 (s, 9H, OTBS), -0.05 (br. s, 3H, OTBS), -0.38 (br. s, 3H, OTBS); ^{13}C NMR (126 MHz, $CDCl_3$, 298 K) δ: 190.4 (C=O^c), 162.4 (d, $J_{C–F}$ = 245.6 Hz), 161.3 (C=O^d), 152.5, 131.4 (d, $J_{C–F}$ = 7.9 Hz), 128.2, 124.1, 121.1, 114.5 (d, $J_{C–F}$ = 21.1 Hz), 76.7, 69.2 (CH^a), 61.6(CH^e), 40.6 (CH^b), 26.3 (OTBS), 18.1 (OTBS), 14.5 ($CH^{e'}$), -4.8 (OTBS), -5.3 (OTBS); IR ν_{max} (cm^{-1}): 2980, 2957, 2930, 2889, 2857, 2133 (C=N_2), 1715 (C=O^d), 1655 (C=O^c), 1651, 1605, 1595, 1508, 1487, 1472, 1391, 1373, 13112, 1300, 1256, 1219, 1206, 1173, 1159, 1126, 1074, 1061, 1015; HRMS (ES+) $[M+H]^+$ calculated for $[C_{25}H_{33}N_3O_4SiF]^+$: 486.2224, found 486.2222.

*Synthesis of methyl 5-(((tert-butyldimethylsilyl)oxy)(phenyl)amino)-2-diazo-5-(4-fluorophenyl)-3-oxo-4-phenylpentanoate (**5m**)*

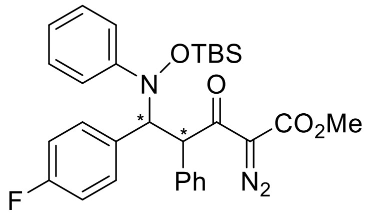

Synthesized in accordance with *General procedure d,* using $B(C_6F_5)_3$ (5 mg, 0.01 mmol), nitrone **1e** (21.5 mg, 0.1 mmol), enoldiazo ester **4c** (66.4 mg, 0.2 mmol) in toluene to afford **5m**. All volatiles were removed *in vacuo* and the crude compound was purified *via* preparative thin layer chromatography using silica gel and hexane/ethyl acetate (90:10 v/v) as eluent. The desired product (**5m**) was obtained as a yellow oil. Yield: 16 mg, 0.03 mmol, 30 %.

^{1}H NMR (500 MHz, $CDCl_3$, 298 K) δ: 7.21 (d, J = 8.1 Hz, 2H, Ar–CH), 7.14 (dd, J = 8.5, 7.3 Hz, 2H, Ar–CH), 7.07–7.02 (m, 2H, Ar–CH), 7.01–6.95 (m, 4H, Ar–CH), 6.87–6.81 (m, 2H, Ar–CH), 6.65 (t, J = 8.9 Hz, 2H, Ar–CH), 5.64 (d, J = 11.7 Hz, 1H, CH^b), 5.26 (d, J = 11.4 Hz, 1H, CH^a), 3.84 (s, 3H, CH^e), 0.96 (s, 9H, OTBS), 0.03 (s, 3H, OTBS), -0.34 (s, 3H, OTBS); ^{13}C NMR (126 MHz, $CDCl_3$, 298 K) δ: 190.7 (C=O^c), 161.9 (d, J_{C-F} = 245.0 Hz), 161.5 (C=O^d), 135.6, 132.9 (d, J_{C-F} = 8.0 Hz), 130.1, 128.3, 127.9, 127.2, 124.3, 121.7, 113.5 (d, $J_{C–F}$ = 21.0 Hz), 75.8 (CH^a), 54.0 (CH^b), 52.3 (CH^e), 26.3, 18.2 (OTBS), -4.5 (OTBS), -5.2 (OTBS); IR ν_{max} (cm^{-1}): 3063, 3034, 2955, 2928, 2857, 2137 (C=N_2), 1721 (C=O^d), 1655 (C=O^c), 1605, 1508, 1485, 1437, 1360, 1316, 1298, 1258, 1225, 1202, 1159, 1132, 1084, 1007; HRMS (ES+) $[M+H]^+$ calculated for $[C_{30}H_{35}N_3O_4FSi]^+$: 548.2381, found 548.2382.

2.3 Further functionalization and Spectral Characterization of Isoxazolidine and Mukaiyama-Mannich Addition Diazo Products

*Synthesis of ethyl 2-(4-fluorophenyl)-4-hydroxy-2,3-dihydro-1H-benzo[b]azepine-5-carboxylate (**6a**)*

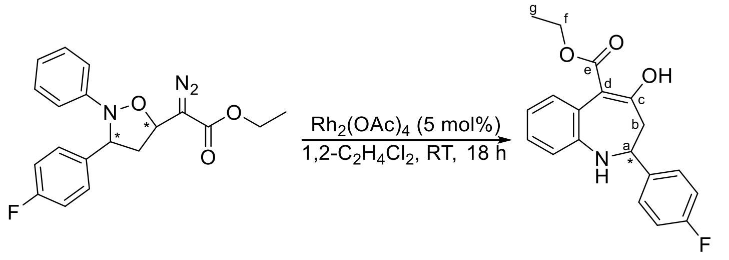

Following a reported method,[16] a microwave vial was charged with $Rh_2(OAc)_4$ (4.6 mg, 0.1 mmol, 0.05 equiv.), closed with a PTFE crimper cap and evacuated/backfilled 3 times with nitrogen. Then, dry 1,2-$C_2CH_4Cl_2$ (1 mL) was added. Separately, ethyl 2-diazo-2-(3-(4-fluorophenyl)-2-phenylisoxazolidin-5-yl)acetate (**3e**) (74 mg, 2.1 mmol, 1 equiv.) was dissolved in dry 1,2-$C_2CH_4Cl_2$ (1 mL). This solution was then added dropwise to the $Rh_2(OAc)_4$ suspension. The reaction mixture was then left to stir overnight at room temperature. After 18 h, the reaction was stopped, and the reaction mixture was passed through a short pad of Celite. The solvent was removed and the crude oil was purified by flash column chromatography (9:1 hexane:EtOAc) to afford the desired product (**6a**) as a yellow oil. Yield: 39 mg, 1.2 mmol, 57%.

^{1}H NMR (500 MHz, $CDCl_3$, 298 K) δ: 13.13 (s, 1H, OH), 7.40 (dd, J = 8.0, 1.8 Hz, 1H, Ar–CH), 7.38–7.34 (m, 2H, Ar–CH), 7.18–7.12 (m, 1H, Ar–CH), 7.06–7.01 (m, 3H, Ar–CH), 6.82 (dd, J = 8.0, 1.2 Hz, 1H, Ar–CH), 5.07 (dd, J = 7.6, 5.8 Hz, 1H, CH^a), 4.35–4.23 (m, 2H, CH_2^f), 2.61–2.51 (m, 2H, CH_2^b), 1.31 (t, J = 7.1 Hz, 3H, CH_3^g); ^{13}C NMR (126 MHz, $CDCl_3$, 298 K) δ: 174.8 (C^c), 171.7 (C=O), 163.5, 161.5, 143.8, 140.7, 132.1, 127.9 (d, $J_{C\text{-}F}$ = 8.1 Hz), 127.6, 126.2, 122.0, 121.5, 115.7 (d, $J_{C\text{-}F}$ = 21.6 Hz), 101.1 (C^d), 68.6 (CH^a), 61.1 (CH^f), 39.5 (CH^b), 14.3 (CH^g); IR ν_{max} (cm^{-1}): 3352 (OH), 3055, 2980, 2928, 1713, 1638 (C=O), 1602 (C=C), 1506, 1474, 1398, 1379, 1341, 1329, 1294, 1281, 1260, 1219, 1157, 1096, 1059, 1015; HRMS (ES+) $[M+H]^+$ calculated for $[C_{19}H_{19}NO_3F]^+$: 328.1349, found 328.1349.

*Synthesis of methyl 2-((tert-butyldimethylsilyl)oxy)-5-(4-chlorophenyl)-4-methyl-3-oxo-1-phenylpyrrolidine-2-carboxylate (**6b**).*

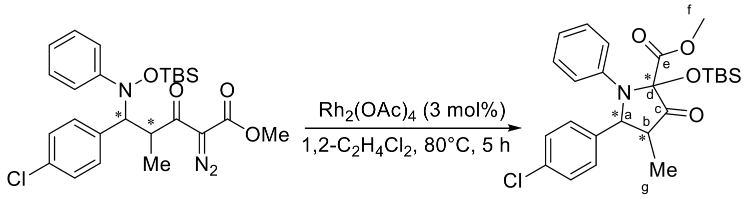

Following a reported method,[17] a microwave vial was charged with $Rh_2(OAc)_4$ (2 mg, 0.05 mmol, 0.03 equiv.), closed with a PTFE crimper cap and evacuated/backfilled 3 times with nitrogen. Then, dry 1,2-$C_2CH_4Cl_2$ (1 mL) was added. Separately, methyl 5-(((tert-butyldimethylsilyl)oxy)(phenyl)amino)-5-(4-chlorophenyl)-2-diazo-4-methyl-3-oxopentanoate (**5e**) (75 mg, 0.15 mmol, 1 equiv.) was dissolved in dry 1,2-$C_2CH_4Cl_2$ (1 mL). This solution was then added dropwise to the $Rh_2(OAc)_4$ suspension dropwise. The reaction mixture was then left to stir for 5 hours at 80 °C. After complete consumption of the starting material, the reaction was stopped, and the reaction mixture was passed through a short pad of Celite. The solvent was removed and the crude oil was purified by flash column chromatography (9:1 hexane:EtOAc) to afford the desired product (**6b**) as a yellow oil. Yield: 44 mg, 0.09 mmol, 62%.

^{1}H NMR (500 MHz, $CDCl_3$, 298 K) δ: 7.39–7.35 (m, 2H, Ar–CH), 7.32–7.28 (m, 2H, Ar–CH), 7.13–7.06 (m, 2H, Ar–CH), 6.90 (dd, *J*=8.9, 1.1, 2H, Ar–CH), 6.85–6.80 (m, 1H, Ar–CH), 4.62 (d, *J*=7.5, 1H, CH^a), 3.50 (s, 3H, CH^f), 2.63–2.56 (m, 1H, CH^b), 1.33 (d, *J*=7.0, 3H, CH^g), 0.99 (s, 9H, OTBS), 0.37 (s, 3H, OTBS), 0.16 (s, 3H, OTBS); ^{13}C NMR (126 MHz, $CDCl_3$, 298 K) δ: 207.0 (C^c), 169.4(C^e), 143.2, 140.4, 133.5, 129.5, 128.8, 127.5, 121.3, 117.5, 92.0, 65.8 (CH^a), 53.0 (CH^f), 49.8 (CH^b), 25.9 (OTBS), 18.8 (OTBS), 11.8 (CH^g), -3.1 (OTBS), -3.4 (OTBS); IR ν_{max} (cm^{-1}): 2953, 2930, 2884, 2857, 1771 (C=O), 1755 (C=O), 1599, 1503, 1491, 1456, 1339, 1250, 1171, 1134, 1090, 1074, 1015; HRMS (ES+) $[M+H]^+$ calculated for $[C_{25}H_{33}NO_4SiCl]^+$: 474.1867, found 474.1864.

3.0 NMR Spectra

Figure S4: ^{1}H NMR (500 MHz, $CDCl_3$, 298 K) spectrum of **1l**.

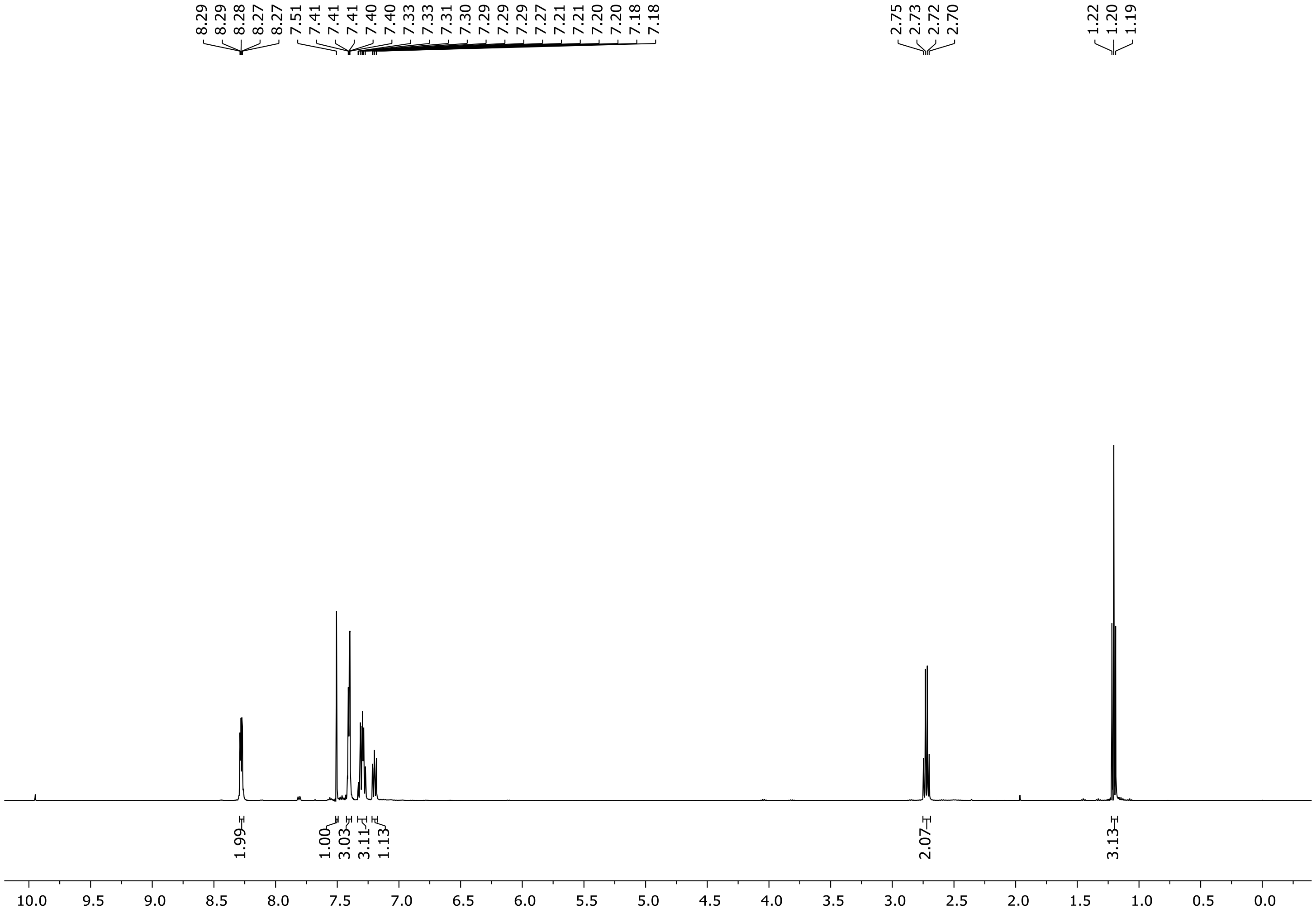

Figure S5: ^{13}C NMR (126 MHz, $CDCl_3$, 298 K) spectrum of **1l**.

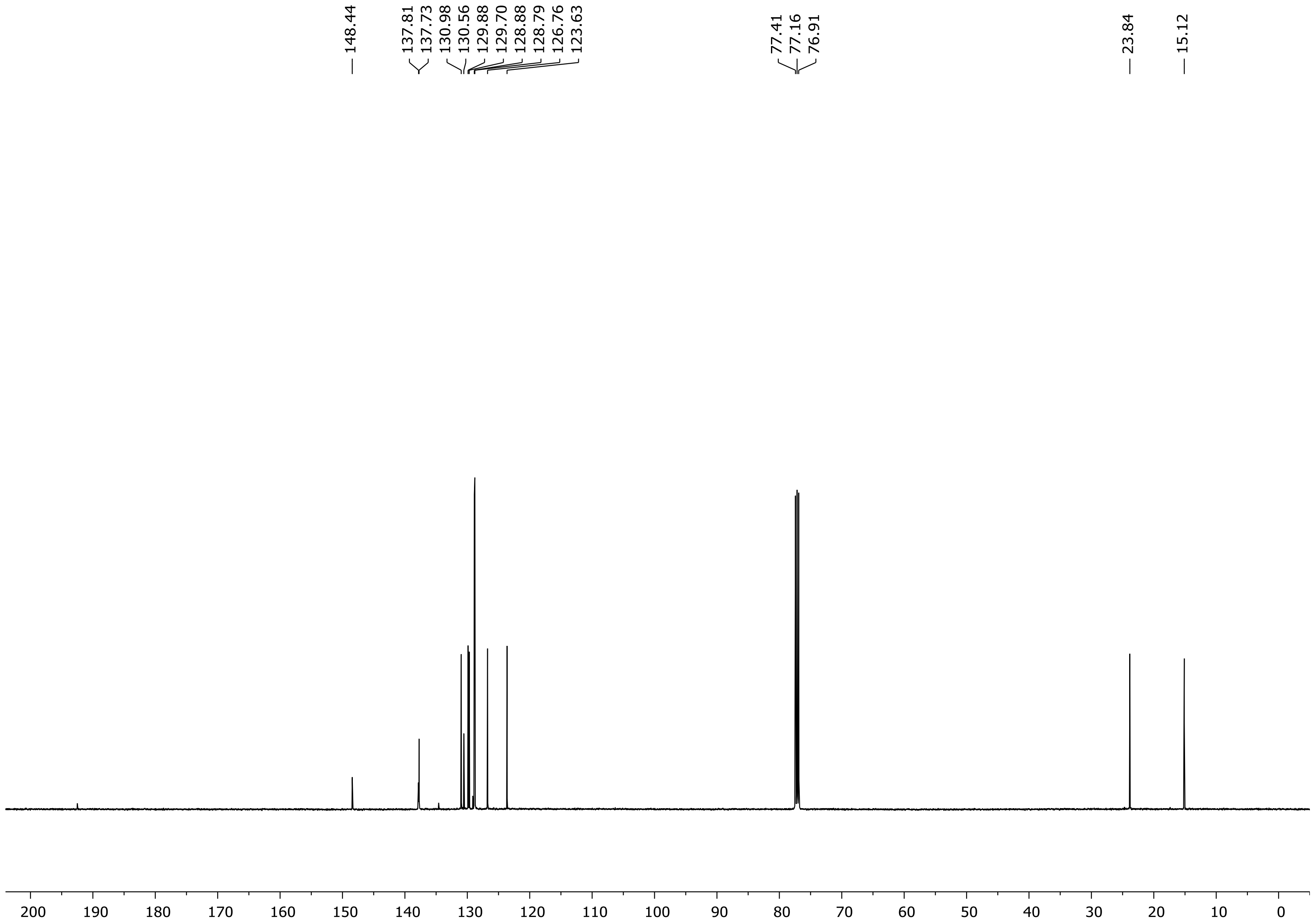

Figure S6: ^{1}H NMR (400 MHz, $CDCl_3$, 298 K) spectrum of **1o**.

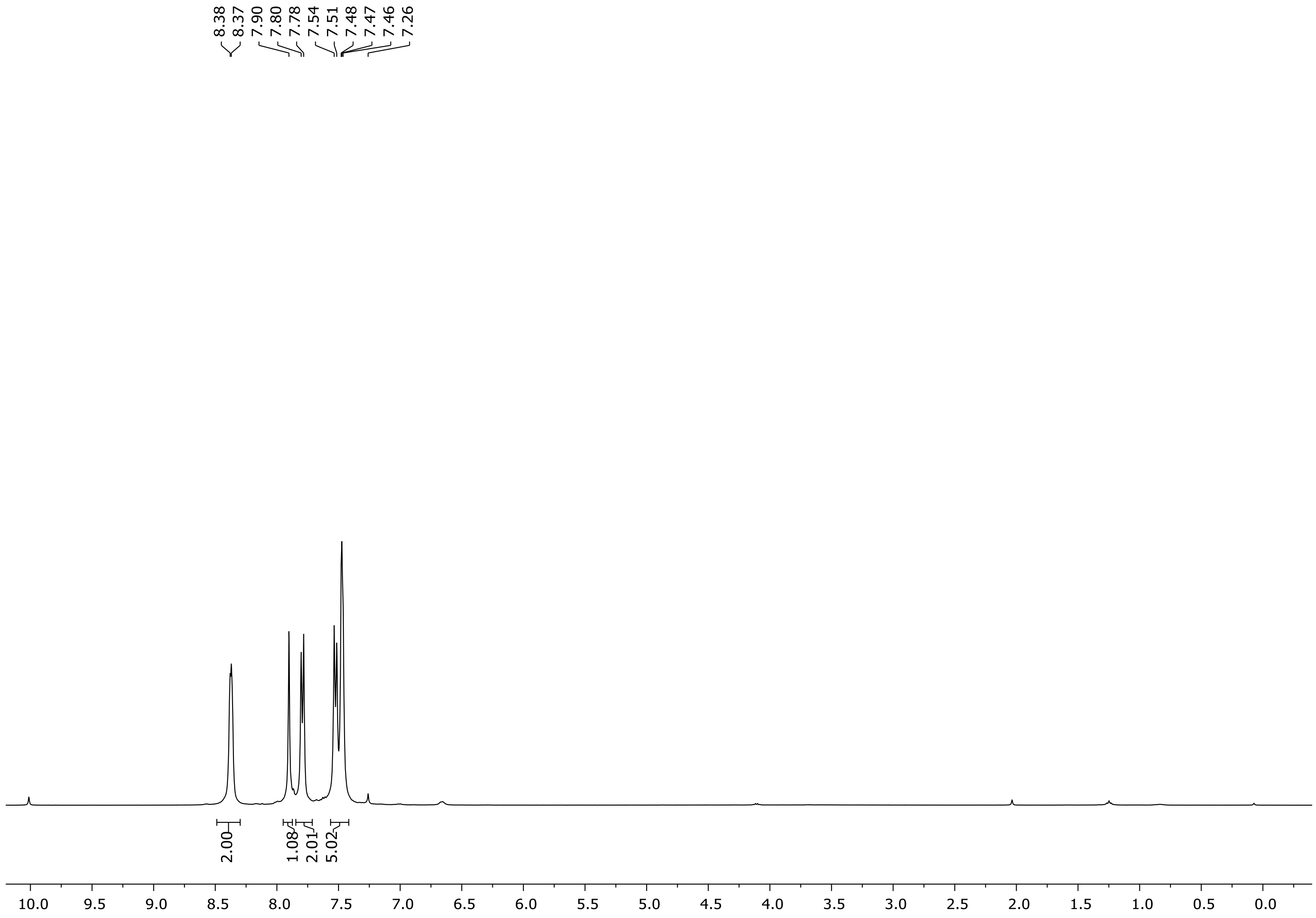

Figure S7: ^{13}C NMR (101 MHz, $CDCl_3$, 298 K) spectrum of **1o**.

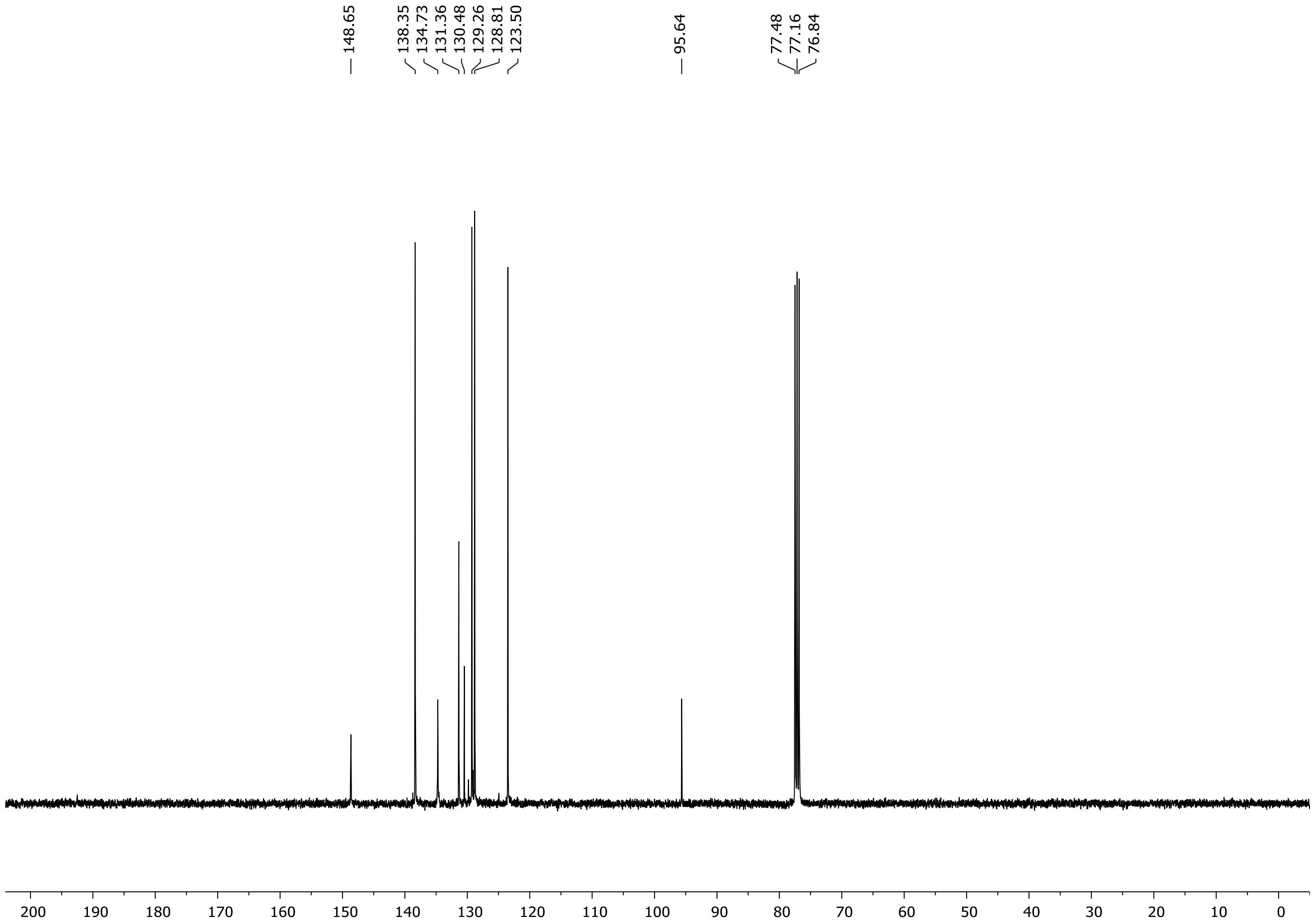

Figure S8: ^{1}H NMR (500 MHz, $CDCl_3$, 298 K) spectrum of **1r**.

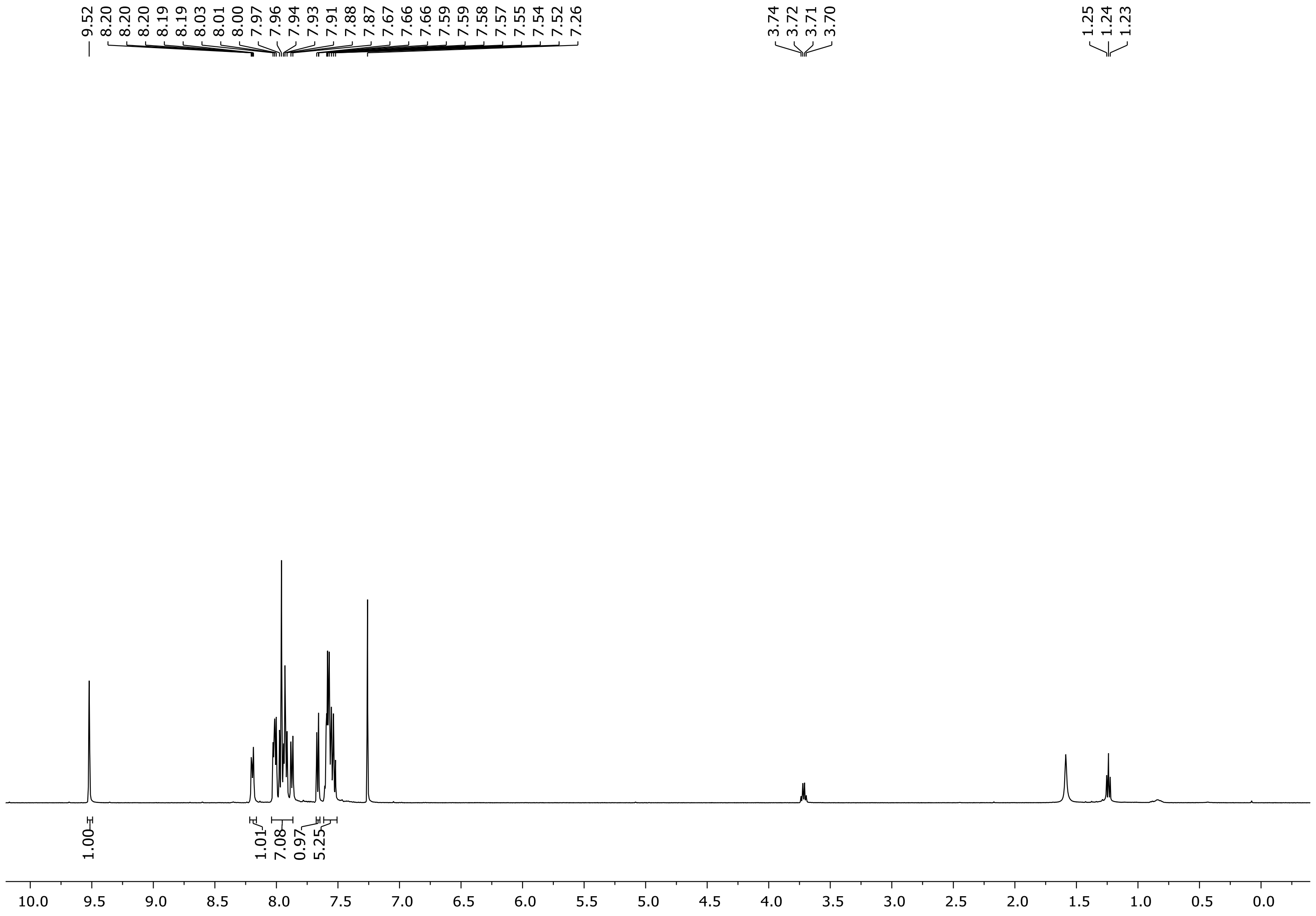

Figure S9: ^{13}C NMR (126 MHz, $CDCl_3$, 298 K) spectrum of **1r**.

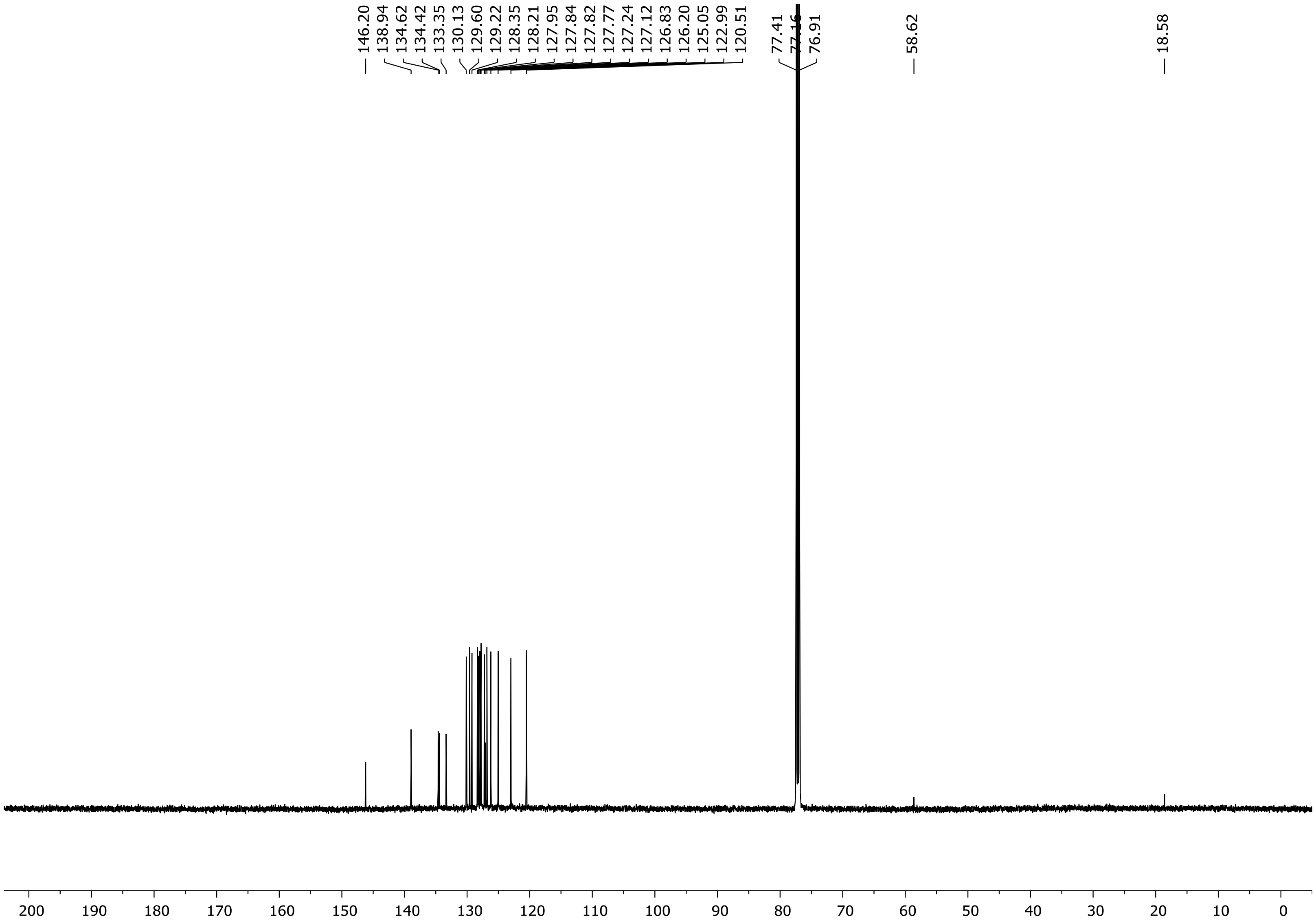

Figure S10: ^{1}H NMR (500 MHz, $CDCl_3$, 298 K) spectrum of **3a**.

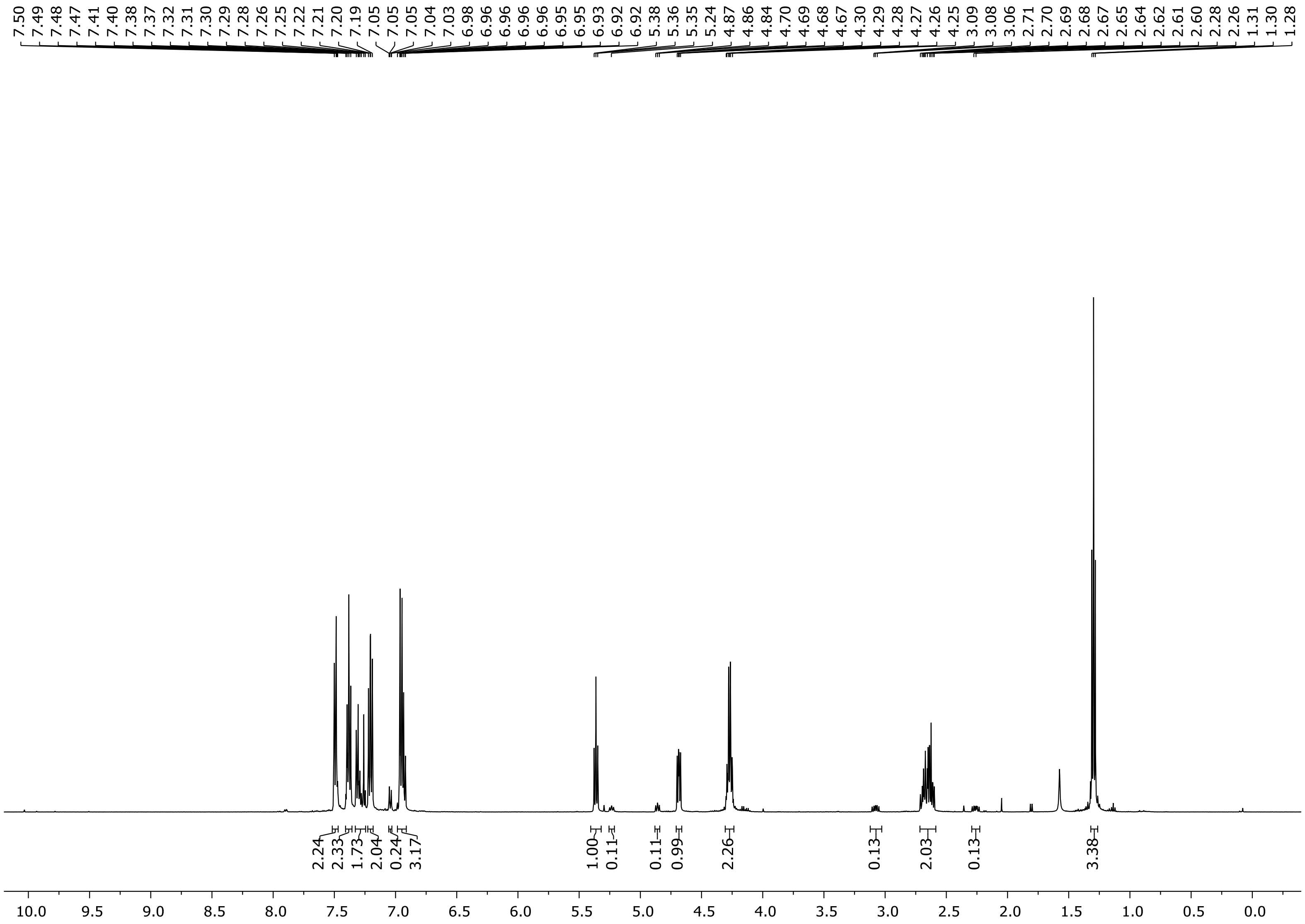

Figure S11: ^{13}C NMR (126 MHz, $CDCl_3$, 298 K) spectrum of **3a**.

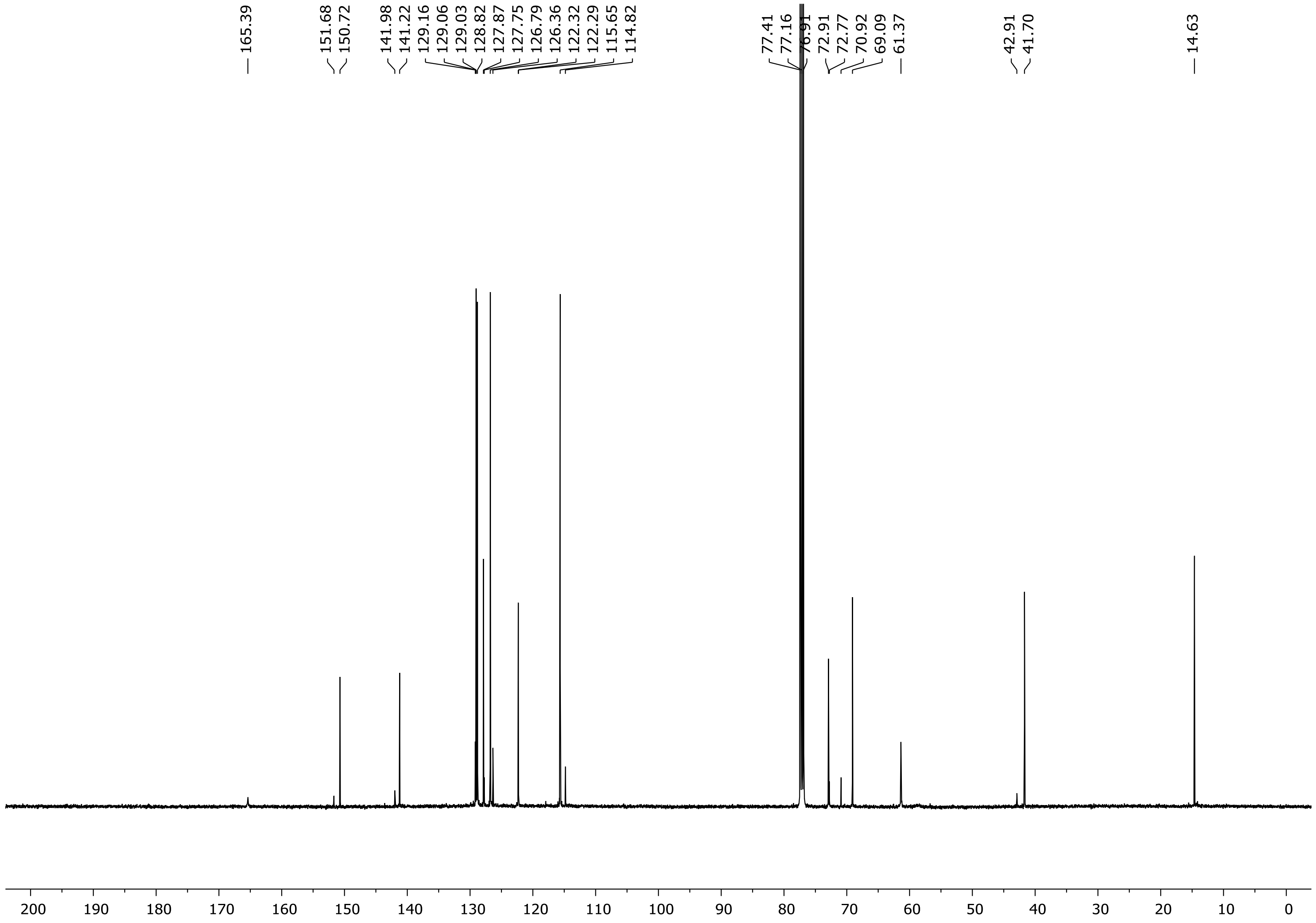

Figure S12: ^{1}H NMR (500 MHz, $CDCl_3$, 298 K) spectrum of **3b**.

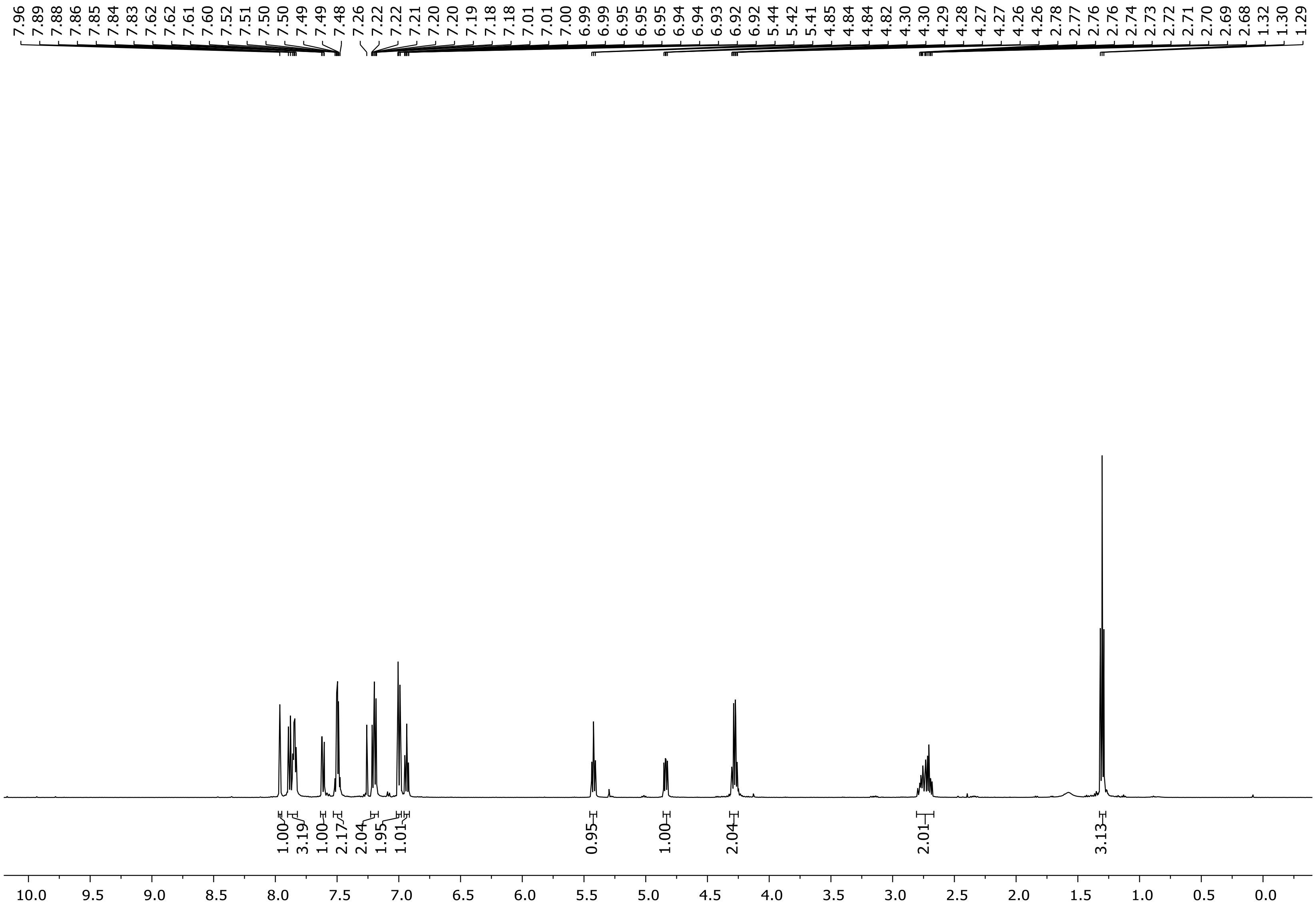

Figure S13: ^{13}C NMR (126 MHz, $CDCl_3$, 298 K) spectrum of **3b**.

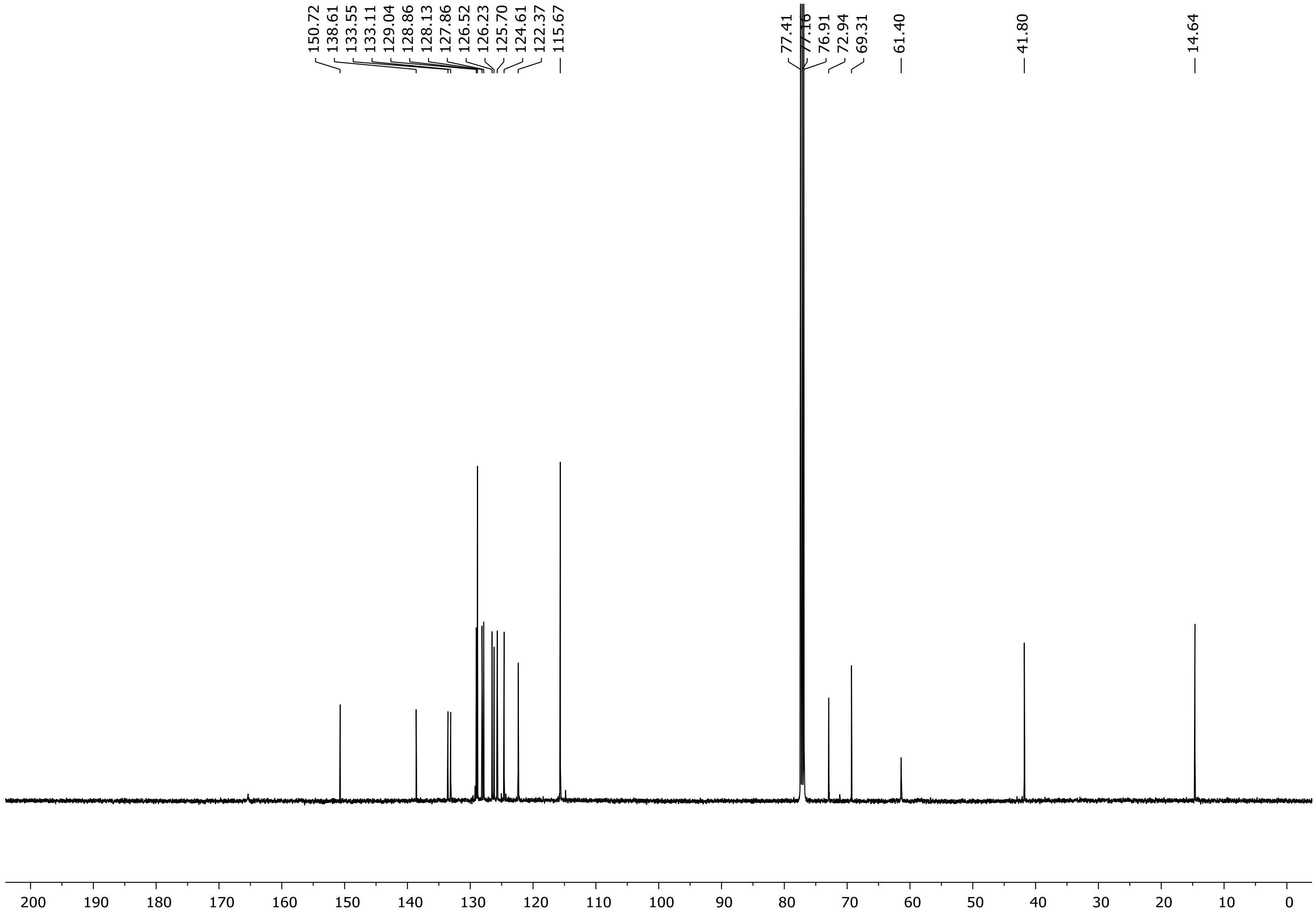

Figure S14: ^{1}H NMR (500 MHz, $CDCl_3$, 298 K) spectrum of **3c**.

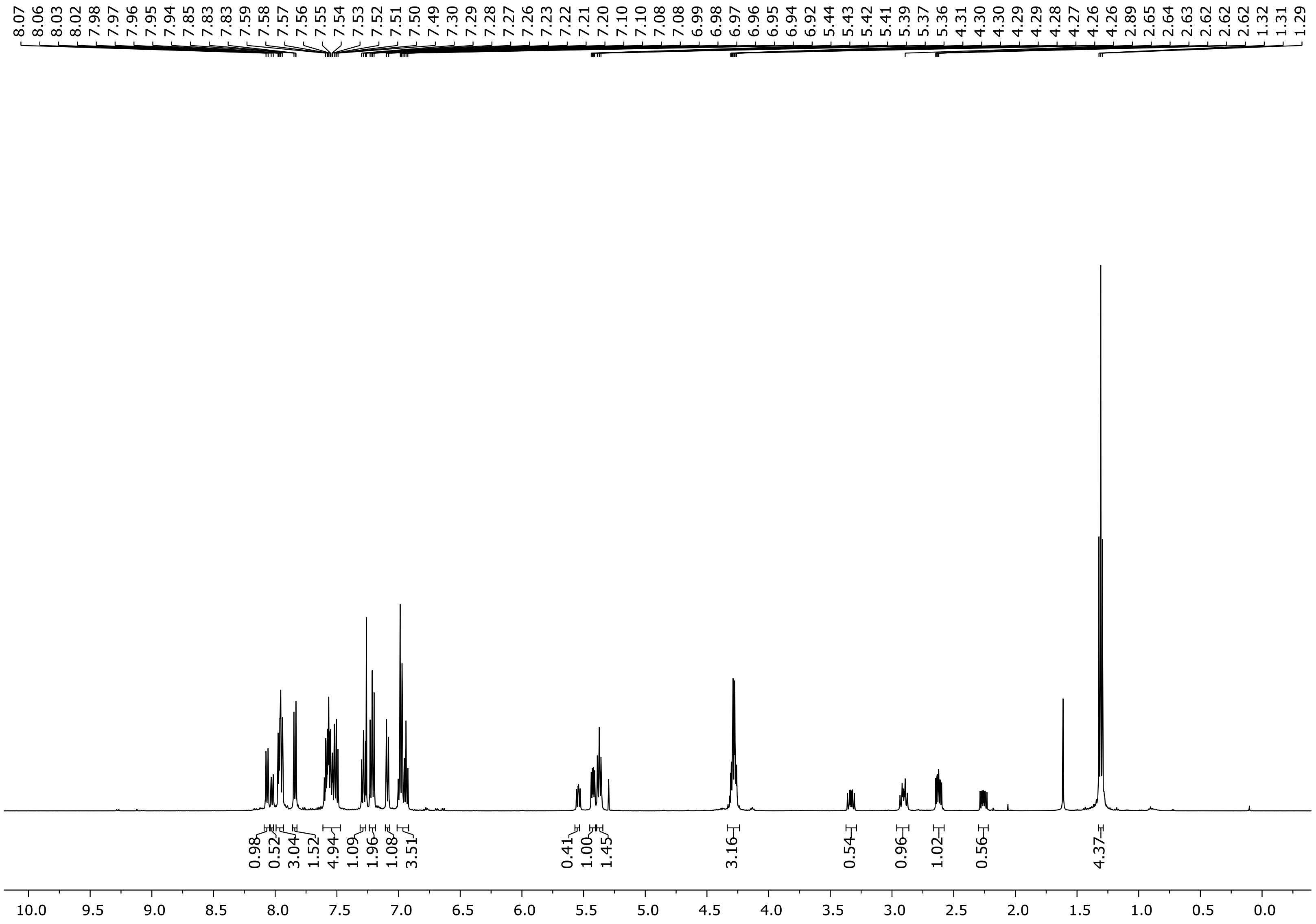

Figure S15: ^{13}C NMR (126 MHz, $CDCl_3$, 298 K) spectrum of **3c**.

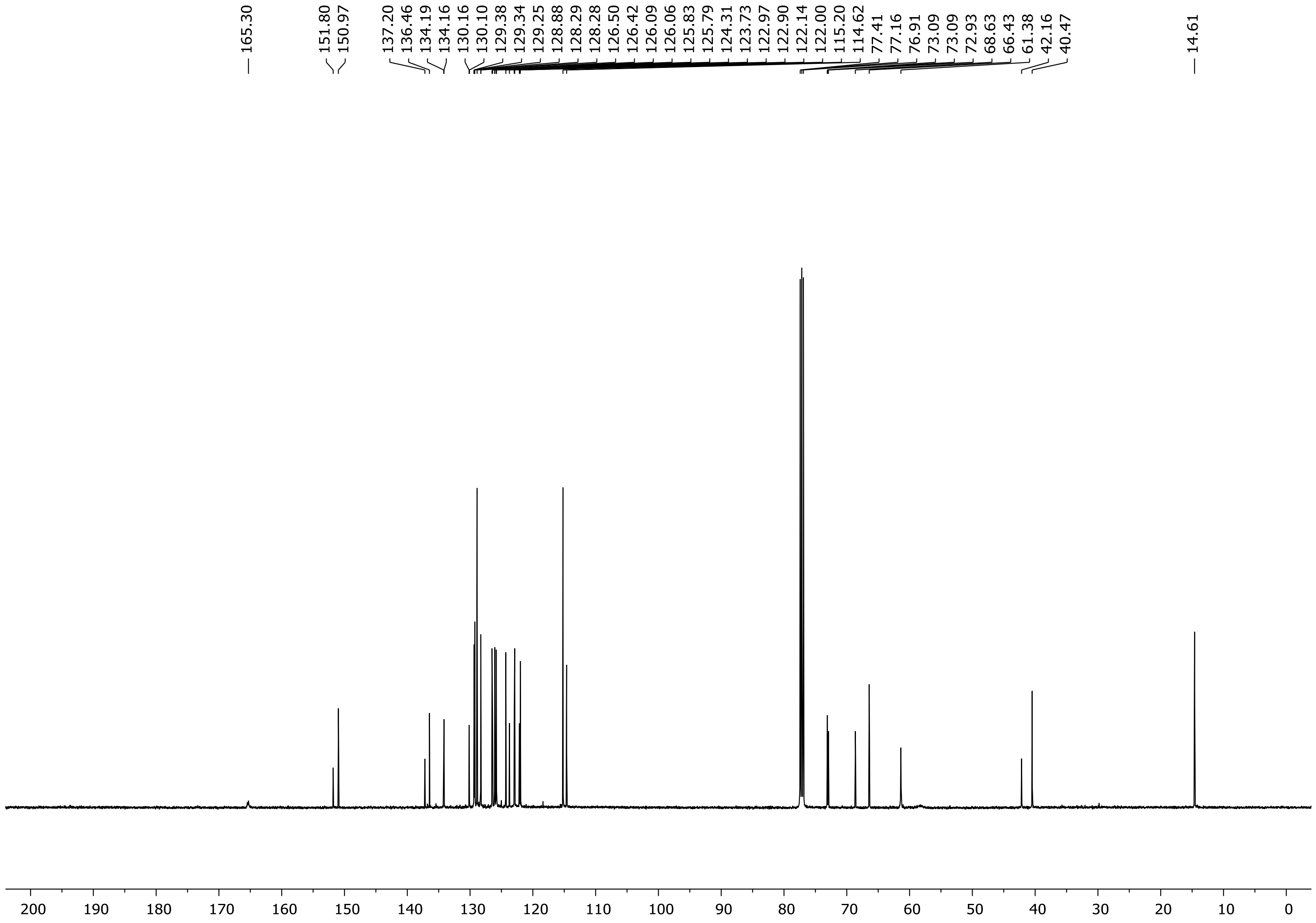

Figure S16: ^{1}H NMR (500 MHz, $CDCl_3$, 298 K) spectrum of **3d**.

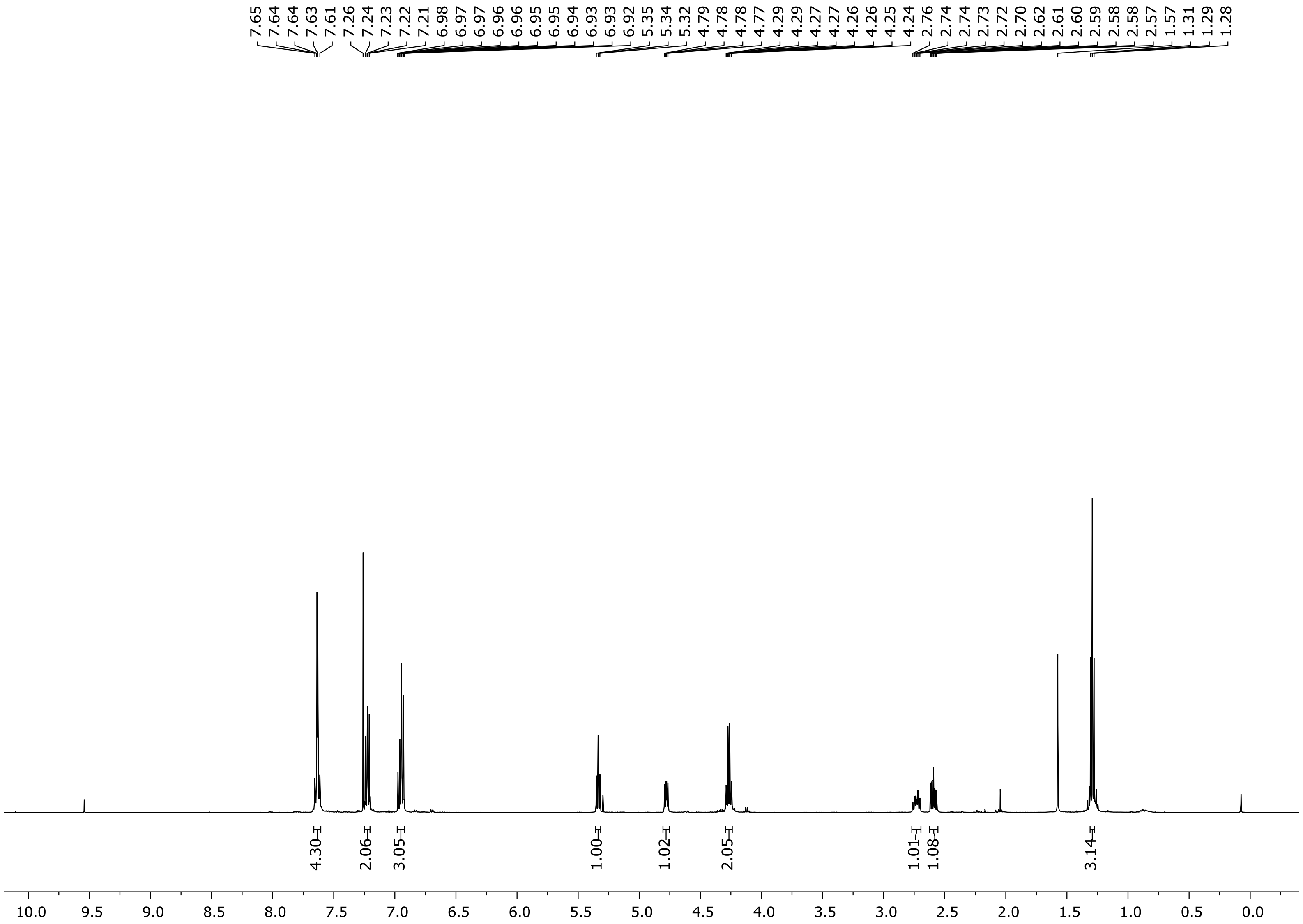

Figure S17: ^{13}C NMR (126 MHz, $CDCl_3$, 298 K) spectrum of **3d**.

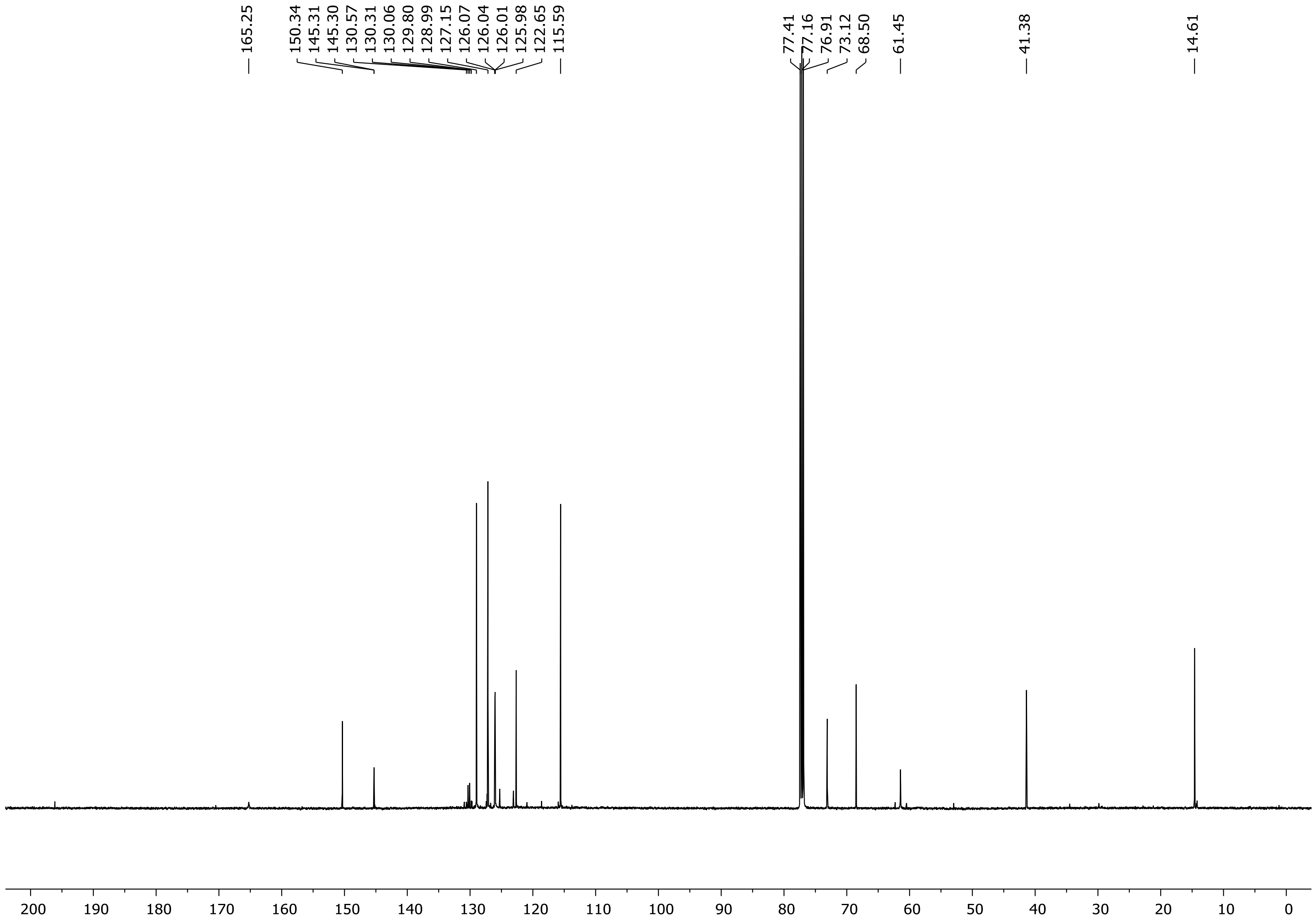

Figure S18: ^{19}F NMR (471 MHz, $CDCl_3$, 298 K) spectrum of **3d**.

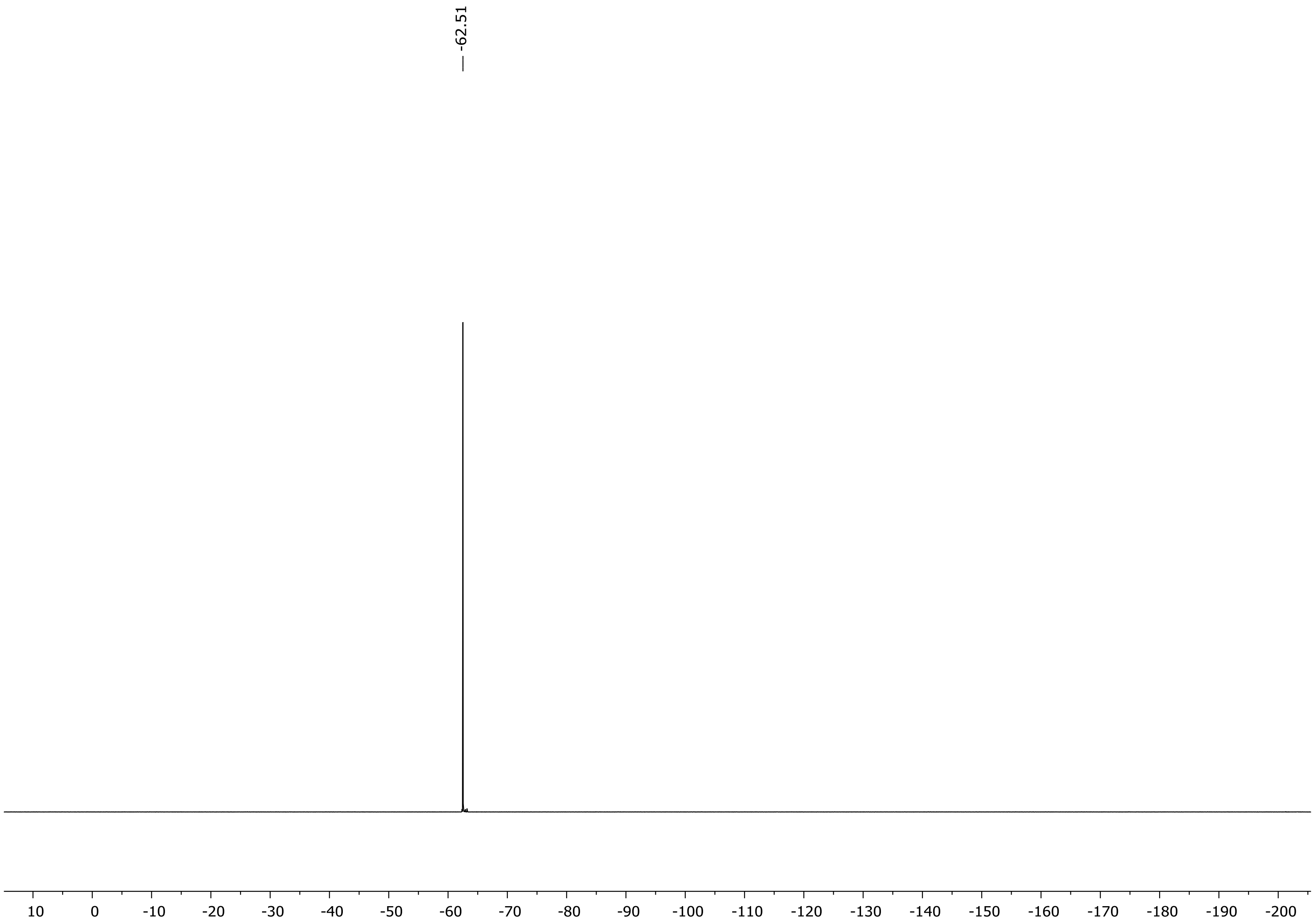

Figure S19: ^{1}H NMR (400 MHz, $CDCl_3$, 298 K) spectrum of **3e**.

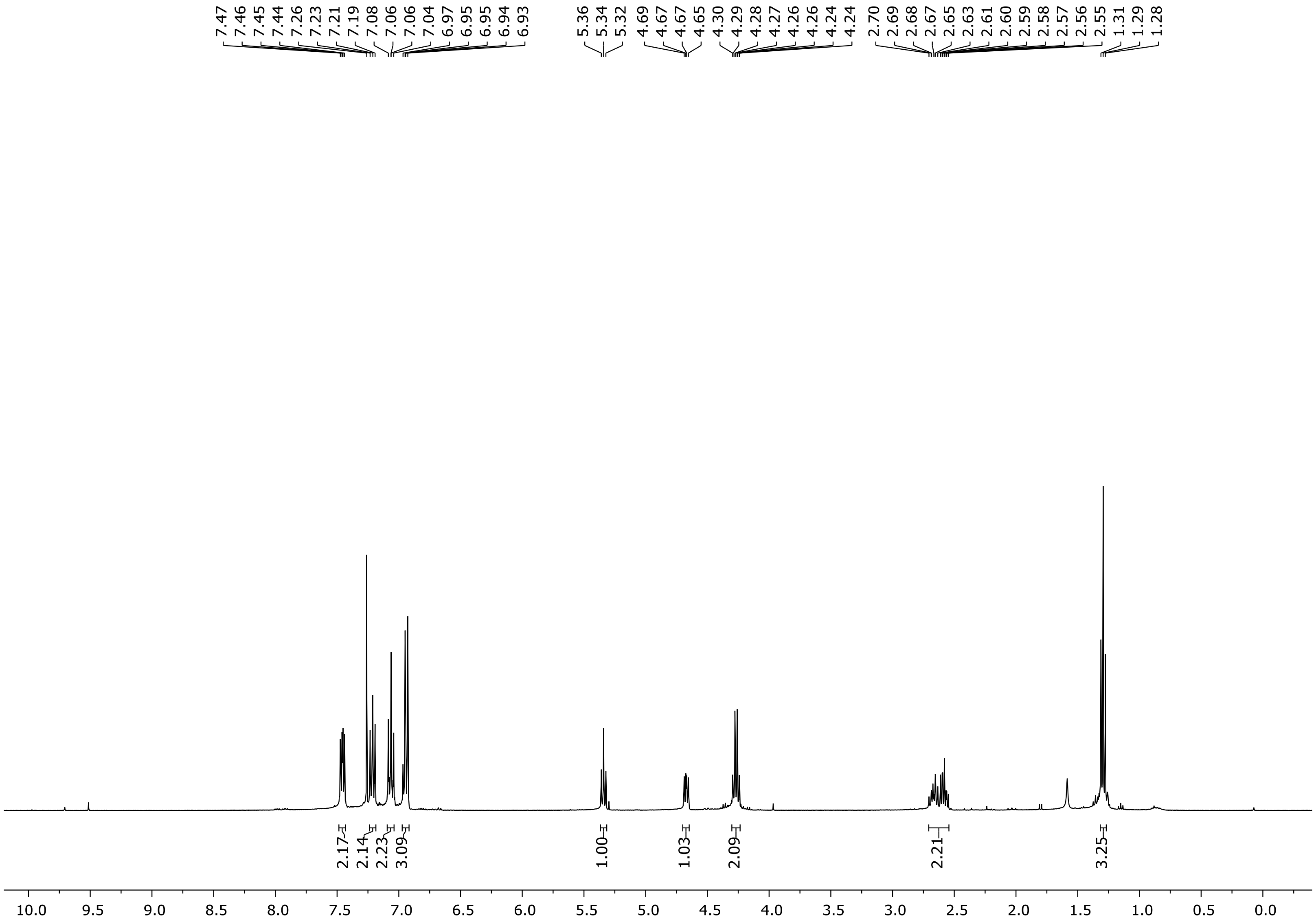

Figure S20: ^{13}C NMR (101 MHz, $CDCl_3$, 298 K) spectrum of **3e**.

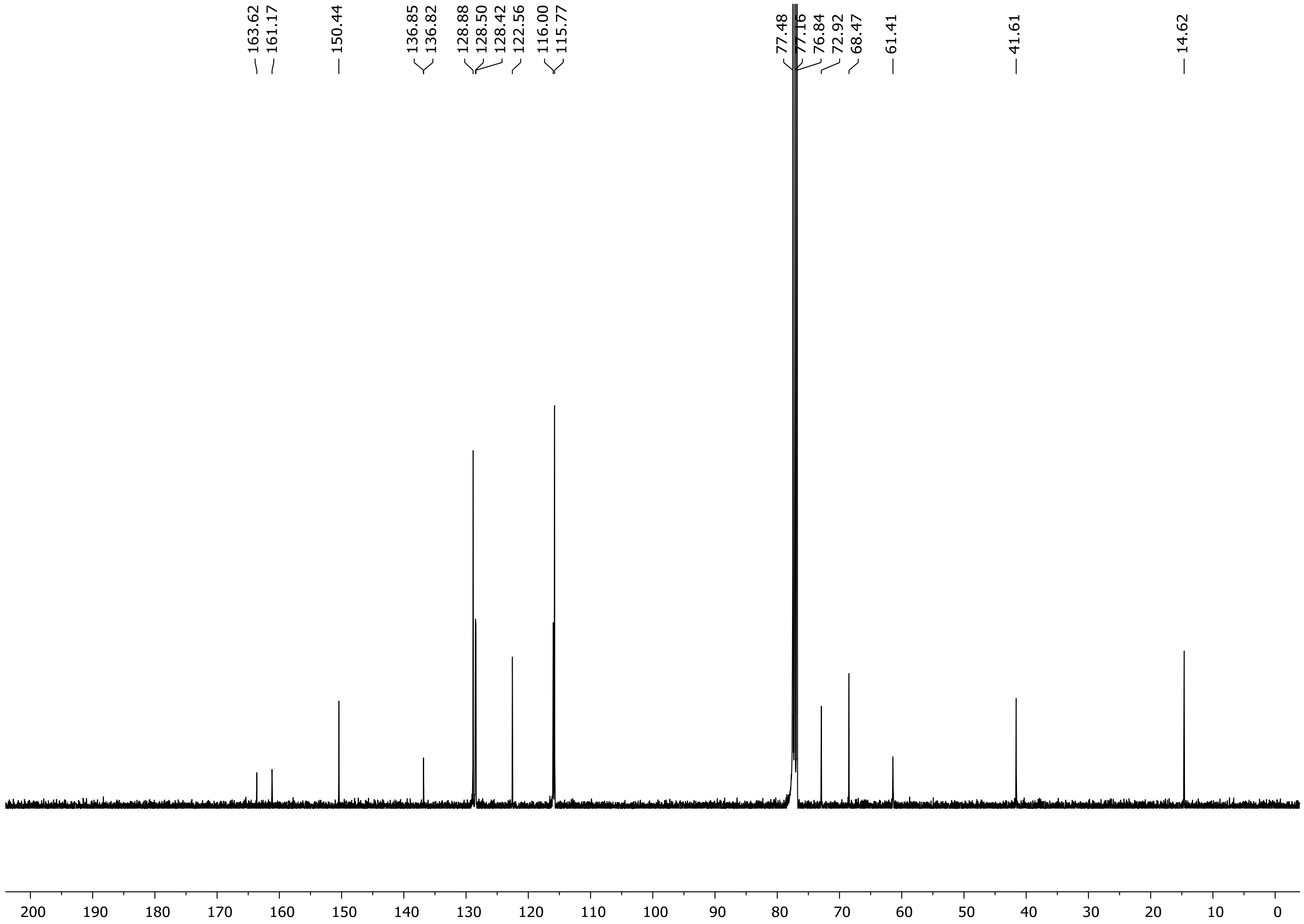

Figure S21: ^{19}F NMR (376 MHz, $CDCl_3$, 298 K) spectrum of **3e**.

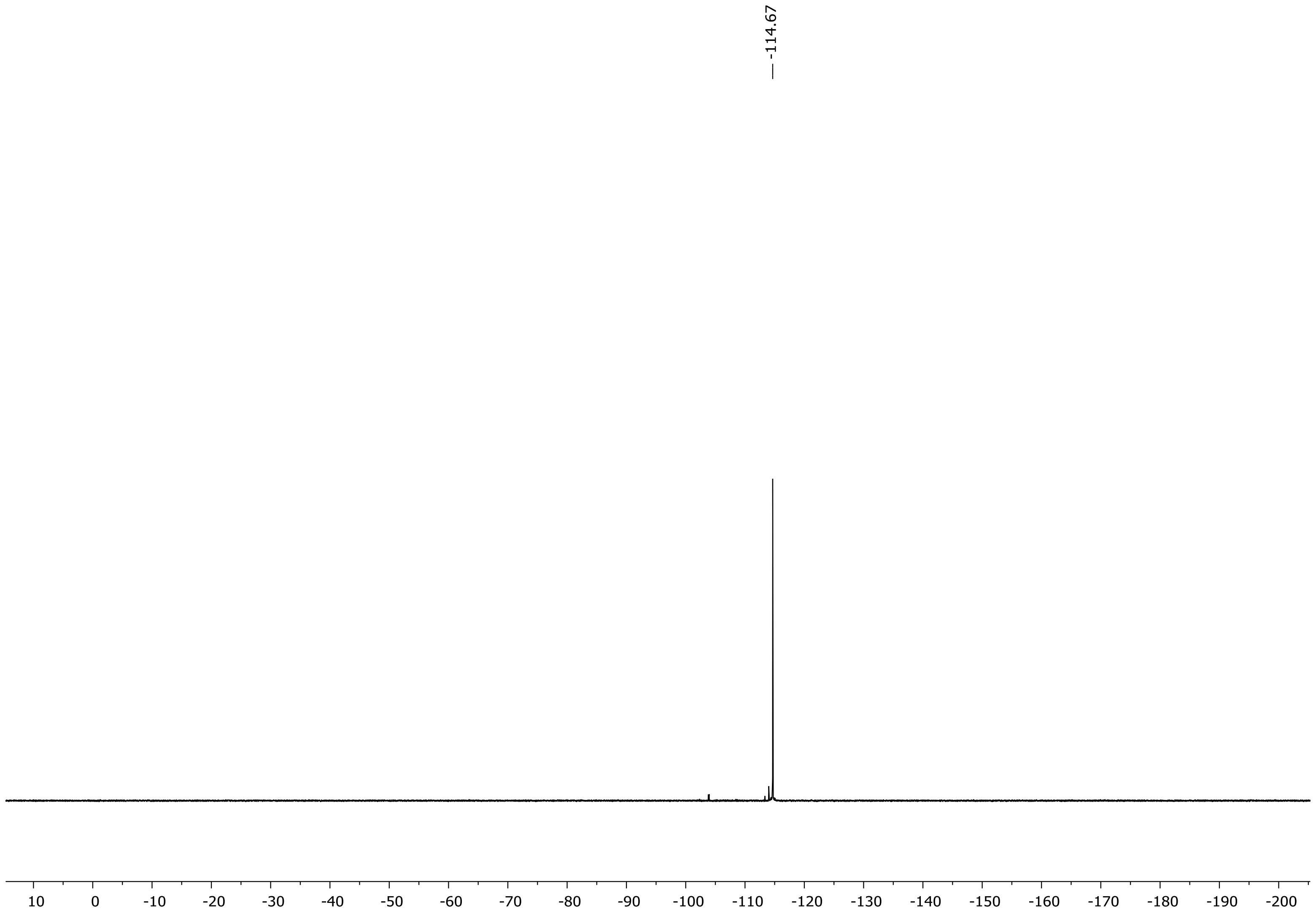

Figure S22: ^{1}H NMR (400 MHz, $CDCl_3$, 298 K) spectrum of **3f**.

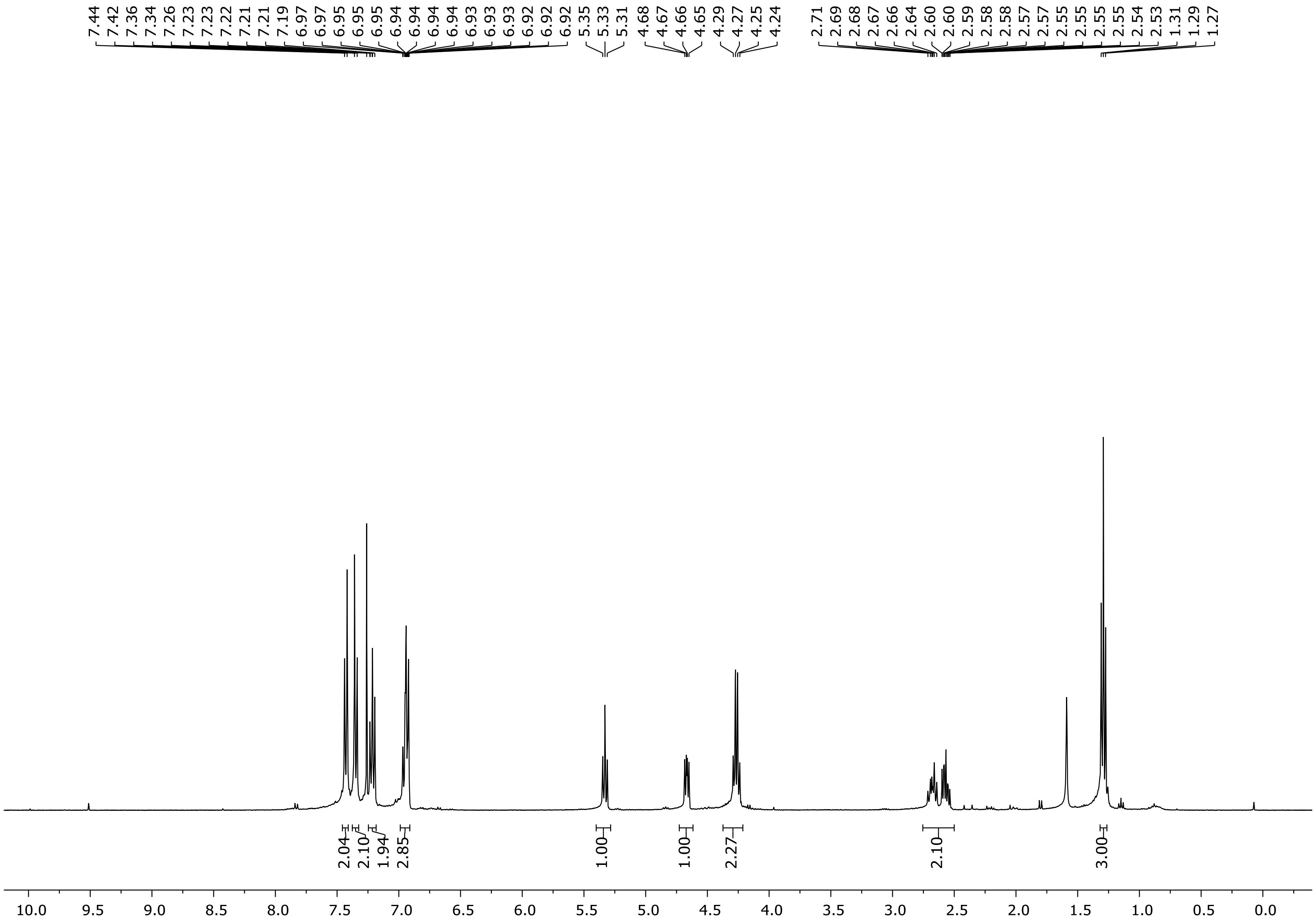

Figure S23: ^{13}C NMR (101 MHz, $CDCl_3$, 298 K) spectrum of **3f**.

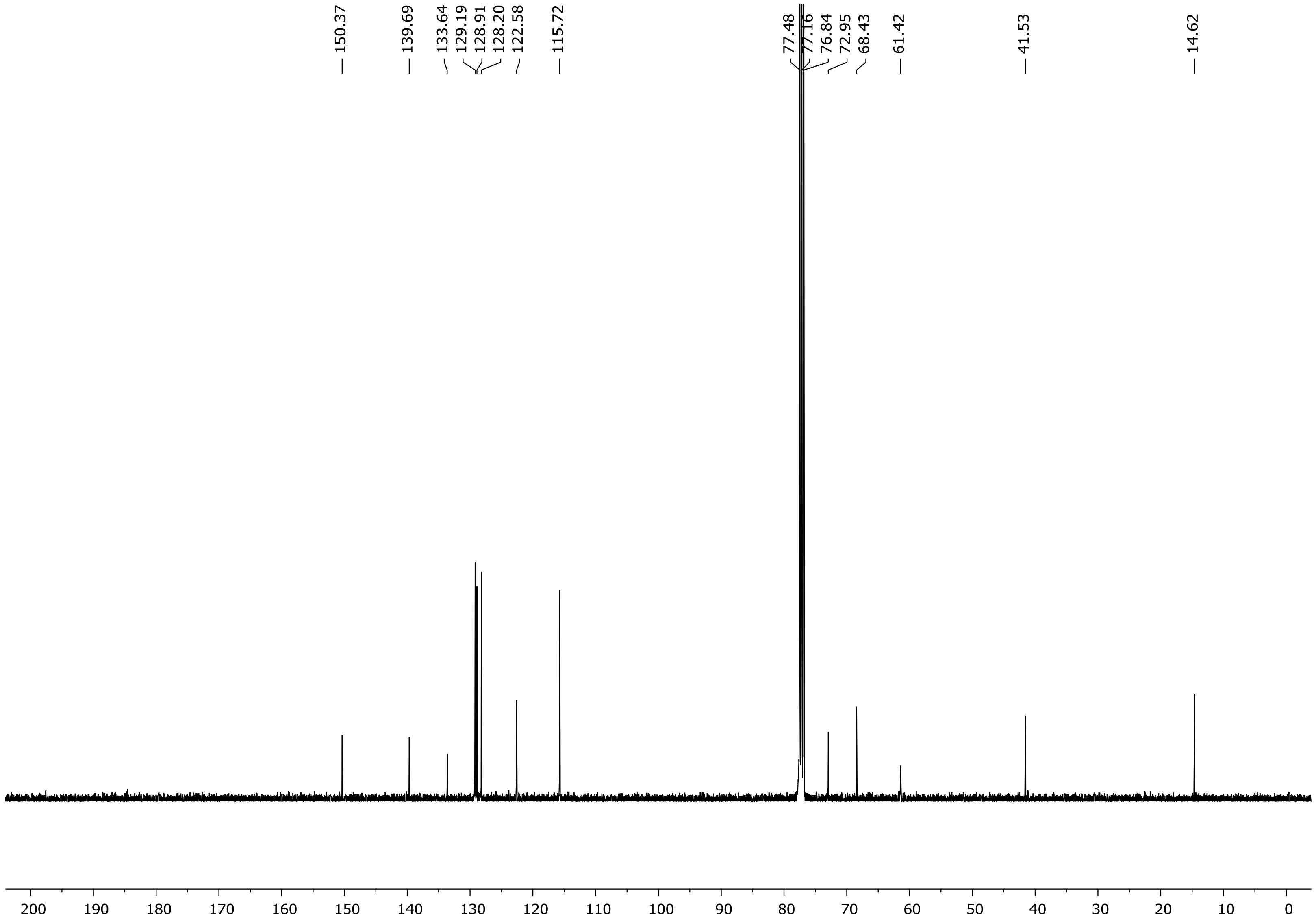

Figure S24: ^{1}H NMR (400 MHz, $CDCl_3$, 298 K) spectrum of **3g.**

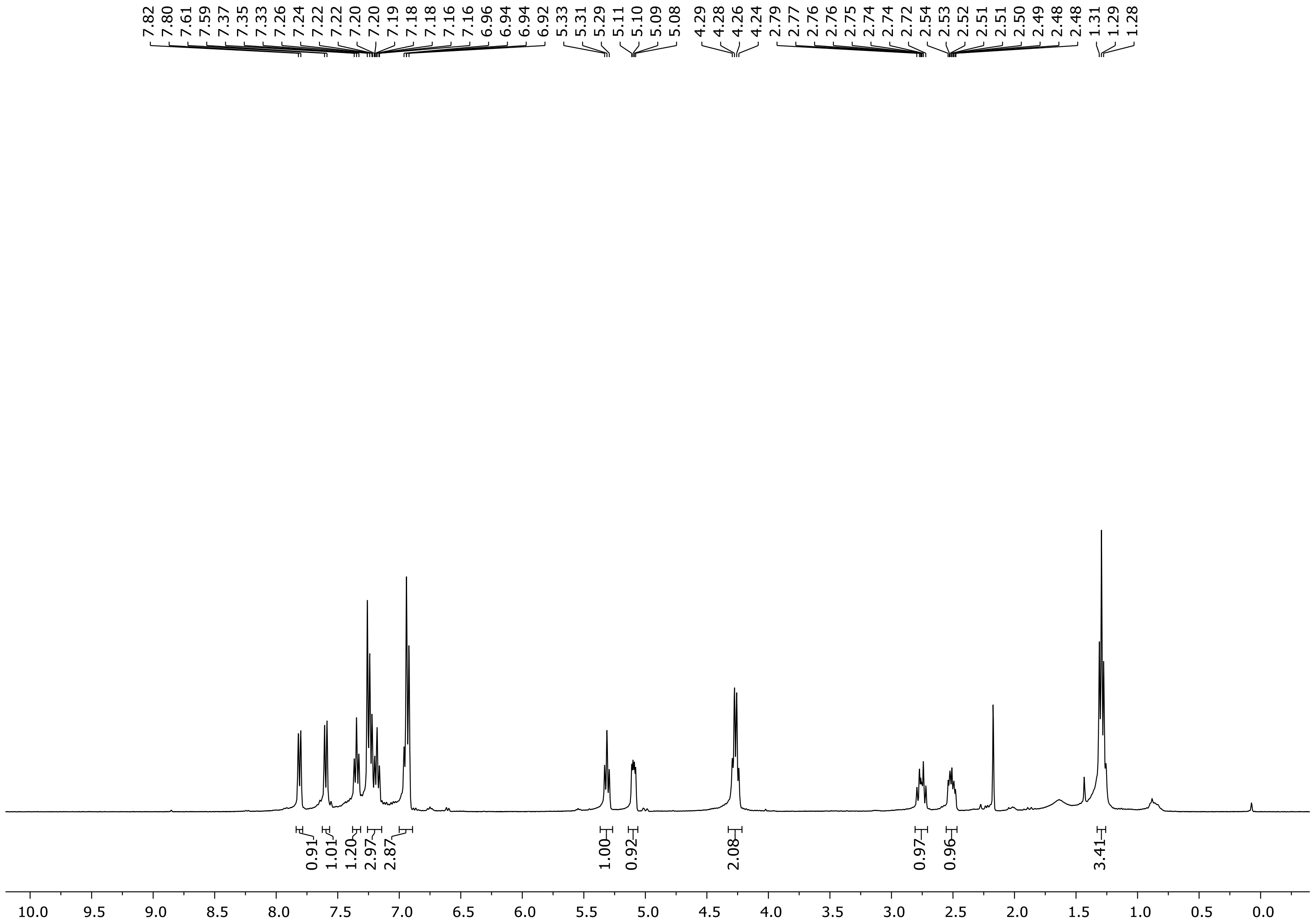

Figure S25: ^{13}C NMR (101 MHz, $CDCl_3$, 298 K) spectrum of **3g**.

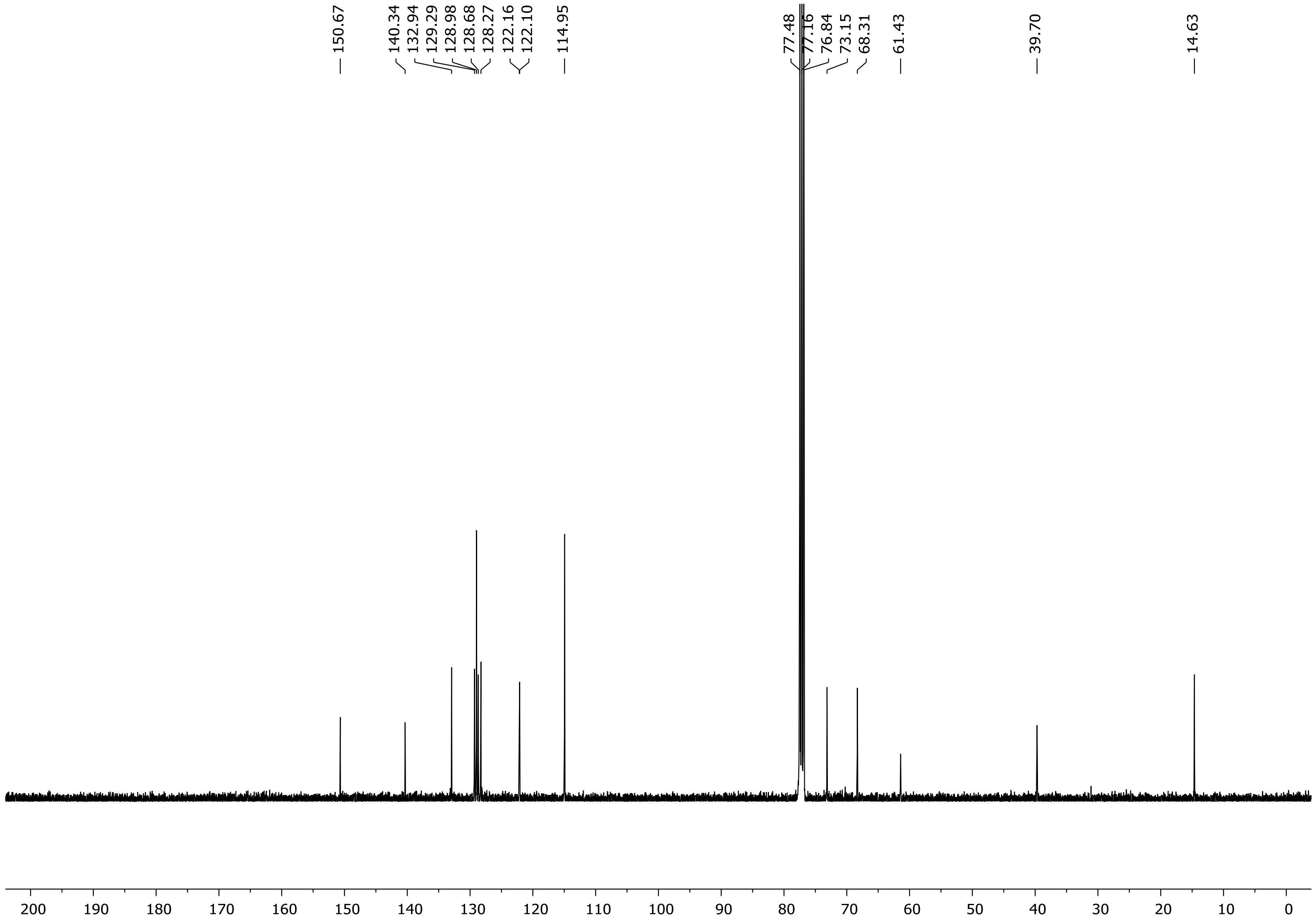

Figure S26: ^{1}H NMR (500 MHz, $CDCl_3$, 298 K) spectrum of **3h**.

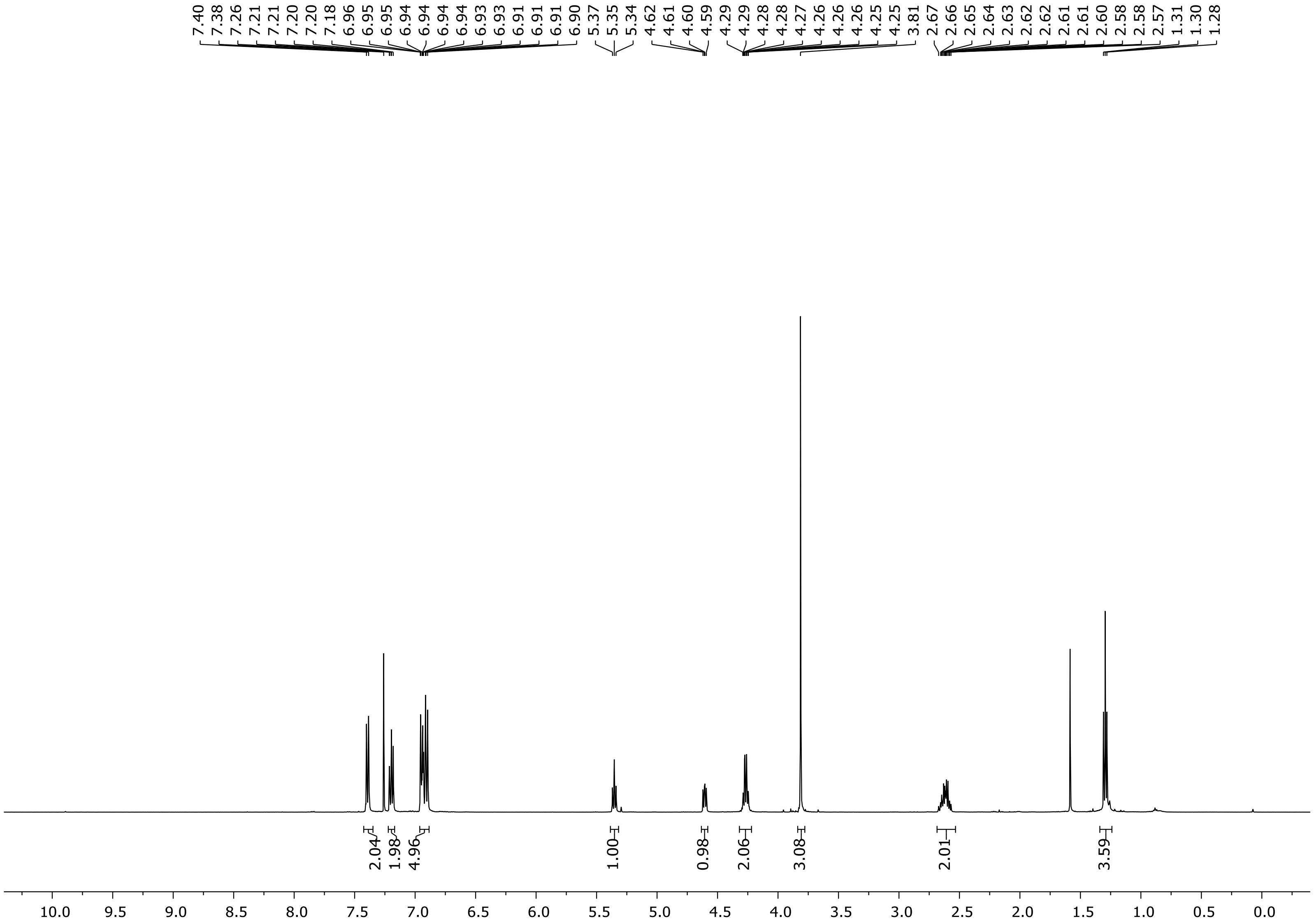

Figure S27: ^{13}C NMR (126 MHz, $CDCl_3$, 298 K) spectrum of **3h**.

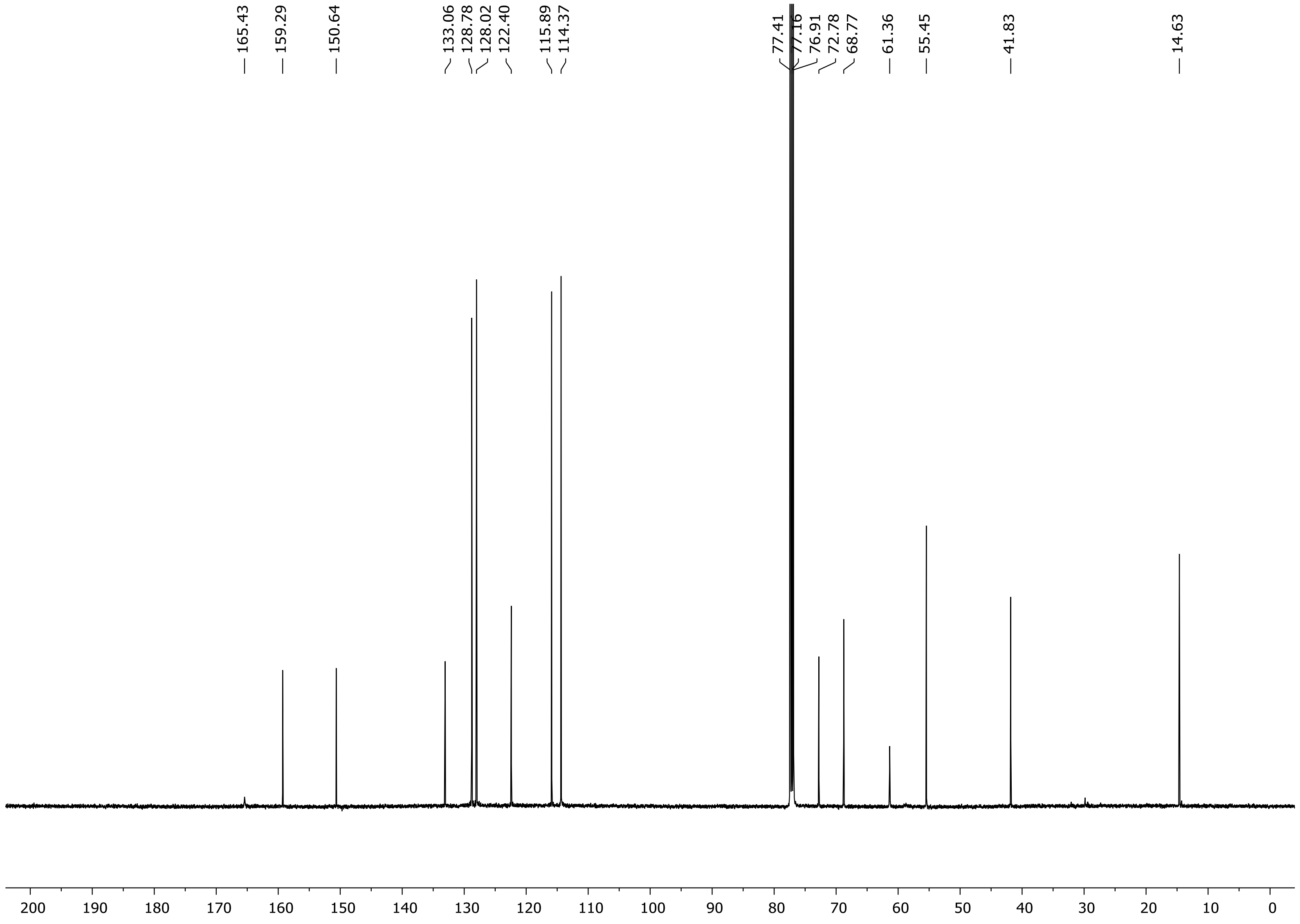

Figure S28: ^{1}H NMR (500 MHz, $CDCl_3$, 298 K) spectrum of **3i**

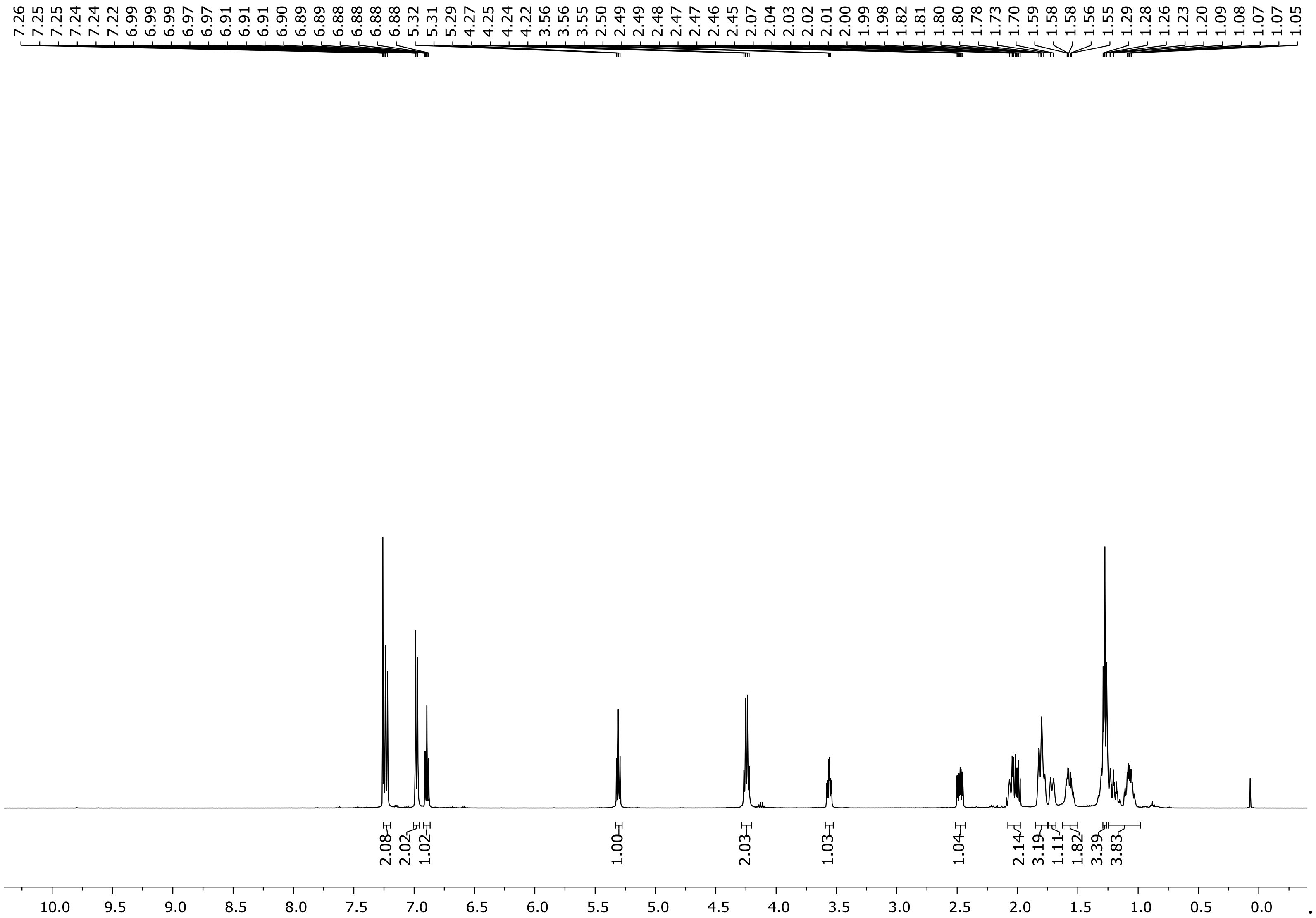

Figure S29: ^{13}C NMR (126 MHz, $CDCl_3$, 298 K) spectrum of **3i**.

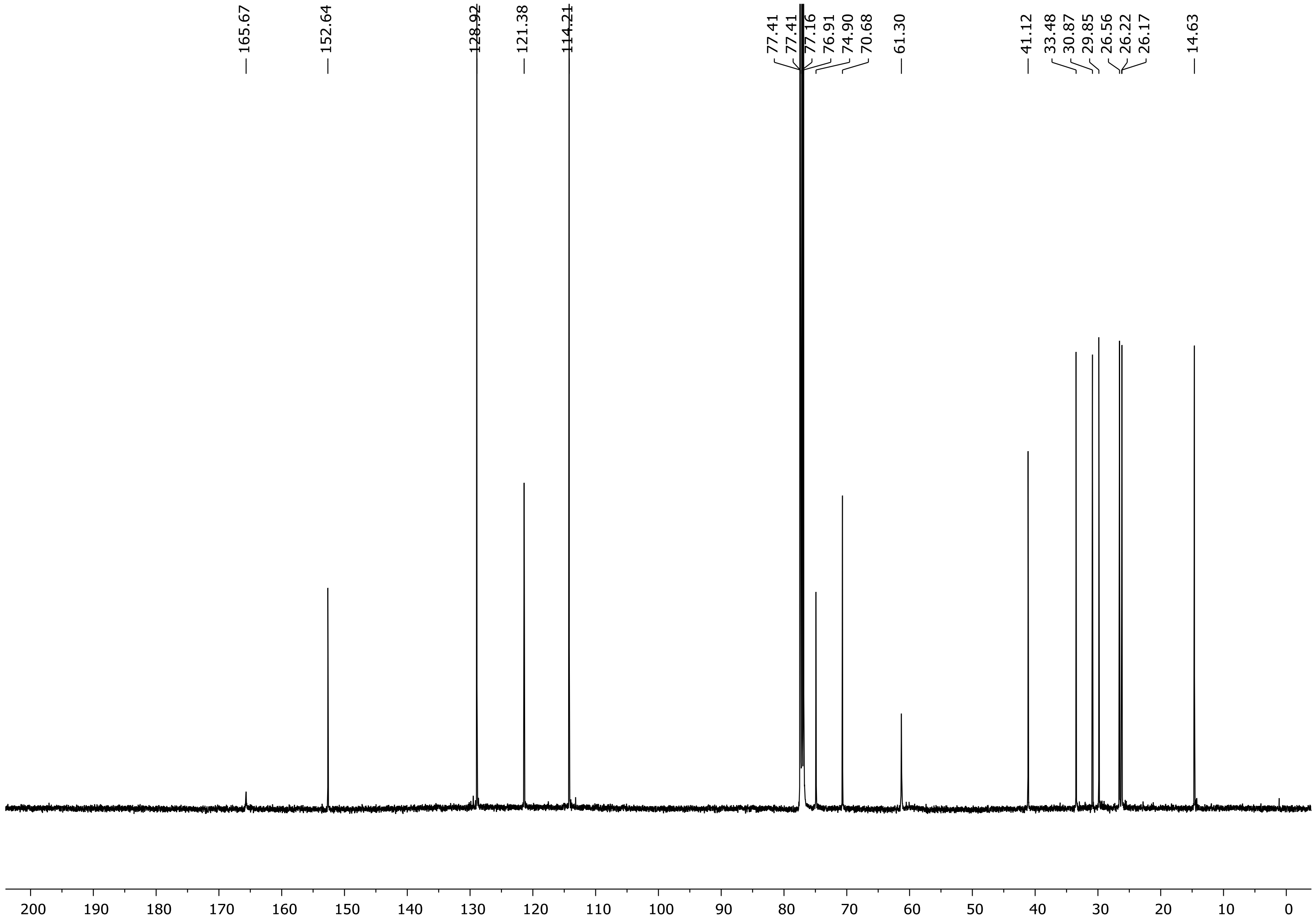

Figure S30: ^{1}H NMR (500 MHz, $CDCl_3$, 298 K) spectrum of **3j**

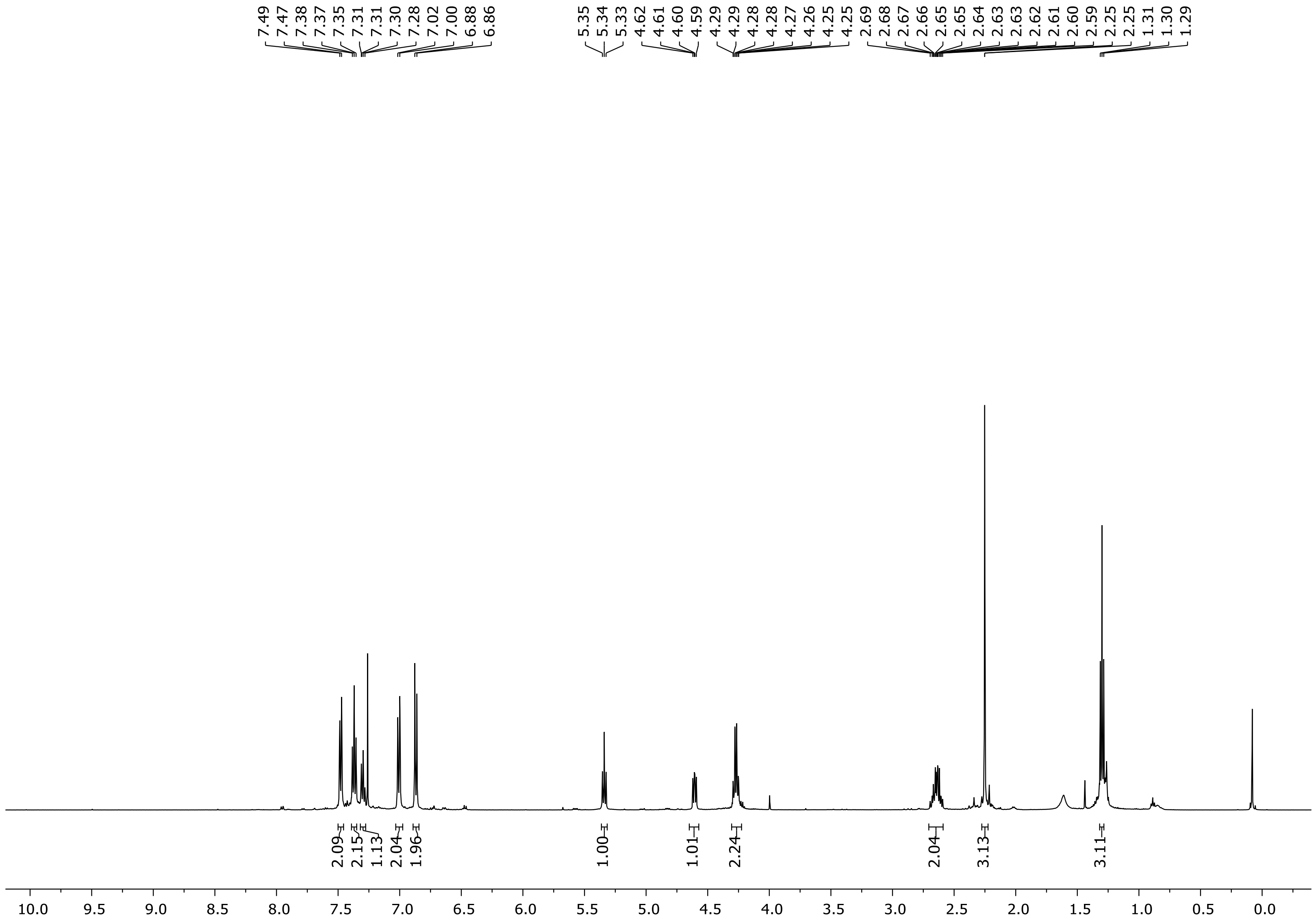

Figure S31: ^{13}C NMR (126 MHz, $CDCl_3$, 298 K) spectrum of **3j**.

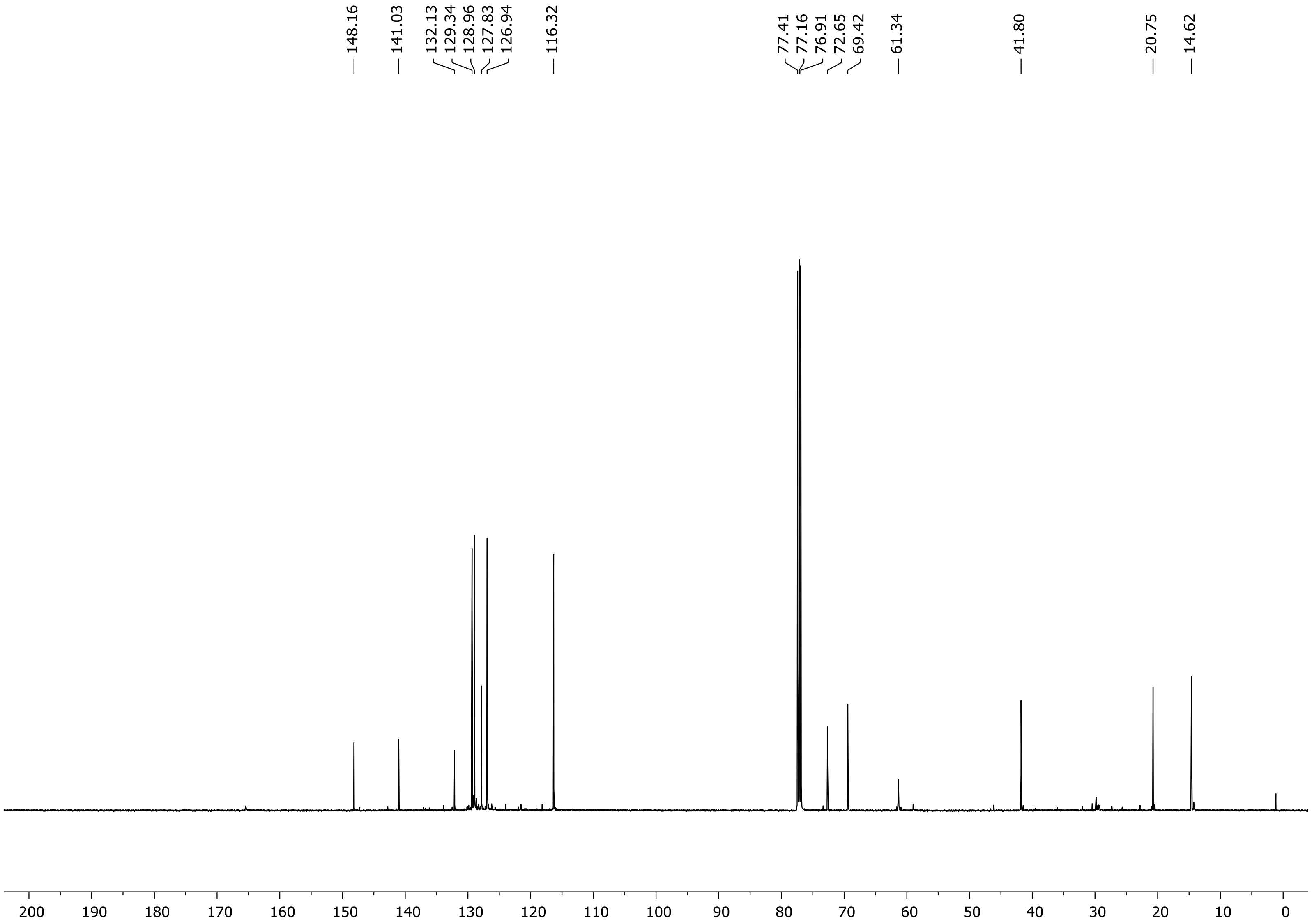

Figure S32: ^{1}H NMR (500 MHz, $CDCl_3$, 298 K) spectrum of **3k.**

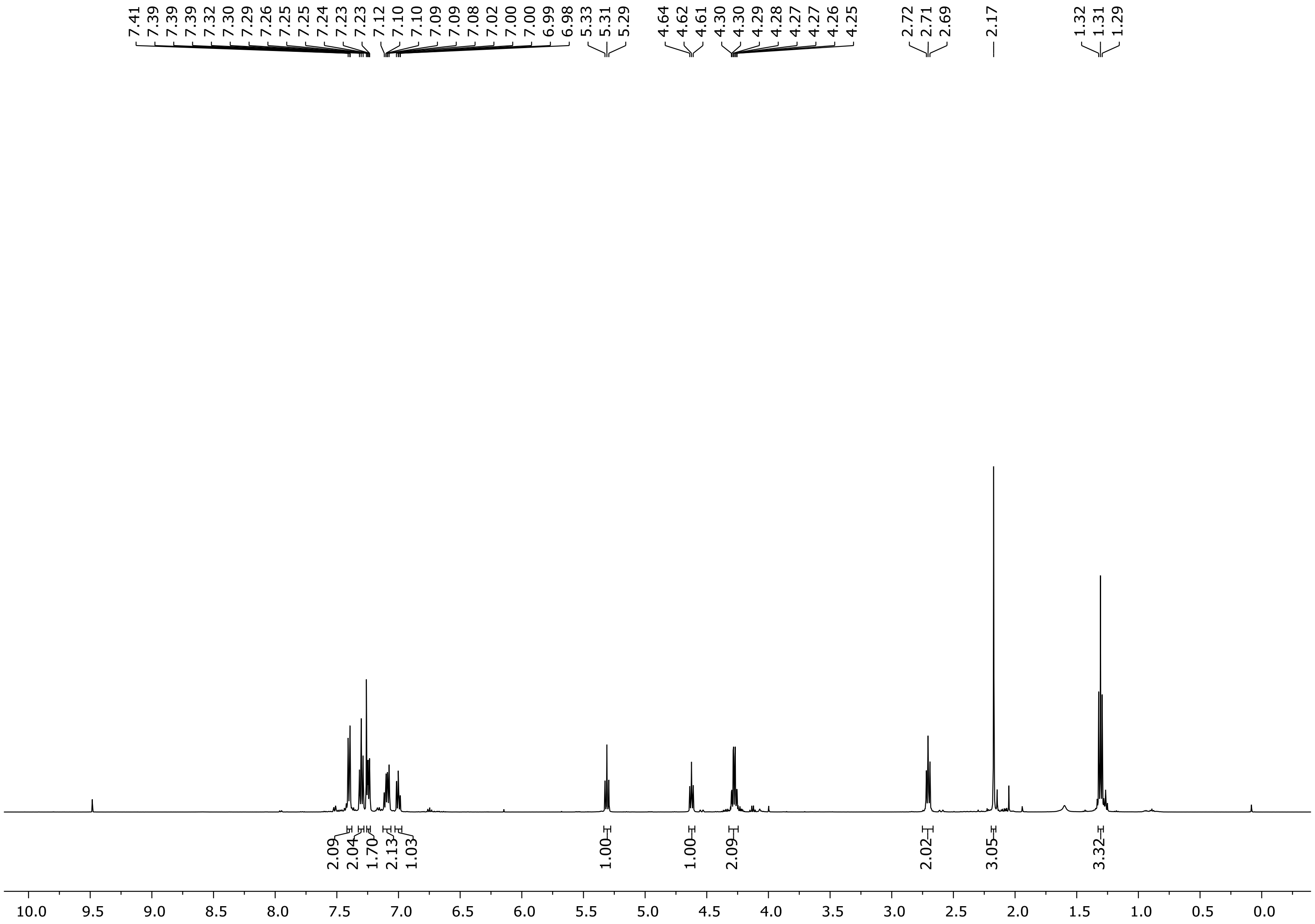

Figure S33: ^{13}C NMR (126 MHz, $CDCl_3$, 298 K) spectrum of **3k**.

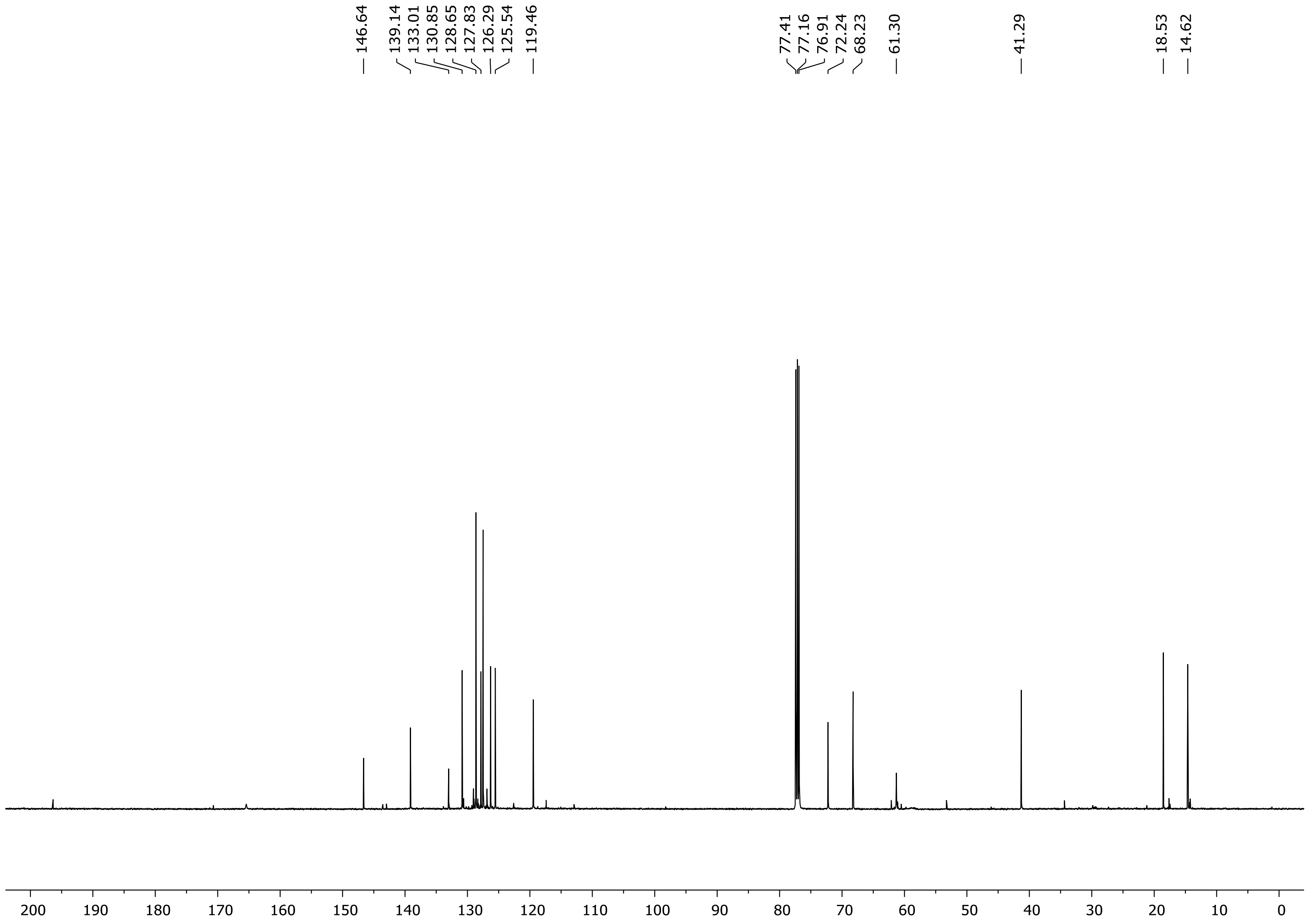

Figure S34: ^{1}H NMR (500 MHz, $CDCl_3$, 298 K) spectrum of **3l.**

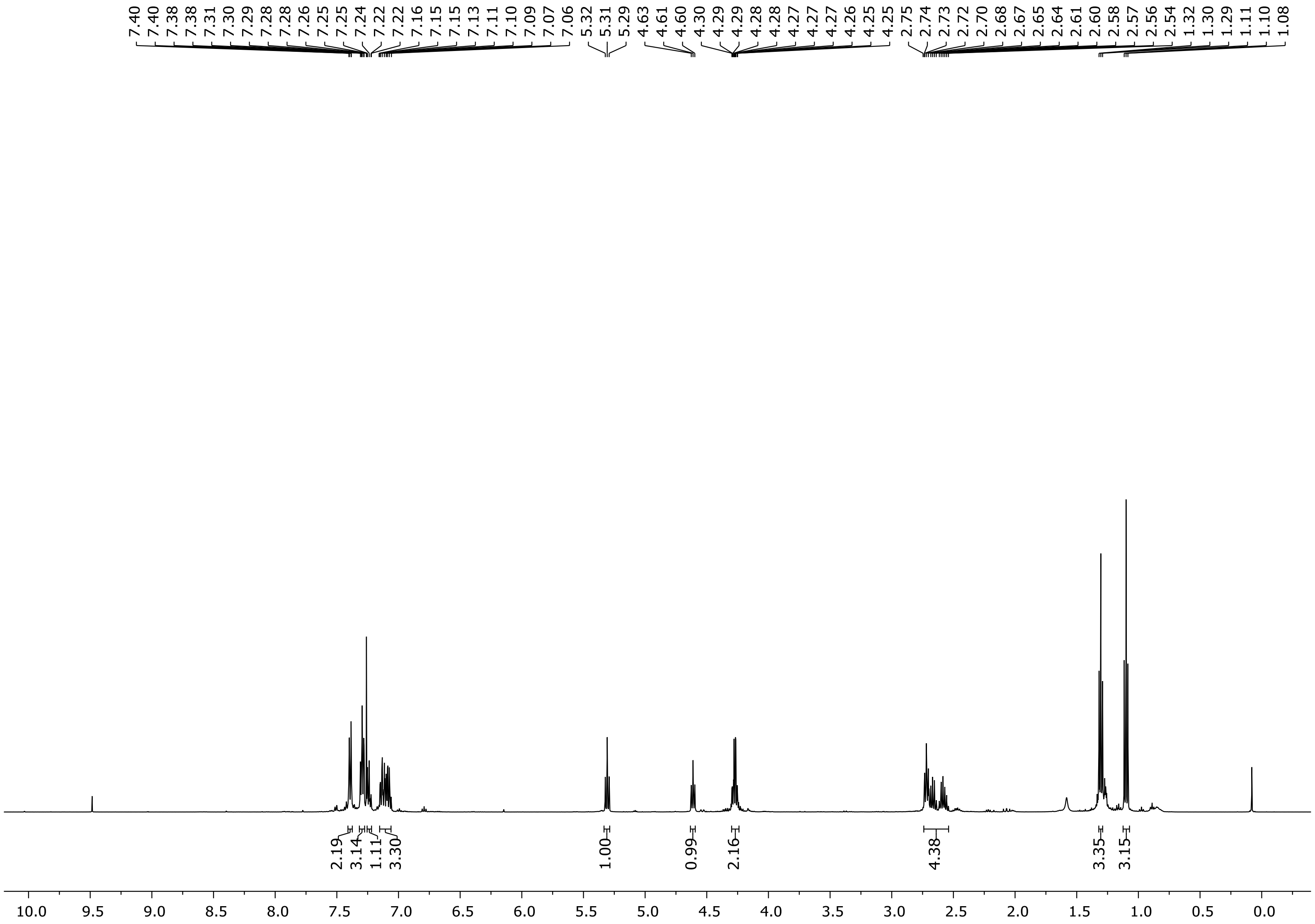

Figure S35: ^{13}C NMR (126 MHz, $CDCl_3$, 298 K) spectrum of **3l**.

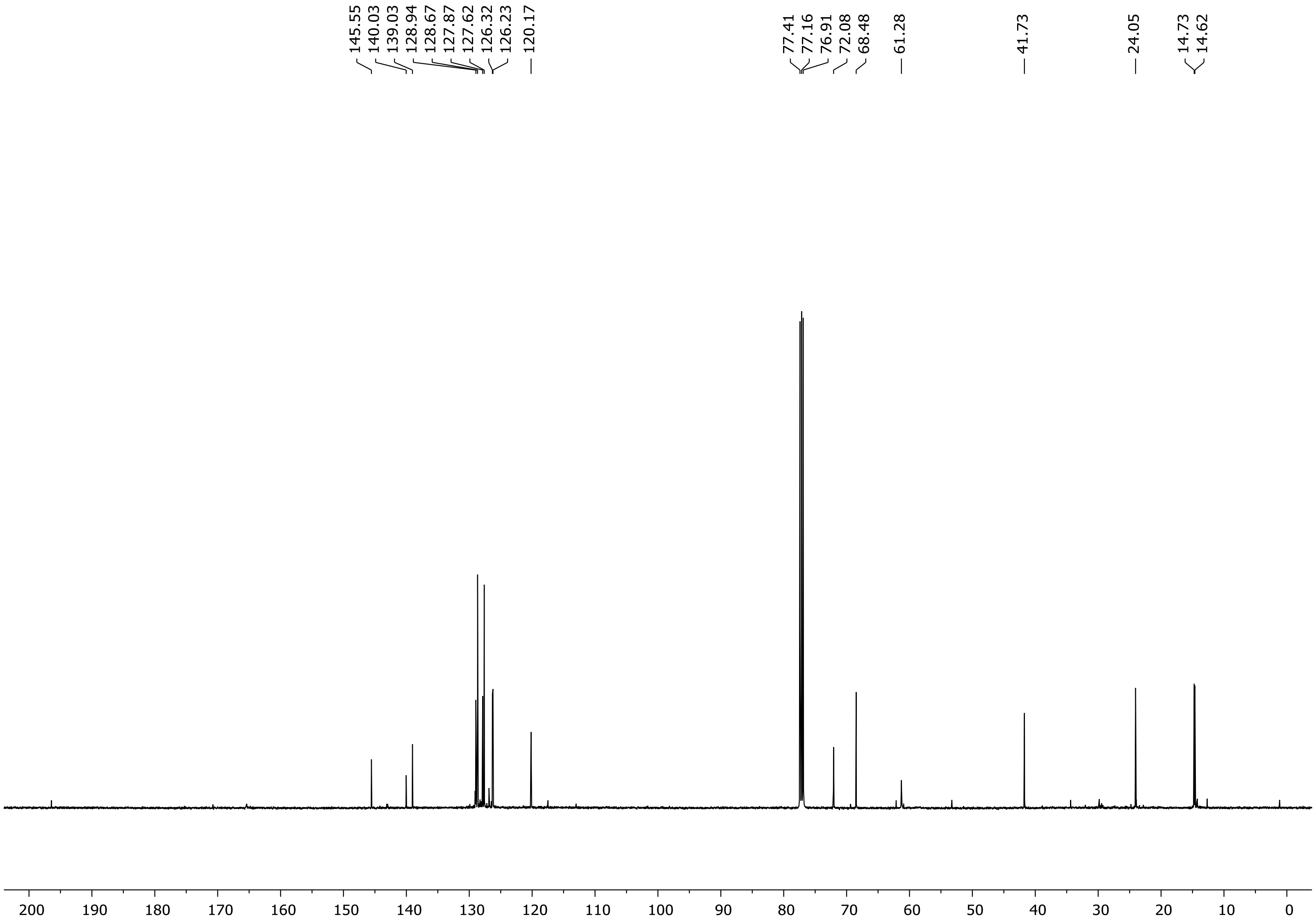

Figure S36: ^{1}H NMR (400 MHz, $CDCl_3$, 298 K) spectrum of **3m.**

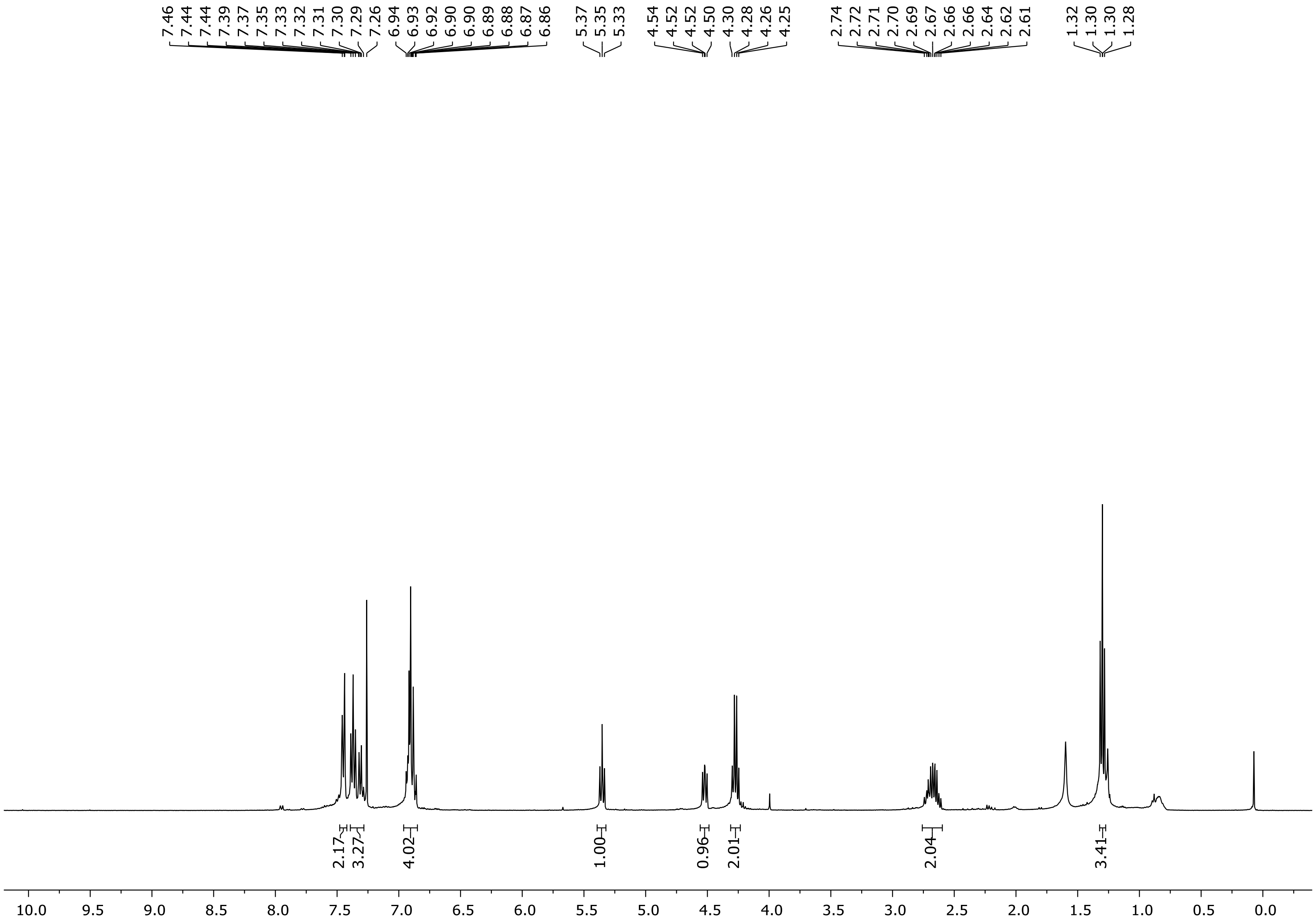

Figure S37: ^{13}C NMR (101 MHz, $CDCl_3$, 298 K) spectrum of **3m**

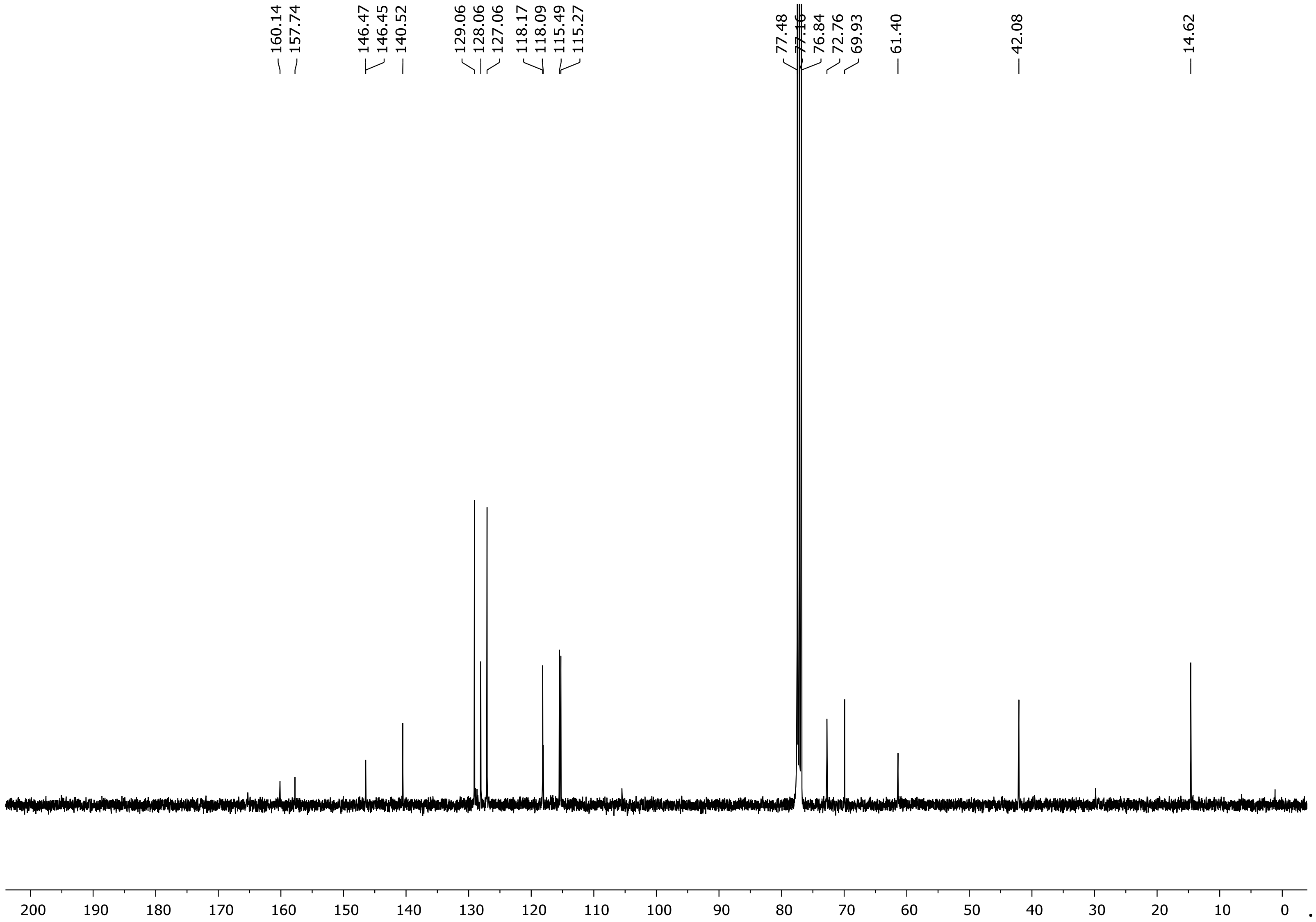

Figure S38: ^{19}F NMR (376 MHz, $CDCl_3$, 298 K) spectrum of **3m**.

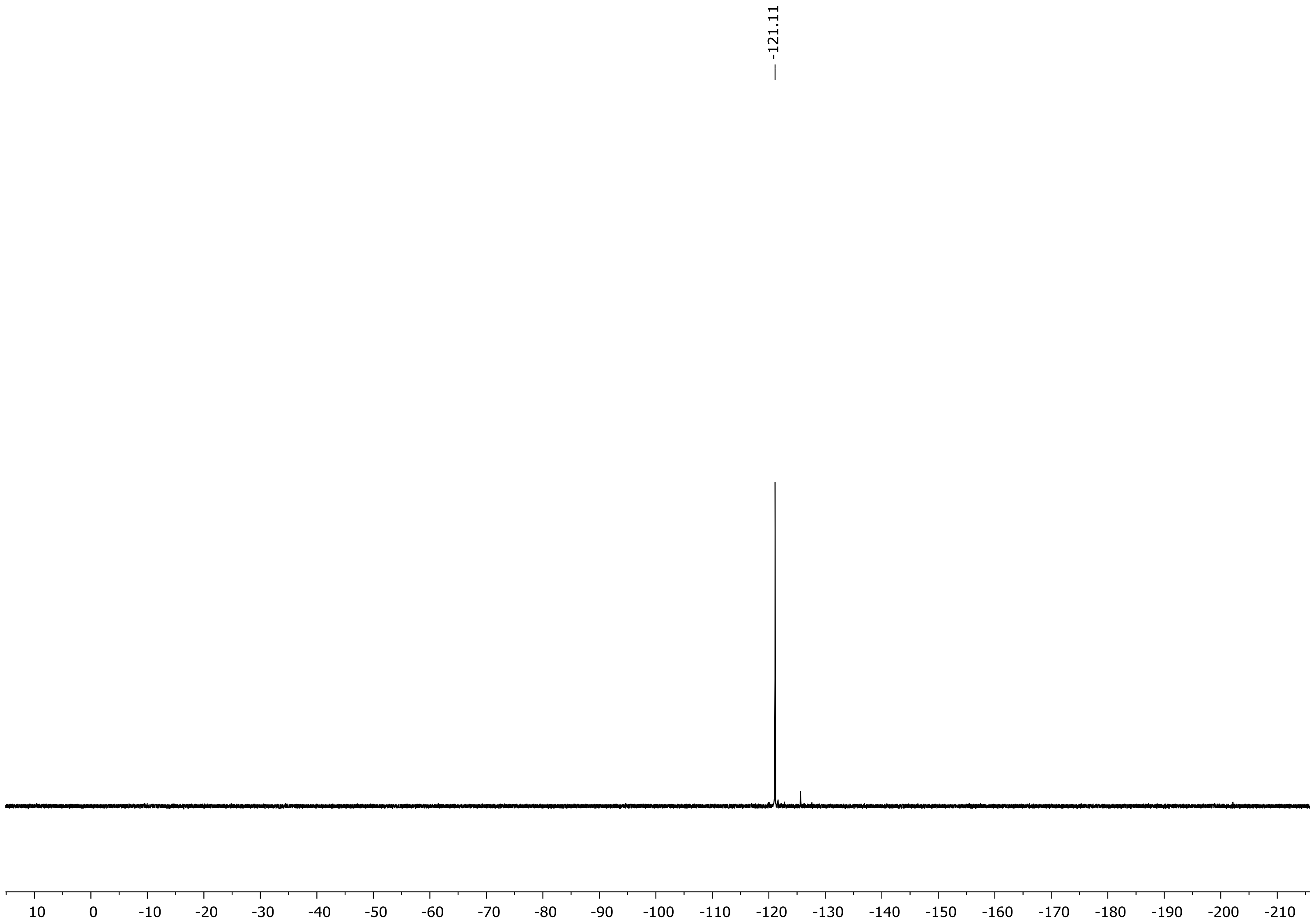

Figure S39: ^{1}H NMR (500 MHz, $CDCl_3$, 298 K) spectrum of *anti*-**3n.**

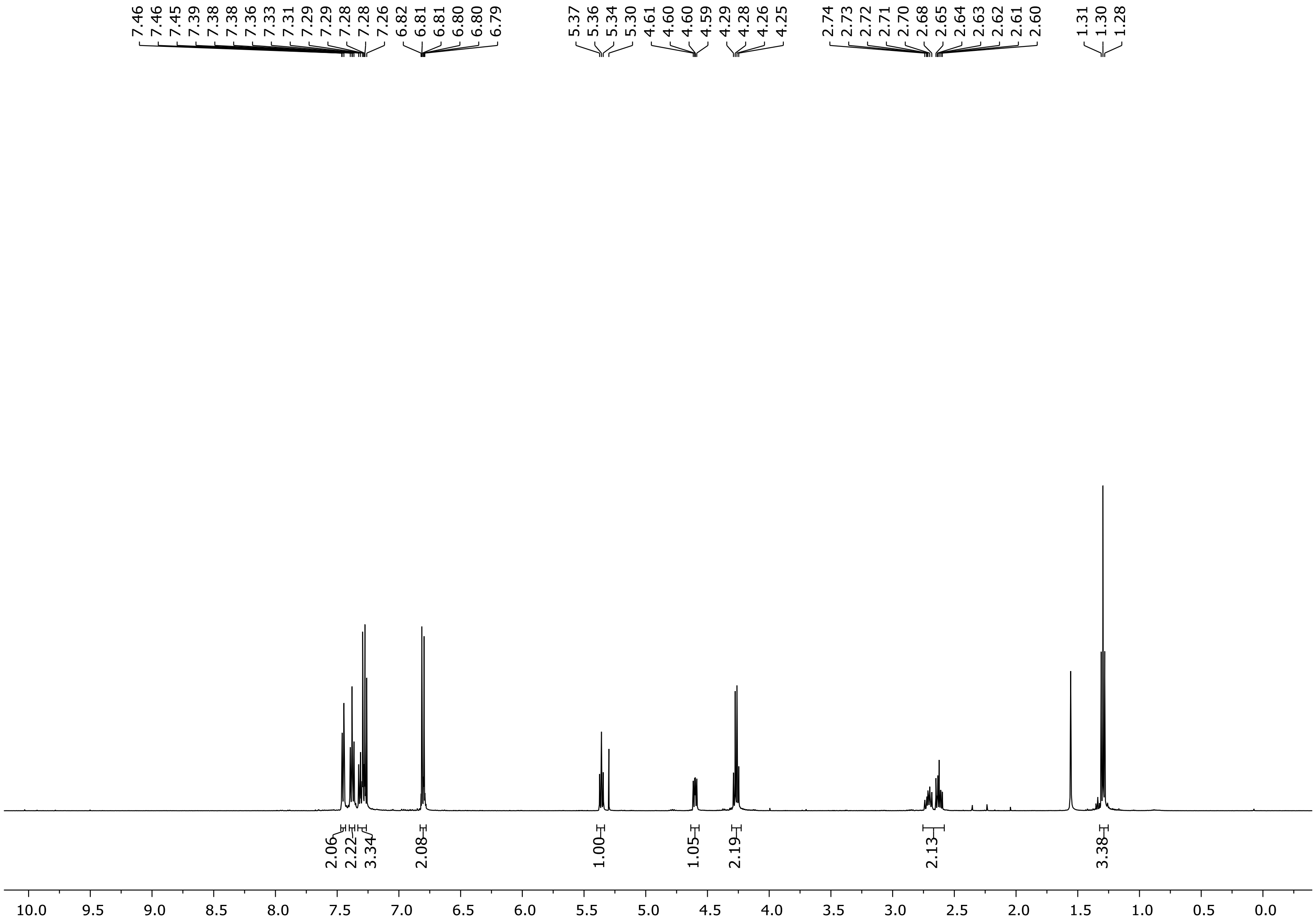

Figure S40: ^{13}C NMR (126 MHz, $CDCl_3$, 298 K) spectrum of *anti*-**3n**.

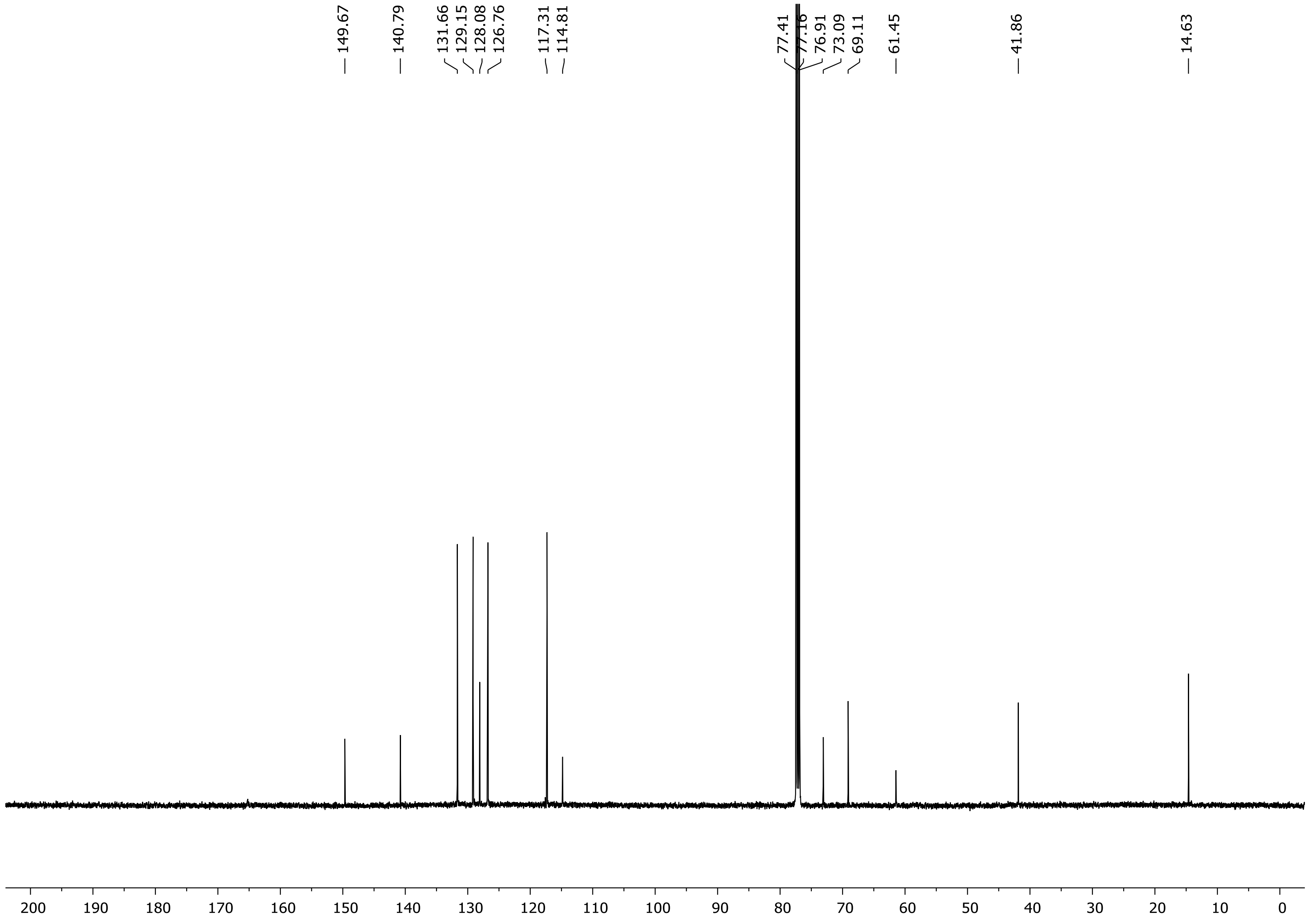

Figure S41: ^{1}H NMR (500 MHz, $CDCl_3$, 298 K) spectrum of *syn*-**3n.**

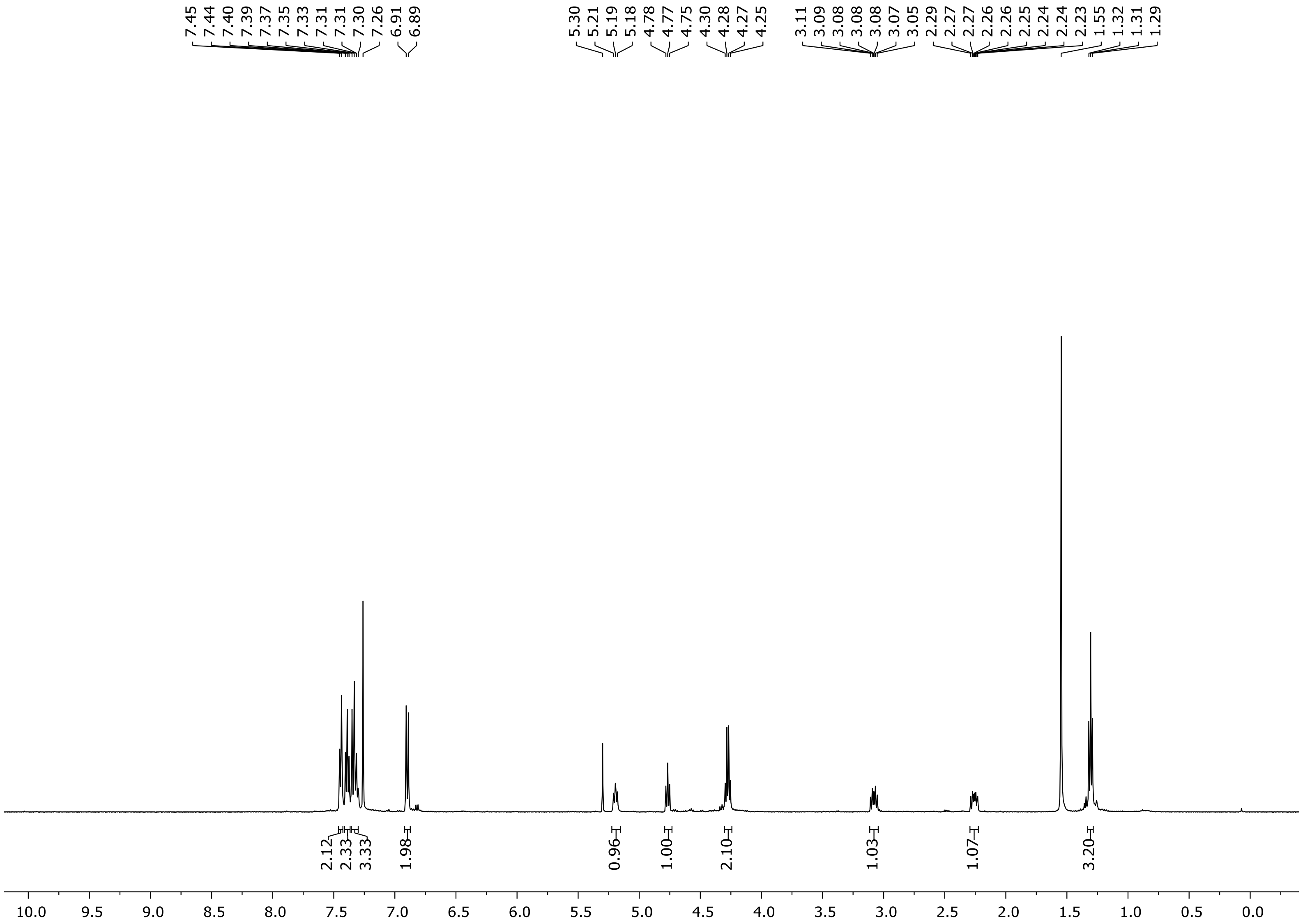

Figure S42: ^{13}C NMR (126 MHz, $CDCl_3$, 298 K) spectrum of *syn*-**3n**.

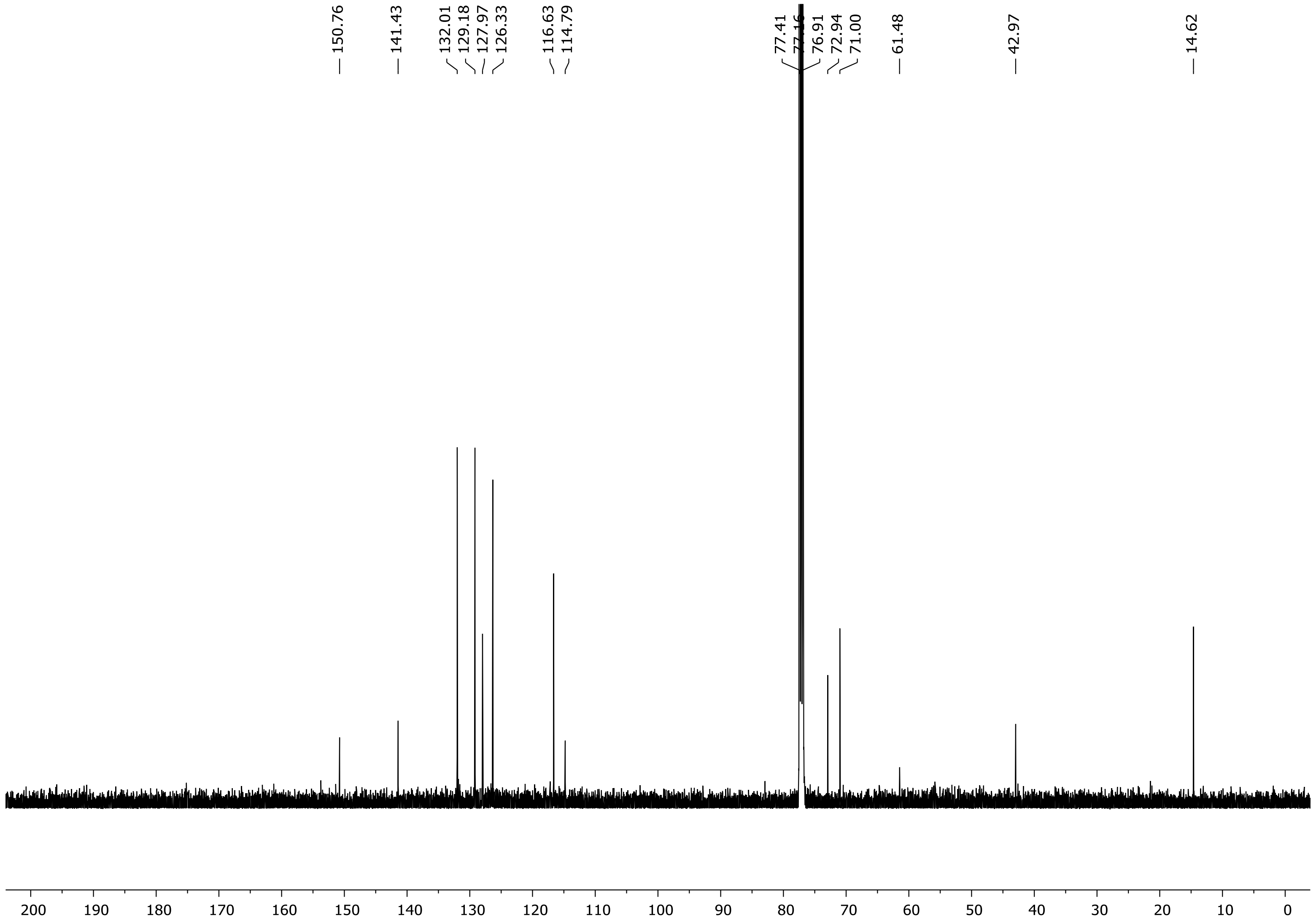

Figure S43: ^{1}H NMR (500 MHz, $CDCl_3$, 298 K) spectrum of **3o.**

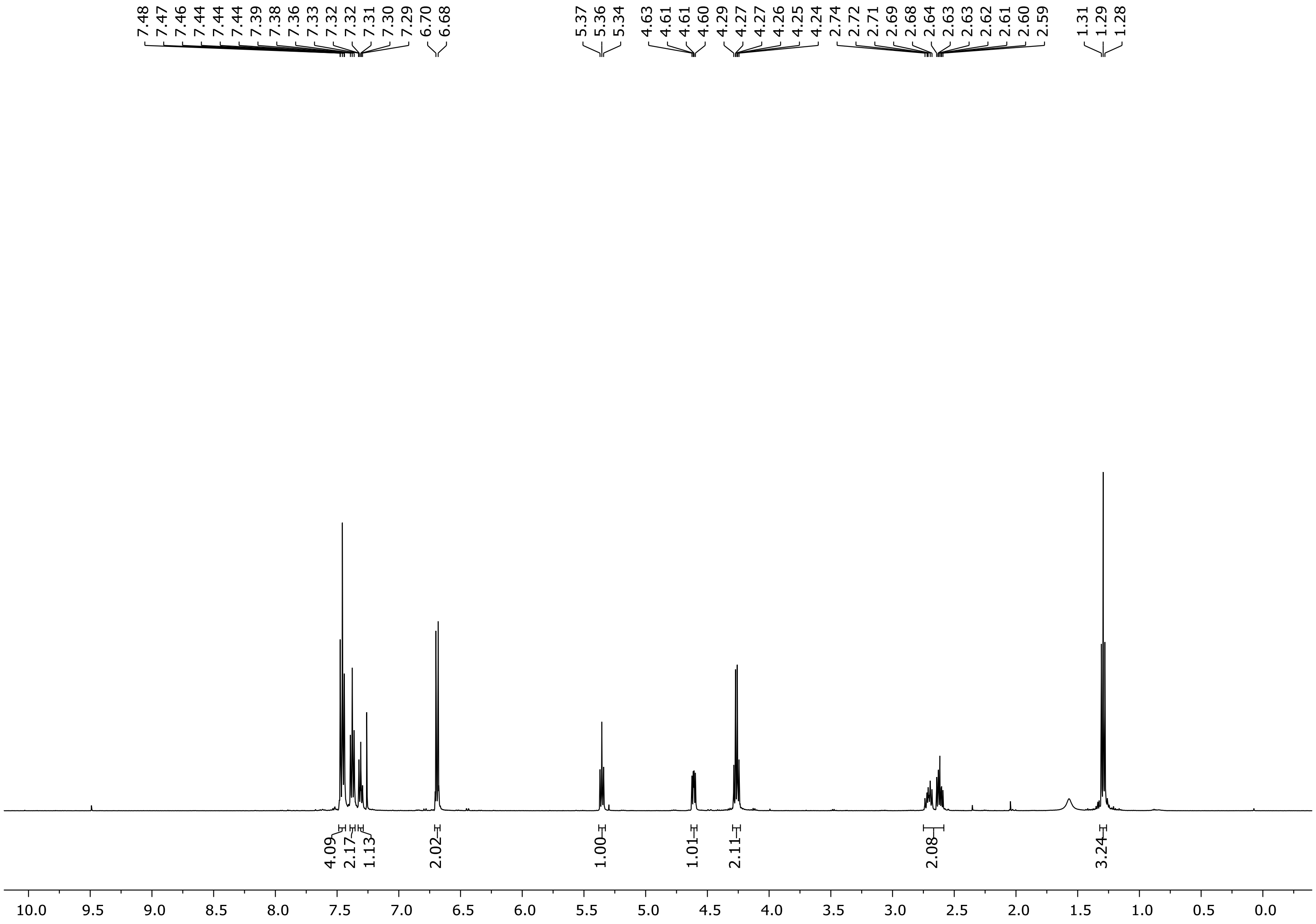

Figure S44: ^{13}C NMR (126 MHz, $CDCl_3$, 298 K) spectrum of **3o**.

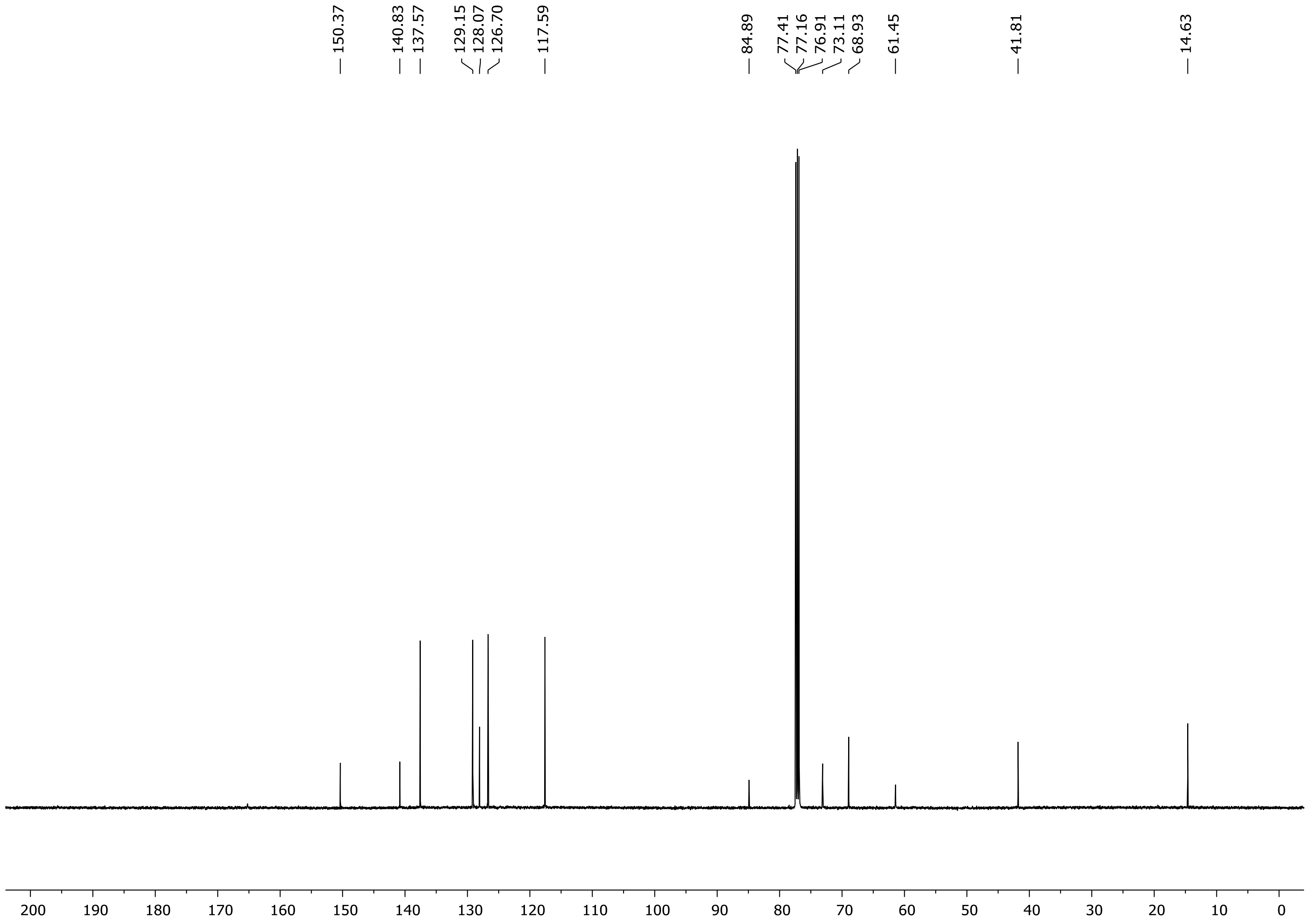

Figure S45: ^{1}H NMR (500 MHz, $CDCl_3$, 298 K) spectrum of **3p.**

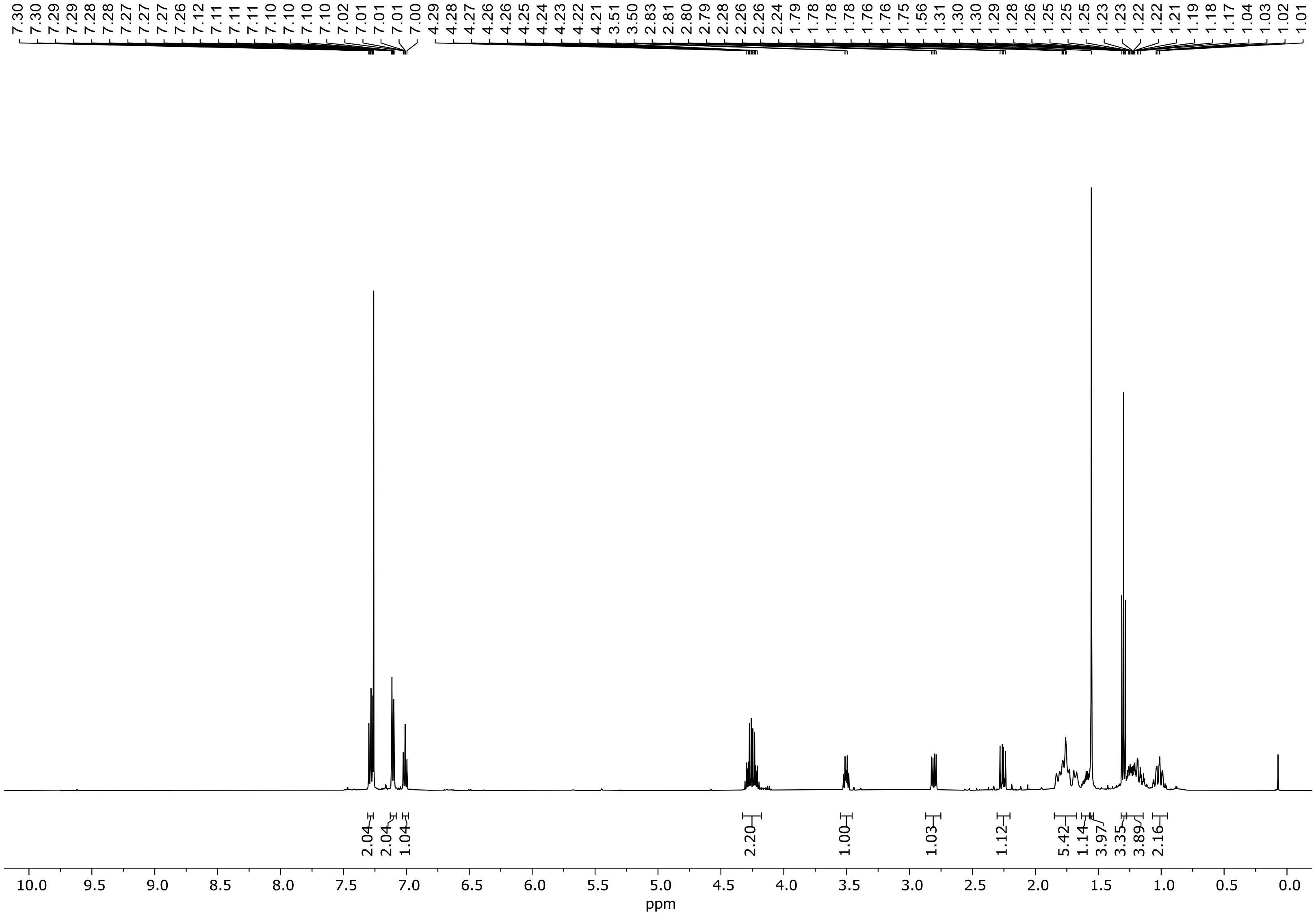

Figure S46: ^{13}C NMR (126 MHz, $CDCl_3$, 298 K) spectrum of **3p**.

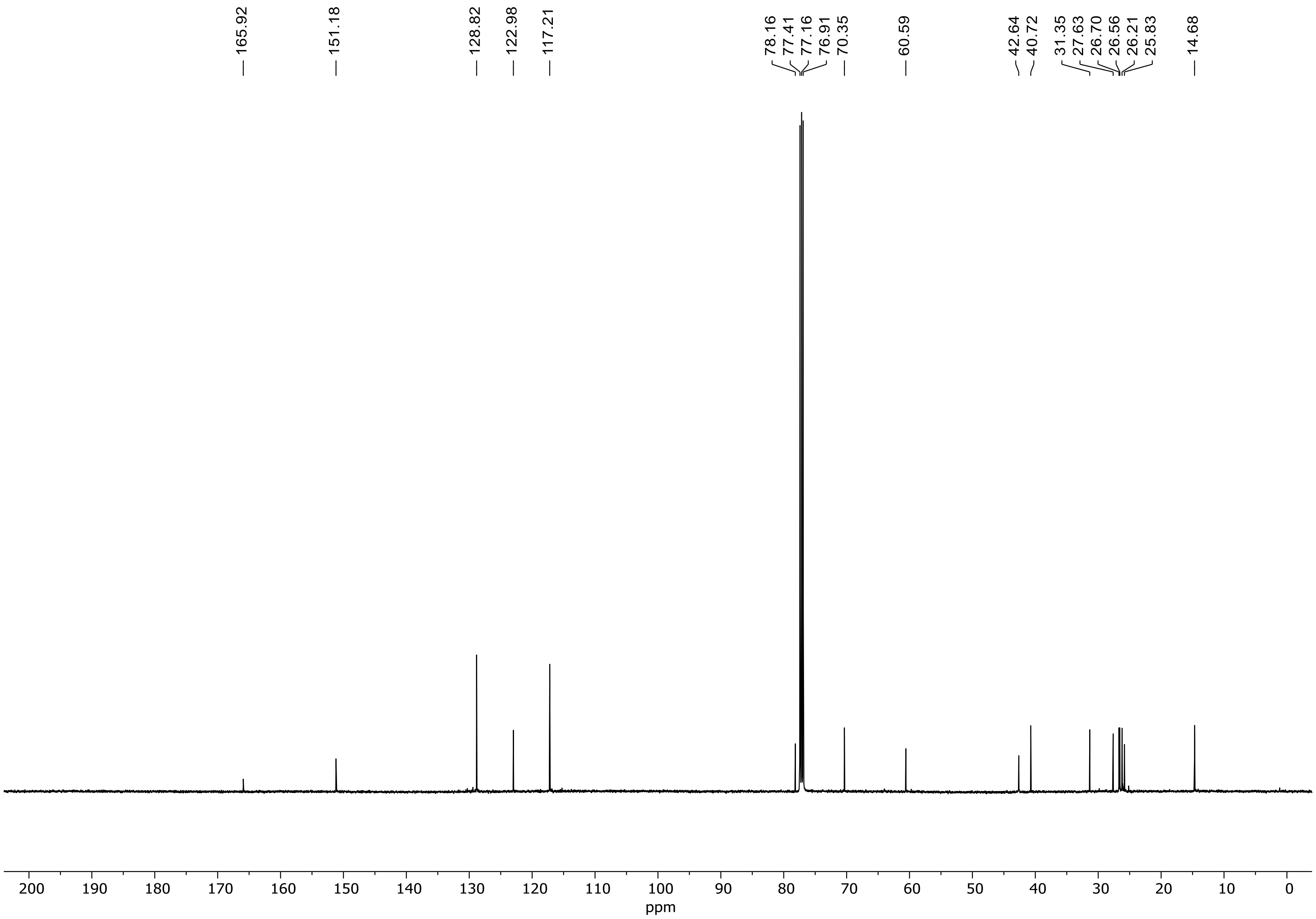

Figure S47: ^{1}H NMR (500 MHz, $CDCl_3$, 298 K) spectrum of **5a.**

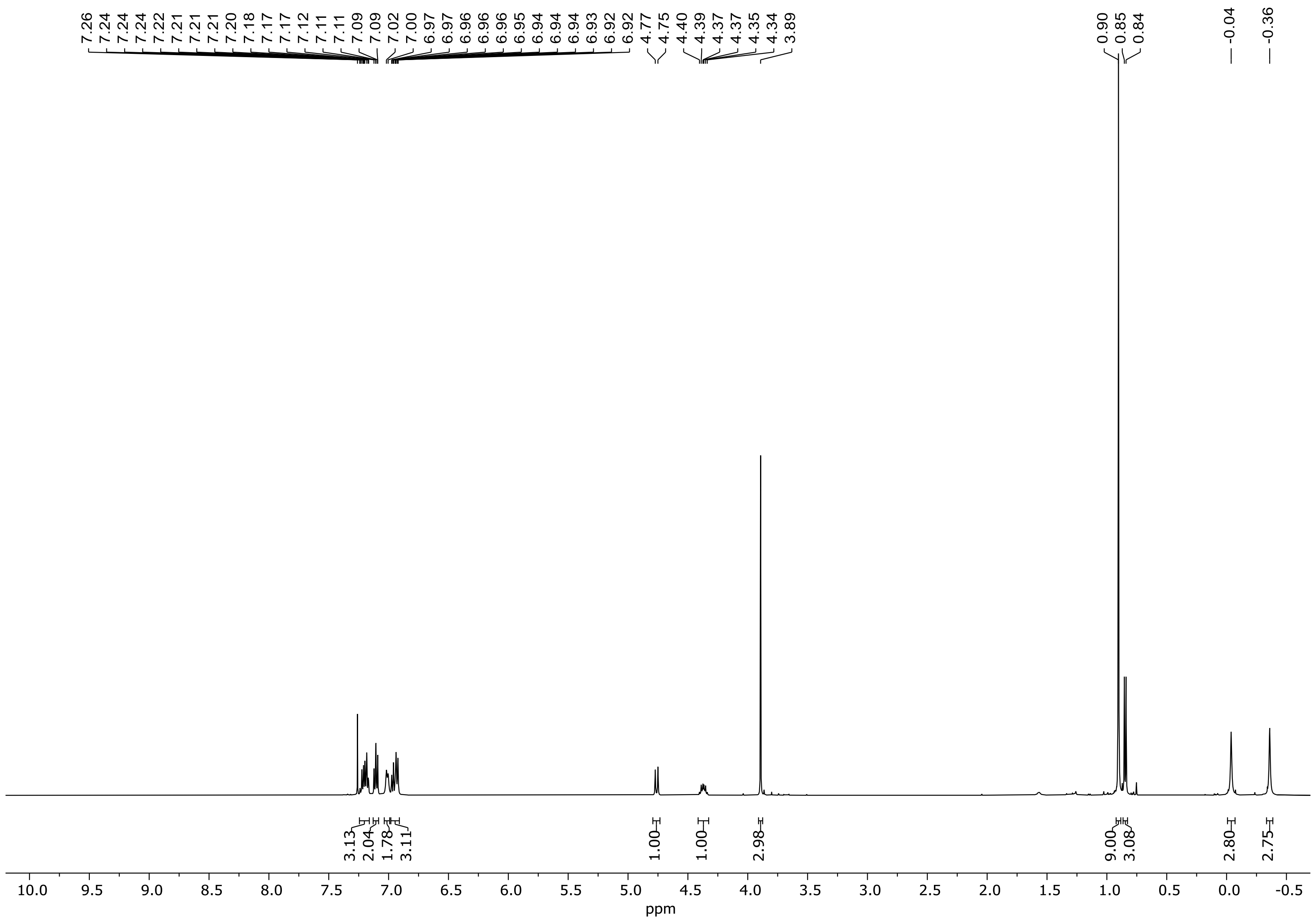

Figure S48: ^{13}C NMR (126 MHz, $CDCl_3$, 298 K) spectrum of **5a**.

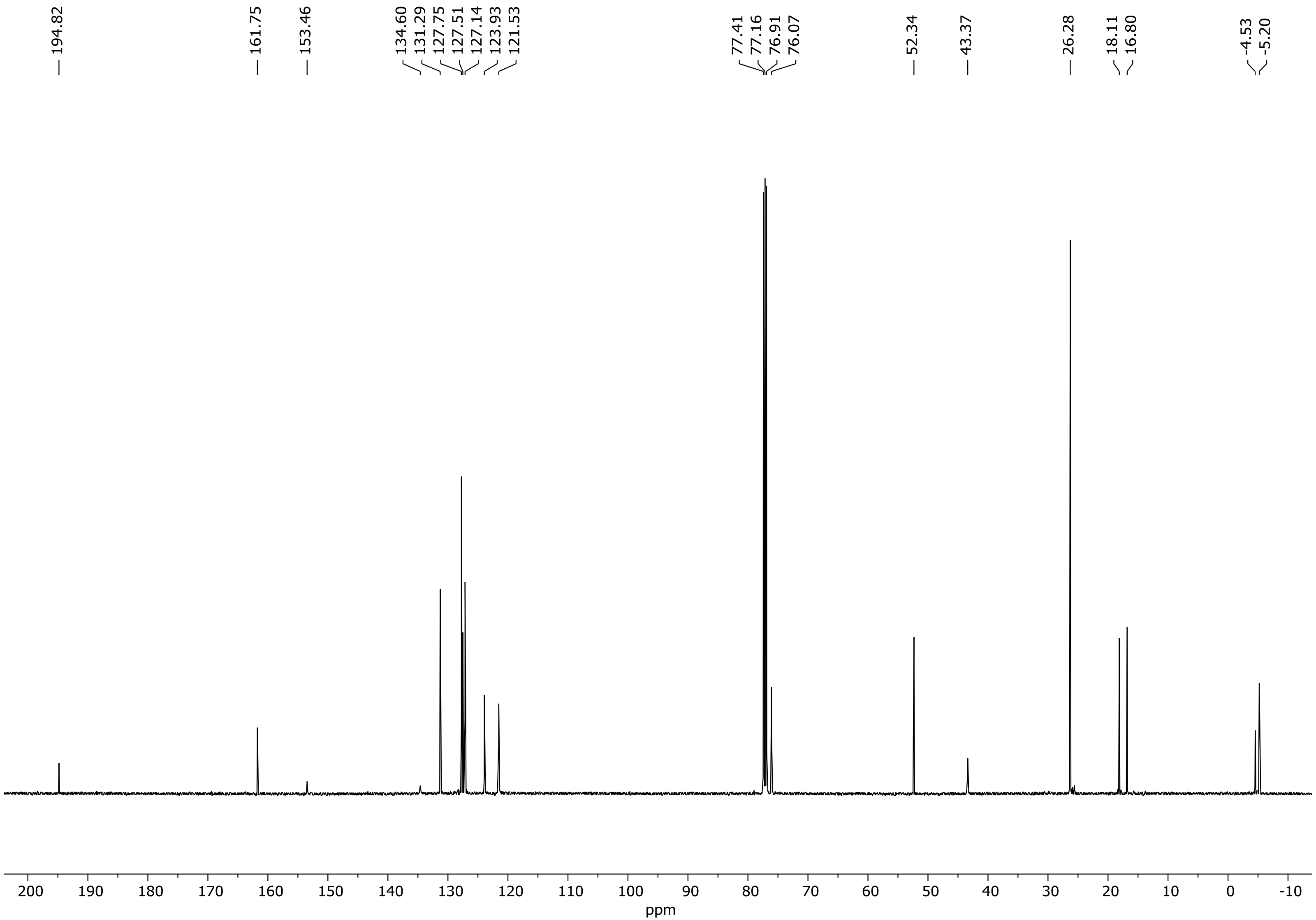

Figure S49: ^{1}H NMR (500 MHz, $CDCl_3$, 298 K) spectrum of **5b.** *NMR shown is that of the minor diastereoisomer.*

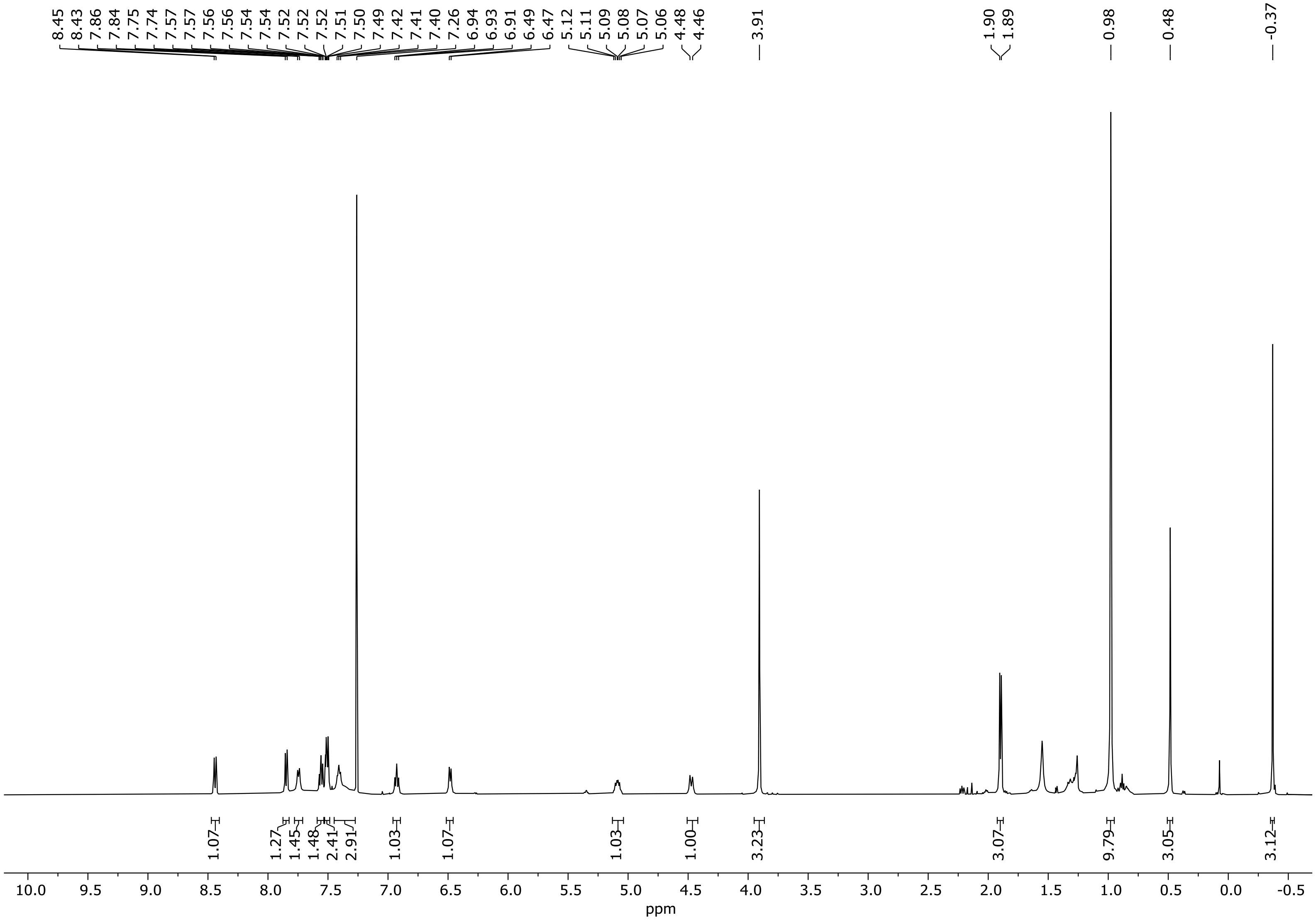

Figure S50: ^{13}C NMR (126 MHz, $CDCl_3$, 298 K) spectrum of **5b**.

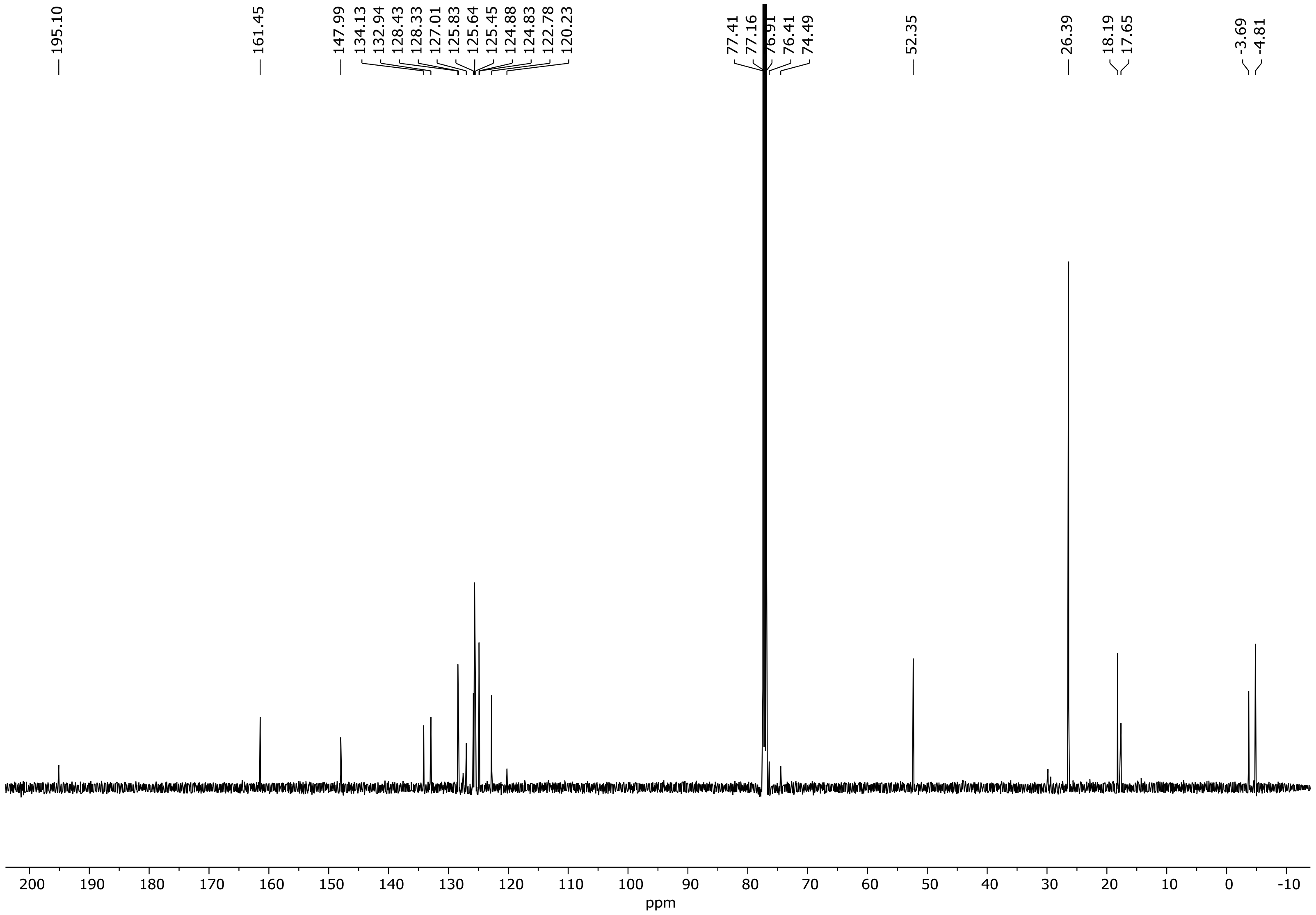

Figure S51: ^{1}H NMR (500 MHz, $CDCl_3$, 298 K) spectrum of **5c.**

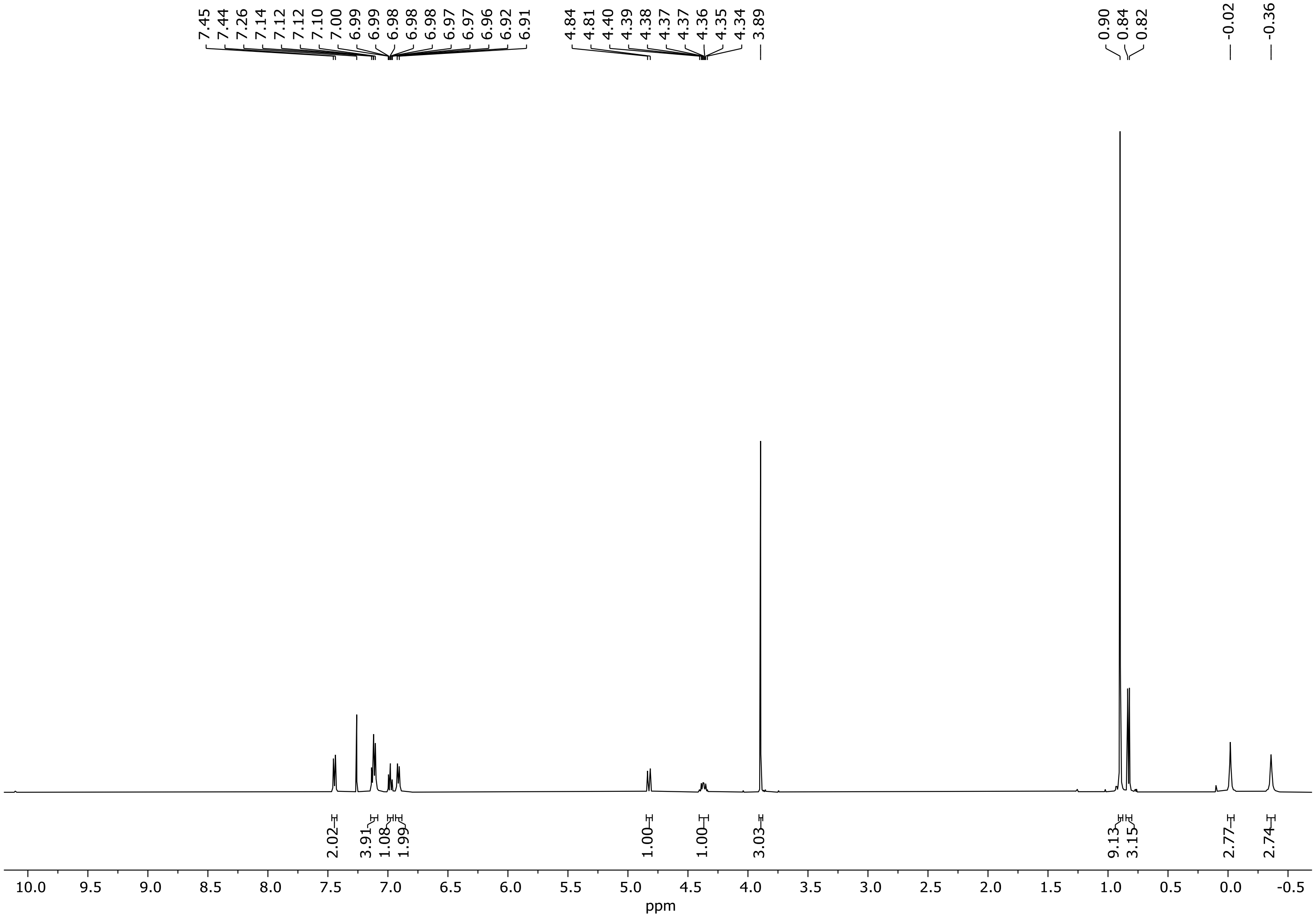

Figure S52: ^{13}C NMR (126 MHz, $CDCl_3$, 298 K) spectrum of **5c**.

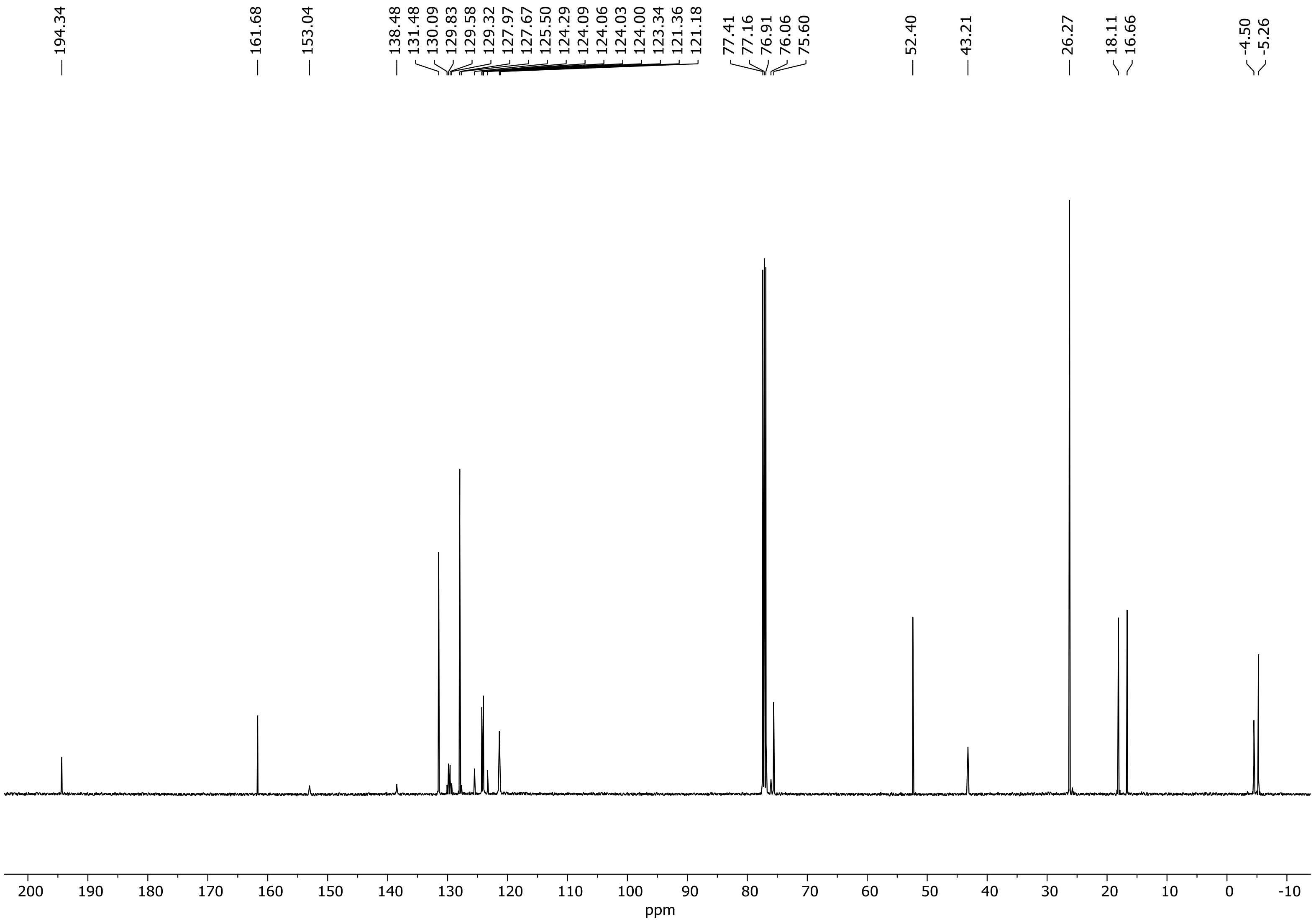

Figure S53: ^{19}F NMR (376 MHz, $CDCl_3$, 298 K) spectrum of **5c**.

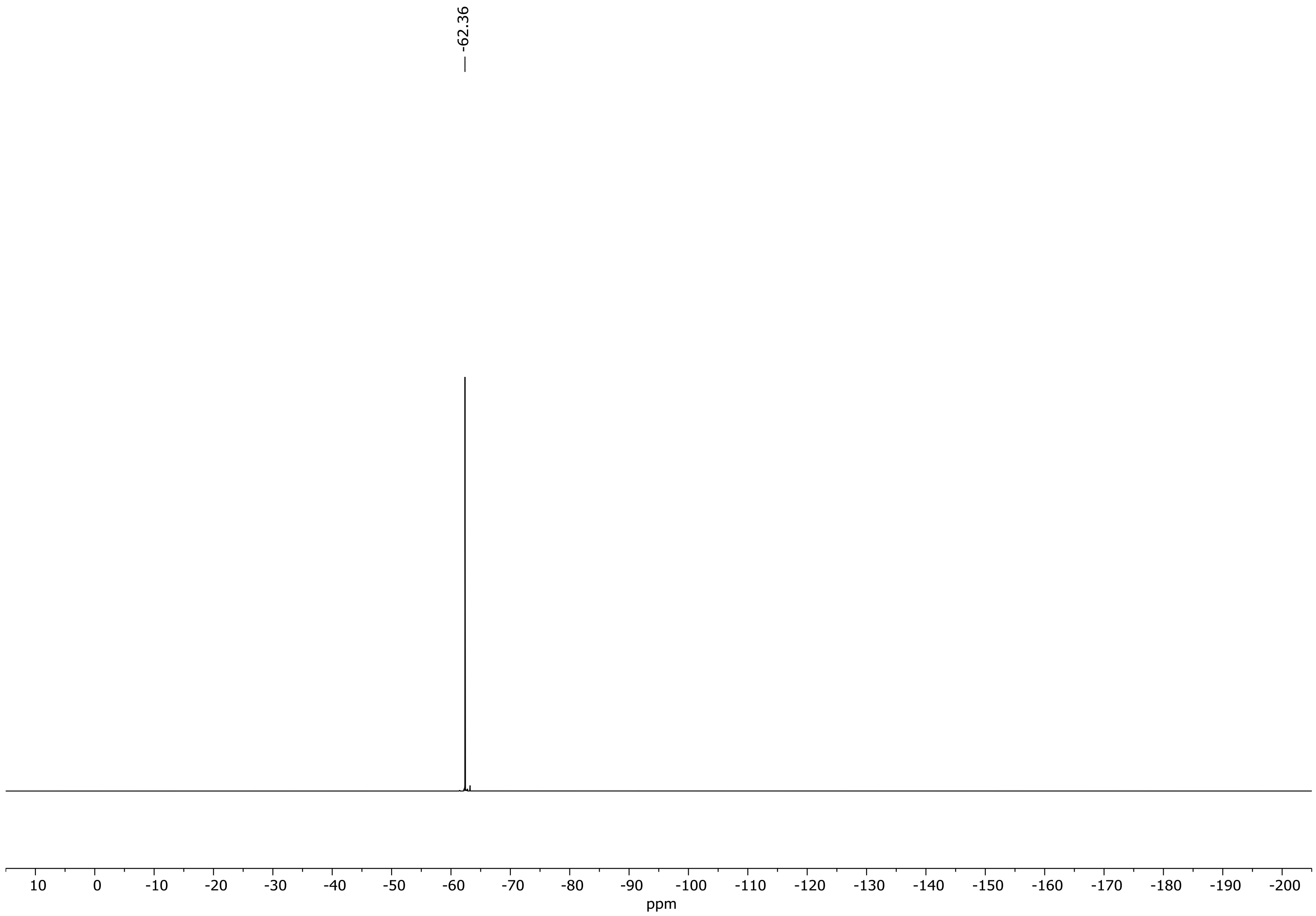

Figure S54: ^{1}H NMR (500 MHz, $CDCl_3$, 298 K) spectrum of *anti*-**5d.**

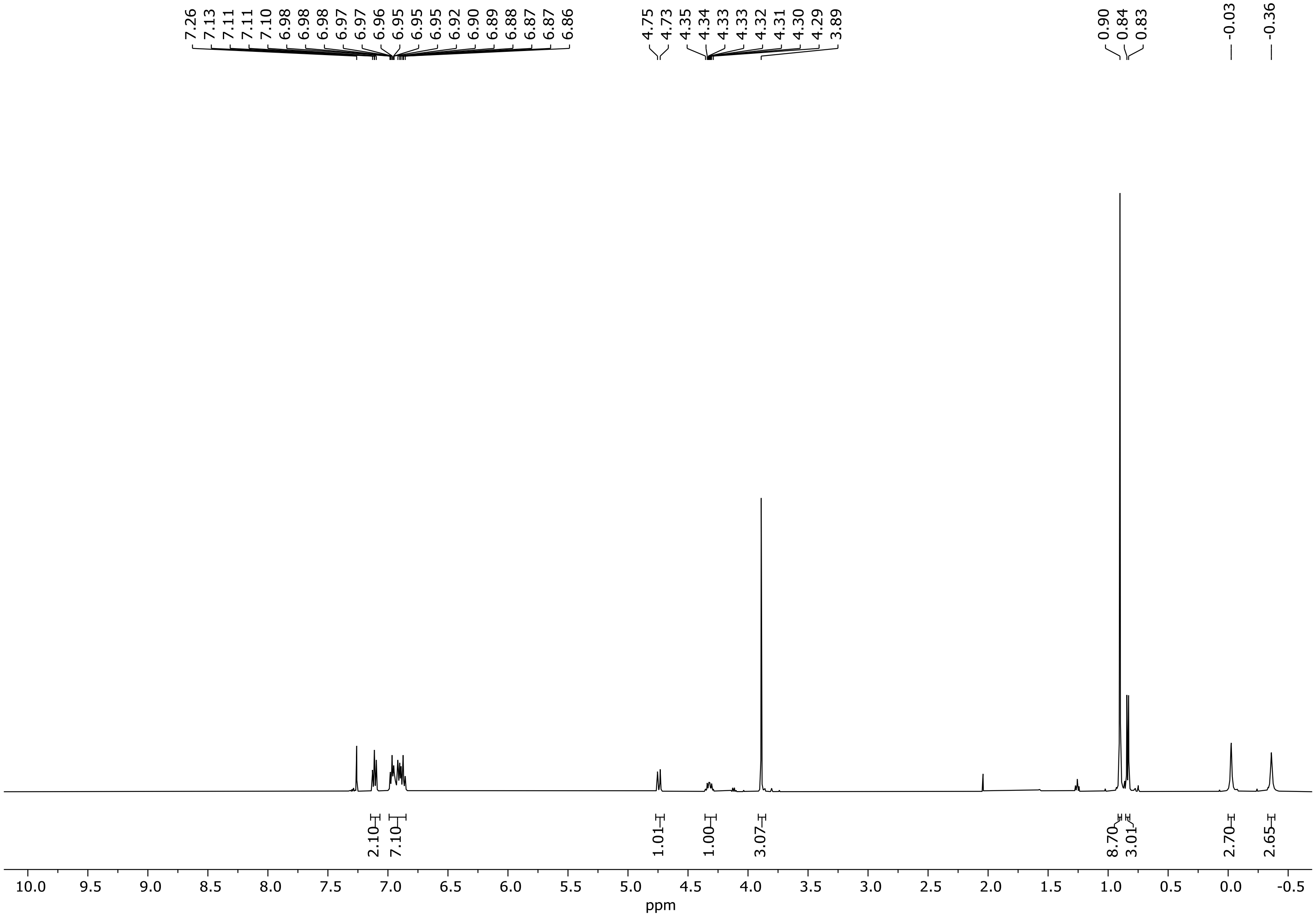

Figure S55: ^{13}C NMR (126 MHz, $CDCl_3$, 298 K) spectrum of *anti*-**5d**.

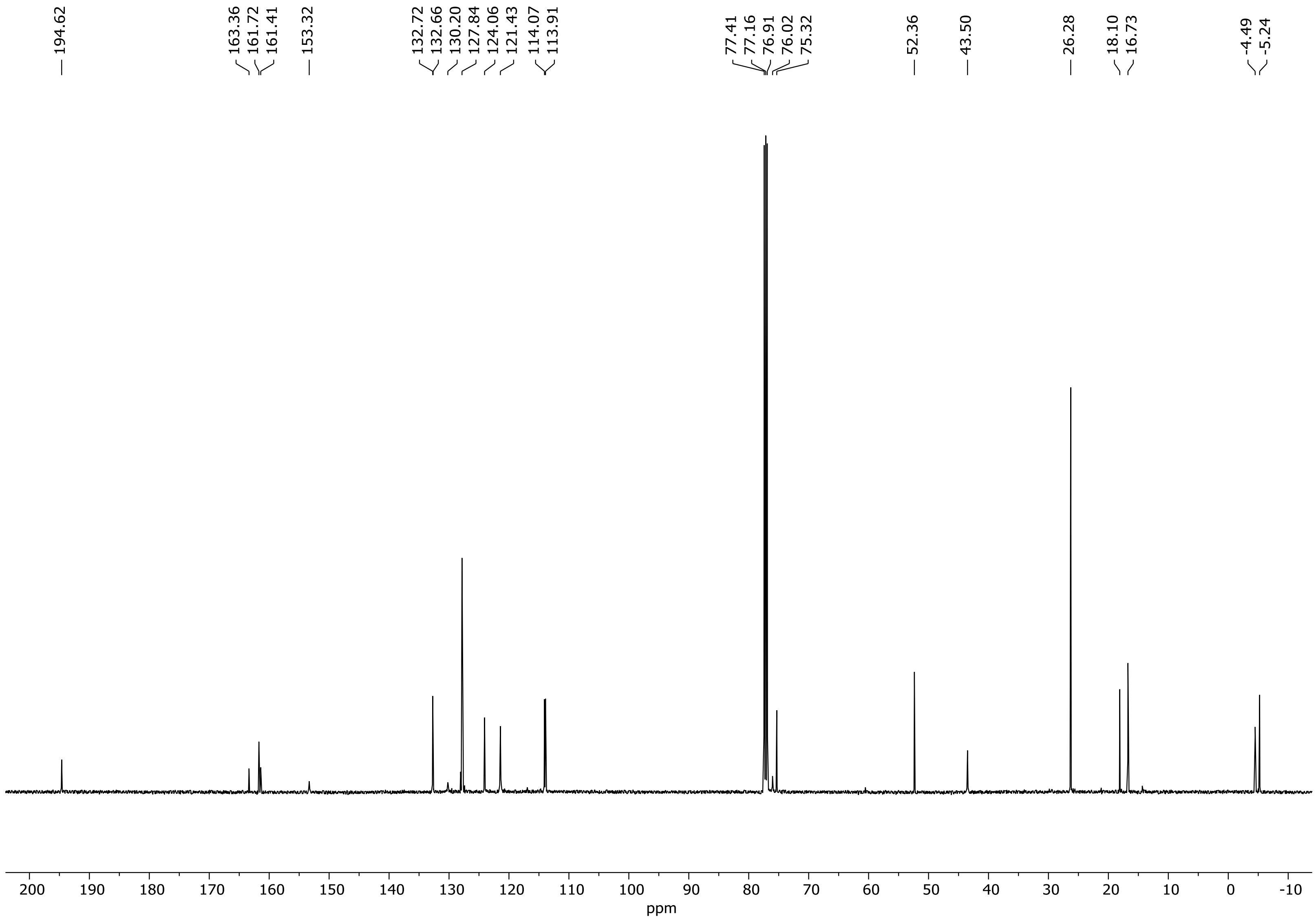

Figure S56: ^{19}F NMR (376 MHz, $CDCl_3$, 298 K) spectrum of *anti*-**5d**.

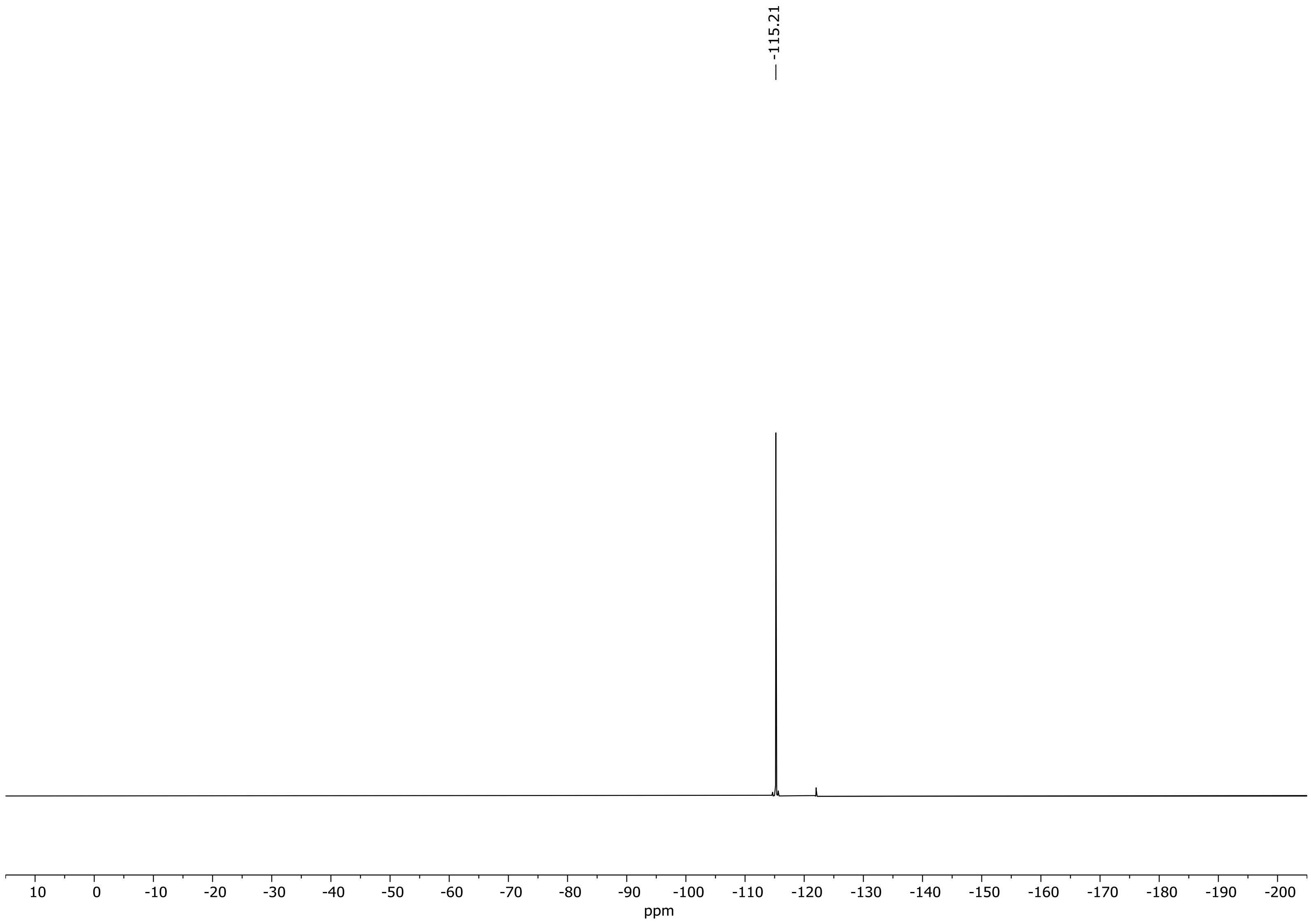

Figure S57: ^{1}H NMR (500 MHz, $CDCl_3$, 298 K) spectrum of *syn*-**5d.**

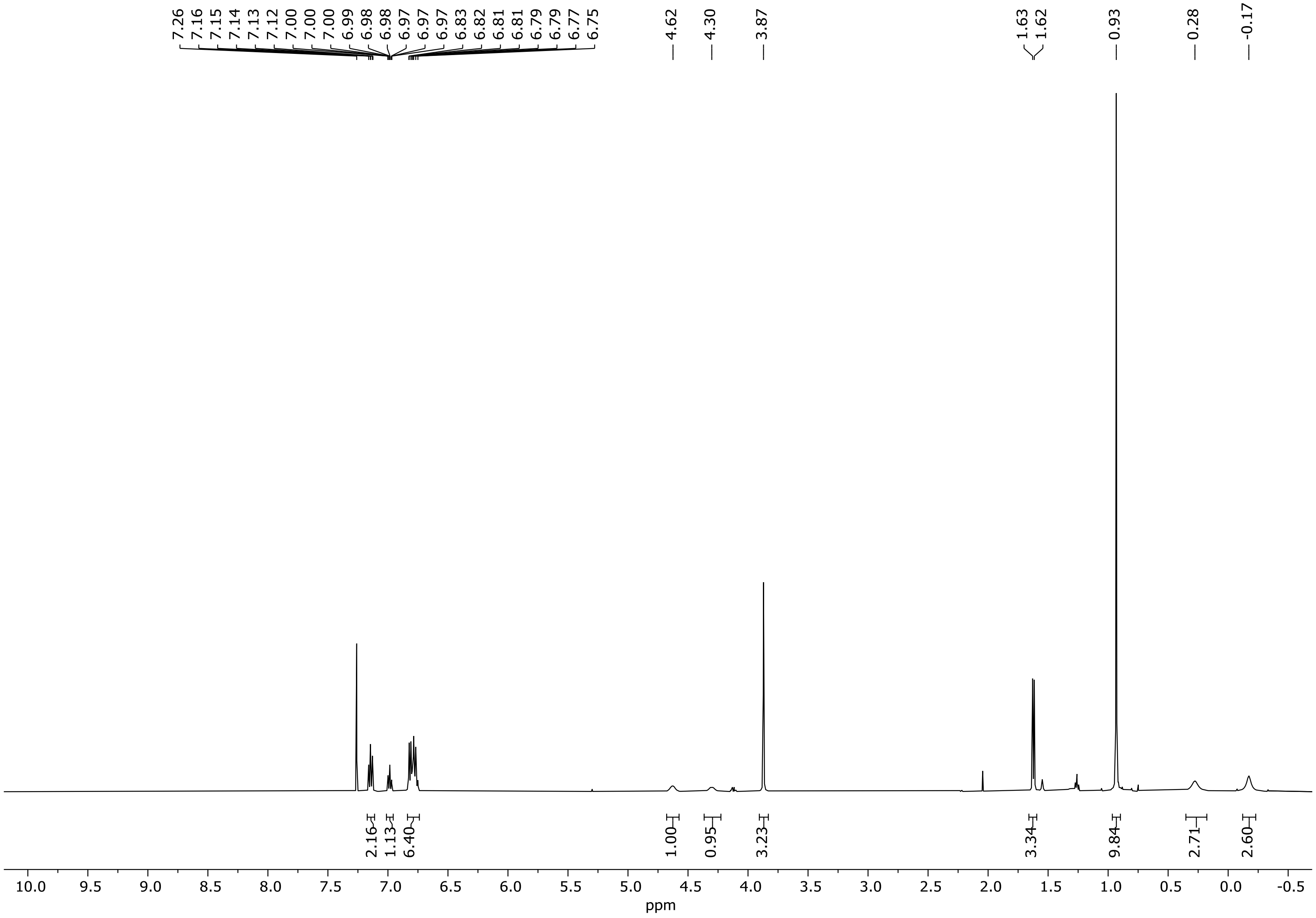

Figure S58: ^{13}C NMR (126 MHz, $CDCl_3$, 298 K) spectrum of *syn*-**5d**.

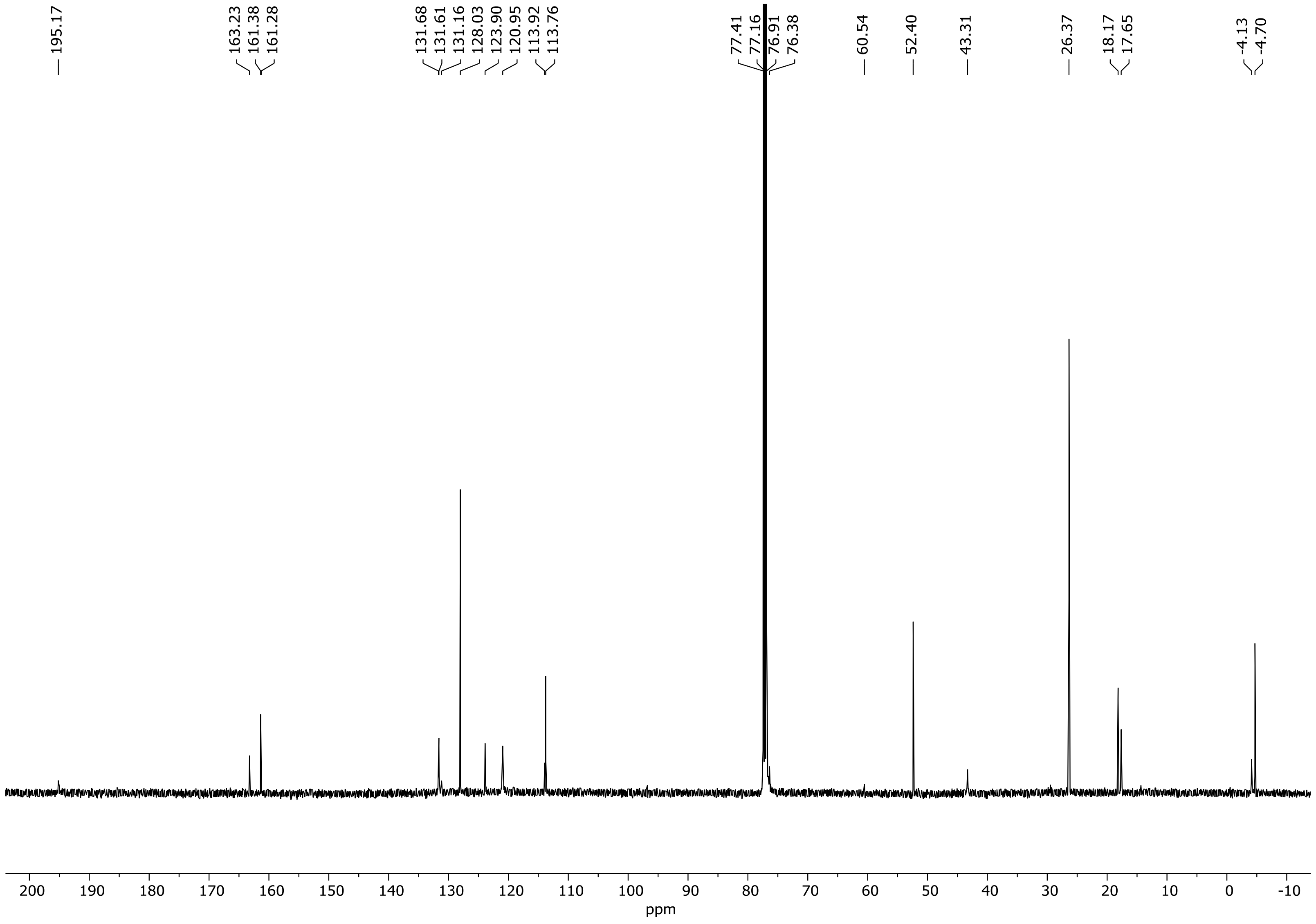

Figure S59: ^{19}F NMR (376 MHz, $CDCl_3$, 298 K) spectrum of *syn*-**5d**.

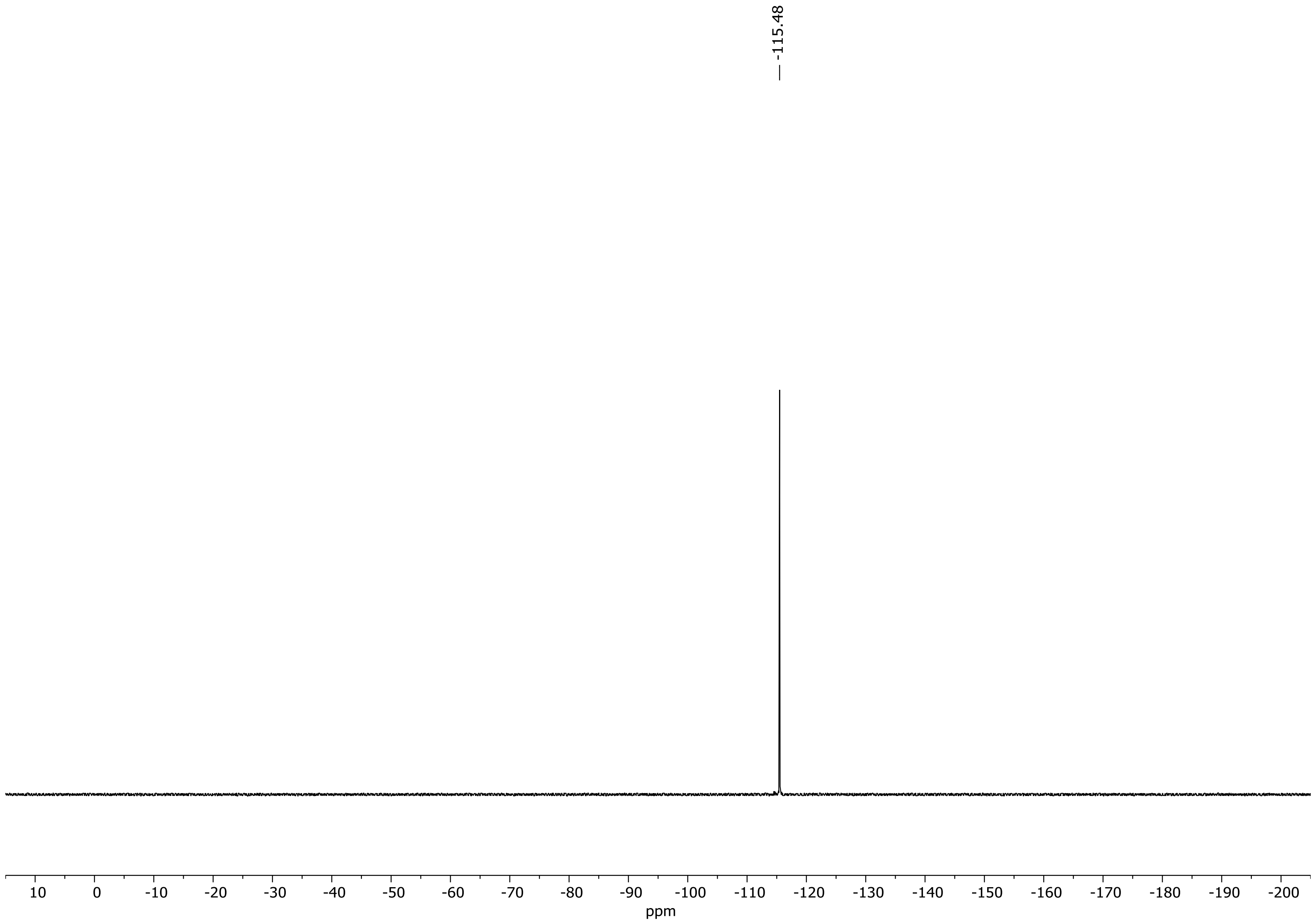

Figure S60: ^{1}H NMR (500 MHz, $CDCl_3$, 298 K) spectrum of **5e.**

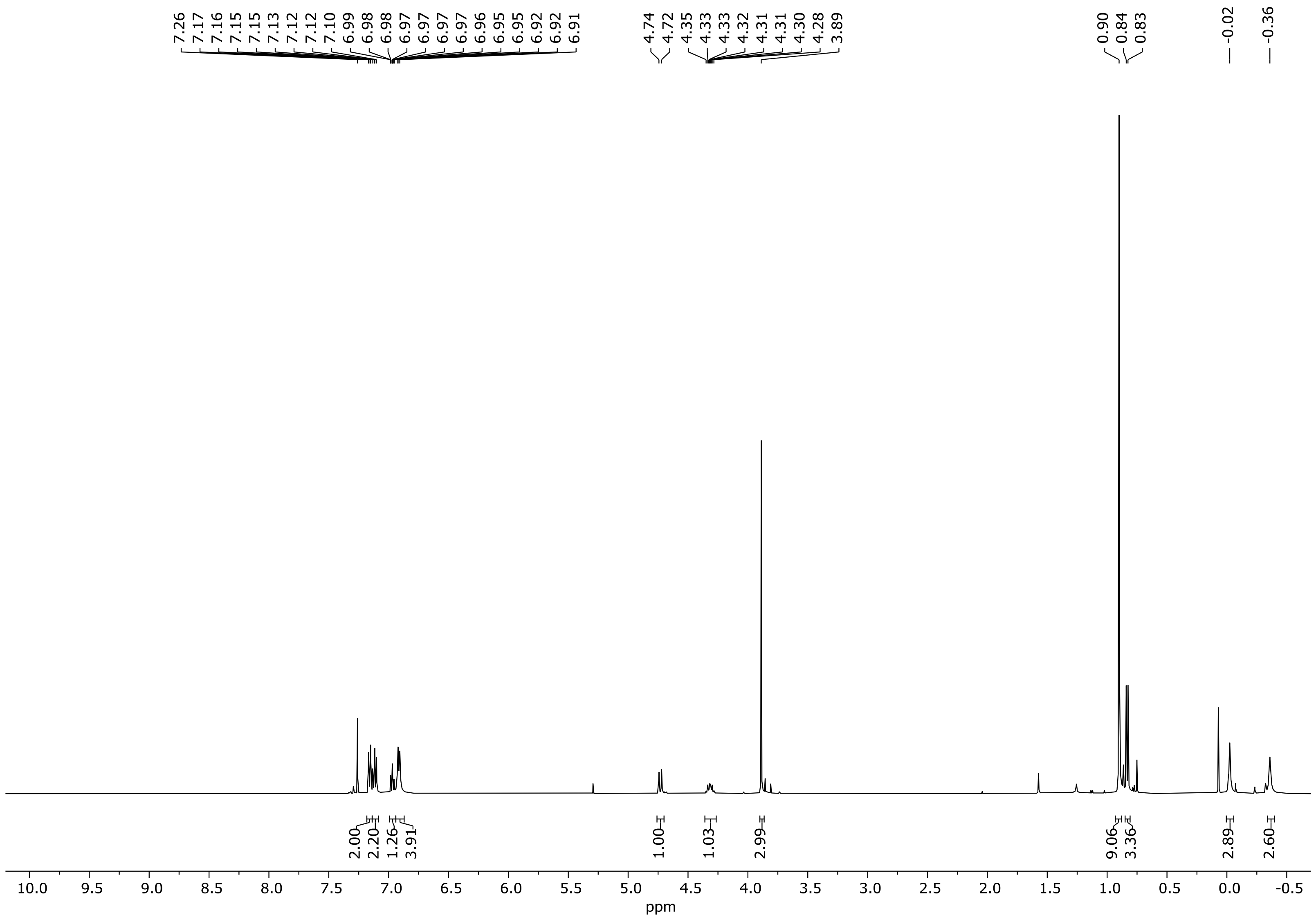

Figure S61: ^{13}C NMR (126 MHz, $CDCl_3$, 298 K) spectrum of **5e**.

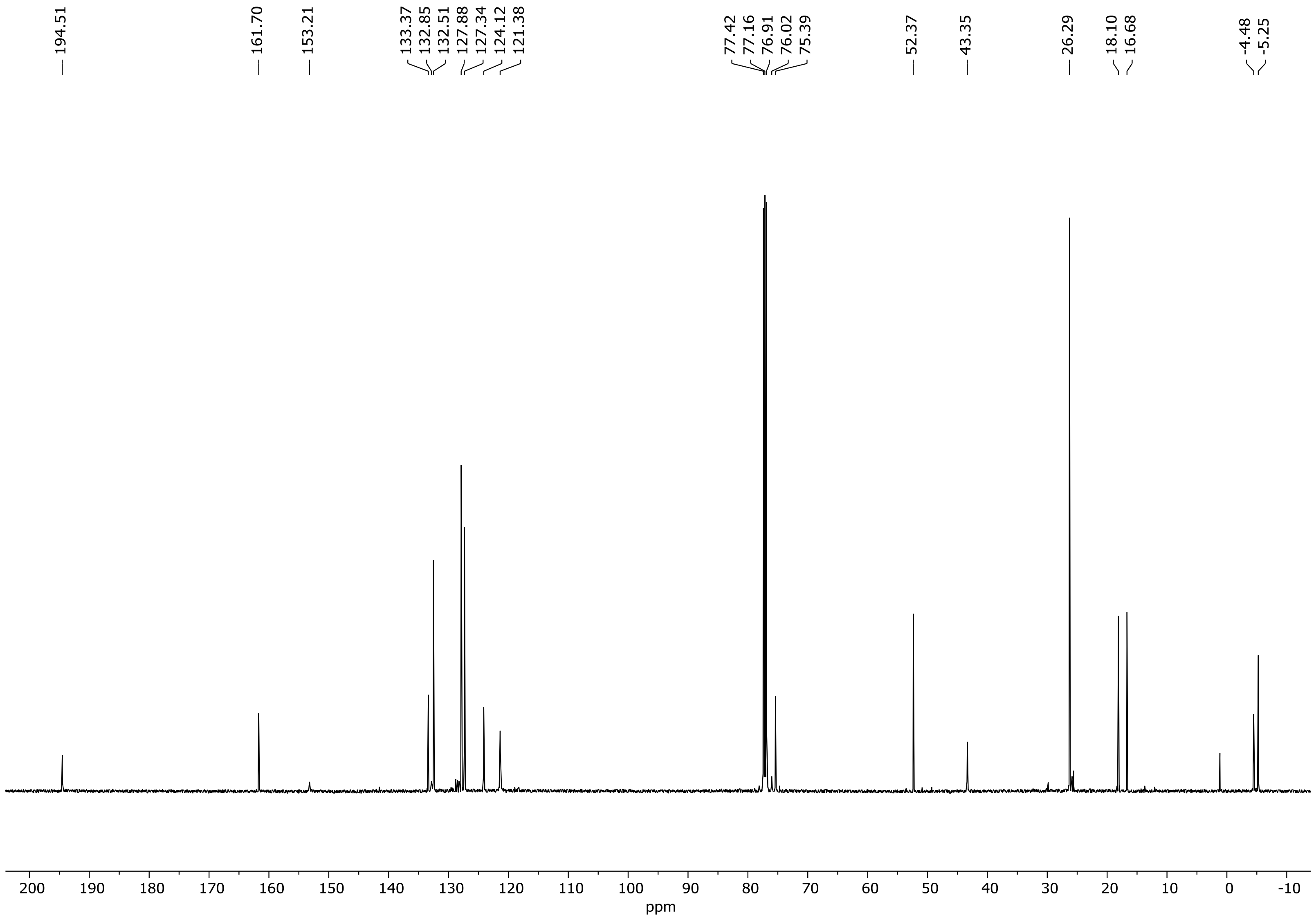

Figure S62: ^{1}H NMR (500 MHz, $CDCl_3$, 298 K) spectrum of **5f.**

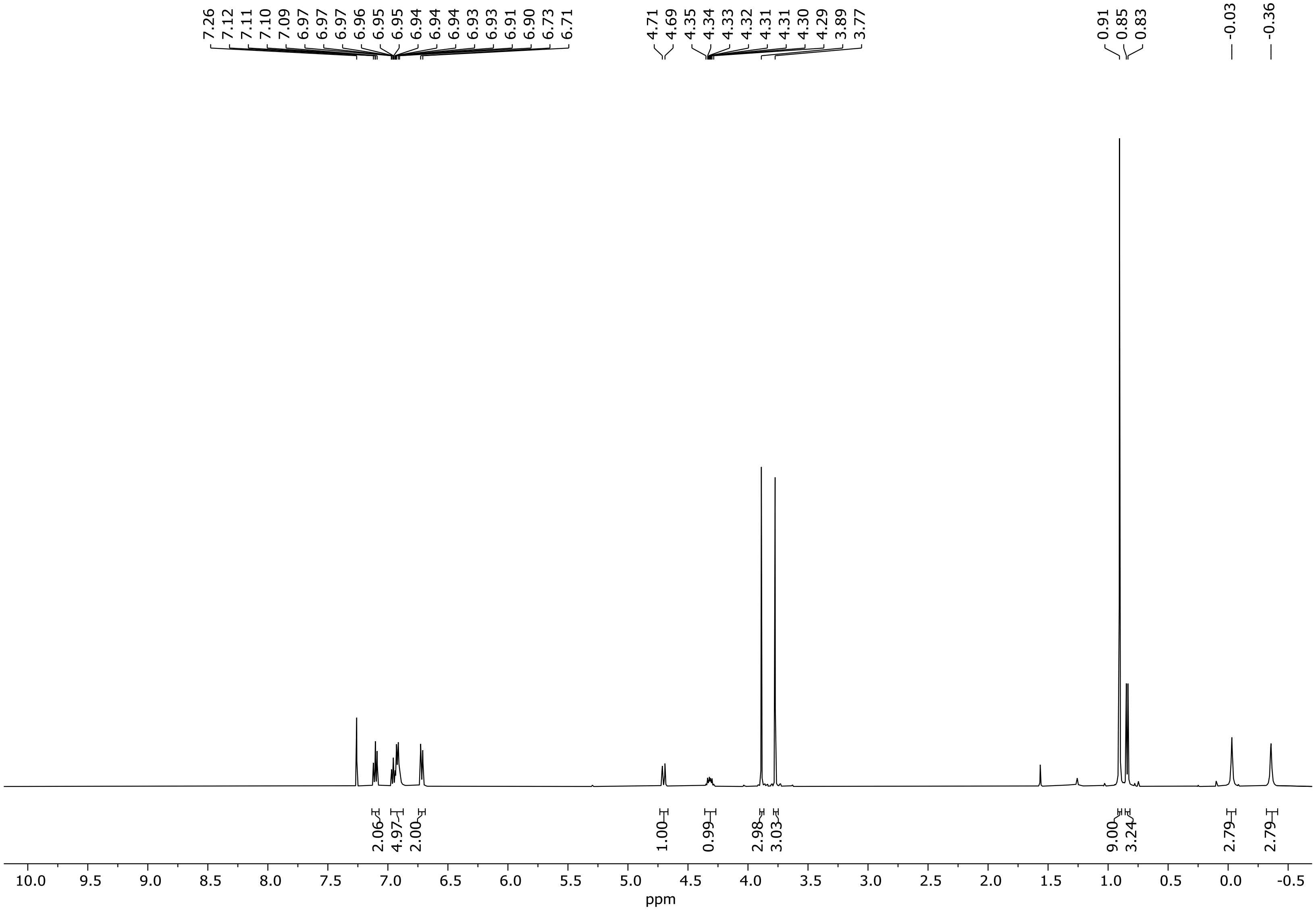

Figure S63: ^{13}C NMR (126 MHz, $CDCl_3$, 298 K) spectrum of **5f**.

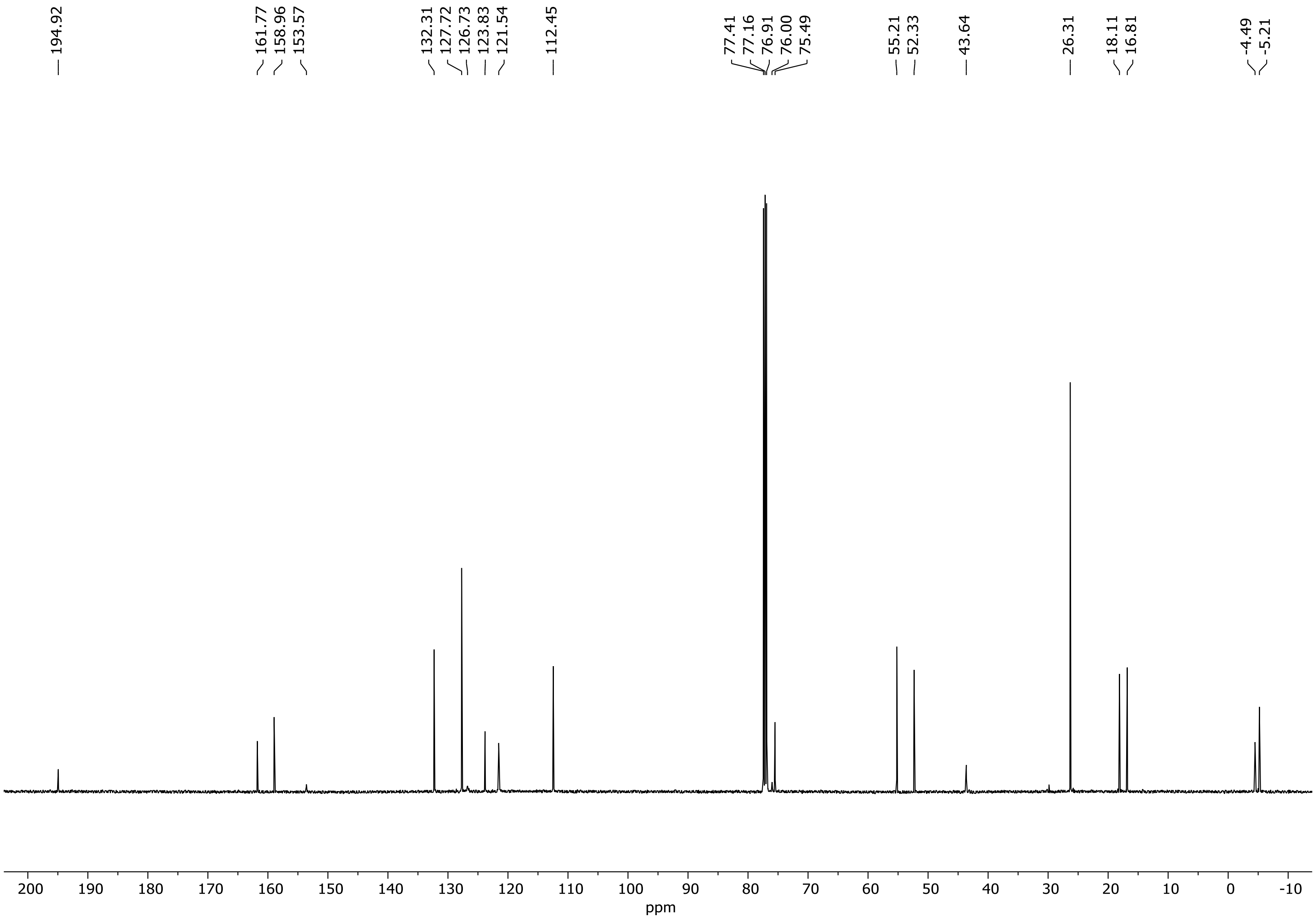

Figure S64: ^{1}H NMR (500 MHz, $CDCl_3$, 298 K) spectrum of **5g.**

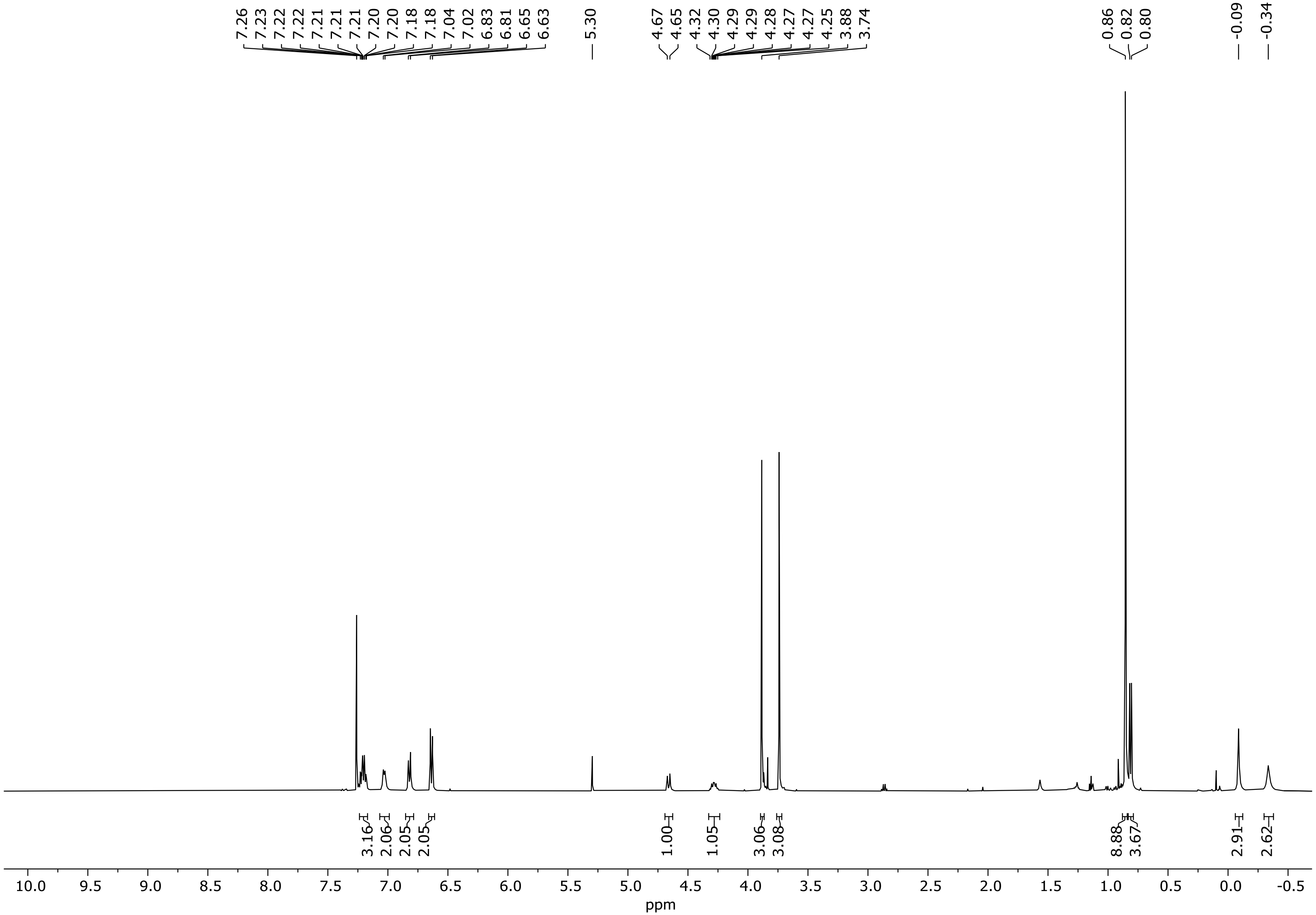

Figure S65: ^{13}C NMR (126 MHz, $CDCl_3$, 298 K) spectrum of **5g**.

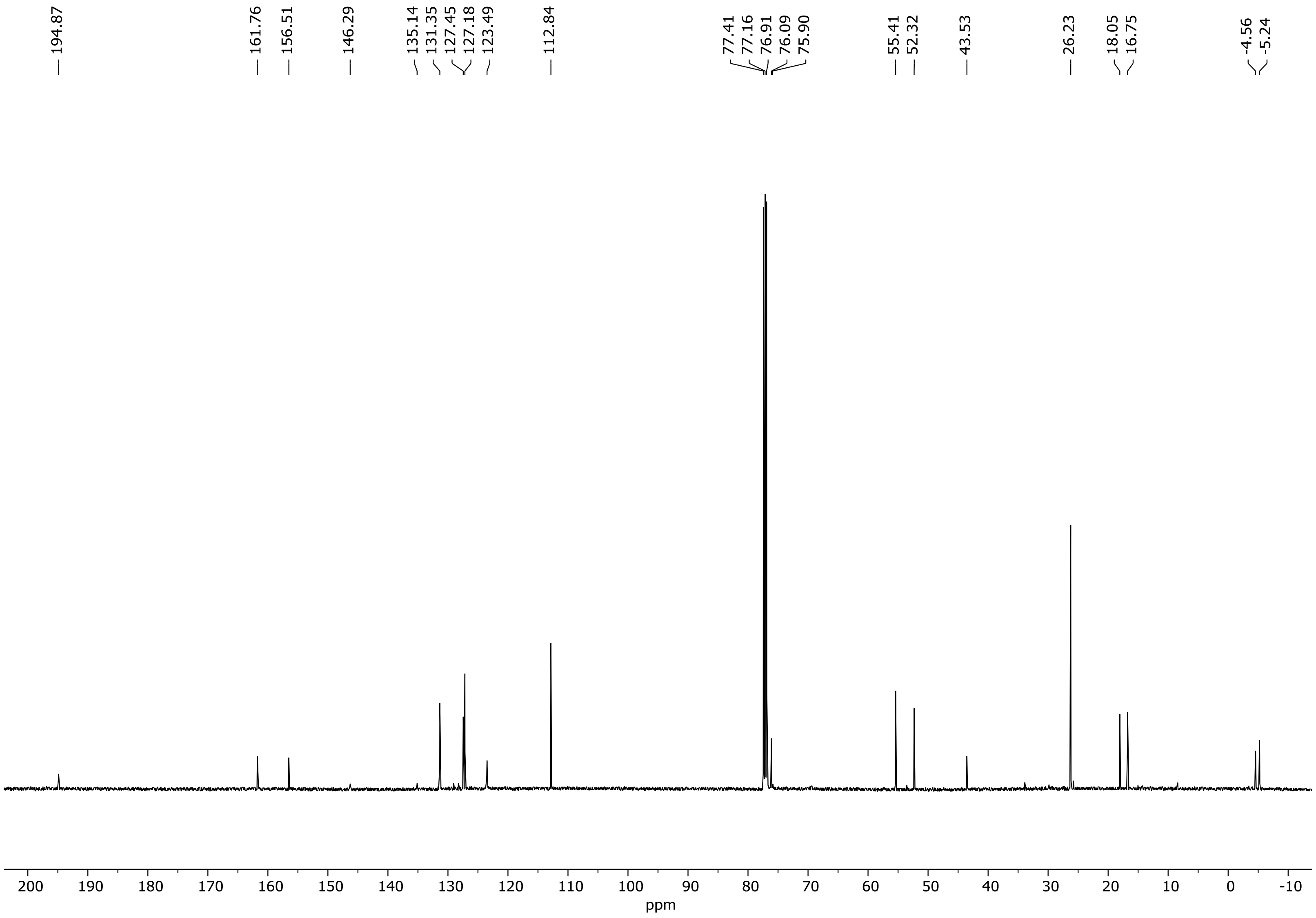

Figure S66: ^{1}H NMR (500 MHz, $CDCl_3$, 298 K) spectrum of **5h.**

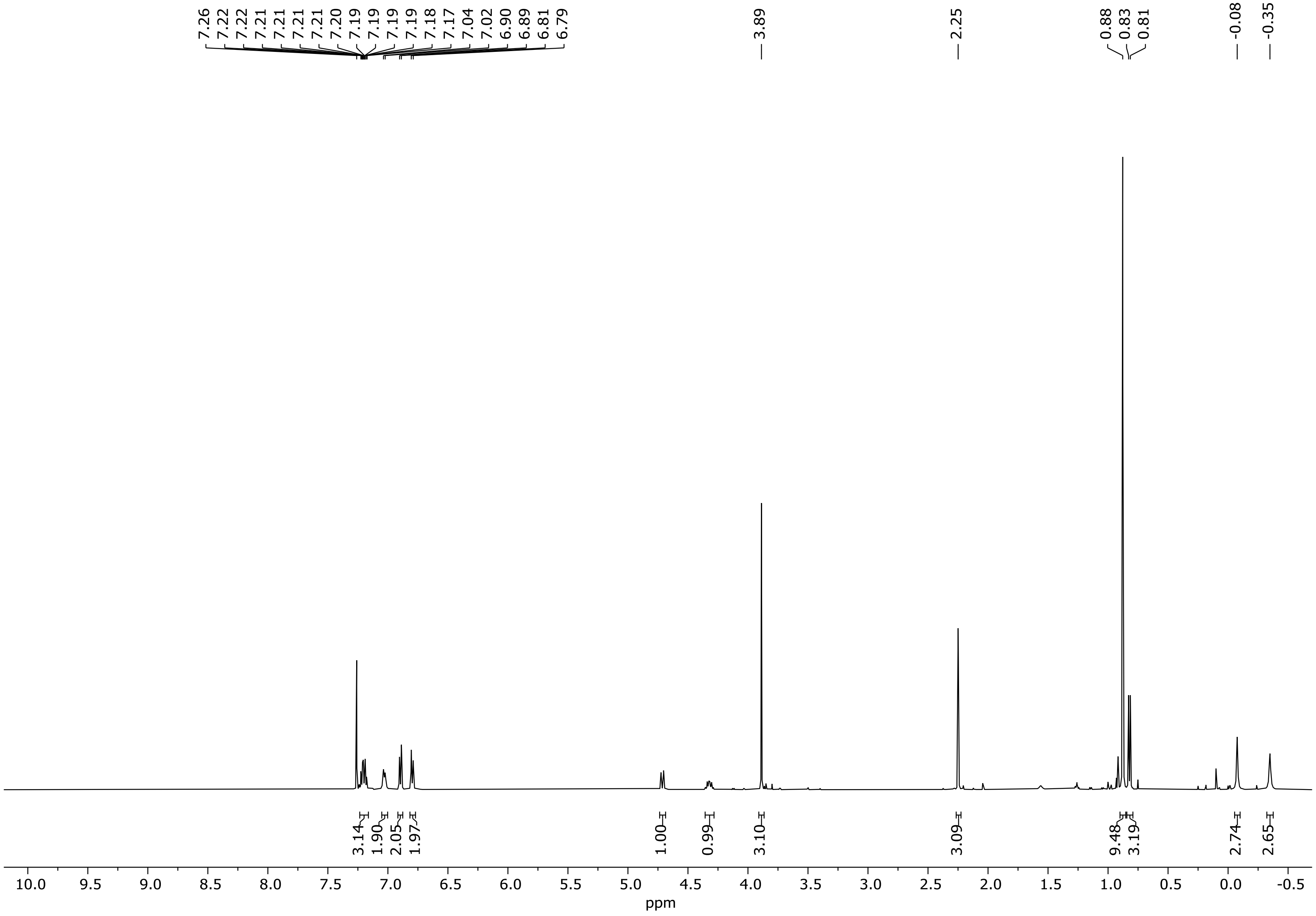

Figure S67: ^{13}C NMR (126 MHz, $CDCl_3$, 298 K) spectrum of **5h**.

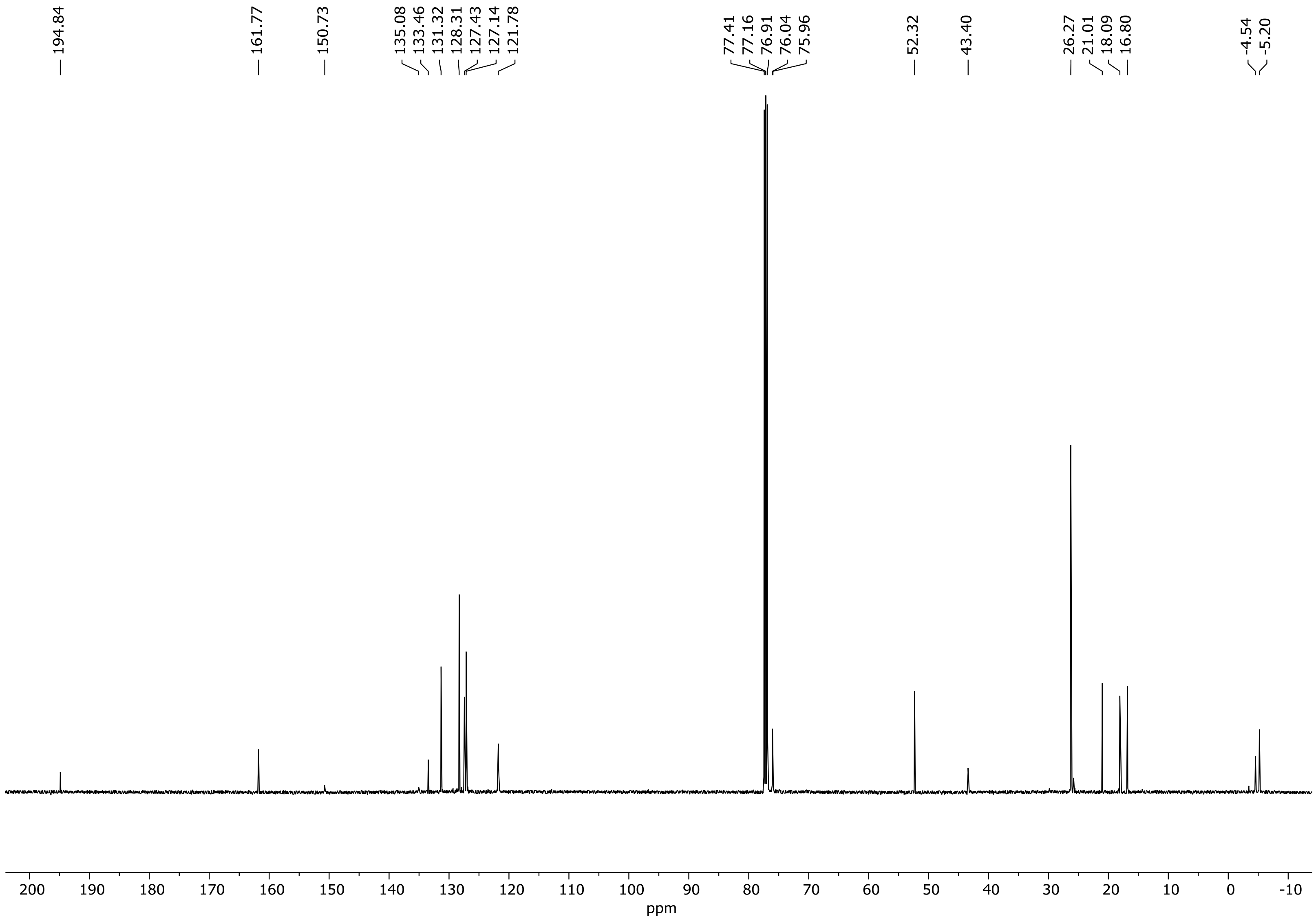

Figure S68: ^{1}H NMR (500 MHz, $CDCl_3$, 298 K) spectrum of **5i.**

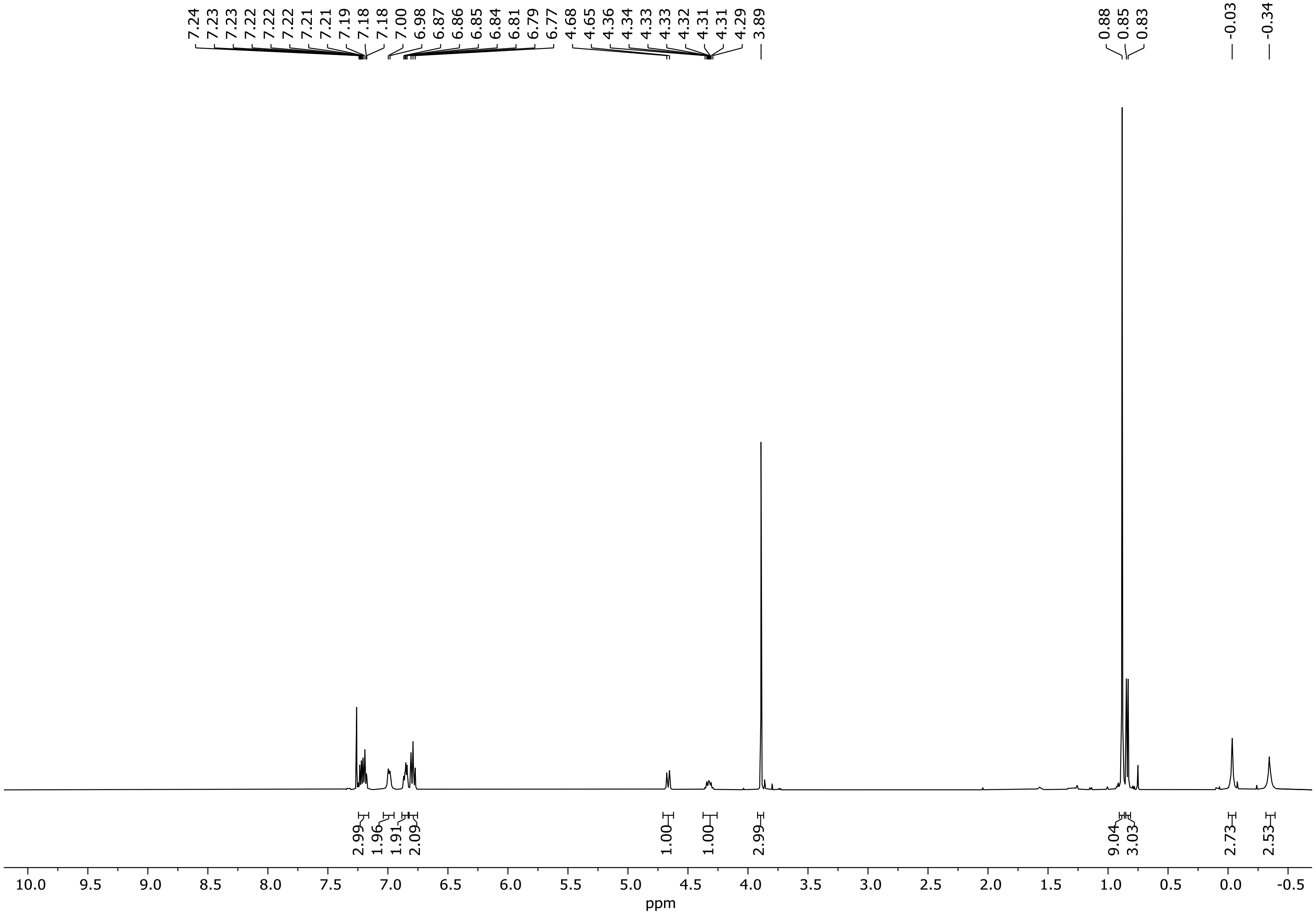

Figure S69: ^{13}C NMR (126 MHz, $CDCl_3$, 298 K) spectrum of **5i**.

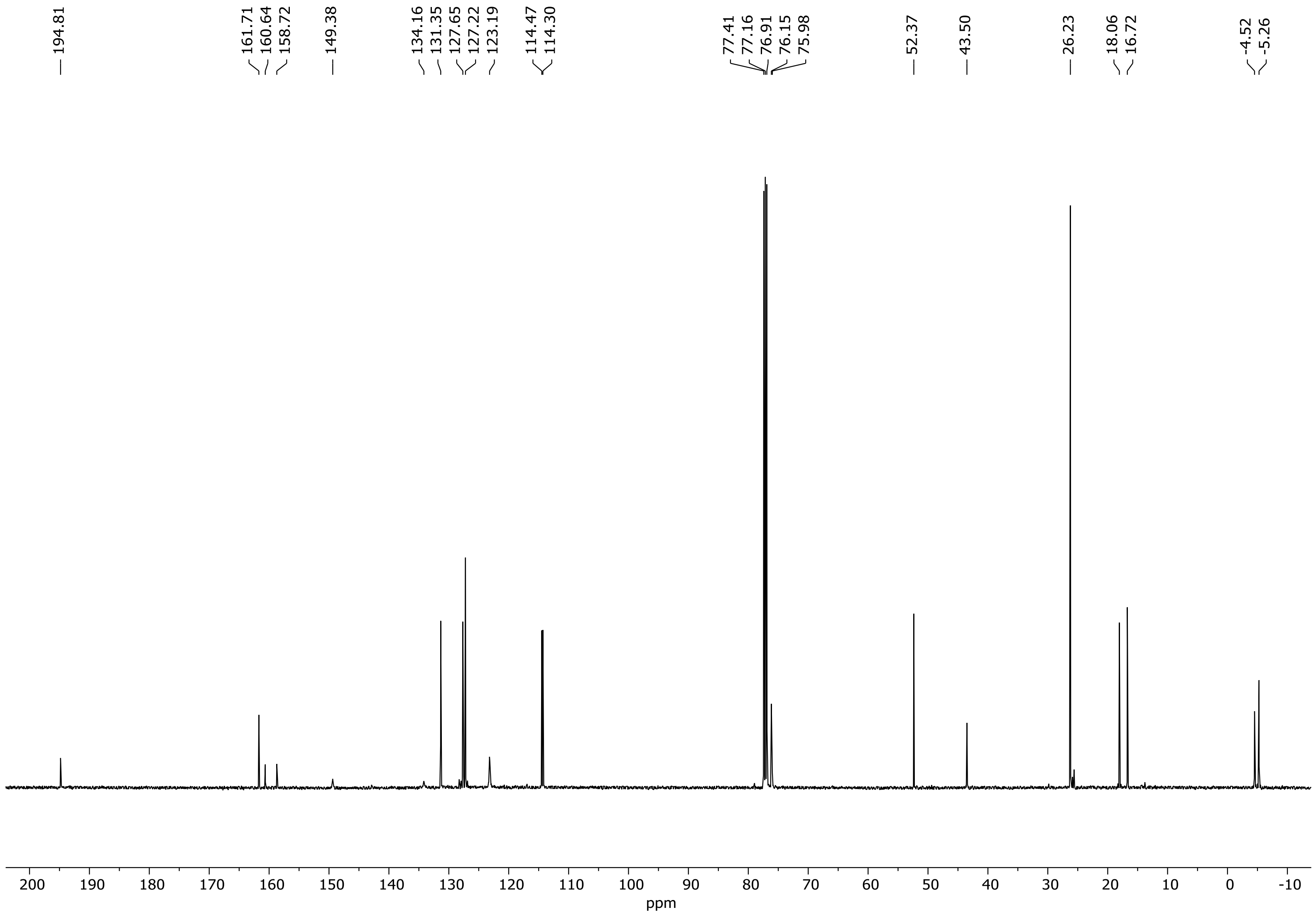

Figure S70: ^{19}F NMR (376 MHz, $CDCl_3$, 298 K) spectrum of **5i**.

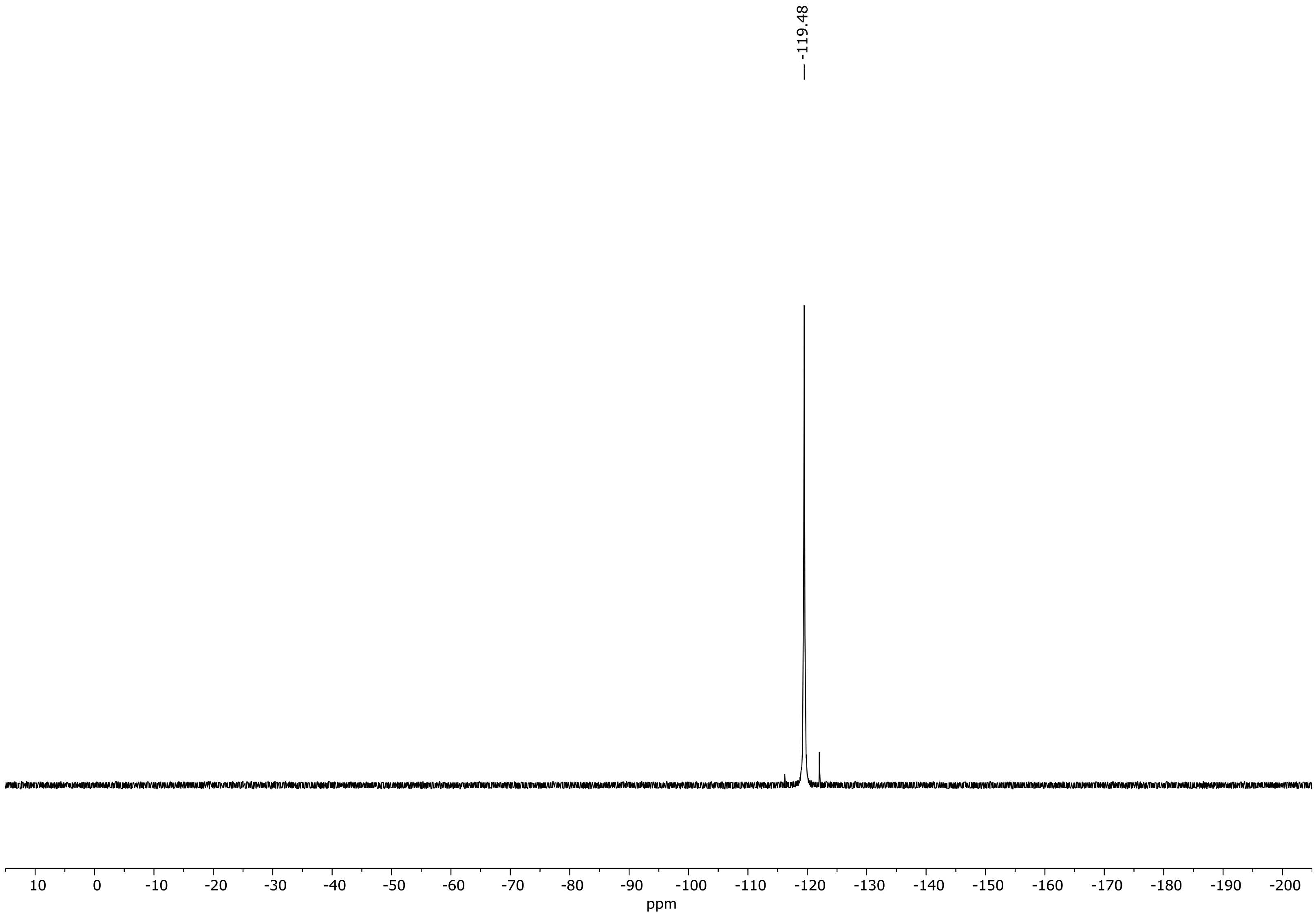

Figure S71: ^{1}H NMR (500 MHz, $CDCl_3$, 298 K) spectrum of **5j.**

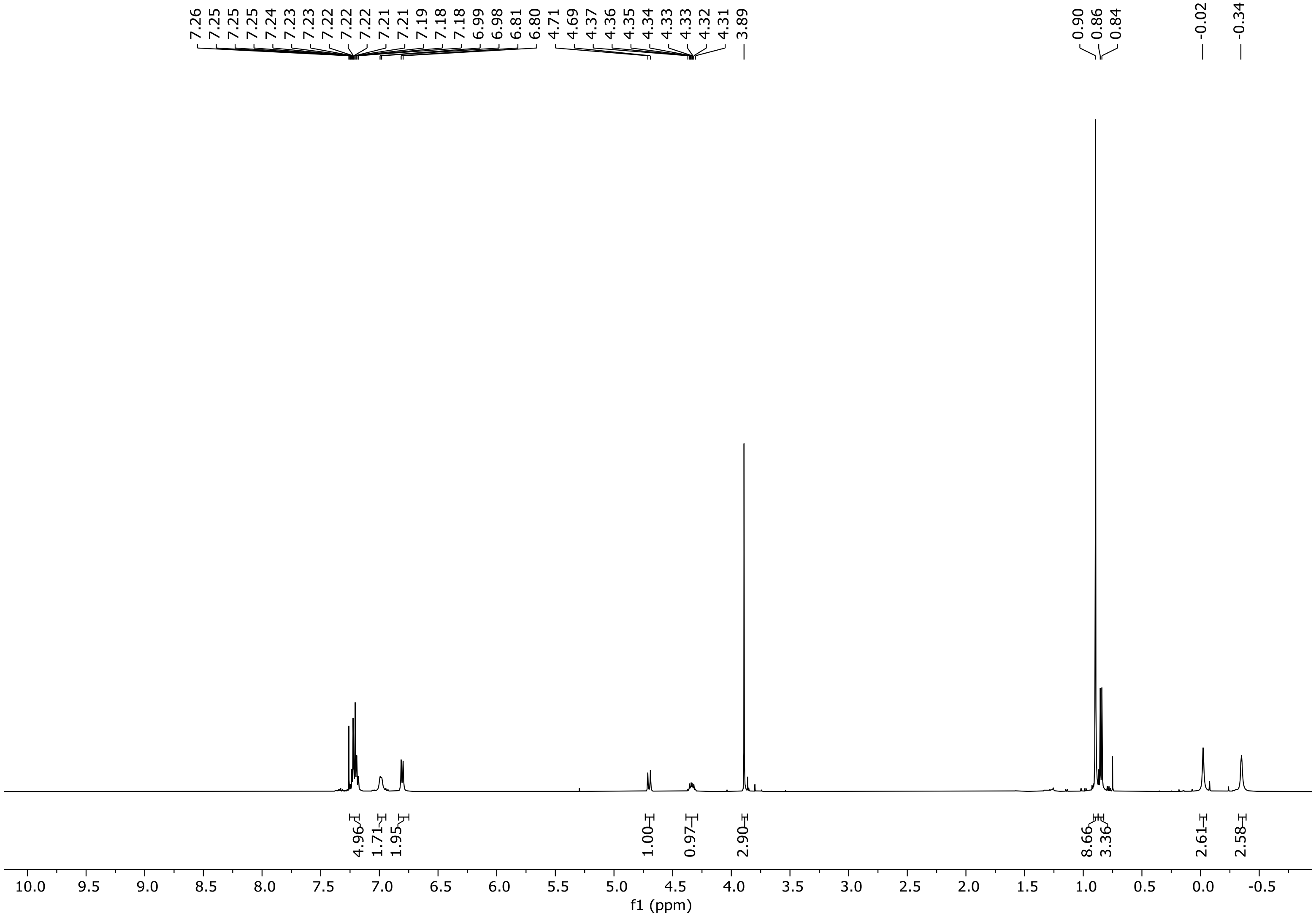

Figure S72: ^{13}C NMR (126 MHz, $CDCl_3$, 298 K) spectrum of **5j**.

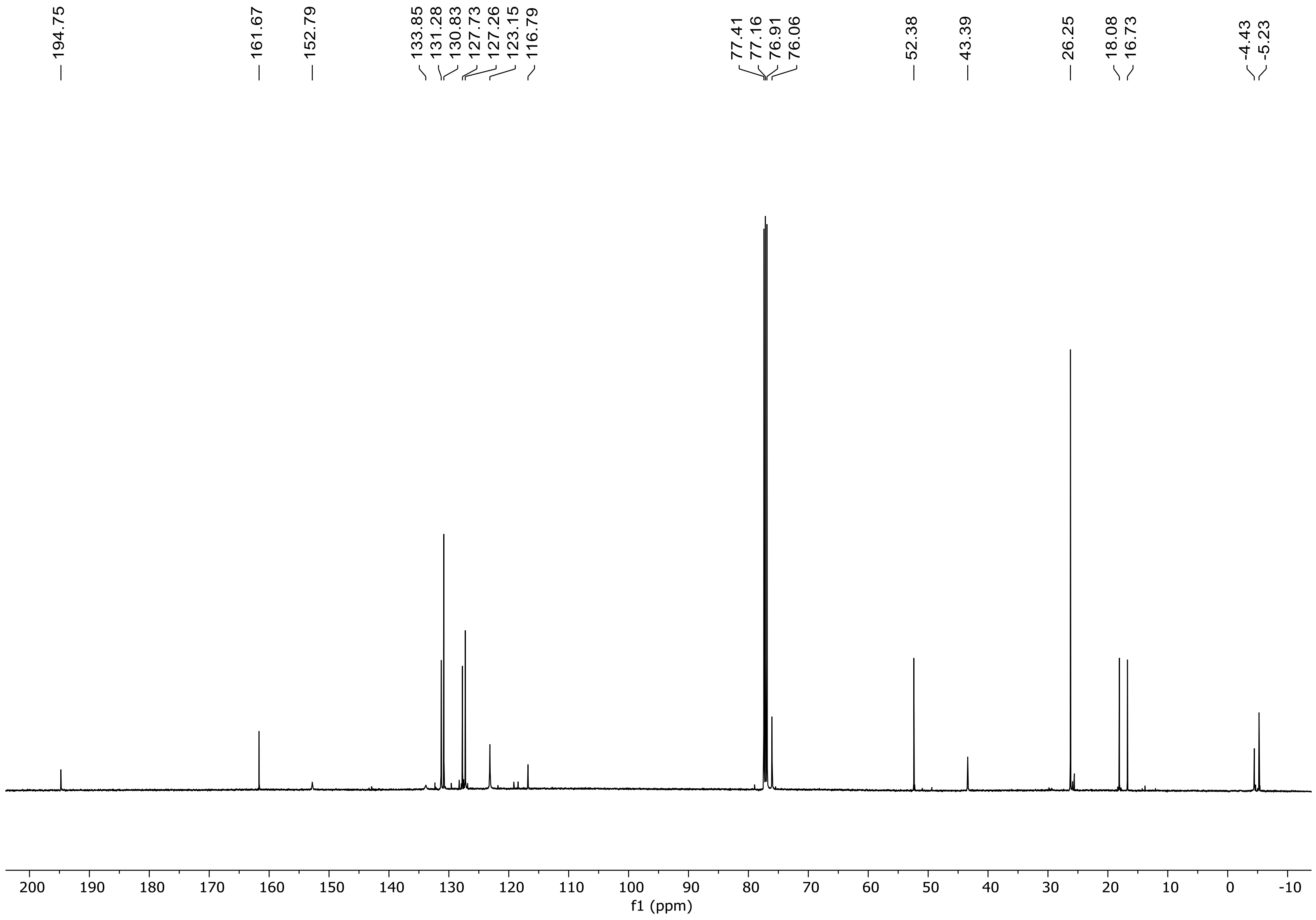

Figure S73: ^{1}H NMR (500 MHz, $CDCl_3$, 298 K) spectrum of **5k.**

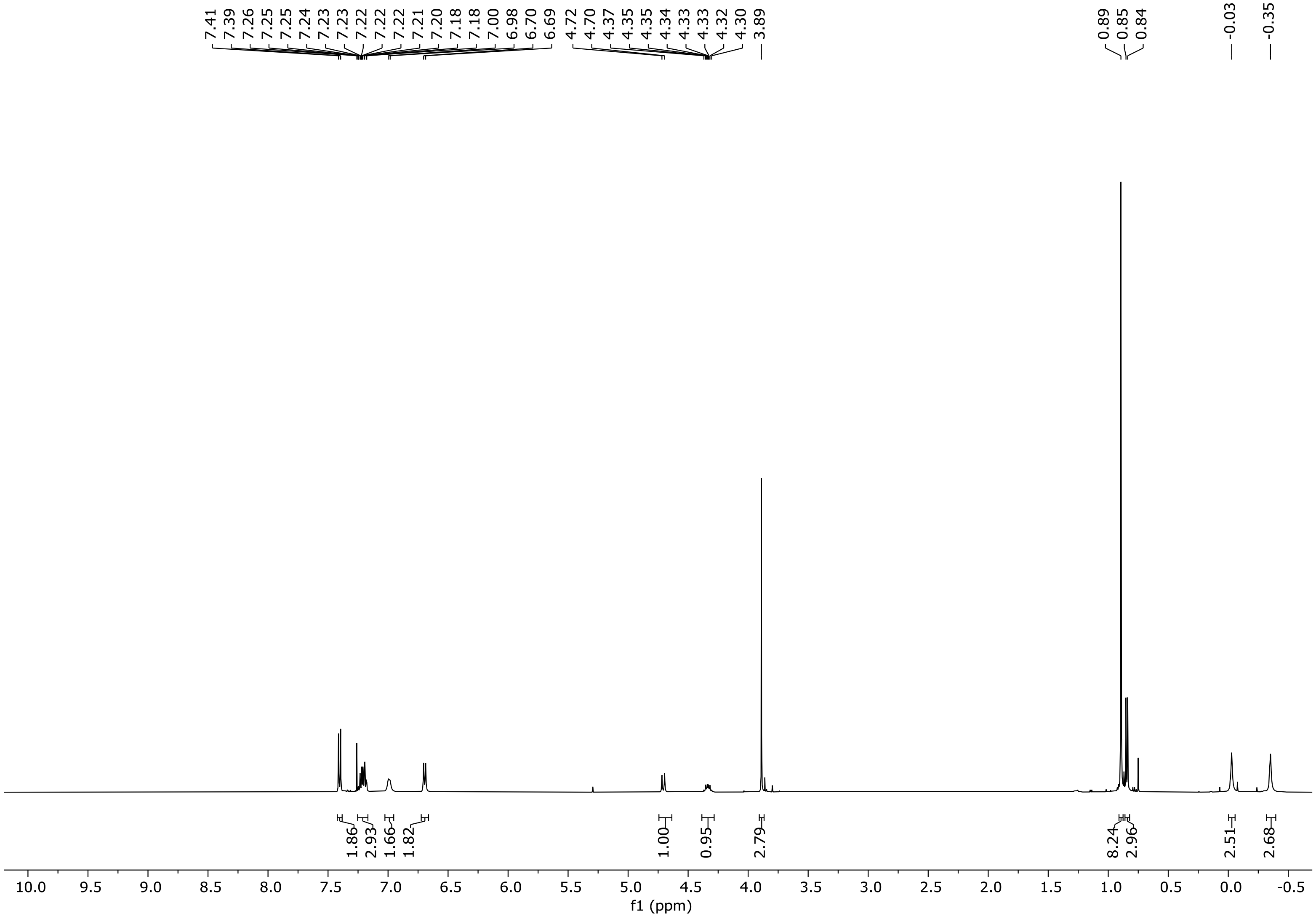

Figure S74: ^{13}C NMR (126 MHz, $CDCl_3$, 298 K) spectrum of **5k**.

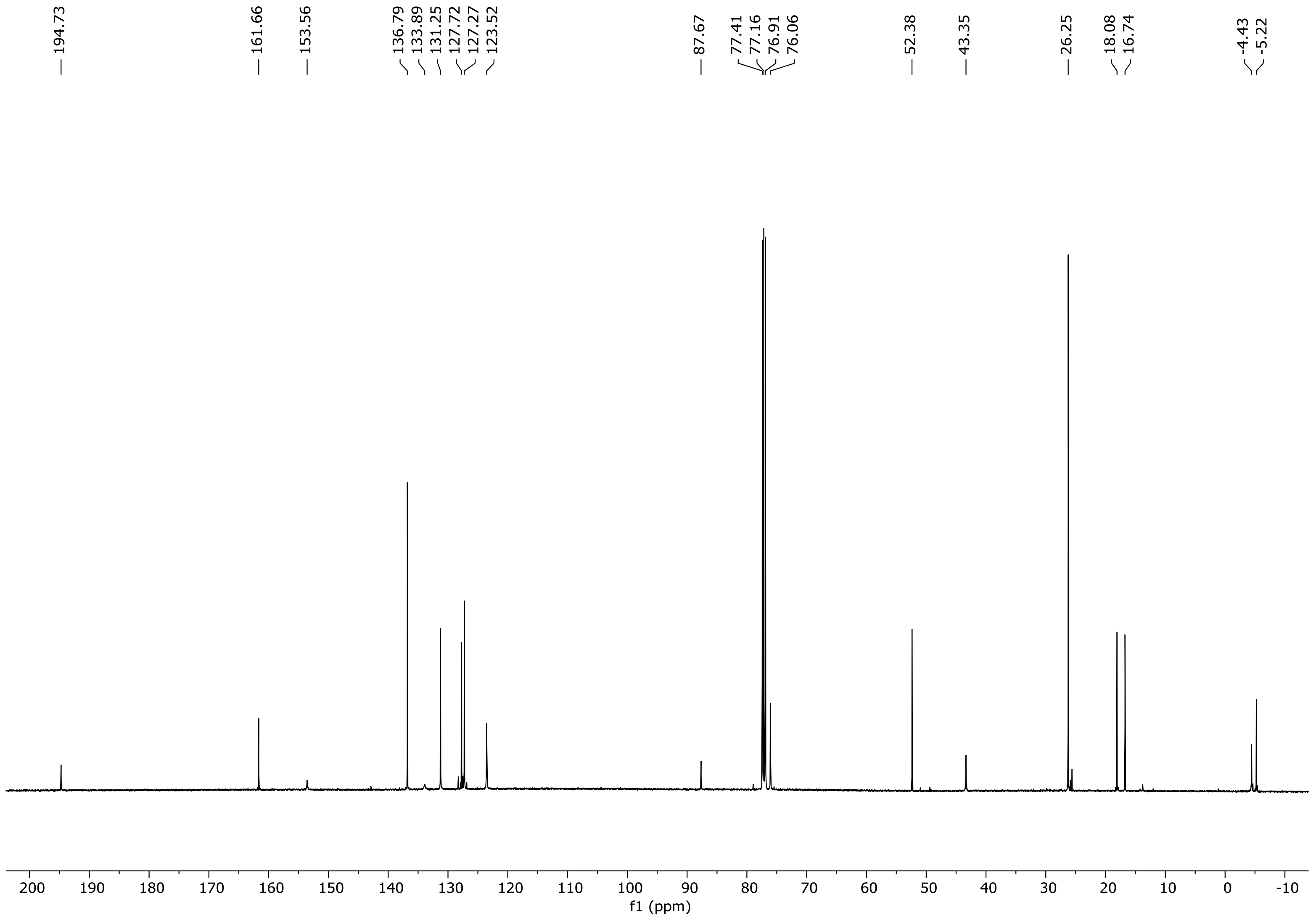

Figure S75: ^{1}H NMR (500 MHz, $CDCl_3$, 298 K) spectrum of **5l.**

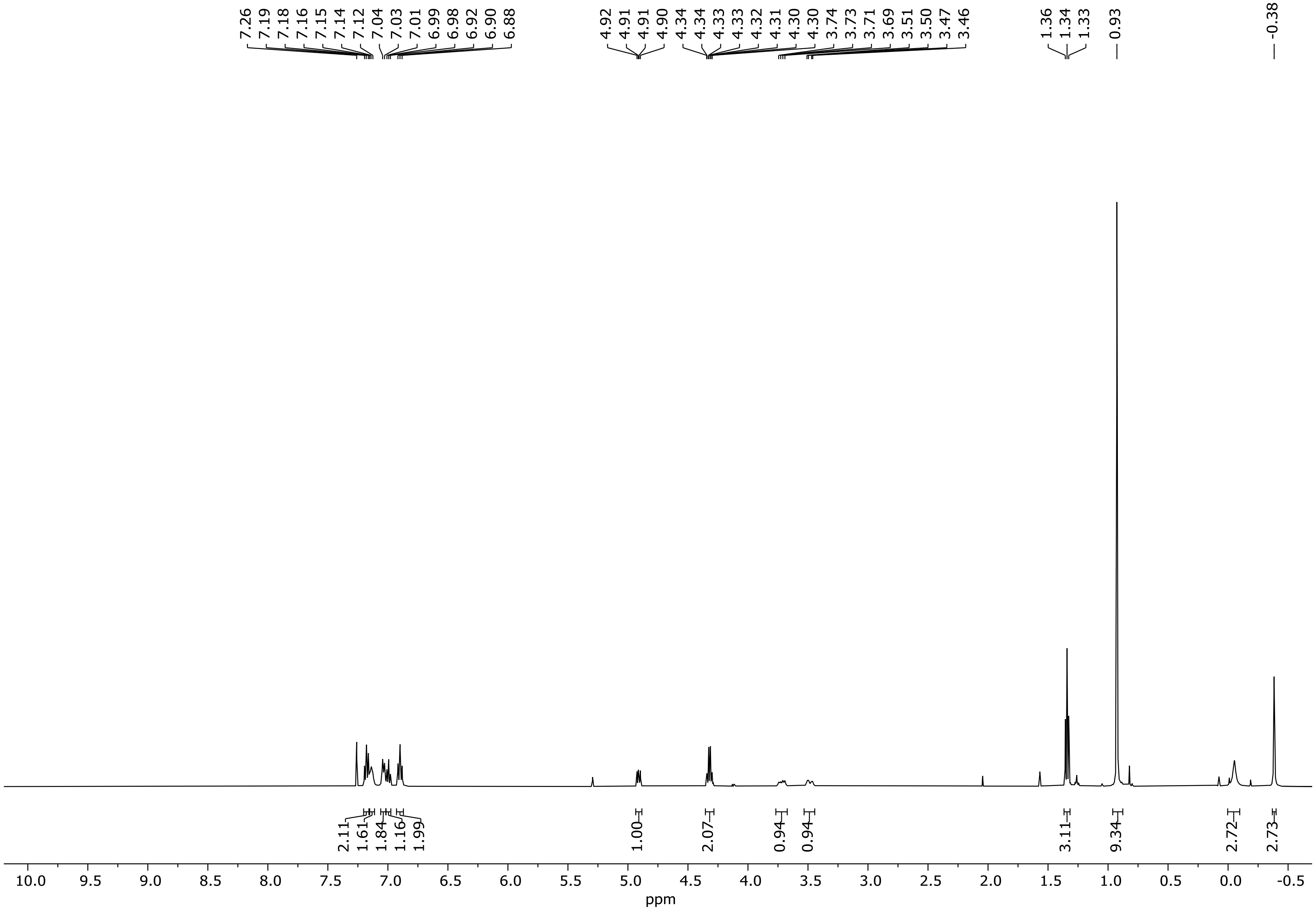

Figure S76: ^{13}C NMR (126 MHz, $CDCl_3$, 298 K) spectrum of **5l**.

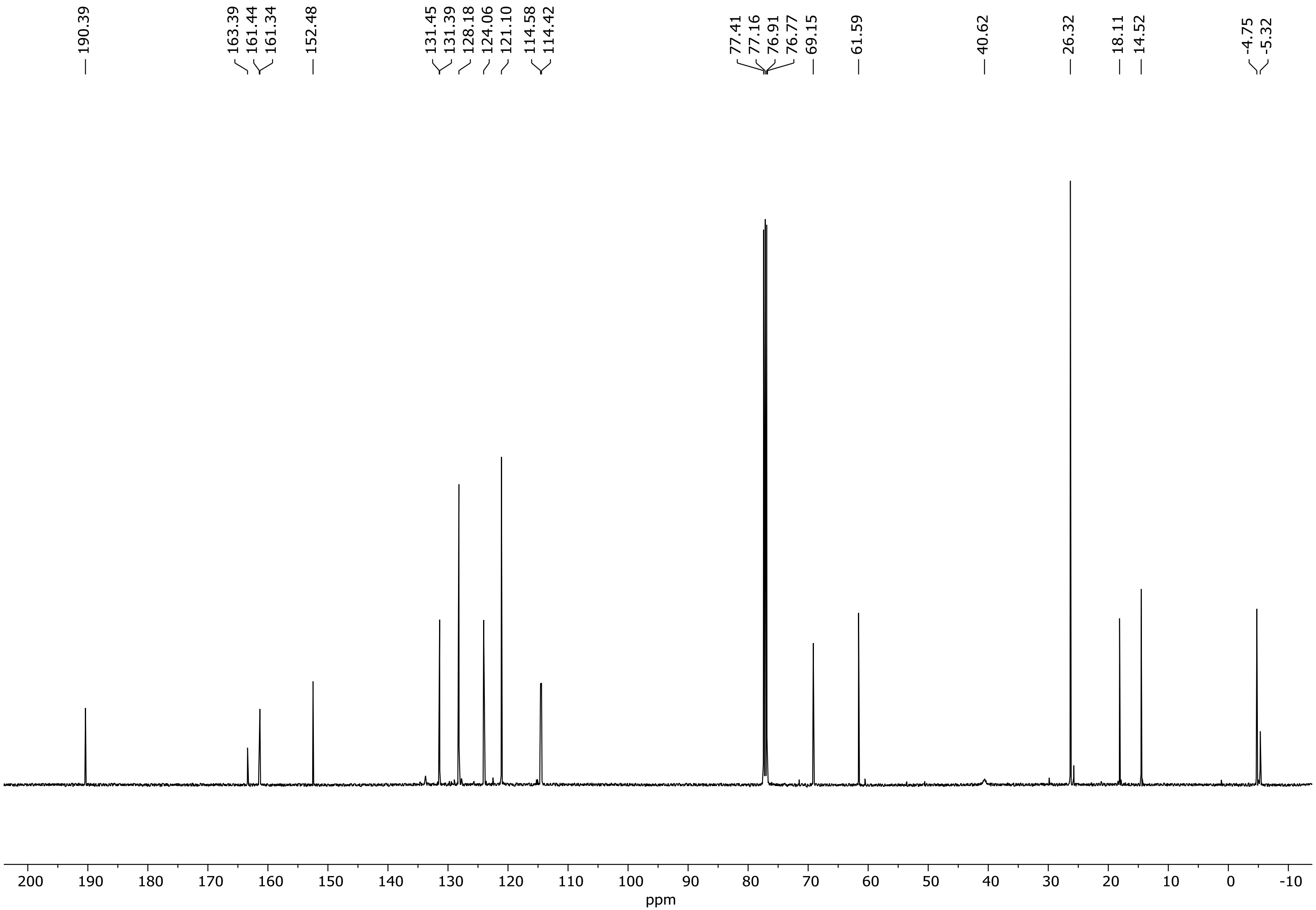

Figure S77: ^{19}F NMR (376 MHz, $CDCl_3$, 298 K) spectrum of **5l**.

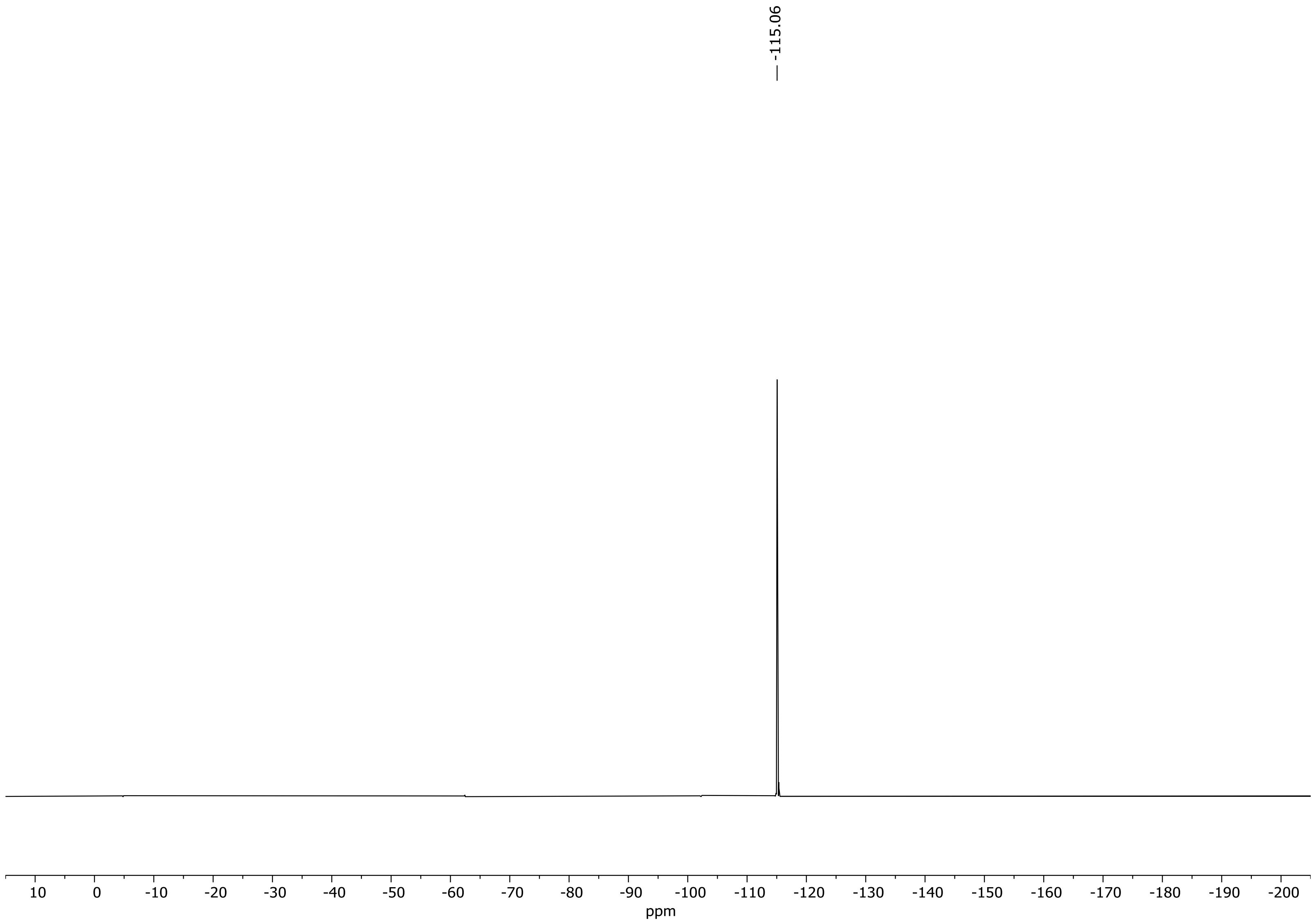

Figure S78: ^{1}H NMR (500 MHz, $CDCl_3$, 298 K) spectrum of **5m.**

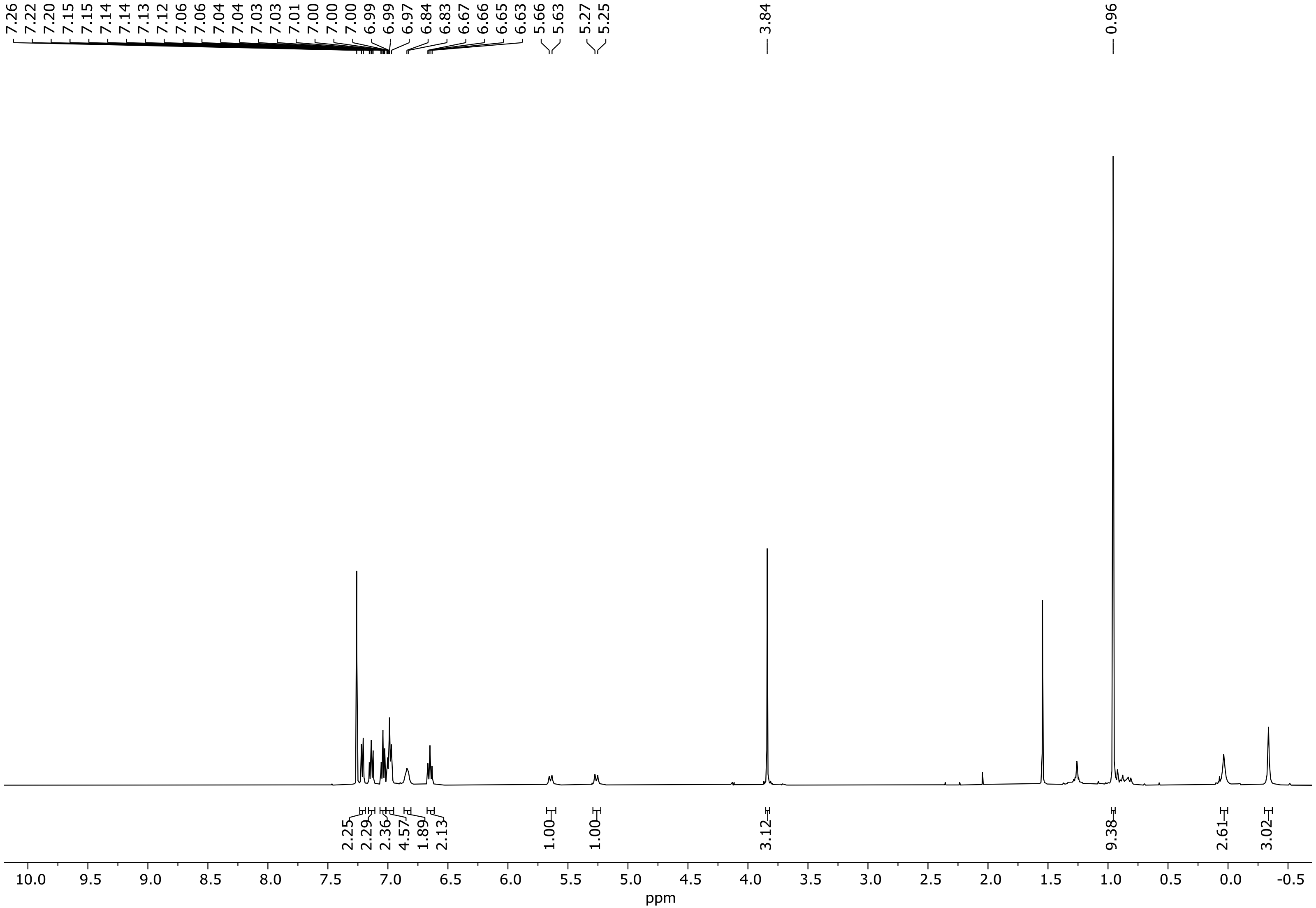

Figure S79: ^{13}C NMR (126 MHz, $CDCl_3$, 298 K) spectrum of **5m**.

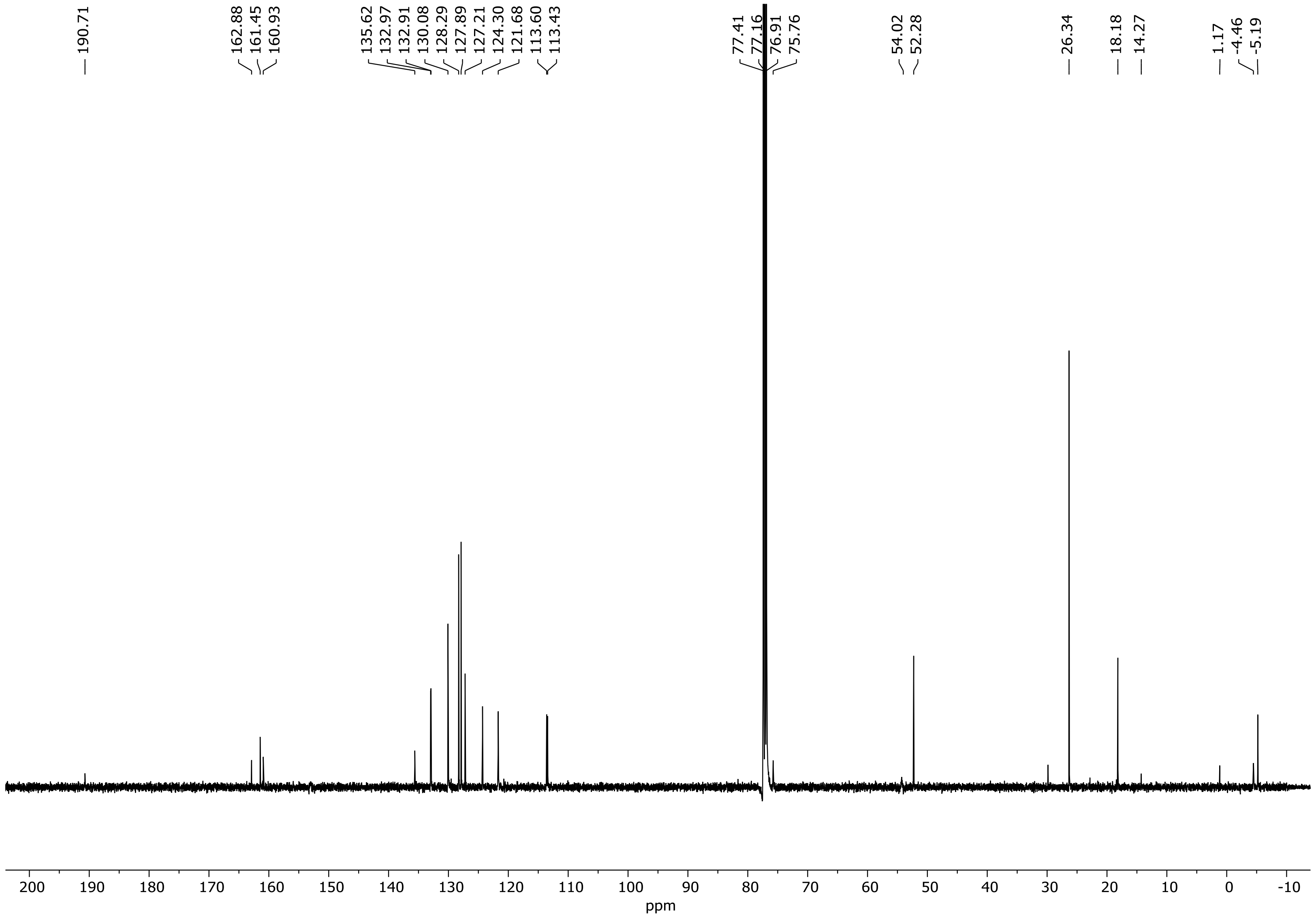

Figure S80: ^{19}F NMR (376 MHz, $CDCl_3$, 298 K) spectrum of **5m**.

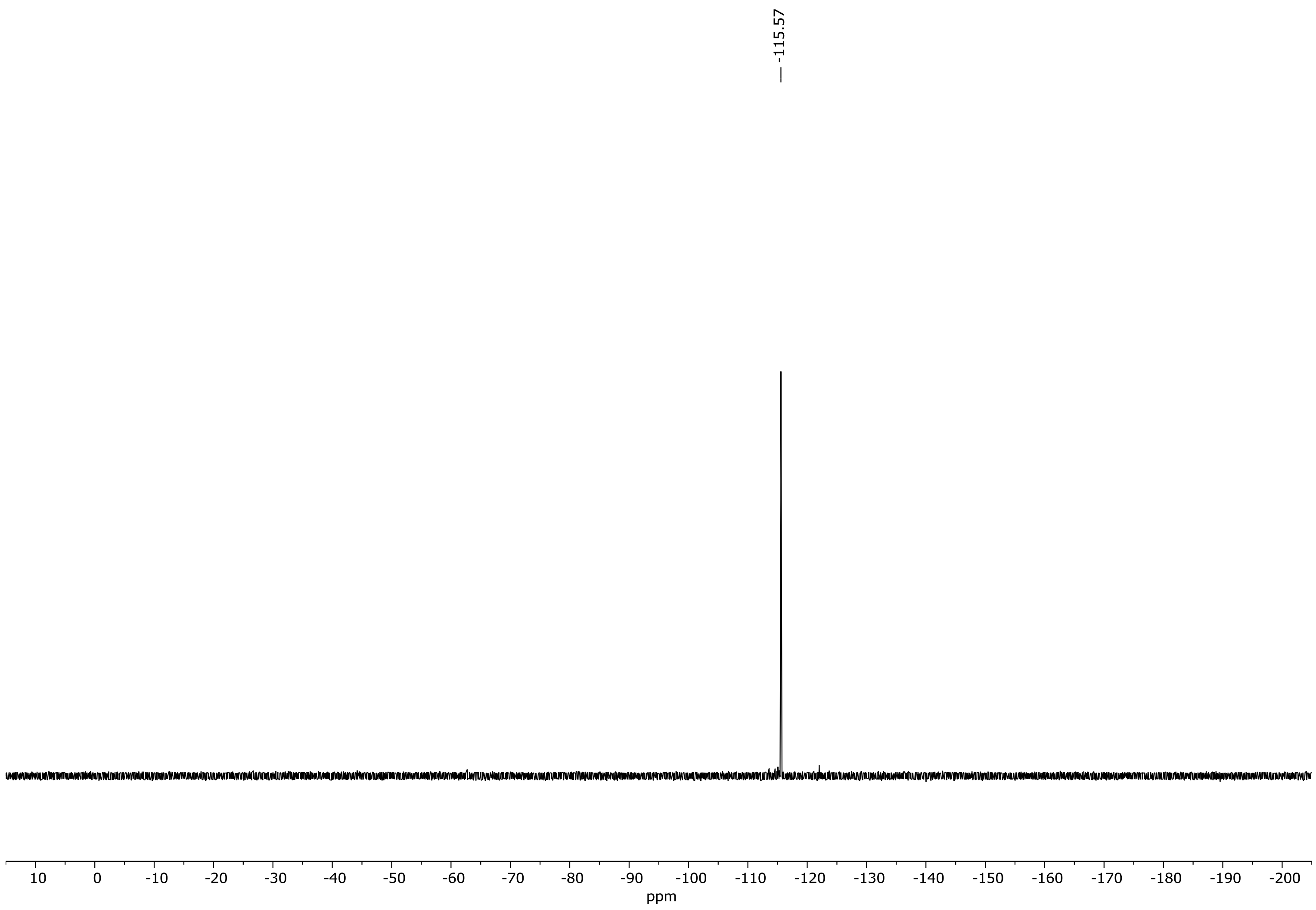

Figure S81: ^{1}H NMR (500 MHz, $CDCl_3$, 298 K) spectrum of **6a.**

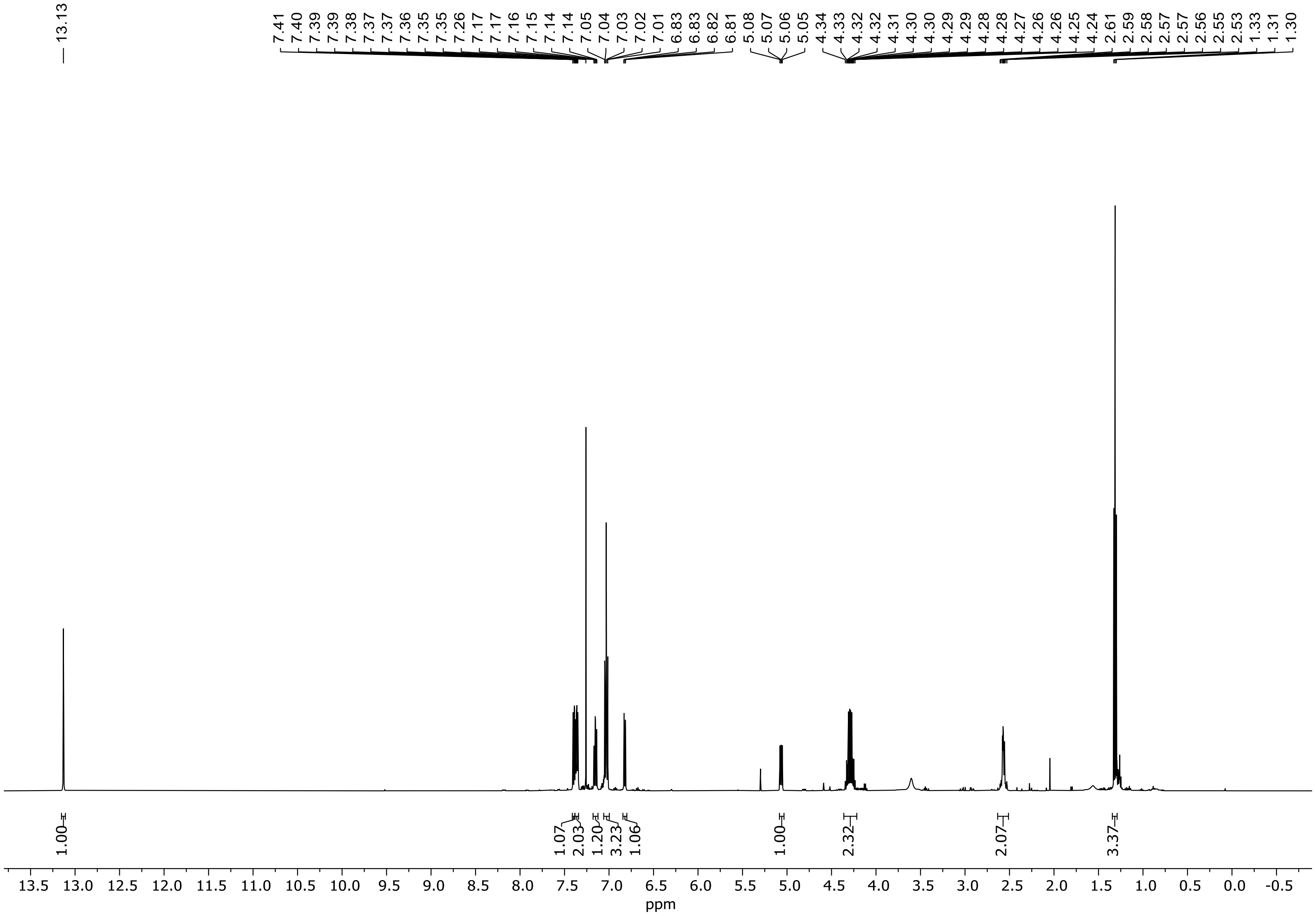

Figure S82: ^{13}C NMR (126 MHz, $CDCl_3$, 298 K) spectrum of **6a**.

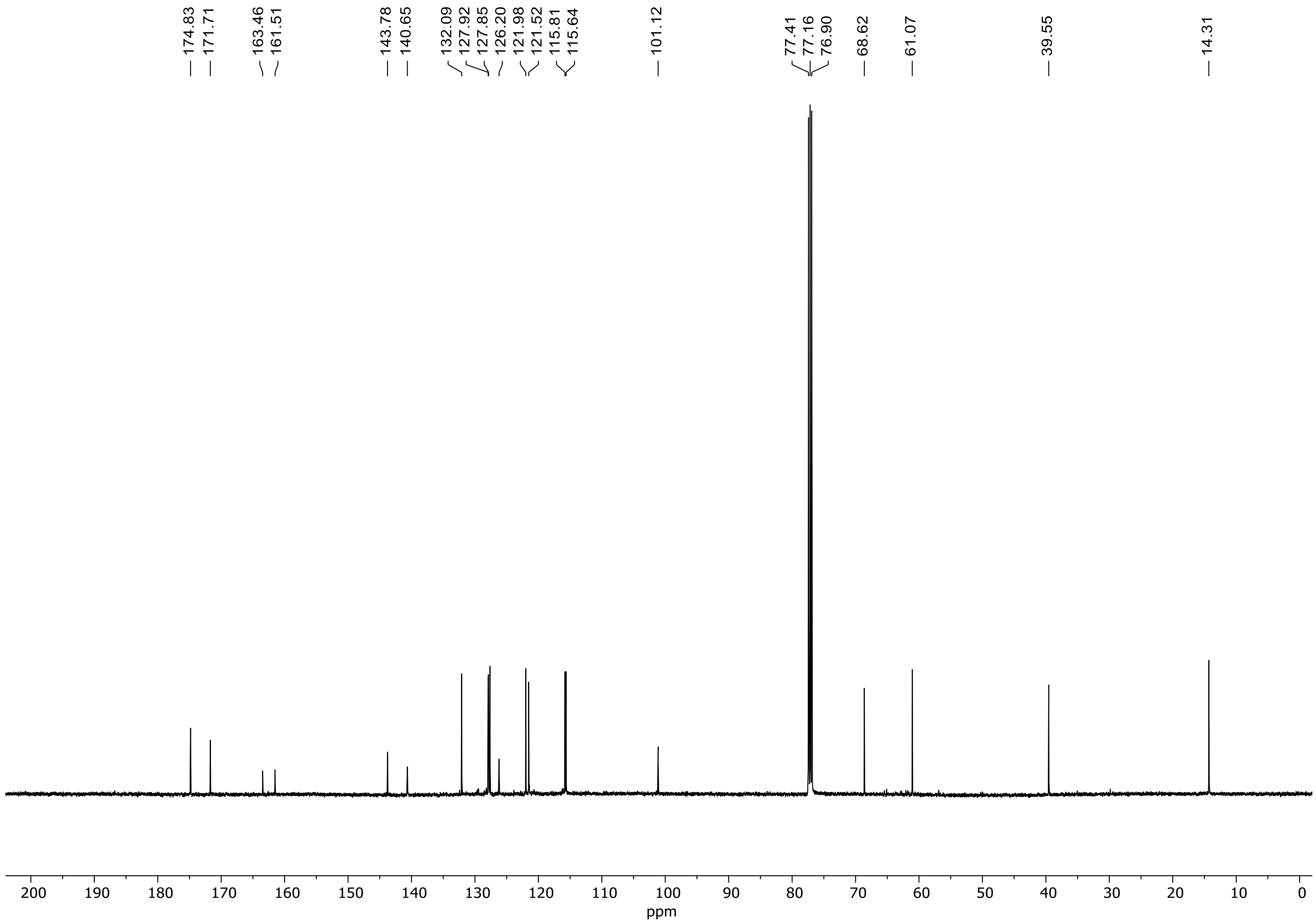

Figure S83: ^{19}F NMR (376 MHz, $CDCl_3$, 298 K) spectrum of **6a**.

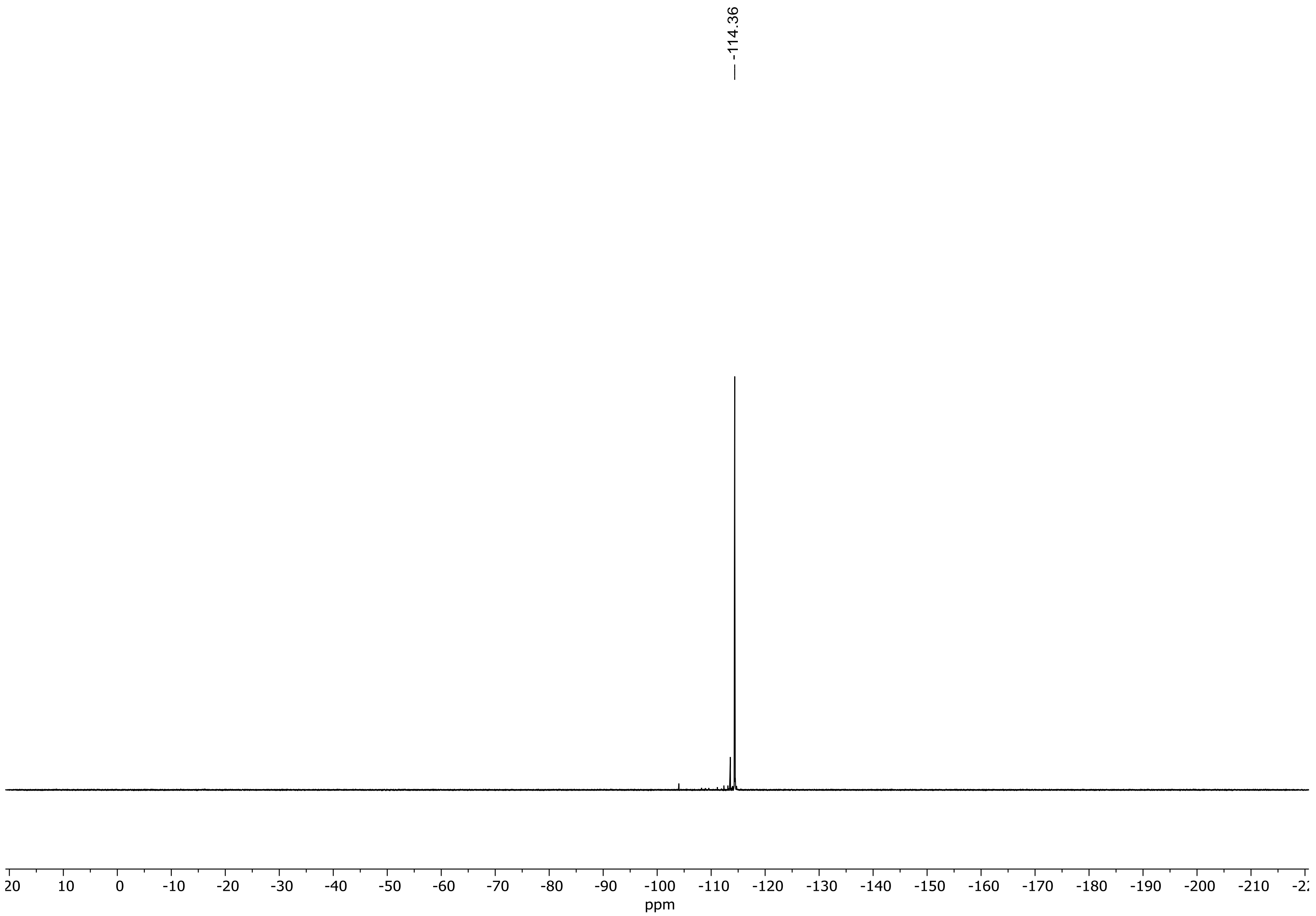

Figure S84: ^{1}H NMR (500 MHz, $CDCl_3$, 298 K) spectrum of **6b.**

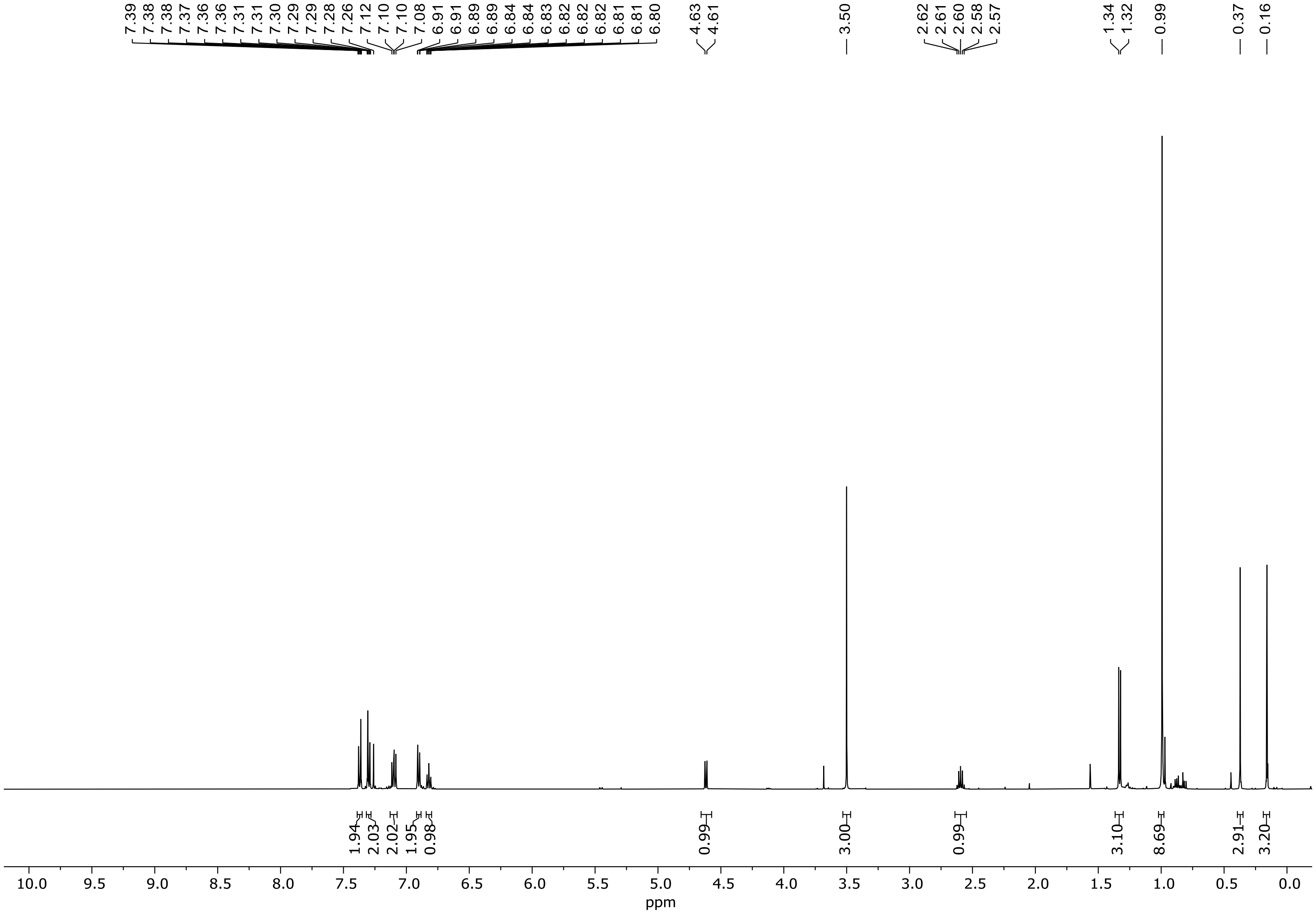

Figure S85: ^{13}C NMR (126 MHz, $CDCl_3$, 298 K) spectrum of **6b**.

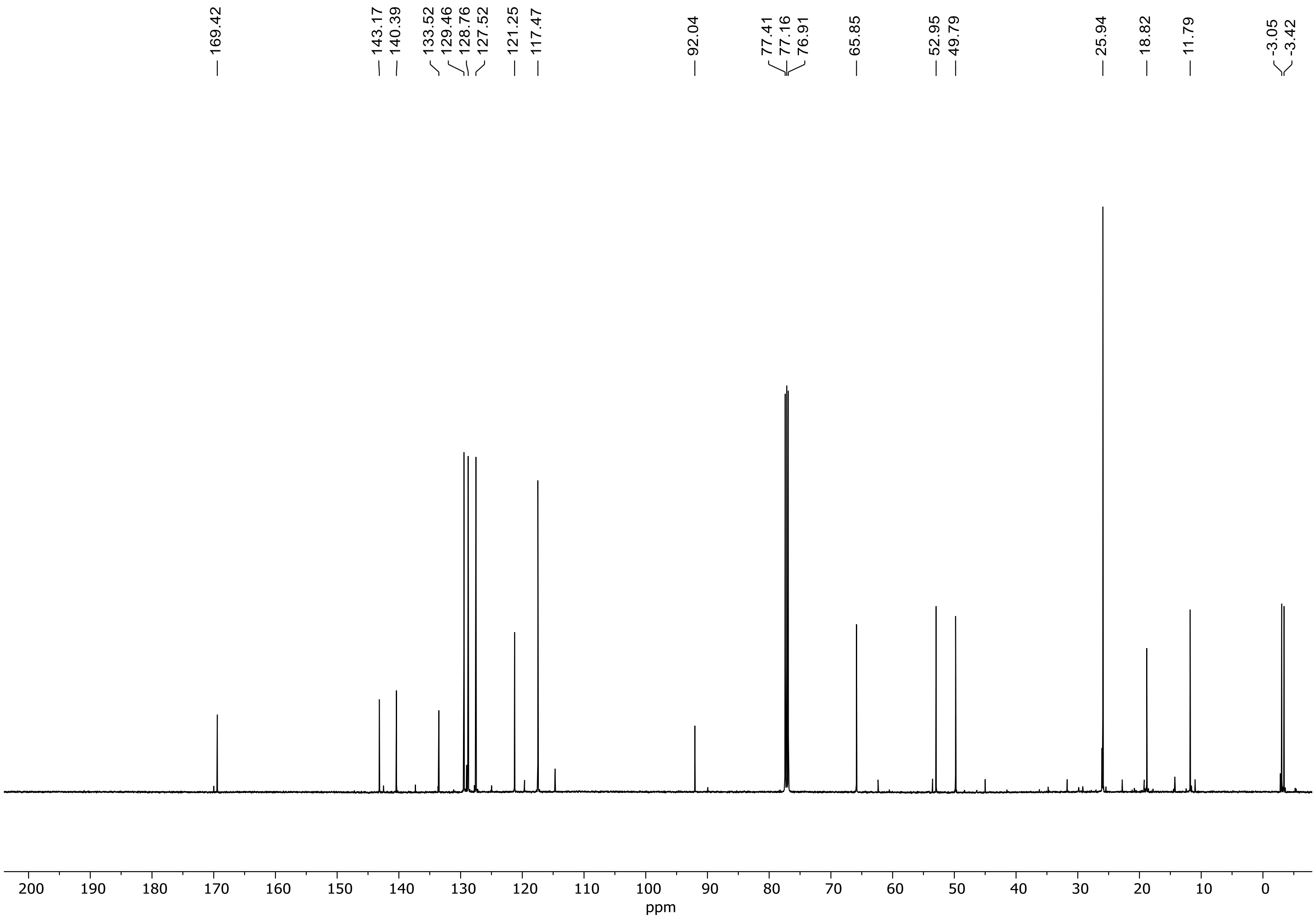

3.1 2D-NMR Spectra

Figure S86: HSQC NMR (500 MHz, $CDCl_3$, 298 K) spectrum of **3p**.

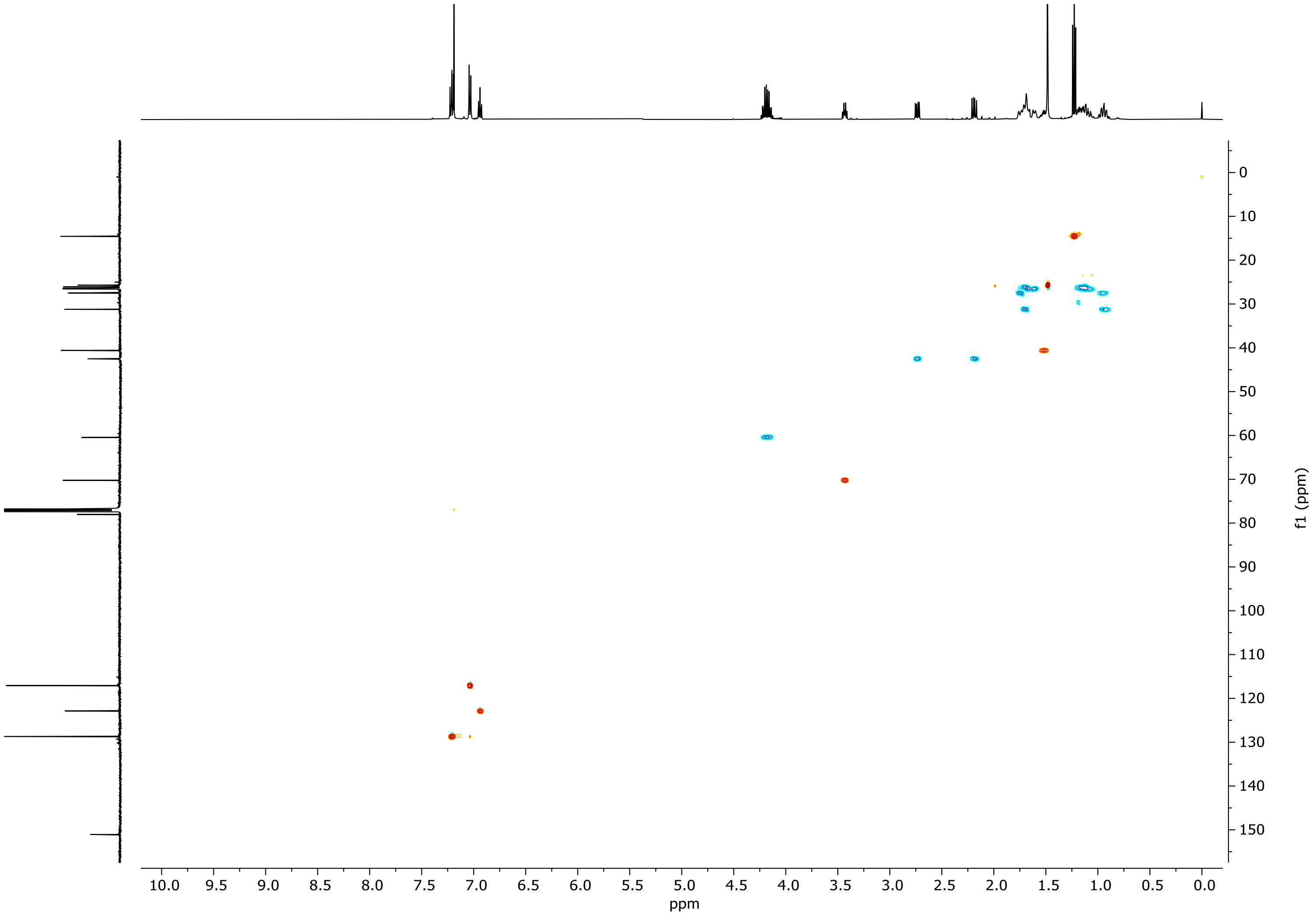

Figure S87: CoSY NMR (500 MHz, $CDCl_3$, 298 K) spectrum of **3p**.

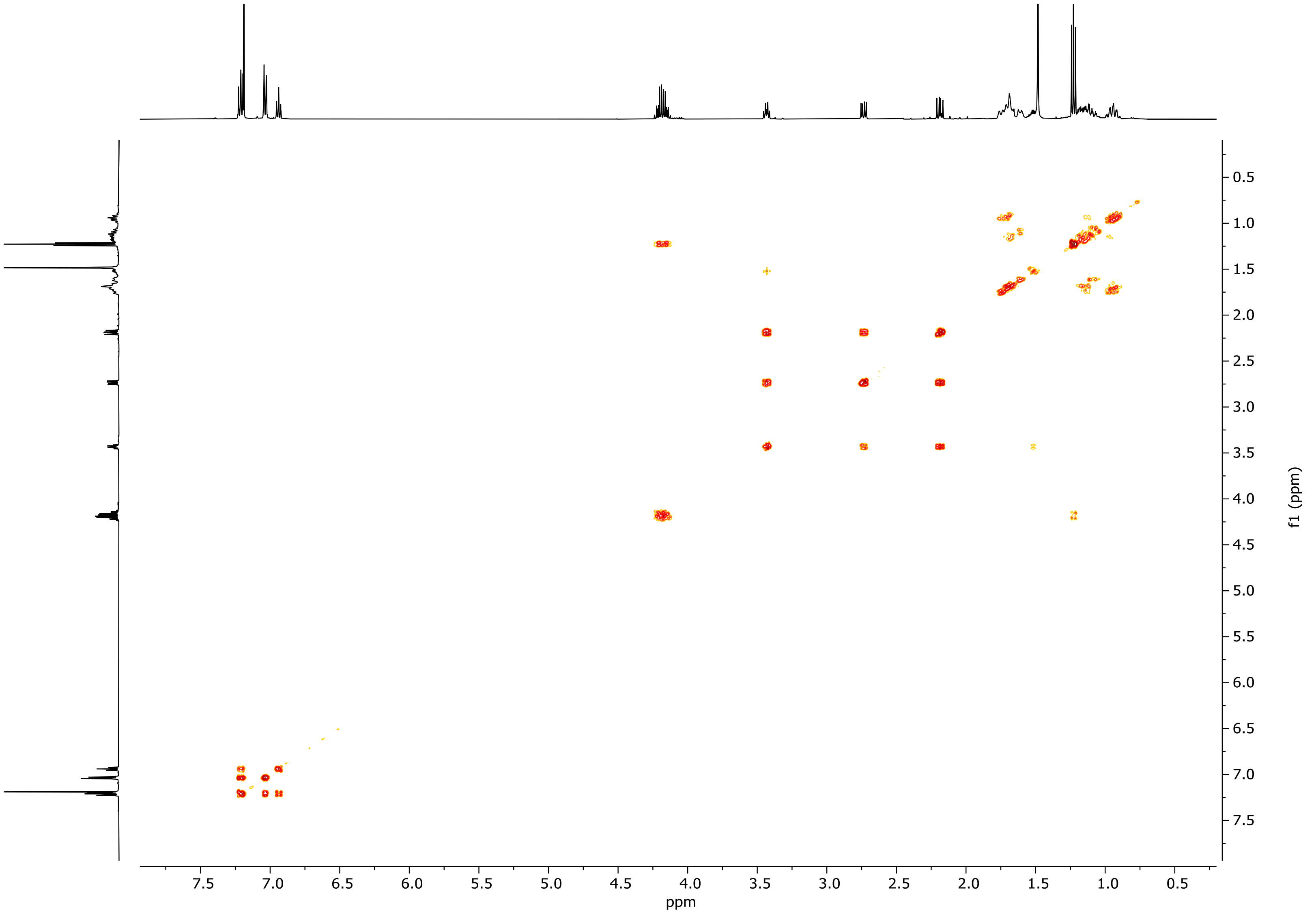

Figure S88: HSQC NMR (500 MHz, $CDCl_3$, 298 K) spectrum of *anti*-**5d**.

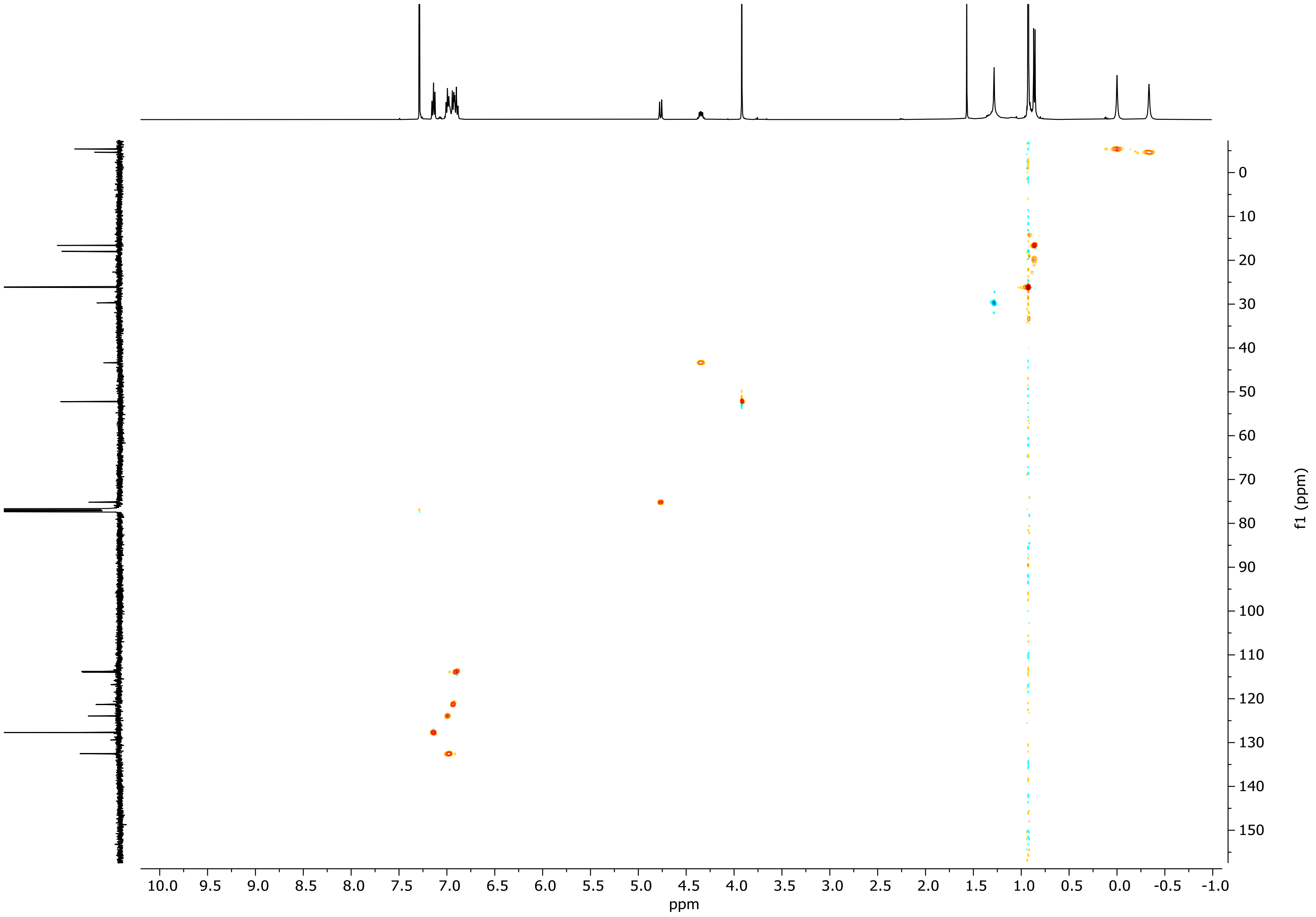

Figure S89: CoSY NMR (500 MHz, $CDCl_3$, 298 K) spectrum of *anti*-**5d**.

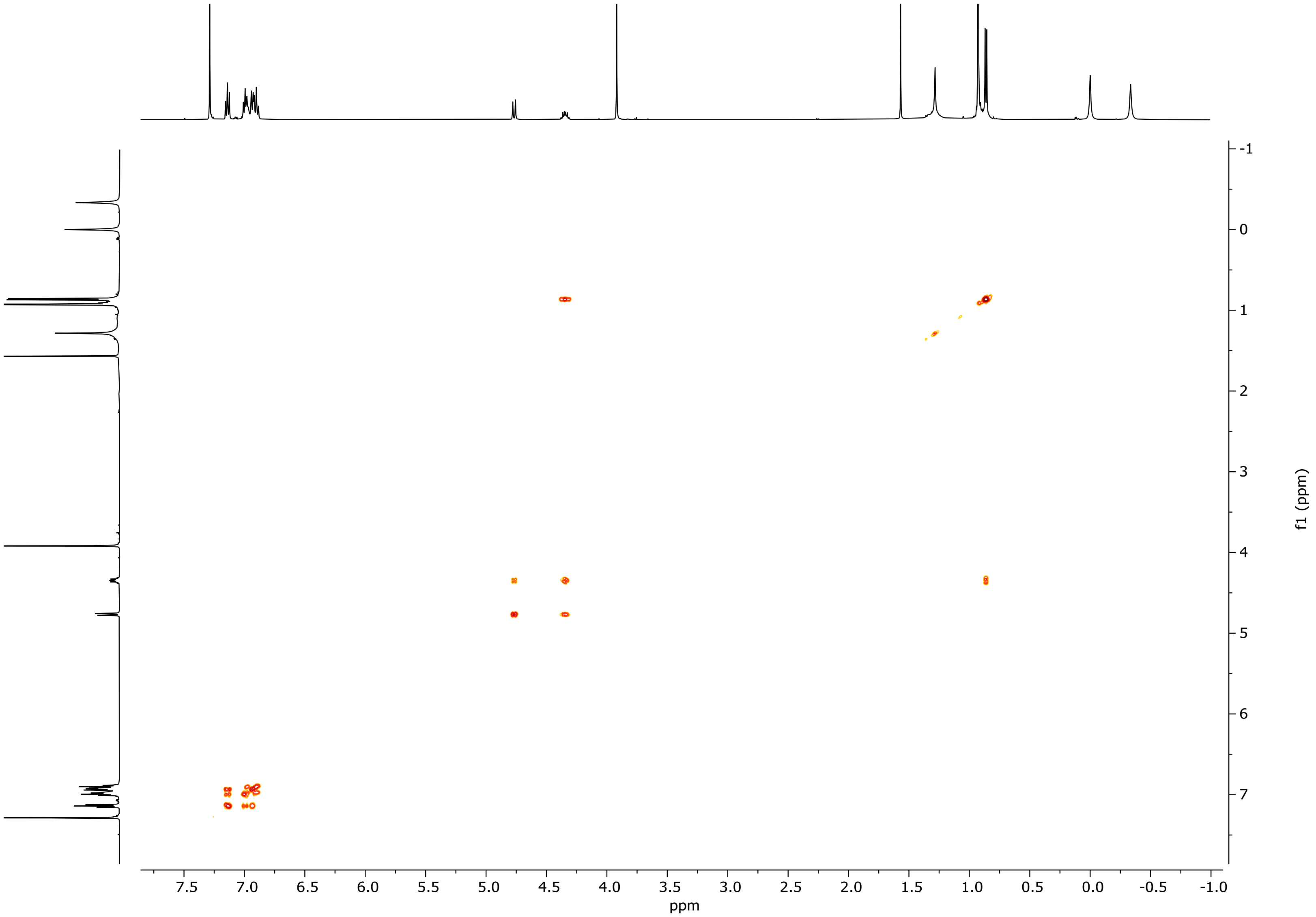

Figure S90: HSQC NMR (500 MHz, $CDCl_3$, 298 K) spectrum of **6a**. The OH peak at 13.13 ppm couldn't be visualized with this technique.

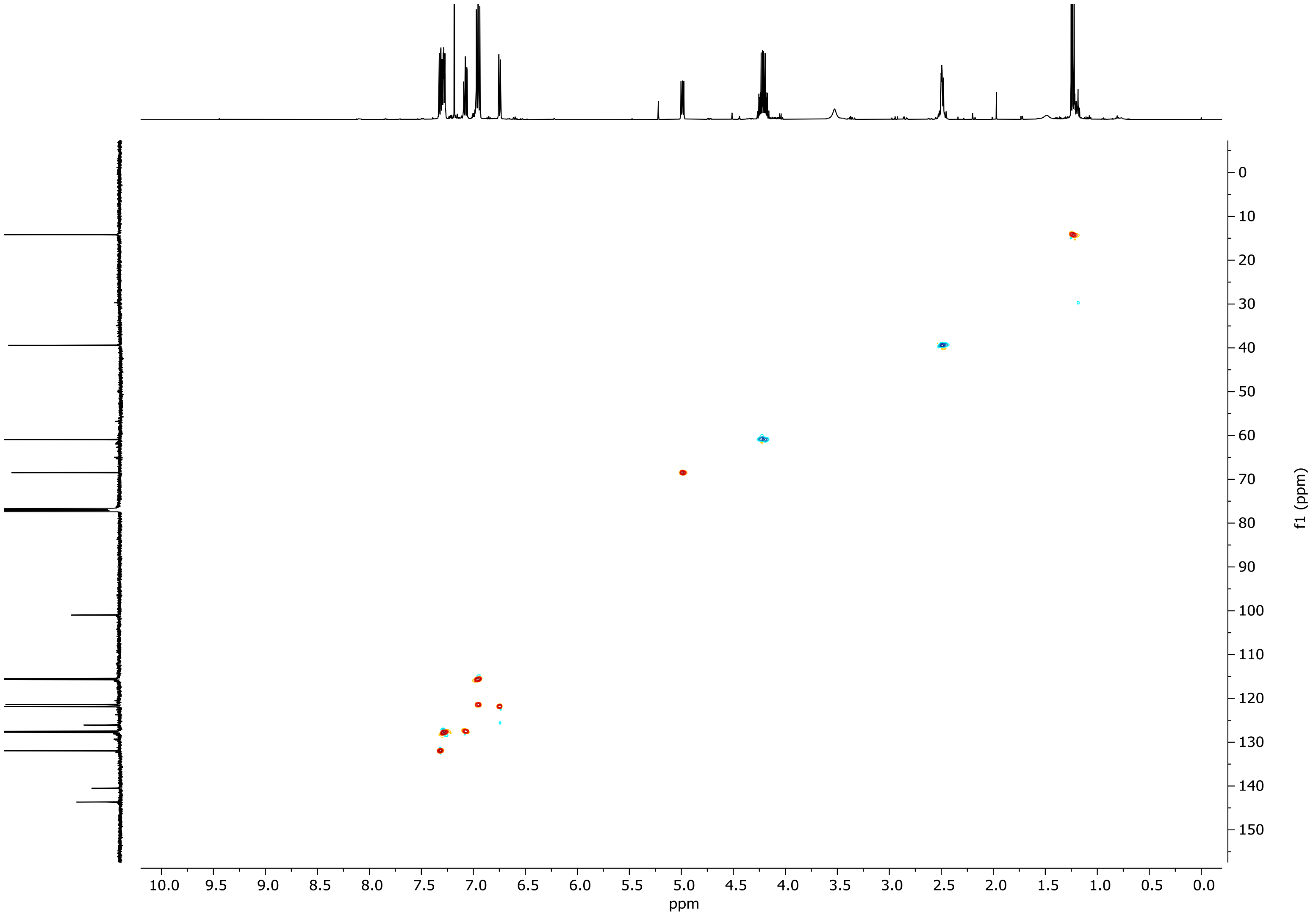

Figure S91: HMBC NMR (500 MHz, $CDCl_3$, 298 K) spectrum of **6a**.

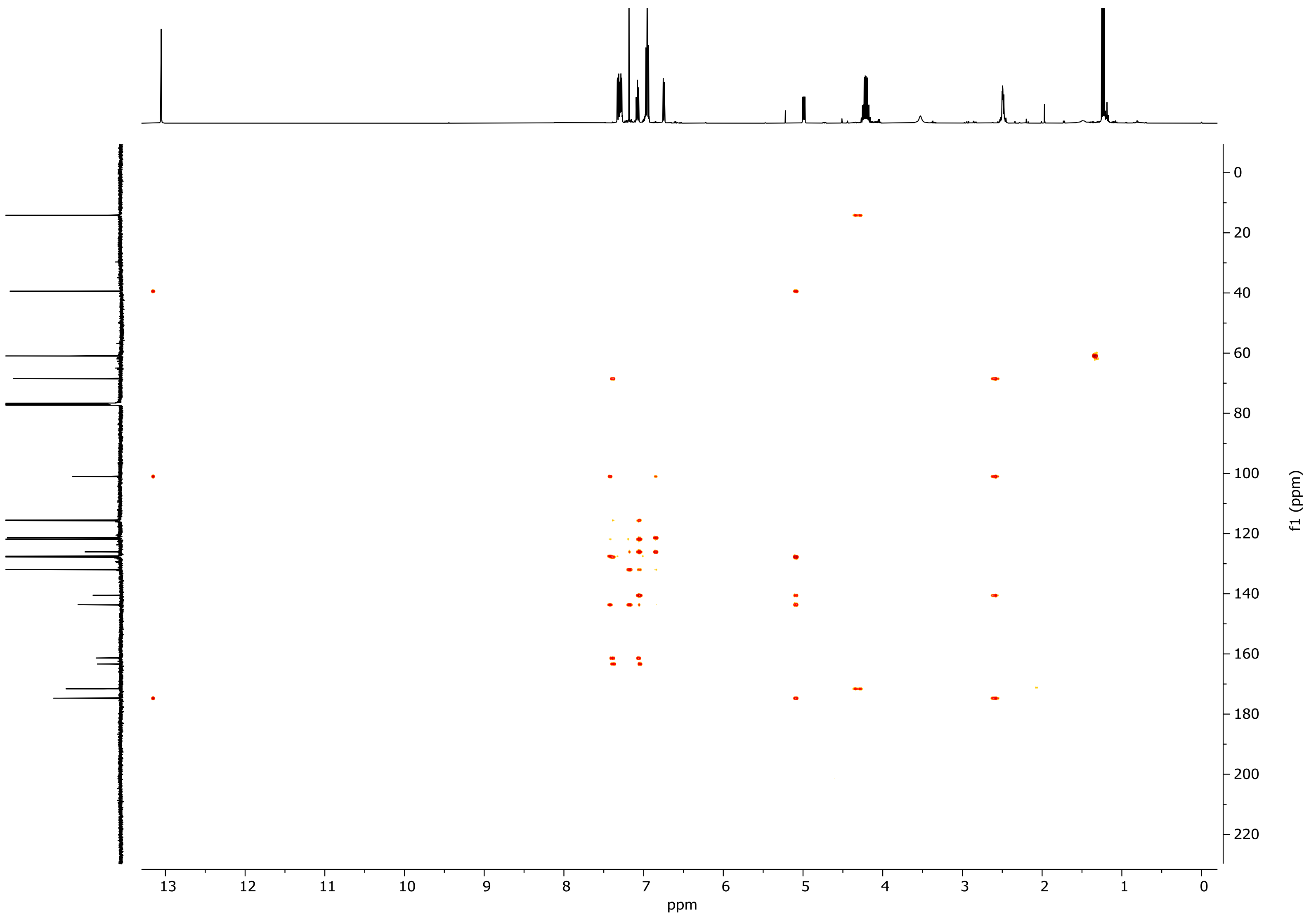

Figure S92: CoSY NMR (500 MHz, $CDCl_3$, 298 K) spectrum of **6a**.

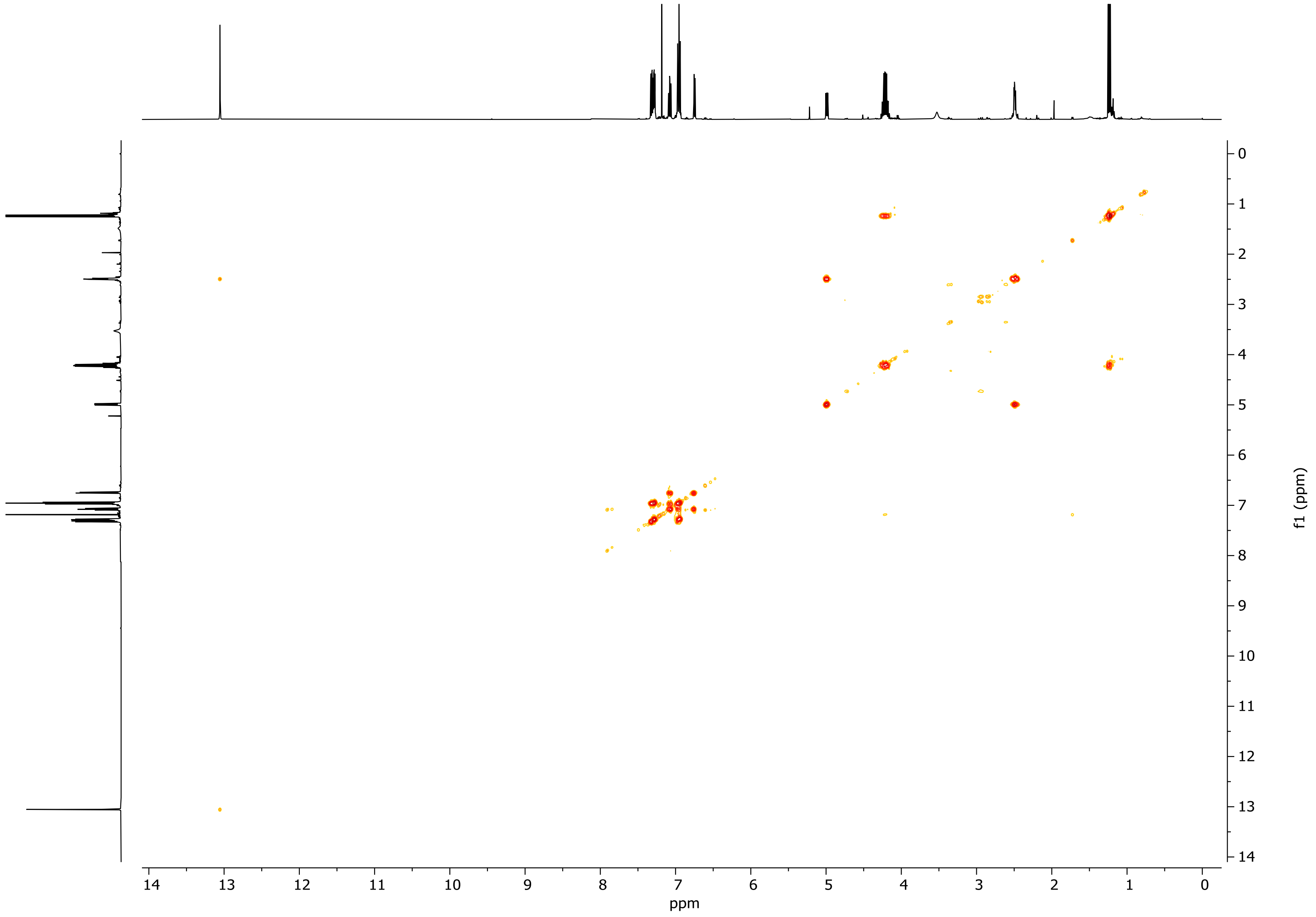

Figure S93: HSQC NMR (500 MHz, $CDCl_3$, 298 K) spectrum of **6b**.

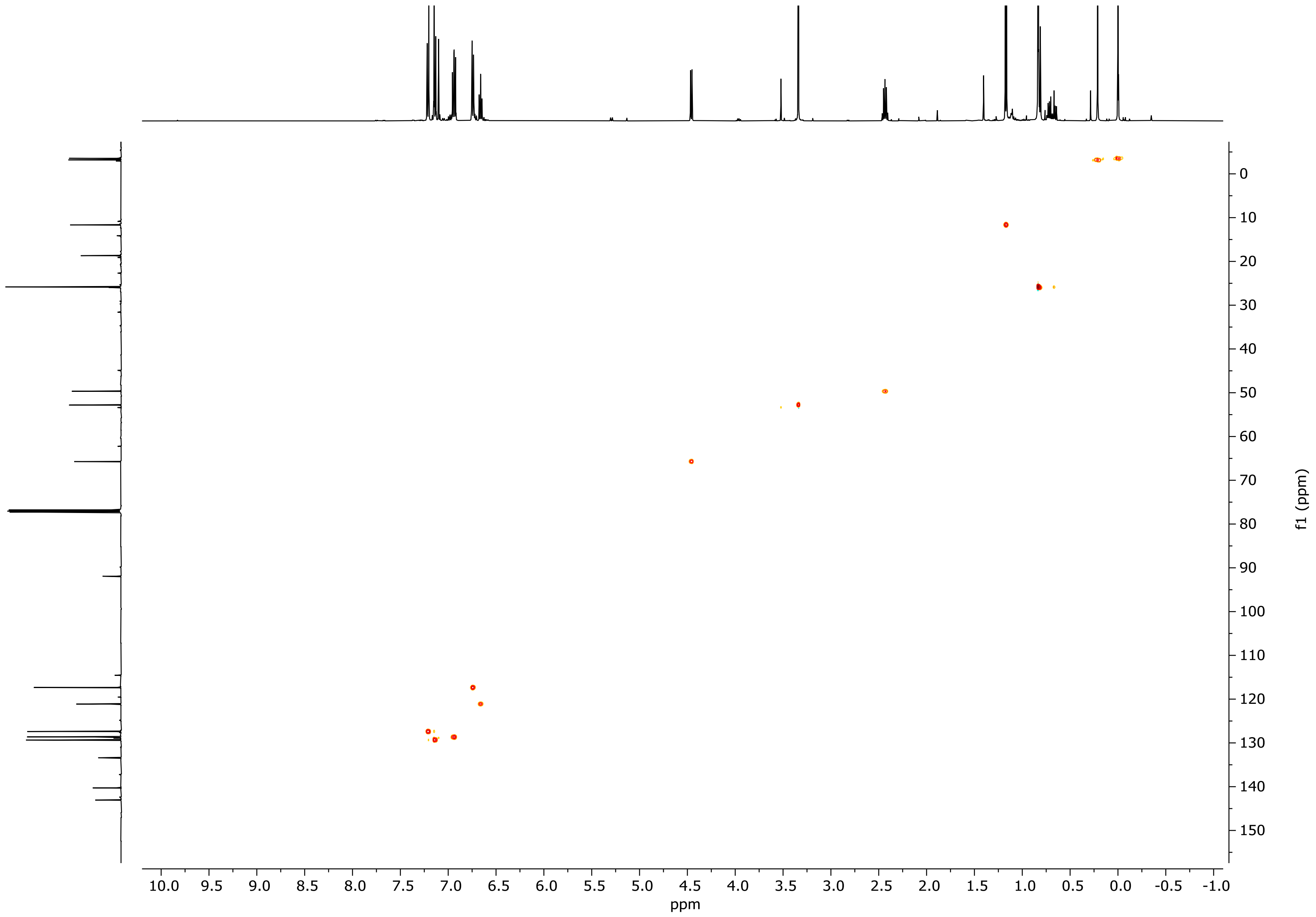

Figure S94: HMBC NMR (500 MHz, $CDCl_3$, 298 K) spectrum of **6b**.

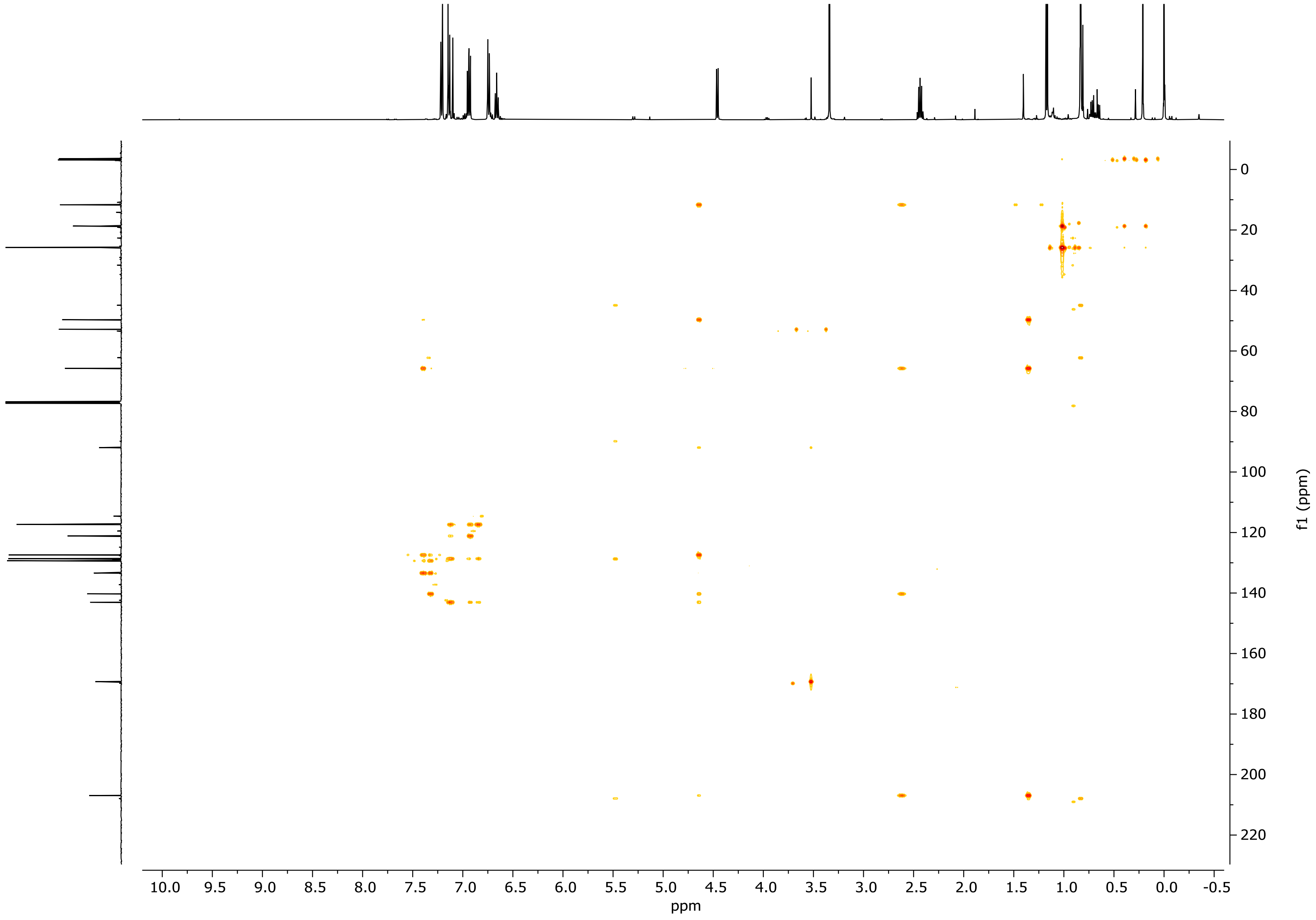

Figure S95: CoSY NMR (500 MHz, $CDCl_3$, 298 K) spectrum of **6b**.

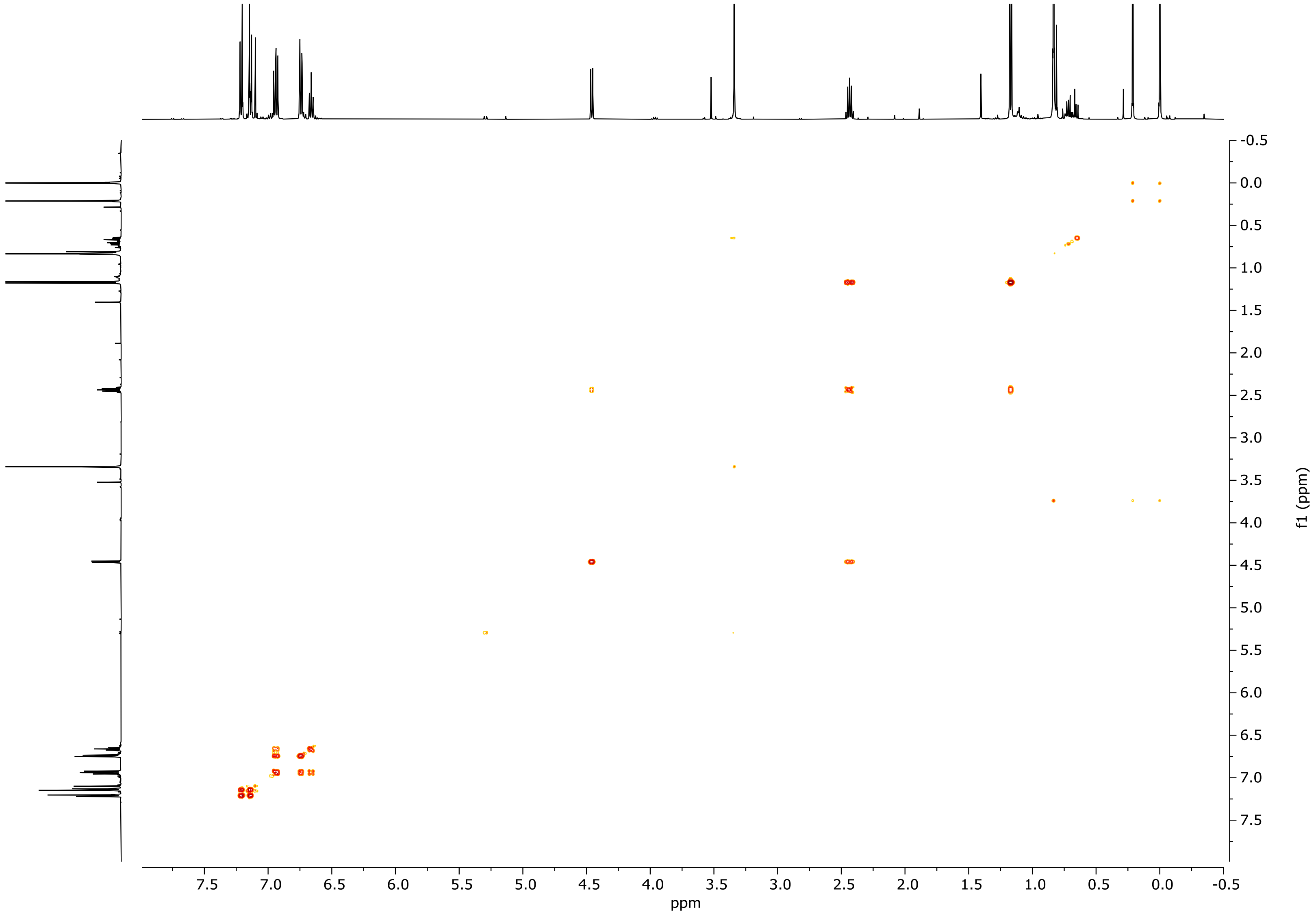

4.0 Crystallographic Data

4.1 Single Crystal X-ray Diffraction

Single crystals of **3i** and **5d** were grown in a fume hood by slow evaporation from 1,2-dichloromethane and acetonitrile respectively at 5 ^{o}C. Crystallographic studies were undertaken on a single crystal mounted in Fomblin®Y and studied on an Agilent SuperNova Dual Atlas three-circle diffractometer using Mo- or Cu-Kα radiation and a CCD detector. Measurements were taken at 293(2) K with temperatures maintained using an Oxford cryostream. Data were collected, integrated, and corrected for absorption within CrysAlisPro.[18] The absorption correction implemented a numerical absorption correction based on Gaussian integration over a multifaceted crystal model. The structure was solved by intrinsic phasing and refined against F^2 within SHELXL-2013.[19] The structure has been deposited with the Cambridge Structural Database [CCDC deposition numbers 2192556 (**3i**) and 2192557 (**5d**)]. This can be obtained free of charge from the Cambridge Crystallographic Data Centre via www.ccdc.cam.ac.uk/data_request/cif.

4.2 Solid-State Structures

Figure S96. Solid-state structure of compound **3i**, thermal ellipsoids drawn at 50% probability. H atoms omitted for clarity. C atoms in black, N in blue, O in red.

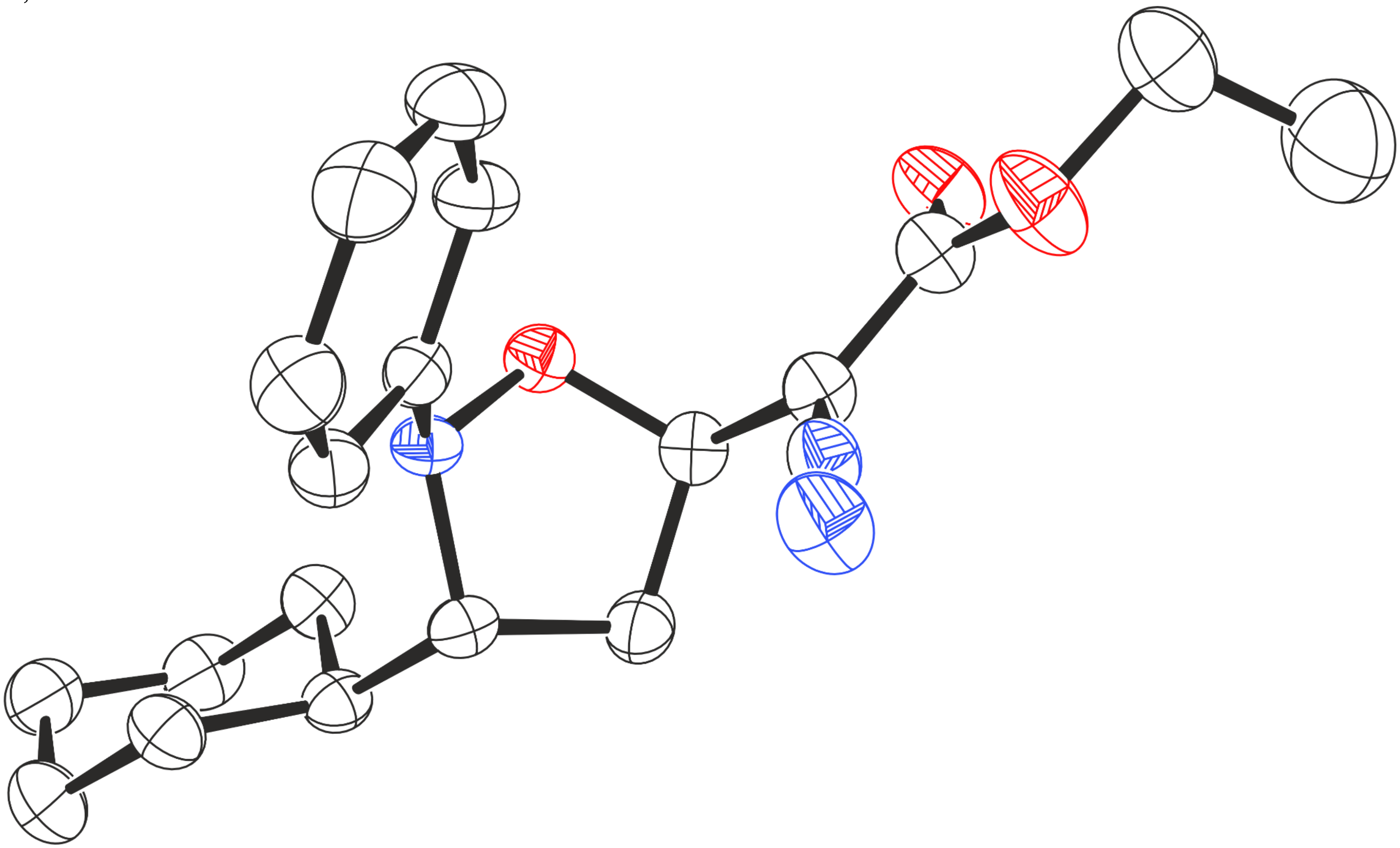

Figure S97. Solid-state structure of compound **5d**, thermal ellipsoids drawn at 50% probability. H atoms omitted for clarity. C atoms in black, N in blue, O in red, Si in beige, F in green.

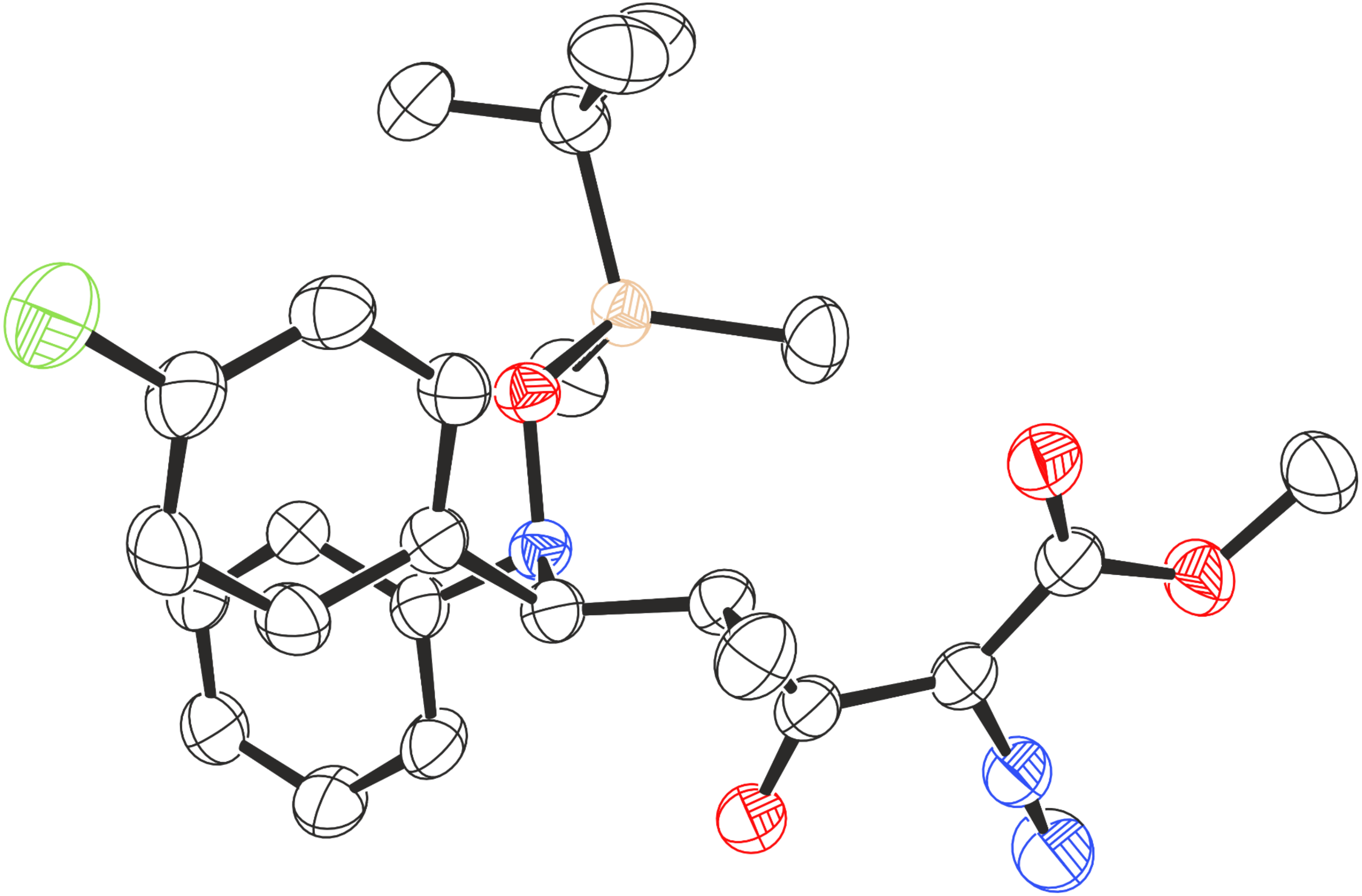

4.3 X-Ray Refinement Data

Table S1. Crystal data and structure refinement for compound **3i**.

**Compound**	**3i**
Empirical formula	$C_{19}H_{25}N_3O_3$
M_r	343.42
Crystal system	Monoclinic
Space group	C2/*c*
Temperature (K)	293
a, *b*, *c* (Å)	22.7917(12), 5.3754(4). 29.5181(18)
α, β, γ (°)	90, 94.943(5), 90
Volume, V (Å^3)	3602.9(4)
Z	8
Density, calc (g cm^{-3})	1.266
Absorption coefficient, μ (mm^{-1})	0.70
Crystal size (mm)	0.49 × 0.15 × 0.09
Radiation type	Cu K\a
Wavelength (Å)	1.54178
θ range (°)	4.7–71.9
Index ranges	-27 ≤ h ≤ 27
	-6 ≤ k ≤6
	-35 ≤ l ≤ 35
Reflections collected	15686
Independent reflections	3555
R(int)	0.051
Absorption correction	Gaussian
Data / restraints / parameters	3555 / 0 / 228
Goodness of fit, S	1.05
Final R indices [I>2σ(I)]	R_1 = 0.0557
	wR_2 = 0.1403
R indices (all data)	R_1 = 0.0740
	wR_2 = 0.1571
Max residual electron density (e^-Å^{-3})	0.60
Min residual electron density (e^-Å^{-3})	-0.41

Table S2. Crystal data and structure refinement for compound **5d**.

**Compound**	**5d**
Empirical formula	$C_{25}H_{32}FN_3O_4Si$
M_r	485.62
Crystal system	Monoclinic
Space group	$P2_1/n$
Temperature (K)	293
a, *b*, *c* (Å)	9.8475(3), 10.9203(2), 24.8357(6)
α, β, γ (°)	90, 100.494(2), 90
Volume, V (Å^3)	2626.10(11)
Z	4
Density, calc (g cm^{-3})	1.228
Absorption coefficient, μ (mm^{-1})	1.138
Crystal size (mm)	0.426 x 0.185 x 0.097
Radiation type	Cu K\a
Wavelength (Å)	1.54178
θ range (°)	4.3790-73.0050
Index ranges	$-11 \leq h \leq 12$
	$-13 \leq k \leq 13$
	$-26 \leq l \leq 30$
Reflections collected	5177
Independent reflections	4451
R(int)	0.0308
Absorption correction	Gaussian
Data / restraints / parameters	5177 / 0 / 321
Goodness of fit, S	1.021
Final R indices [I>2σ(I)]	R_1 = 0.0443
	wR_2 = 0.1048
R indices (all data)	R_1 = 0.0373
	wR_2 = 0.0976
Max residual electron density (e^-Å^{-3})	0.330
Min residual electron density (e^-Å^{-3})	-0.243

5.0 References

[1] Santi, M.; Ould, D. M. C.; Wenz, J.; Soltani, Y.; Melen, R. L.; Wirth, T. Metal-Free Tandem Rearrangement/Lactonization: Access to 3,3-Disubstituted Benzofuran-2-(3H)-ones. *Angew. Chem. Int. Ed.* **2019**, *58*, 7861–7865.

[2] Zheng, H.; Wang, K.; Faghihi, I.; Griffith, W. P.; Arman, H.; Doyle, M. P. Diverse Reactions of Vinyl Diazo Compounds with Quinone Oxonium Ions, Quinone Imine Ketals, and Eschenmoser's Salt. *ACS Catal*. **2021**, *11*, 9869−9874.

[3] Lo, M. M.; Fu, G. C. Cu(I)/Bis(azaferrocene)-Catalyzed Enantioselective Synthesis of β-Lactams via Coupling of Alkynes with Nitrones. *J. Am. Chem. Soc.* **2002**, *124*, 4572−4573.

[4] Choi, M.; Viji, M.; Kim, D.; Lee, Y. H.; Sim, J.; Kwak, Y.-S.; Lee, K.; Lee, H.; Jung, J.-K. Metal-free approach for the L-proline mediated synthesis of nitrones from nitrosobenzene. *Tetrahedron* **2018**, *74*, 4182–4187.

[5] Tian, Z.; Xu, J.; Liu, B.; Tan, Q.; Xu, B. Copper-Catalyzed Synthesis of Polysubstituted Pyrroles through [3+1+1] Cycloaddition Reaction of Nitrones and Isocyanides. *Org. Lett.* **2018**, *20*, 2603–2606.

[6] Liu, T.; Liu, Z.; Liu, Z.; Hu., Z.; Wang, Y. Coupling of *N*-Nosylhydrazones with Nitrosoarenes: Transition-Metal-Free Approach to (*Z*)-*N*-Arylnitrones. *Synthesis* **2018**, *50*, 1728−1736.

[7] Duguet, N.; Slawin, A. M. Z.; Smith, A. D. An Asymmetric Hetero-Claisen Approach to 3-Alkyl-3-aryloxindoles. *Org. Lett.* **2009**, *11*, 3858–3861.

[8] Zhou, Z.; Liu, G.; Chen, Y.; Lu, X. Rhodium(III)-Catalyzed Redox-Neutral C−H Annulation of Arylnitrones and Alkynes for the Synthesis of Indole Derivatives. *Adv. Synth. Catal.* **2015**, *357*, 2944–2950.

[9] Mukherjee, A.; Dateer, R. B.; Chaudhuri, R.; Bhunia, S.; Karad, S. N.; Liu, R.-S. Gold-Catalyzed 1,2-Difuntionalizations of Aminoalkynes Using Only N- and O-Containing Oxidants. *J. Am. Chem. Soc.* **2011**, *133*, 15372−15375.

[10] Tyrrell, E.; Allen, J.; Jones, K.; Beauchet, R. Asymmetric 1,3-Dipolar Cycloaddition Reactions of Nitrones with (*S*)-(-)-4-Benzyl-*N*-methacryloyl-2-oxazolidinone. *Synthesis* **2005**, *14*¸2393–2399.

[11] Röske, A.; Alt, I.; Plietker, B. Scope and Limitations of TBA[Fe]-Catalyzed Carbene Transfer to X–H-bonds–Indication of a Mechanistic Dichotomy. *ChemCatChem.* **2019**, *11*, 5260−5263.

[12] Doyle, M. P.; Yan, M.; Hu, W.; Gronenberg, L. S. Highly Selective Catalyst-Directed Pathways to Dihydropyrroles from Vinyldiazoacetates and Imines. *J. Am. Chem. Soc.* **2003**, *125*, 4692–4693.

[13] Qian, Y.; Xu, X.; Wang, X.; Zavalij, P. J.; Hu, W.; Doyle, M. P. Rhodium(II)- and Copper(II)-Catalyzed Reactions of Enol Diazoacetates with Nitrones: Metal Carbene versus Lewis Acid Directed Pathways. *Angew. Chem. Int. Ed.* **2012**, *51*, 5900–5903.

[14] Shved, A. S.; Tabolin, A. A.; Novikov, R. A.; Nelyubina, Y. V.; Timofeev, V. P.; Ioffe, S. L. Six-Membered Cyclic Nitroso Acetals: Synthesis and Studies of the Nitrogen Inversion Process of *N*-Siloxy-3,6-dihydro-2*H*-1,2-oxazines. *Eur. J. Org. Chem.* **2016**, *2016*, 5569–5578.

[15] Deng, Y.; Jing, C.; Doyle, M. P. Dinitrogen extrusion from enoldiazo compounds under thermal conditions: synthesis of donor-acceptor cyclopropenes. *Chem. Commun.* **2015**, *51*, 12924–12927.

[16] Pagar, V. V.; Liu, R.-S. Gold-Catalyzed Cycloaddition Reactions of Ethyl Diazoacetate, Nitrosoarenes, and Vinyldiazo Carbonyl Compounds: Synthesis of Isoxazolidine and Benzo[b]azepine Derivatives. *Angew. Chem. Int. Ed*. **2015**, 54, 4923–4926.
[17] Xu, X.; Ratnikov, M. O.; Zavalij, P. Y.; Doyle, M. P. Multi-functionalized 3-Hydroxypyrroles in a Three-Step, One-Pot Cascade Process from Methyl 3-TBSO-2-diazo-3-butenoate and Nitrones. *Org. Lett*. **2011**, 13, 6122–6125.
[18] SHELXL-2013, G.M. Sheldrick, University of Göttingen, Germany (2013).
[19] CrysAlisPro, Agilent Technologies, Version 1.171.37.33 (release 27-03–2014 CrysAlis 171.NET).
