# Supplementary material for: B(C6F5)3-Catalyzed Diastereoselective and Divergent Reactions of Vinyldiazo Esters with Nitrones: Synthesis of Highly Functionalized Diazo Compounds
Source: Org Lett. 2023 Jan 12;25(3):500–5. doi: 10.1021/acs.orglett.2c04198 (PMC9887602; doi:10.1021/acs.orglett.2c04198)
Supplement: Supplementary file 2 — ol2c04198_si_002.zip [file ol2c04198_si_002.zip › HMRS/3c_EC_ES.pdf]

Single Mass Analysis

Tolerance = 5.0 PPM / DBE: min = -1.5, max = 100.0
Element prediction: Off
Number of isotope peaks used for i-FIT = 3

Monoisotopic Mass, Odd and Even Electron Ions
11 formula(e) evaluated with 1 results within limits (all results (up to 1000) for each mass)
Elements Used:
C: 0-23 H: 0-22 N: 0-3 O: 0-3

Minimum:				-1.5				
Maximum:		5.0	5.0	100.0				
Mass	Calc. Mass	mDa	PPM	DBE	i-FIT	Norm	Conf(%)	Formula
388.1656	388.1661	-0.5	-1.3	14.5	1124.4	n/a	n/a	C23 H22 N3 O3
