# Supplementary material for: B(C6F5)3-Catalyzed Diastereoselective and Divergent Reactions of Vinyldiazo Esters with Nitrones: Synthesis of Highly Functionalized Diazo Compounds
Source: Org Lett. 2023 Jan 12;25(3):500–5. doi: 10.1021/acs.orglett.2c04198 (PMC9887602; doi:10.1021/acs.orglett.2c04198)
Supplement: Supplementary file 2 — ol2c04198_si_002.zip [file ol2c04198_si_002.zip › HMRS/3j_EC_ESP.pdf]

## Single Mass Analysis

Tolerance = 5.0 PPM / DBE: min = -1.5, max = 100.0
Element prediction: Off
Number of isotope peaks used for i-FIT = 3

Monoisotopic Mass, Odd and Even Electron Ions
39 formula(e) evaluated with 1 results within limits (all results (up to 1000) for each mass)
Elements Used:
C: 0-20 H: 0-22 N: 0-3 O: 0-3 Br: 0-1

| | | | | | | | | |
|---|---|---|---|---|---|---|---|---|
| Minimum: | | | | -1.5 | | | | |
| Maximum: | | 5.0 | 5.0 | 100.0 | | | | |
| Mass | Calc. Mass | mDa | PPM | DBE | i-FIT | Norm | Conf(%) | Formula |
| 352.1661 | 352.1661 | 0.0 | 0.0 | 11.5 | 587.9 | n/a | n/a | C20 H22 N3 O3 |
