# Supplementary material for: B(C6F5)3-Catalyzed Diastereoselective and Divergent Reactions of Vinyldiazo Esters with Nitrones: Synthesis of Highly Functionalized Diazo Compounds
Source: Org Lett. 2023 Jan 12;25(3):500–5. doi: 10.1021/acs.orglett.2c04198 (PMC9887602; doi:10.1021/acs.orglett.2c04198)
Supplement: Supplementary file 2 — ol2c04198_si_002.zip [file ol2c04198_si_002.zip › HMRS/5j_EC_ES.pdf]

Elemental Composition Report

Single Mass Analysis

Tolerance = 10.0 PPM / DBE: min = -1.5, max = 100.0
Element prediction: Off
Number of isotope peaks used for i-FIT = 3

Monoisotopic Mass, Odd and Even Electron Ions
74 formula(e) evaluated with 1 results within limits (up to 1 closest results for each mass)
Elements Used:
C: 0-25 H: 0-33 N: 0-3 O: 0-4 Si: 0-1 Br: 0-1

Minimum:				-1.5				
Maximum:		5.0	10.0	100.0				
Mass	Calc. Mass	mDa	PPM	DBE	i-FIT	Norm	Conf(%)	Formula
546.1425	546.1424	0.1	0.2	11.5	915.5	n/a	n/a	C25 H33 N3 O4 Si Br
