# Supplementary figures and images for: B(C6F5)3-Catalyzed Diastereoselective and Divergent Reactions of Vinyldiazo Esters with Nitrones: Synthesis of Highly Functionalized Diazo Compounds
Source: Org Lett. 2023 Jan 12;25(3):500–5. doi: 10.1021/acs.orglett.2c04198 (PMC9887602; doi:10.1021/acs.orglett.2c04198)

12-Jul-2022

KSPHPh

XEVO-G2XSQTOF#NotSet  
Cardiff University  
1: TOF MS ES+  
8.44e6

RLM\_MS39020\_ESP 8 (0.172) Cm (8-1)

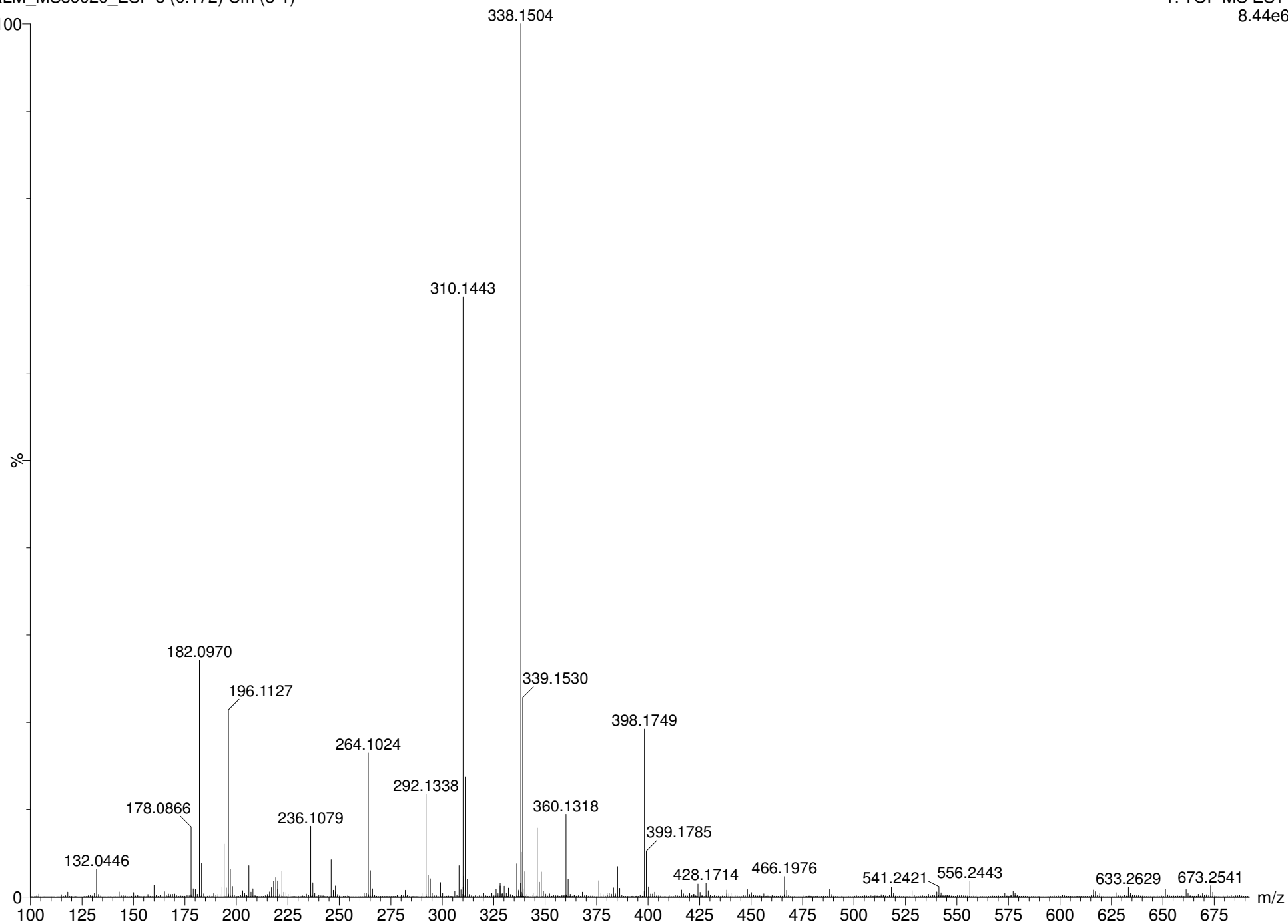

Supplement: Supplementary file 2 — ol2c04198_si_002.zip [file ol2c04198_si_002.zip › HMRS/3a_HR_ESP.pdf]

12-Jul-2022

XEVO-G2XSQTOF#NotSet  
Cardiff University  
1: TOF MS ES+  
2.69e7

RM\_MS39013\_ESP 8 (0.172) Cm (8-1)

KSNAP4H

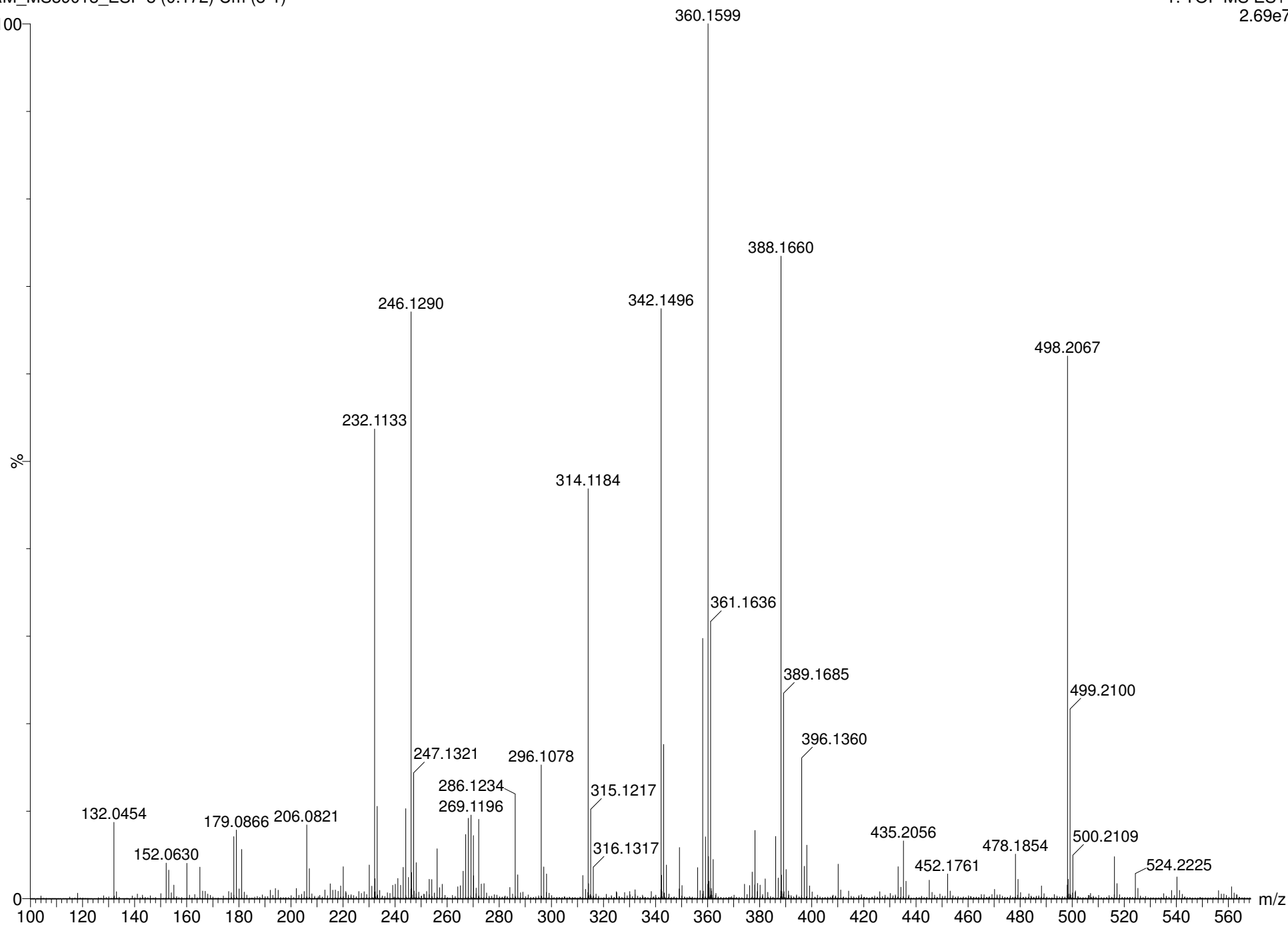

Supplement: Supplementary file 2 — ol2c04198_si_002.zip [file ol2c04198_si_002.zip › HMRS/3b_HR_ES.pdf]

12-Jul-2022

KSCF34H

XEVO-G2XSQTOF#NotSet

Cardiff University

1: TOF MS ES+

2.55e6

RM\_MS39015\_ESP 10 (0.225) Cm (10-1)

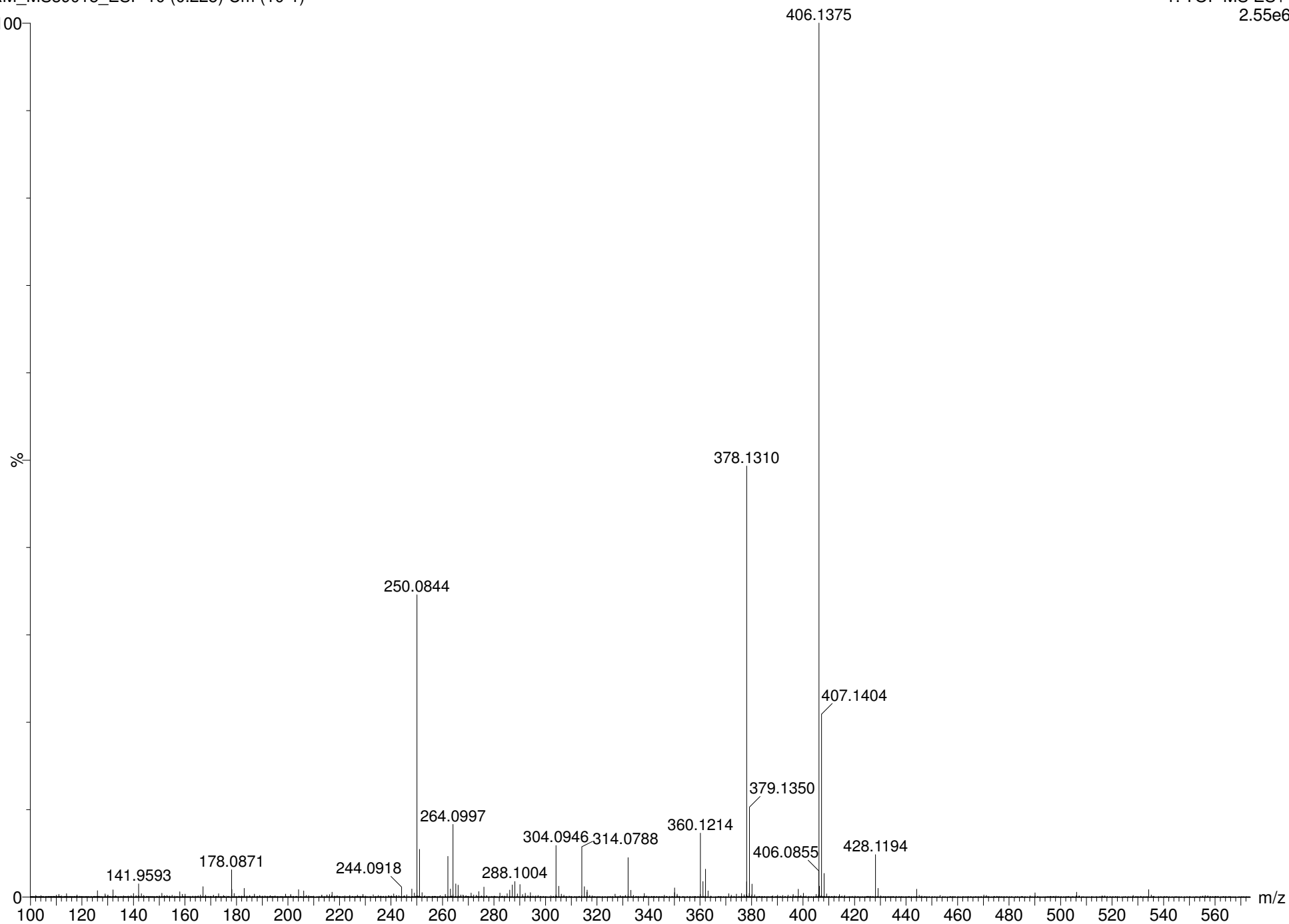

Supplement: Supplementary file 2 — ol2c04198_si_002.zip [file ol2c04198_si_002.zip › HMRS/3d_HR_ES.pdf]

13-Jul-2022

KS4F4H

XEVO-G2XSQTOF#NotSet  
Cardiff University  
1: TOF MS ES+  
2.29e6

RLM\_MS39032\_ESP 15 (0.310) Cm (15-1)

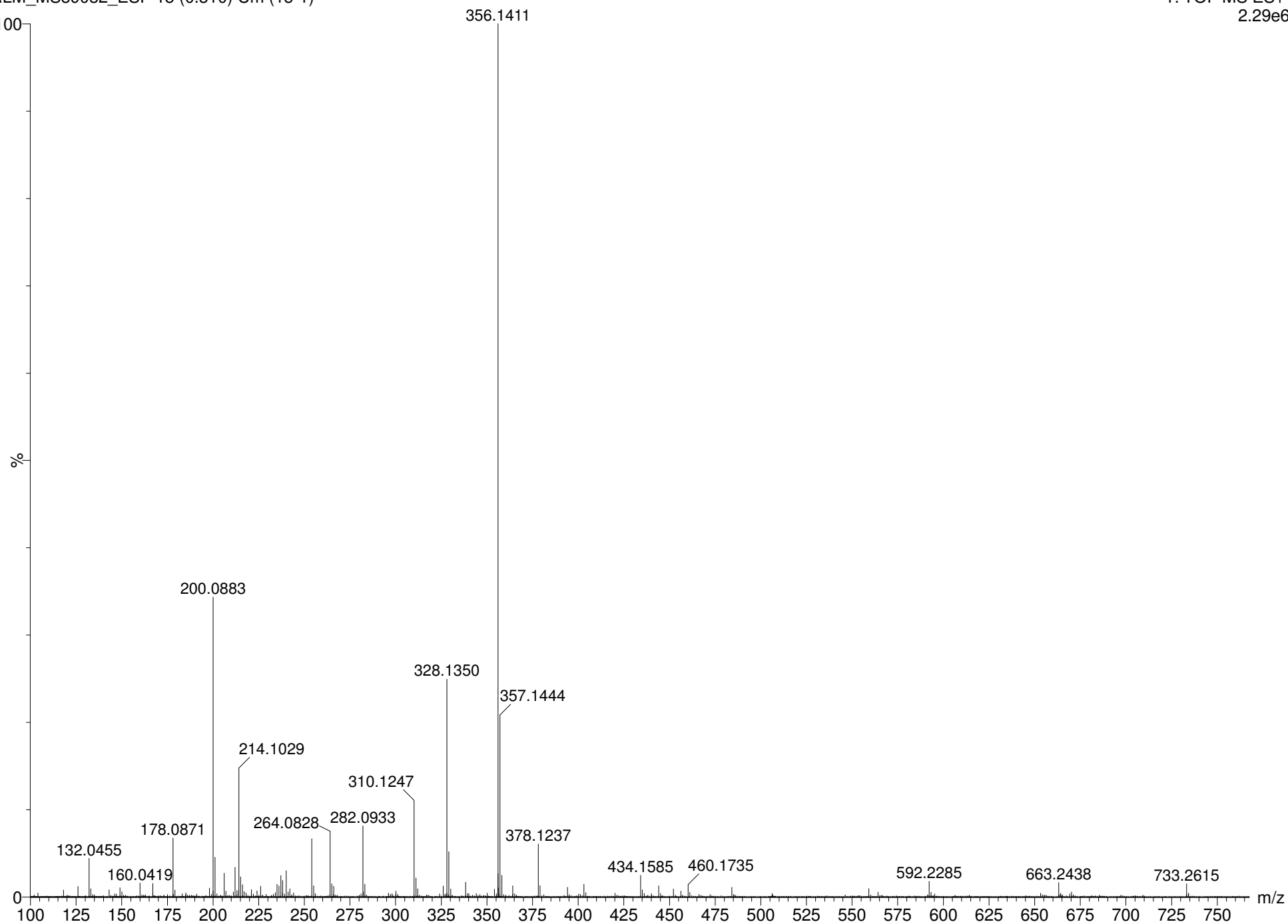

Supplement: Supplementary file 2 — ol2c04198_si_002.zip [file ol2c04198_si_002.zip › HMRS/3e_HR_ESP.pdf]

13-Jul-2022

XEVO-G2XSQTOF#NotSet

Cardiff University

1: TOF MS ES+

7.90e6

RLM\_MS39031\_ESP 13 (0.276) Cm (13-1)

KS4Cl4H

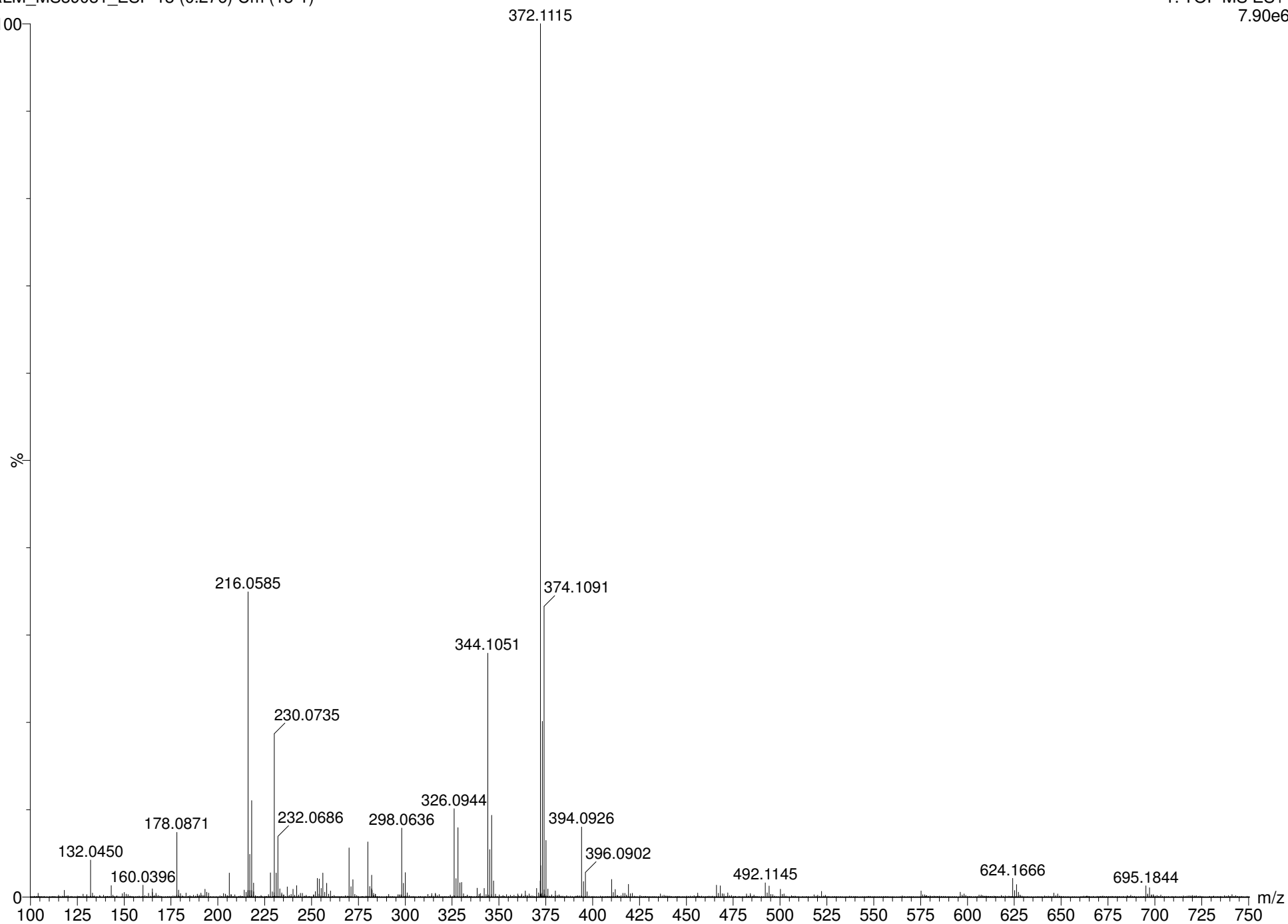

Supplement: Supplementary file 2 — ol2c04198_si_002.zip [file ol2c04198_si_002.zip › HMRS/3f_HR_ESP.pdf]

13-Jul-2022

XEVO-G2XSQTOF#NotSet  
Cardiff University  
1: TOF MS ES+  
1.29e5

RLM\_MS39033\_ESP 16 (0.327) Cm (16-1)

KS2Br4H

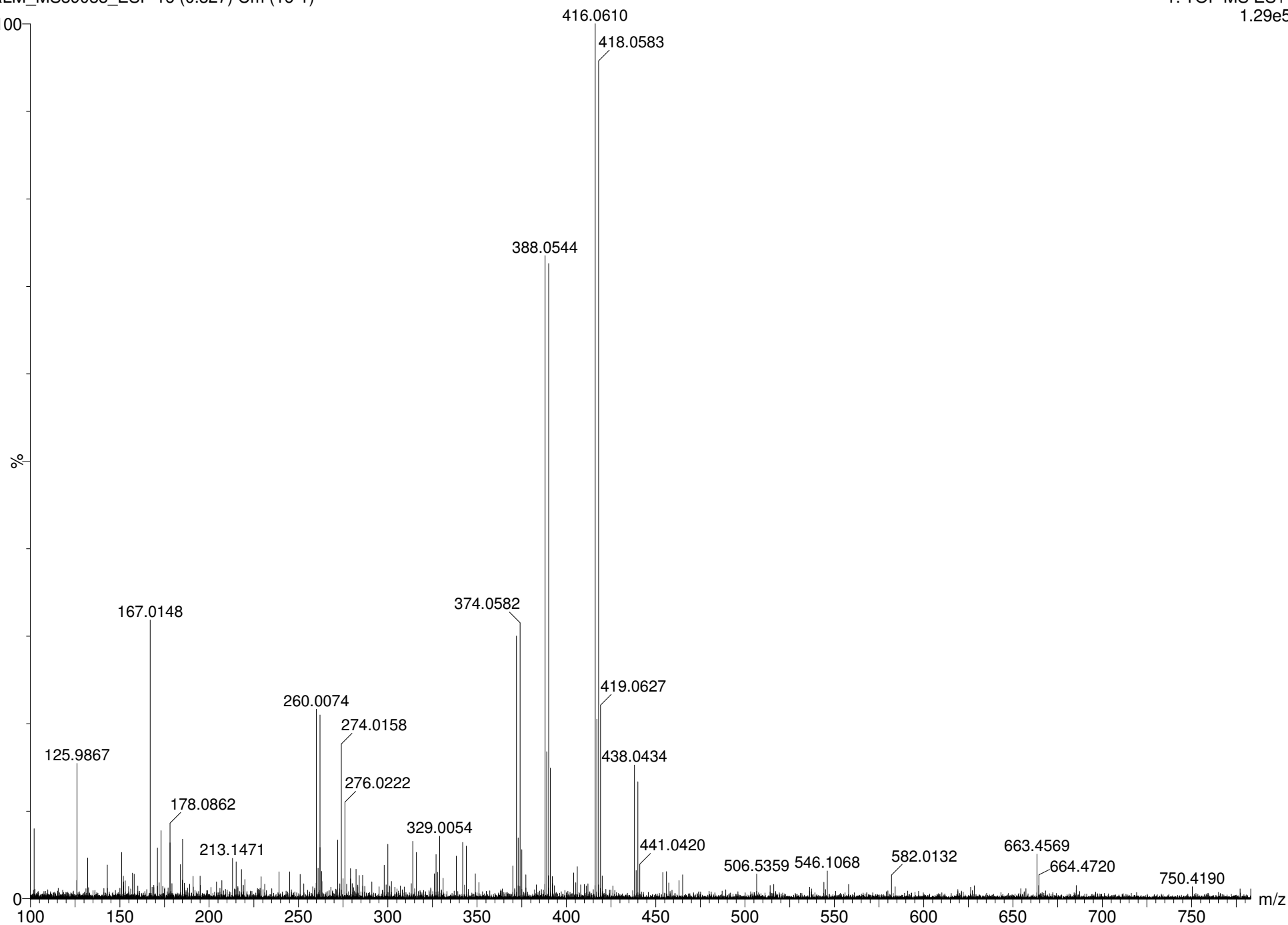

Supplement: Supplementary file 2 — ol2c04198_si_002.zip [file ol2c04198_si_002.zip › HMRS/3g_HR_ESP.pdf]

13-Jul-2022

XEVO-G2XSQTOF#NotSet  
Cardiff University  
1: TOF MS ES+  
6.23e6

RLM\_MS39029\_ESP 11 (0.242) Cm (11-1)

Ks4OMe4H

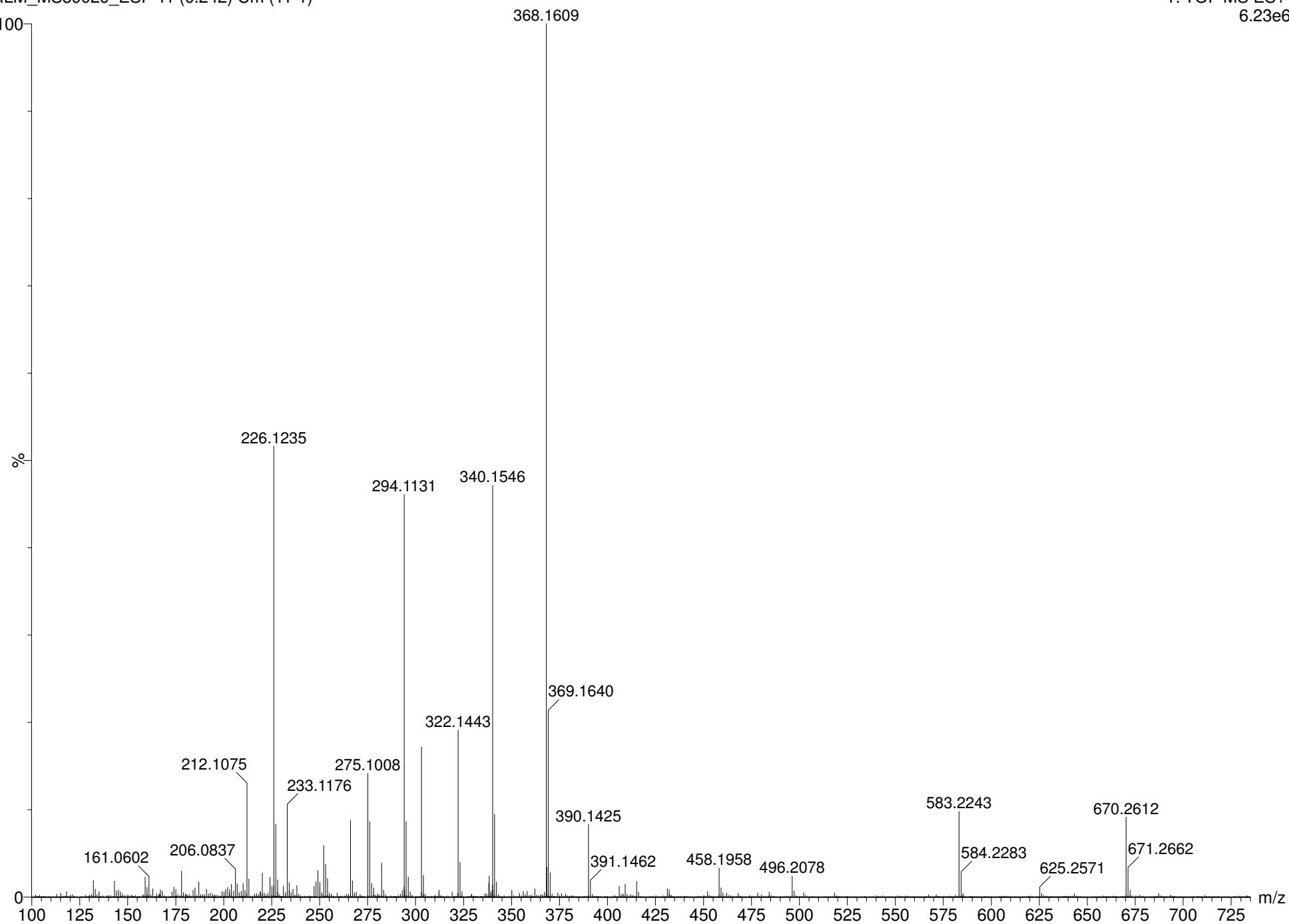

Supplement: Supplementary file 2 — ol2c04198_si_002.zip [file ol2c04198_si_002.zip › HMRS/3h_HR_ESP.pdf]

12-Jul-2022

XEVO-G2XSQTOF#NotSet  
Cardiff University  
1: TOF MS ES+  
1.20e6

RLM\_MS39023\_ESP 14 (0.293) Cm (14-1)

KSC4C14H

344.1975

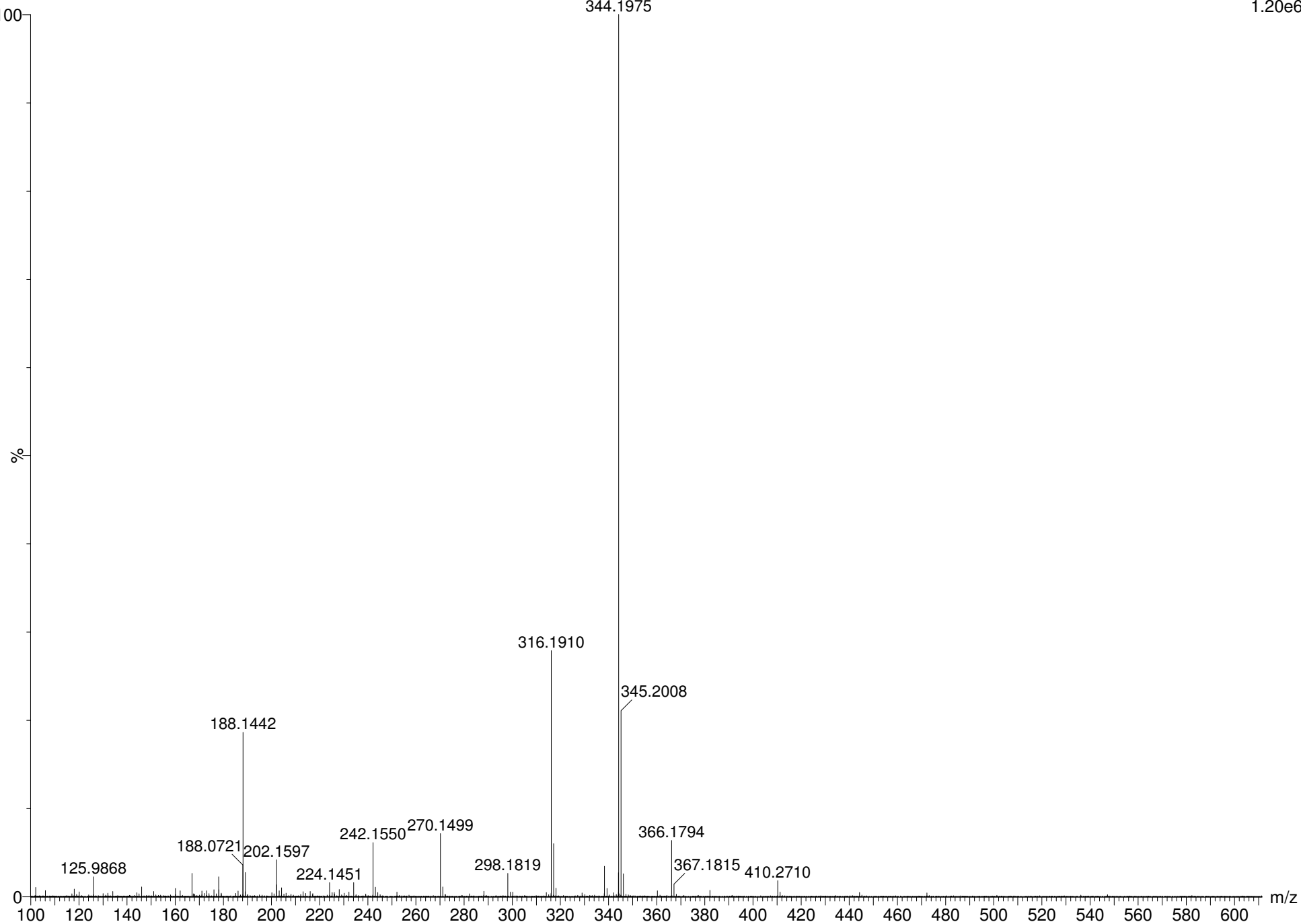

Supplement: Supplementary file 2 — ol2c04198_si_002.zip [file ol2c04198_si_002.zip › HMRS/3i_HR_ESP.pdf]

13-Jul-2022

XEVO-G2XSQTOF#NotSet  
Cardiff University  
1: TOF MS ES+  
2.81e6

RLM\_MS39028\_ESP 15 (0.310) Cm (15-1)

KS4H4Me

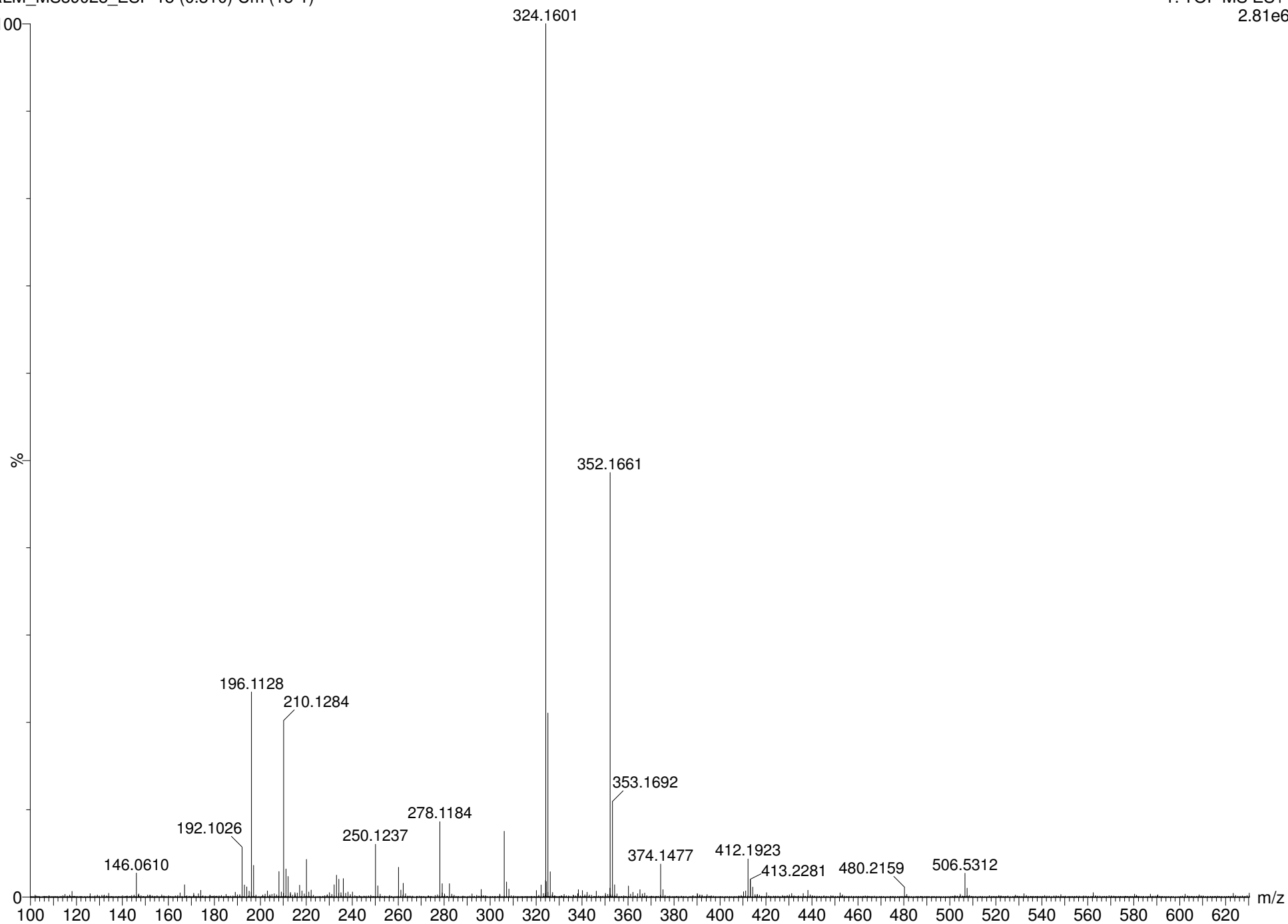

Supplement: Supplementary file 2 — ol2c04198_si_002.zip [file ol2c04198_si_002.zip › HMRS/3j_HR_ESP.pdf]

13-Jul-2022

KS4H2Me

XEVO-G2XSQTOF#NotSet  
Cardiff University  
1: TOF MS ES+  
6.32e5

RLM\_MS39027\_ESPrpt 34 (0.691) Cm (34-1)

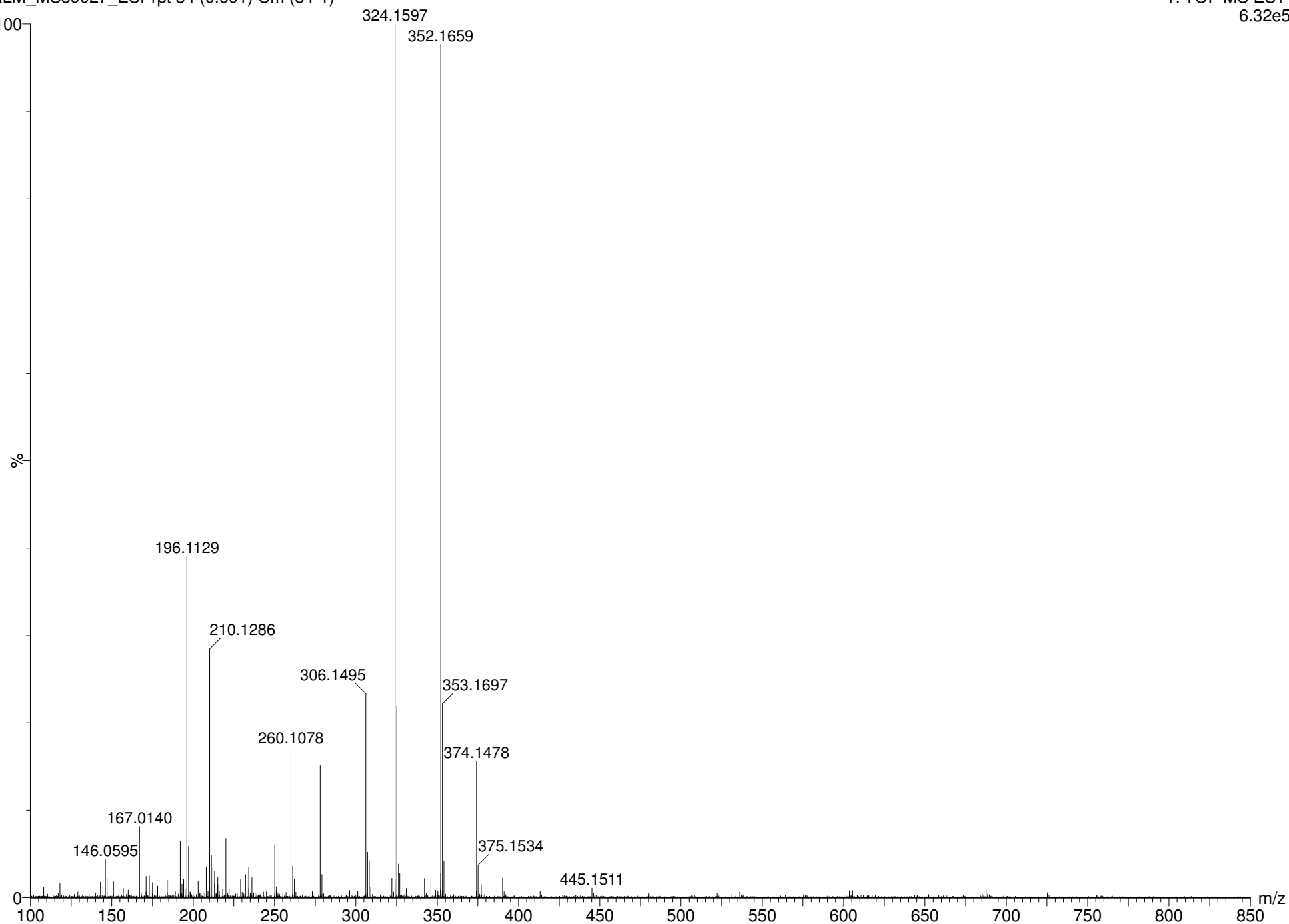

Supplement: Supplementary file 2 — ol2c04198_si_002.zip [file ol2c04198_si_002.zip › HMRS/3k_HR_ESP.pdf]

13-Jul-2022

XEVO-G2XSQTOF#NotSet

KS4H2Et

Cardiff University

RLM\_MS39030\_ESP 16 (0.327) Cm (16-1)

1: TOF MS ES+

1.69e6

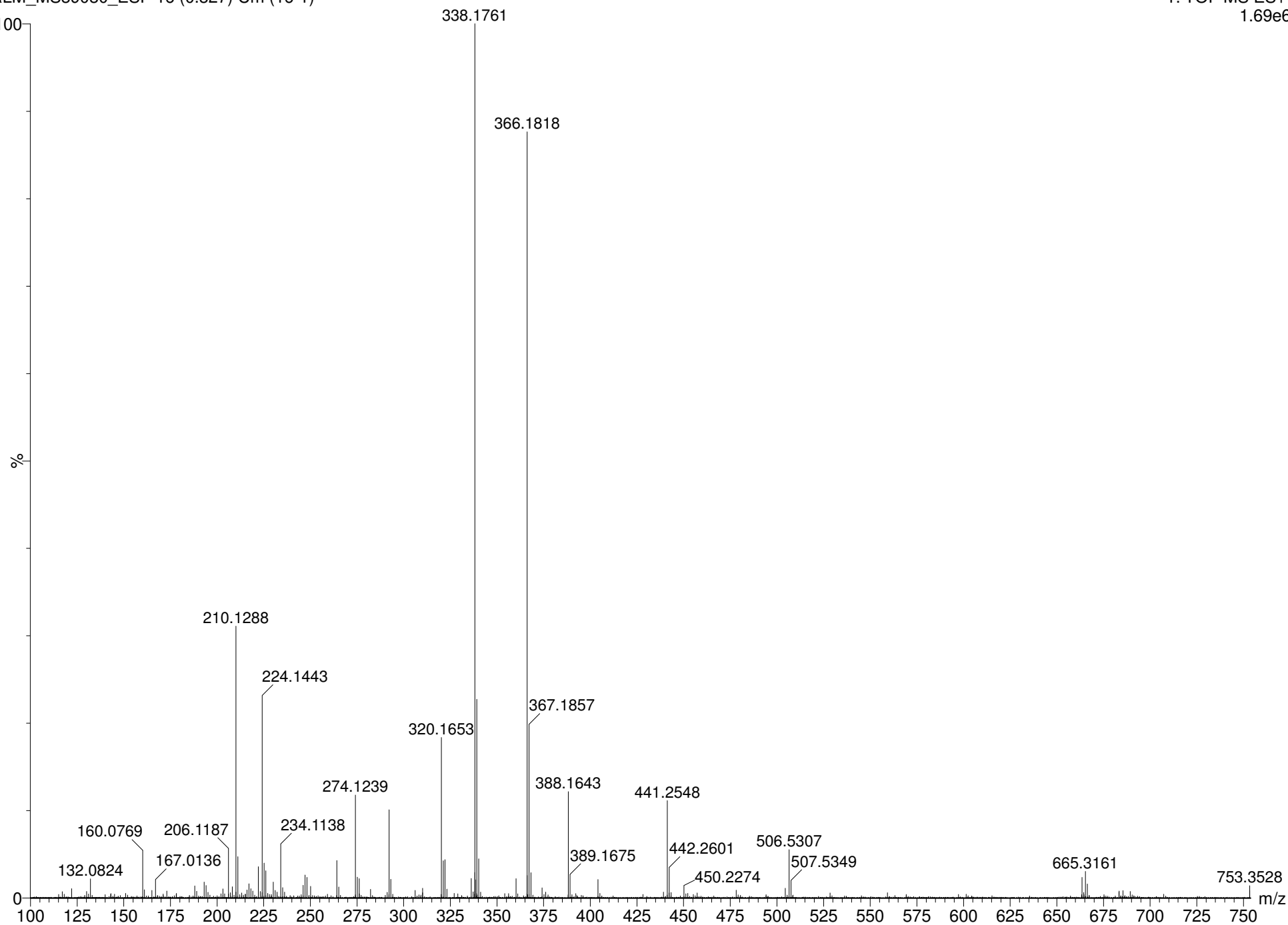

Supplement: Supplementary file 2 — ol2c04198_si_002.zip [file ol2c04198_si_002.zip › HMRS/3l_HR_ESP.pdf]

12-Jul-2022

XEVO-G2XSQTOF#NotSet  
Cardiff University  
1: TOF MS ES+  
2.79e6

RLM\_MS39022\_ESP 13 (0.276) Cm (13-1)

KS4H4F

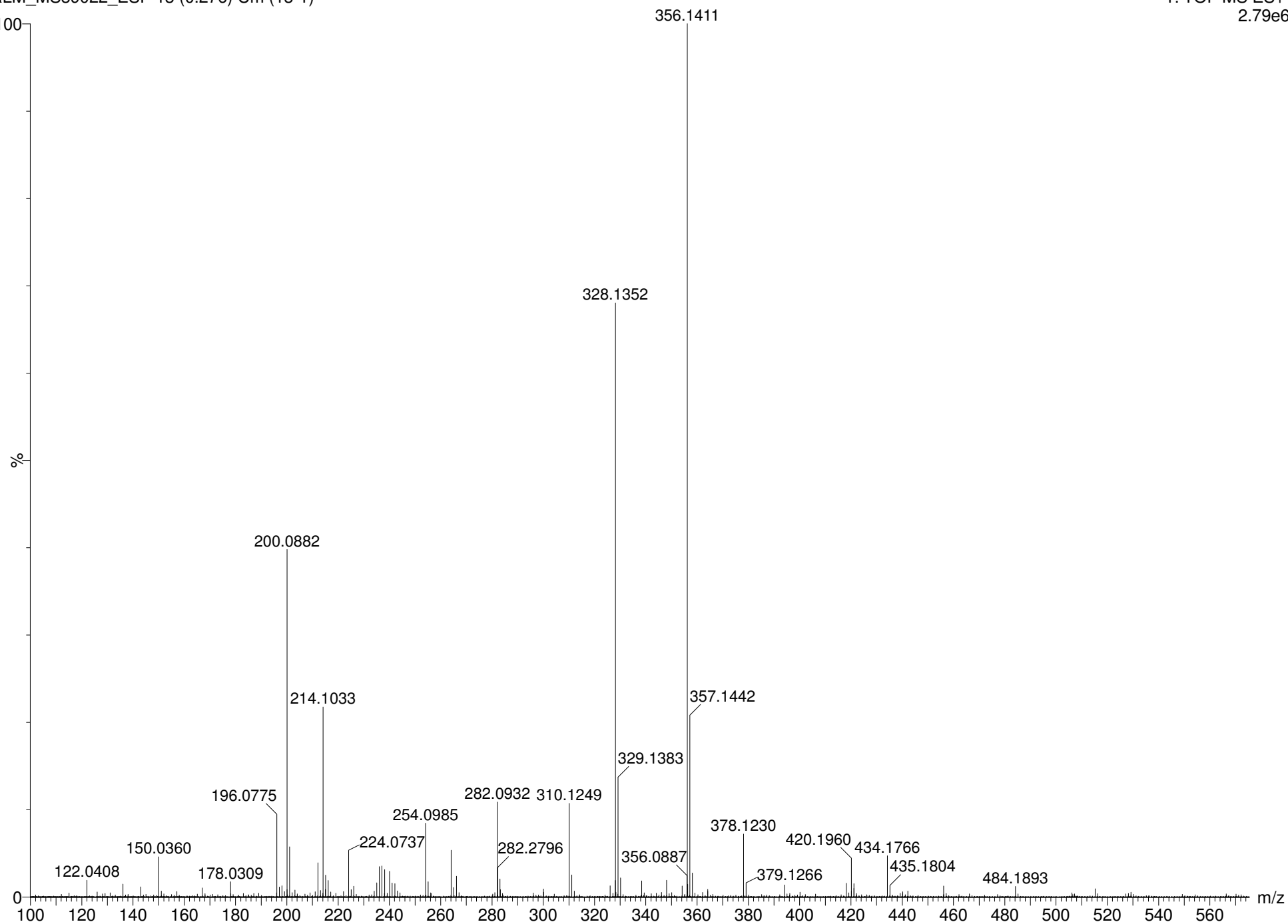

Supplement: Supplementary file 2 — ol2c04198_si_002.zip [file ol2c04198_si_002.zip › HMRS/3m_HR_ESP.pdf]

12-Jul-2022

XEVO-G2XSQTOF#NotSet  
Cardiff University  
1: TOF MS ES+  
5.60e5

RLM\_MS39024\_ESP 9 (0.209) Cm (9-1)

KS4H4I

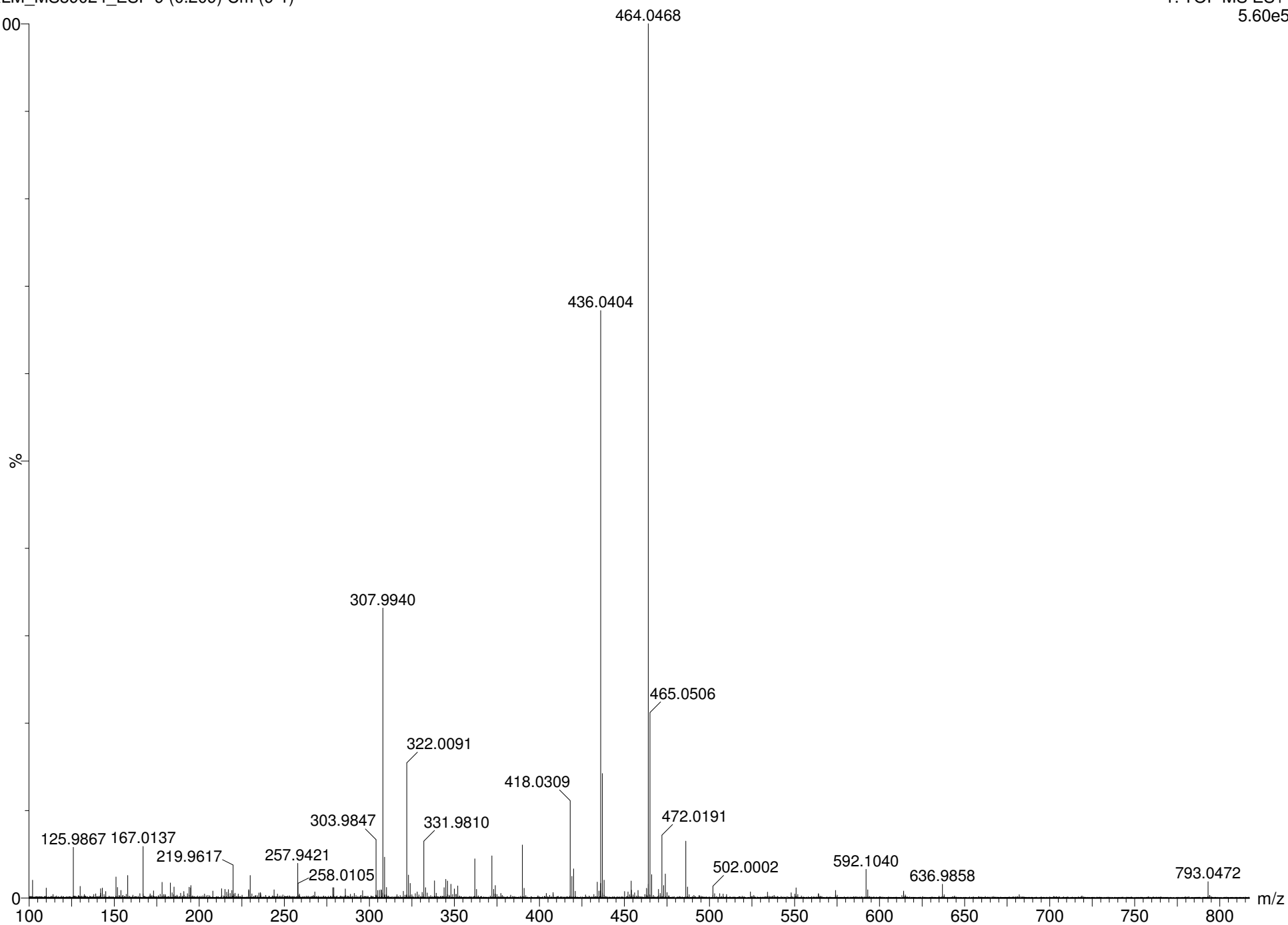

Supplement: Supplementary file 2 — ol2c04198_si_002.zip [file ol2c04198_si_002.zip › HMRS/3o_HR_ESP.pdf]

11-Nov-2022

XEVO-G2XSQTOF#NotSet  
Cardiff University  
1: TOF MS ES+  
9.29e6

RM\_MS40162\_ESP 13 (0.260)

MG328A

330.2079

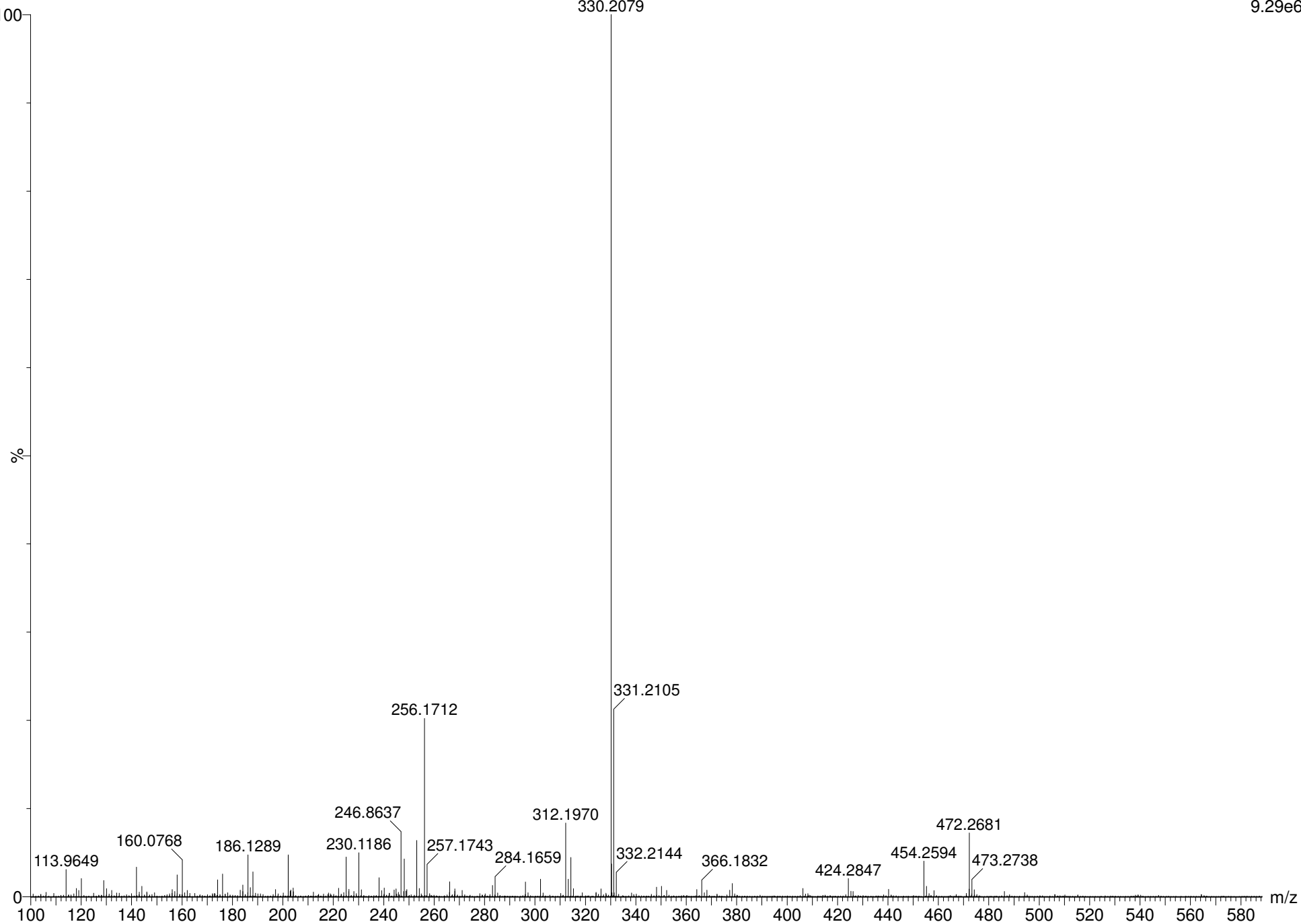

Supplement: Supplementary file 2 — ol2c04198_si_002.zip [file ol2c04198_si_002.zip › HMRS/3p_HR_ES.pdf]

01-Aug-2022

MG297A

XEVO-G2XSQTOF#NotSet  
Cardiff University  
1: TOF MS ES+  
1.88e7

RLM\_MS39133\_ESP 14 (0.293) Cm (14-1x20.000)

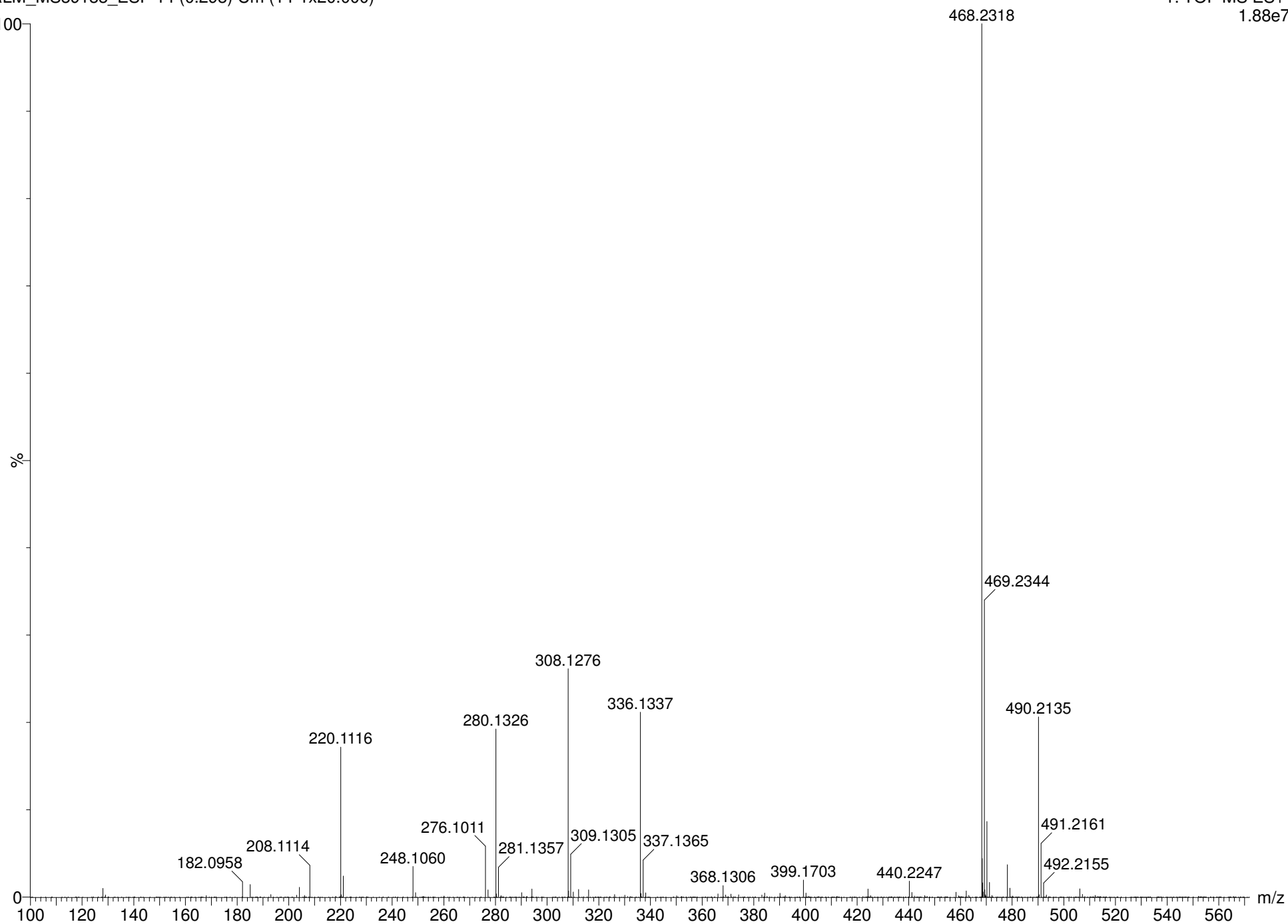

Supplement: Supplementary file 2 — ol2c04198_si_002.zip [file ol2c04198_si_002.zip › HMRS/5a_HR_ES.pdf]

01-Aug-2022

MG298A

XEVO-G2XSQTOF#NotSet  
Cardiff University  
1: TOF MS ES+  
1.14e7

RLM\_MS39139\_ESP 15 (0.310)

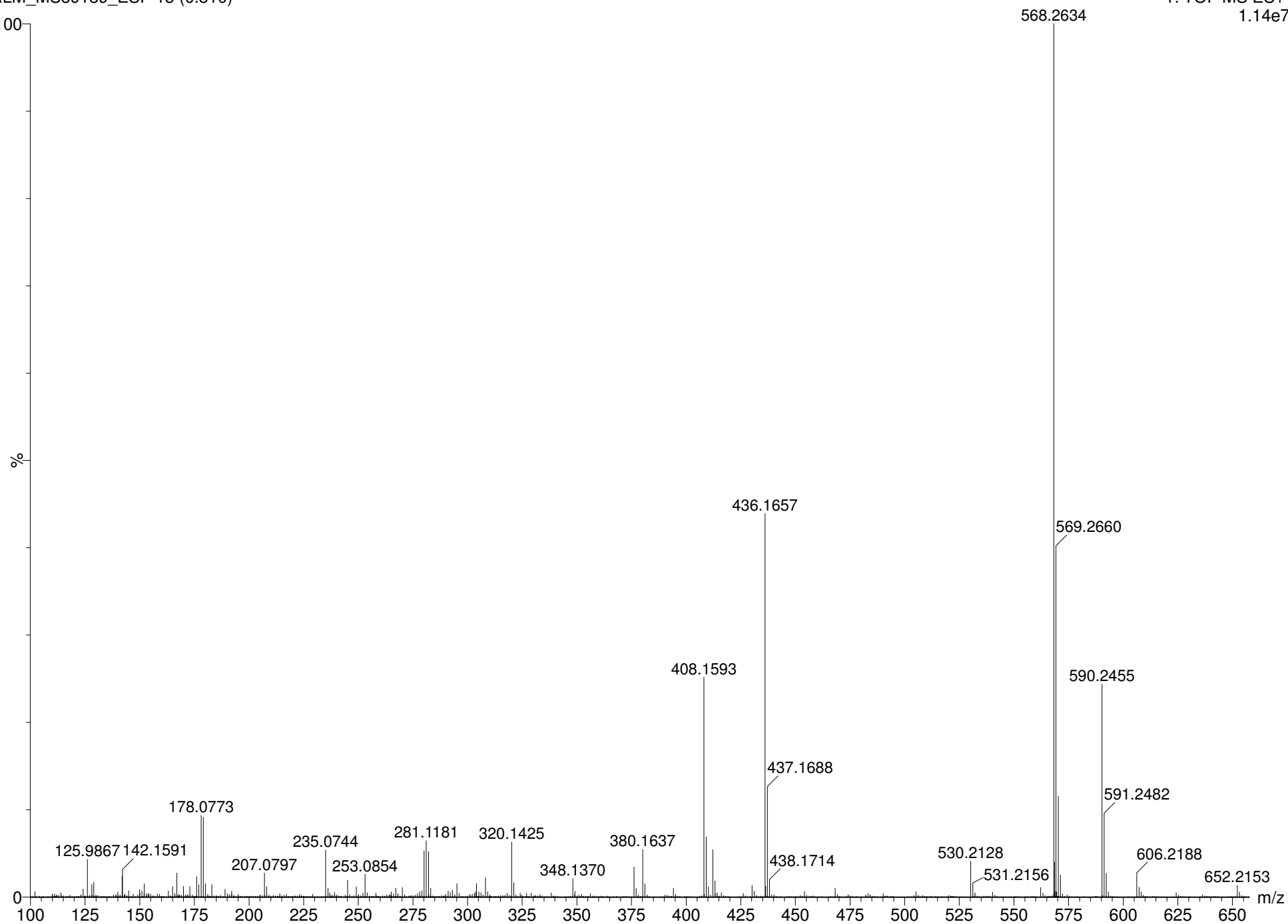

Supplement: Supplementary file 2 — ol2c04198_si_002.zip [file ol2c04198_si_002.zip › HMRS/5b_HR_ES.pdf]

01-Aug-2022

MG299A

XEVO-G2XSQTOF#NotSet  
Cardiff University  
1: TOF MS ES+  
8.86e6

RLM\_MS39140\_ESP 16 (0.327) Cm (16-1x20.000)

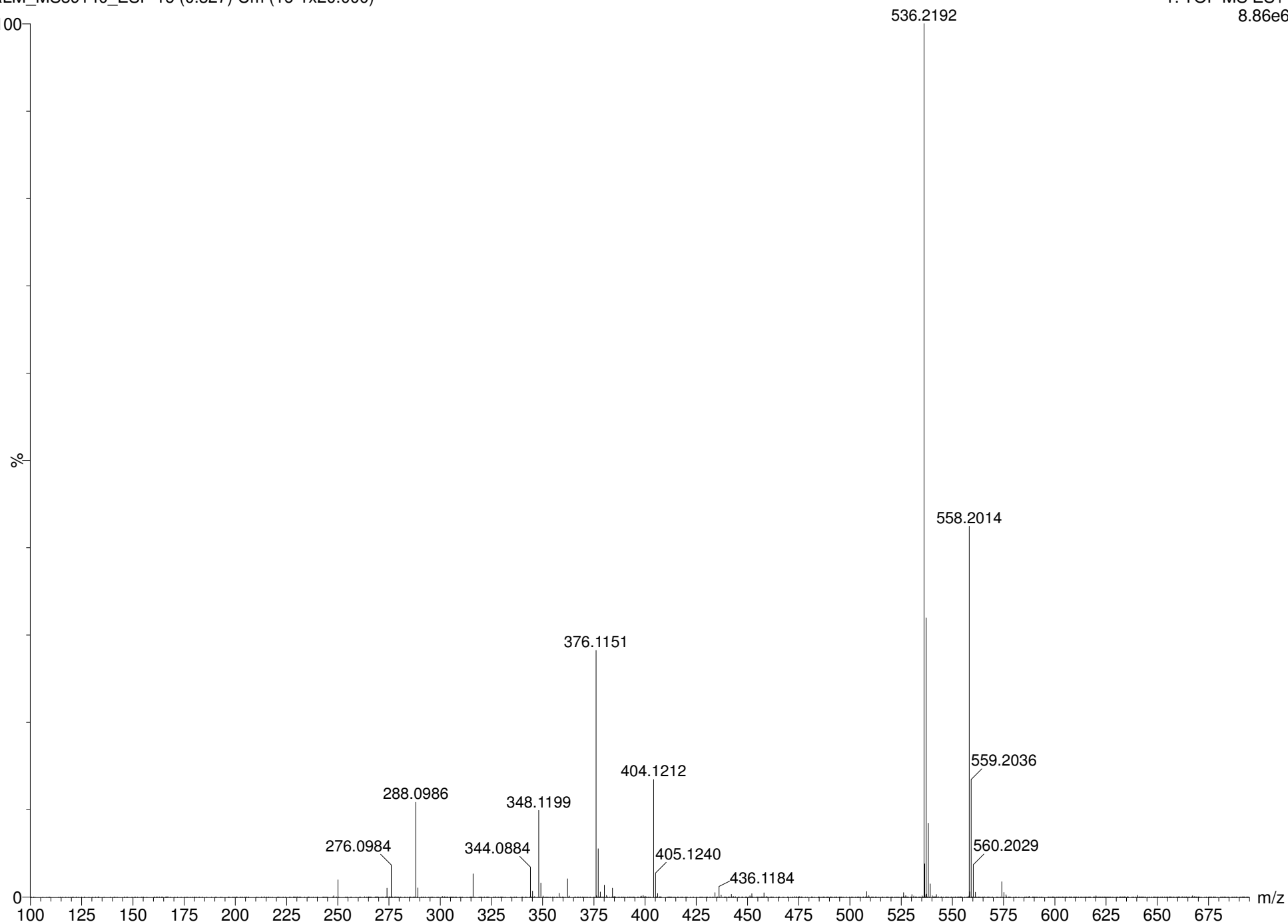

Supplement: Supplementary file 2 — ol2c04198_si_002.zip [file ol2c04198_si_002.zip › HMRS/5c_HR_ES.pdf]

01-Aug-2022

MG308A

XEVO-G2XSQTOF#NotSet  
Cardiff University  
1: TOF MS ES+  
1.89e7

RLM\_MS39136\_ESP 11 (0.242) Cm (11-1x20.000)

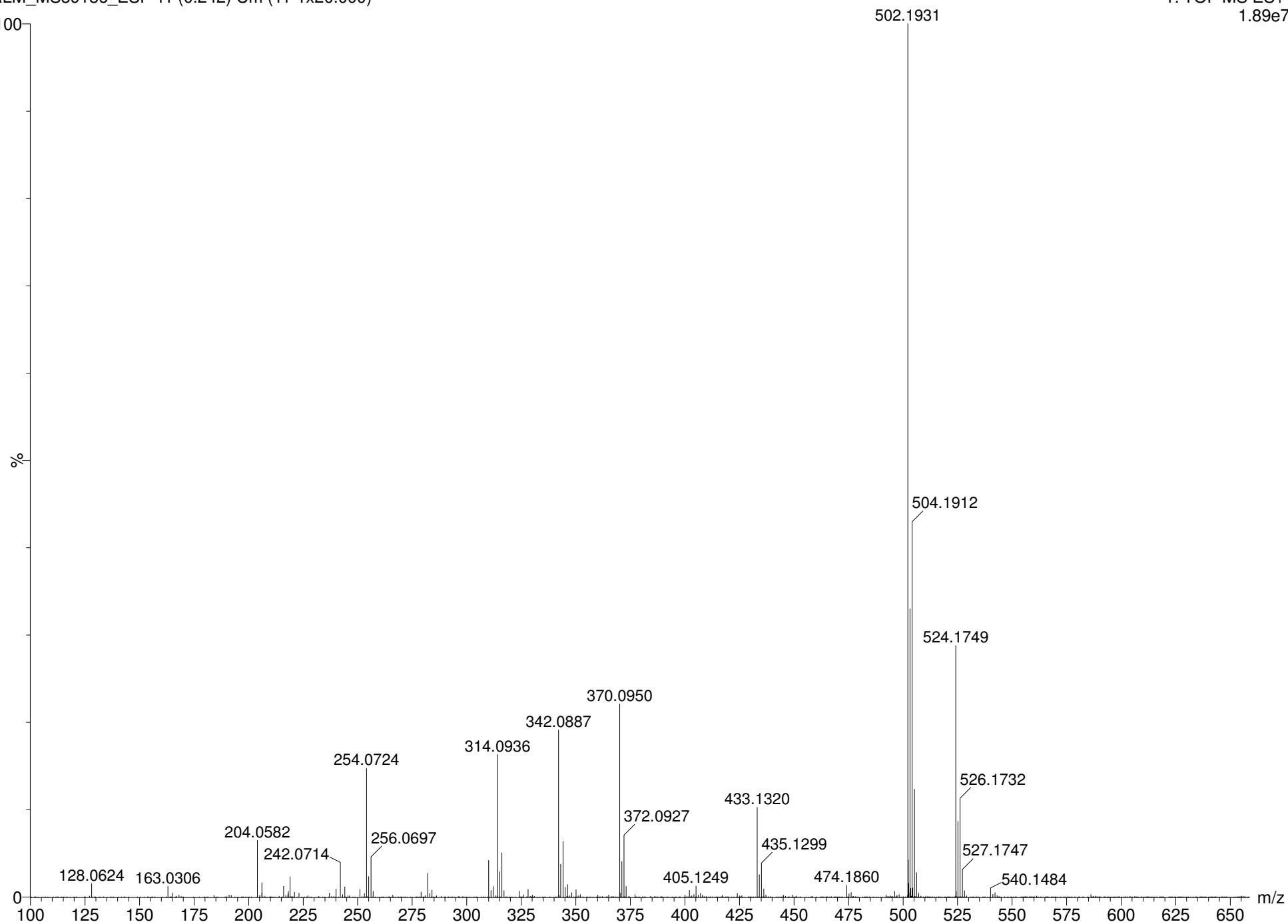

Supplement: Supplementary file 2 — ol2c04198_si_002.zip [file ol2c04198_si_002.zip › HMRS/5e_HR_ES.pdf]

01-Aug-2022

MG291A

XEVO-G2XSQTOF#NotSet  
Cardiff University  
1: TOF MS ES+  
6.94e6

RLM\_MS39134\_ESP 7 (0.155) Cm (7-1x20.000)

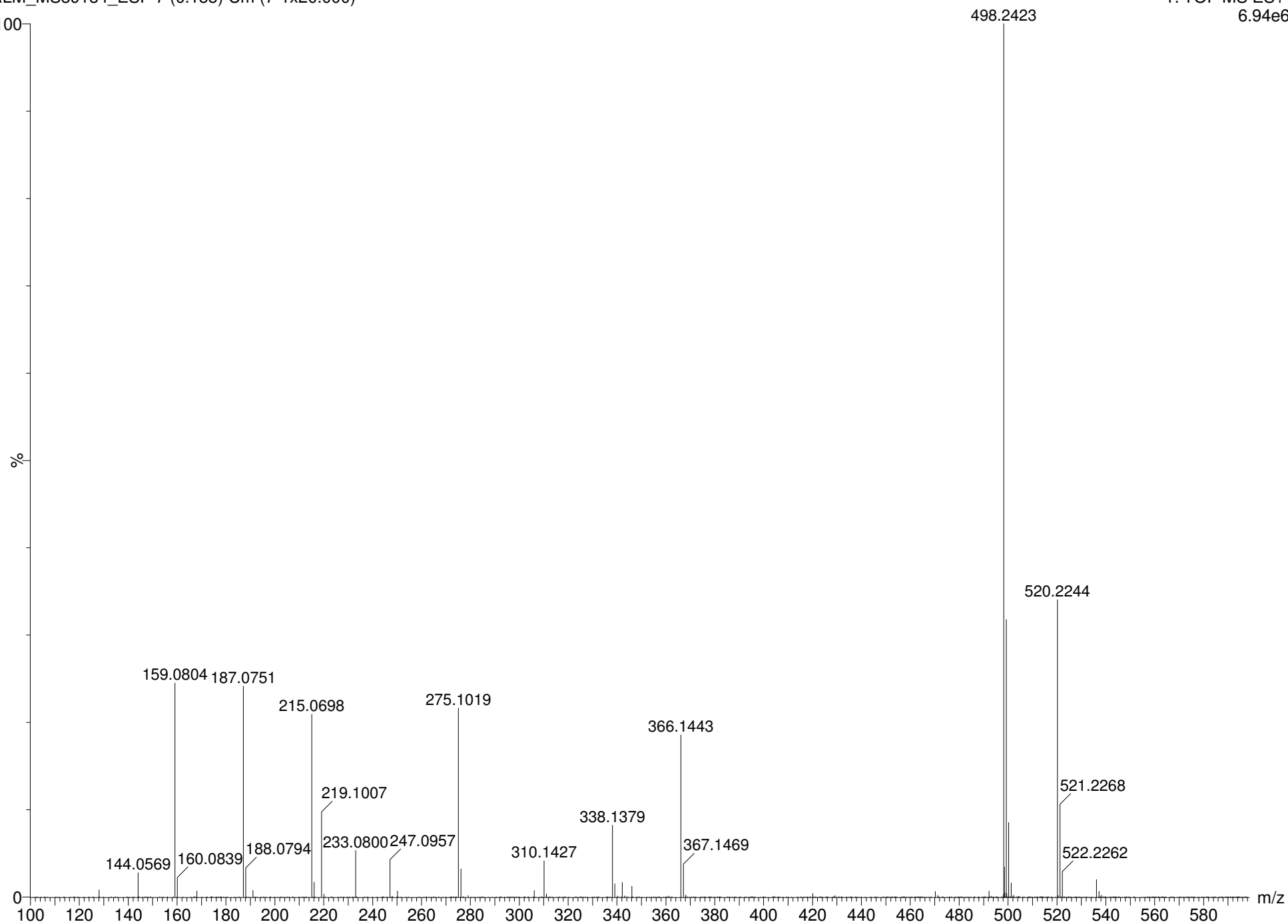

Supplement: Supplementary file 2 — ol2c04198_si_002.zip [file ol2c04198_si_002.zip › HMRS/5f_HR_ES.pdf]

01-Aug-2022

RLM\_MS39135\_ESP 10 (0.226) Cm (10-1x20.000)

MG296A

XEVO-G2XSQTOF#NotSet  
Cardiff University  
1: TOF MS ES+  
1.99e7

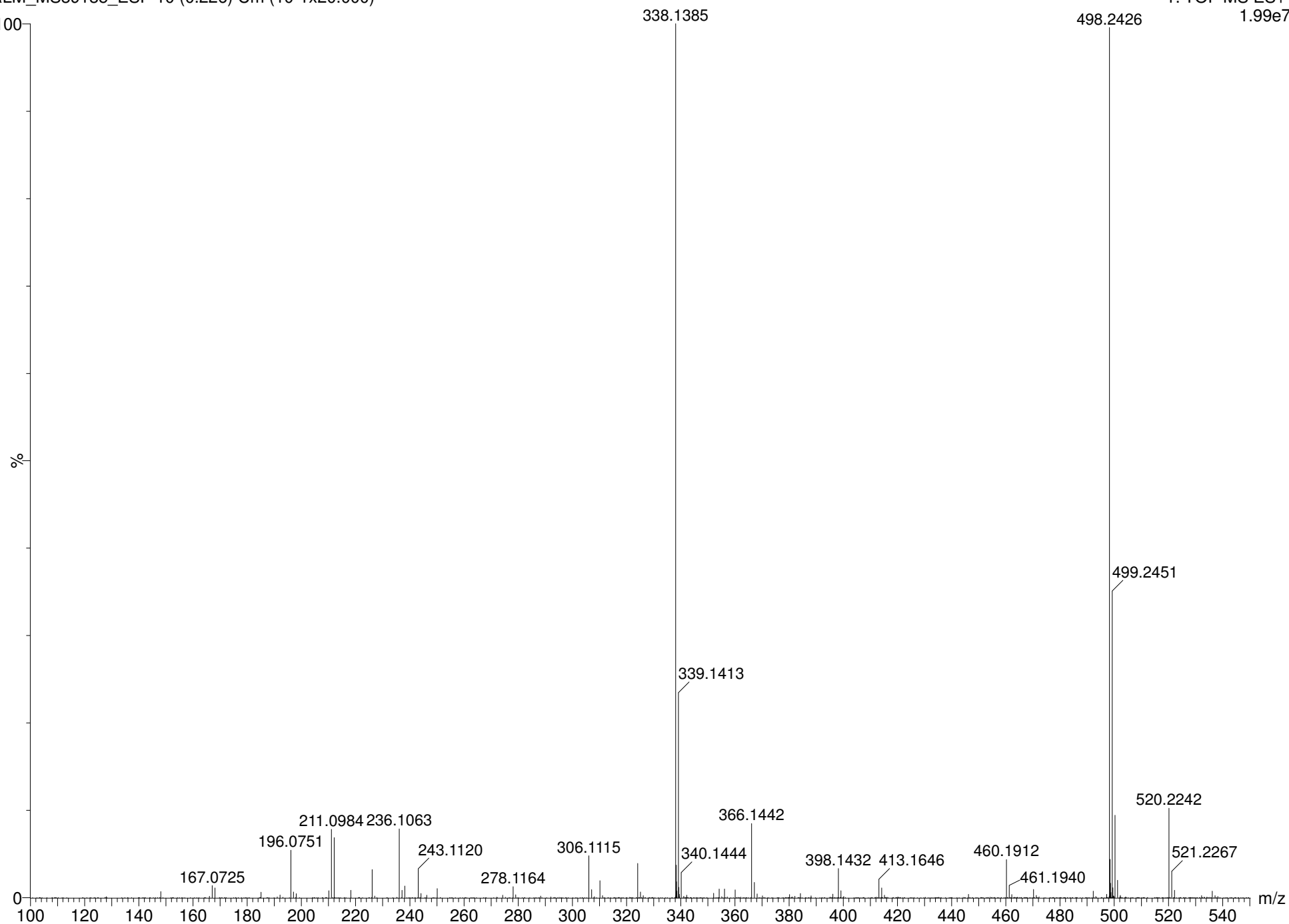

Supplement: Supplementary file 2 — ol2c04198_si_002.zip [file ol2c04198_si_002.zip › HMRS/5g_HR_ES.pdf]

01-Aug-2022

MG300A

XEVO-G2XSQTOF#NotSet  
Cardiff University  
1: TOF MS ES+  
2.38e7

RLM\_MS39137\_ESP 8 (0.172) Cm (8-1x20.000)

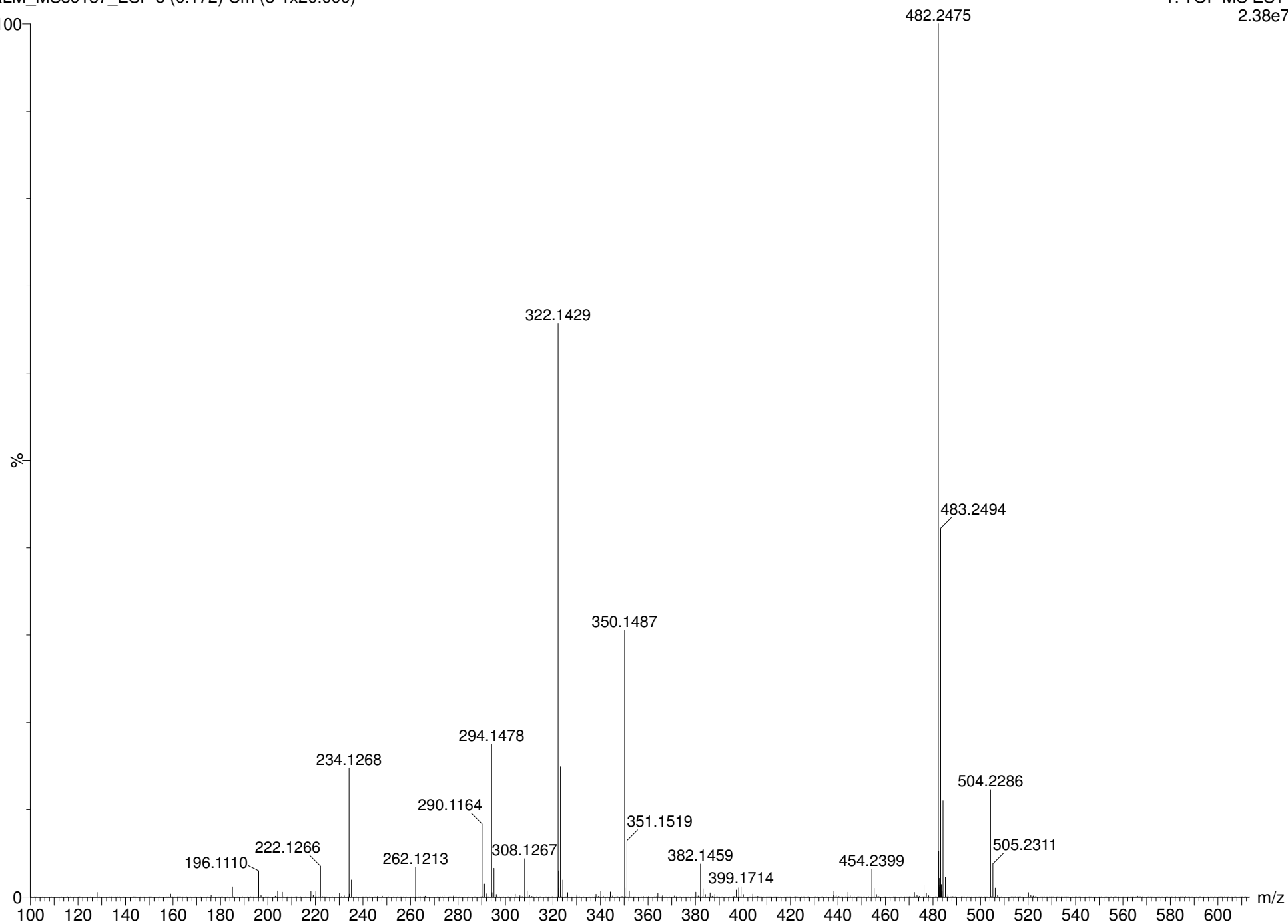

Supplement: Supplementary file 2 — ol2c04198_si_002.zip [file ol2c04198_si_002.zip › HMRS/5h_HR_ES.pdf]

01-Aug-2022

MG295A

XEVO-G2XSQTOF#NotSet

Cardiff University

1: TOF MS ES+

3.12e6

RLM\_MS39145\_ESP 17 (0.364) Cm (17-1x20.000)

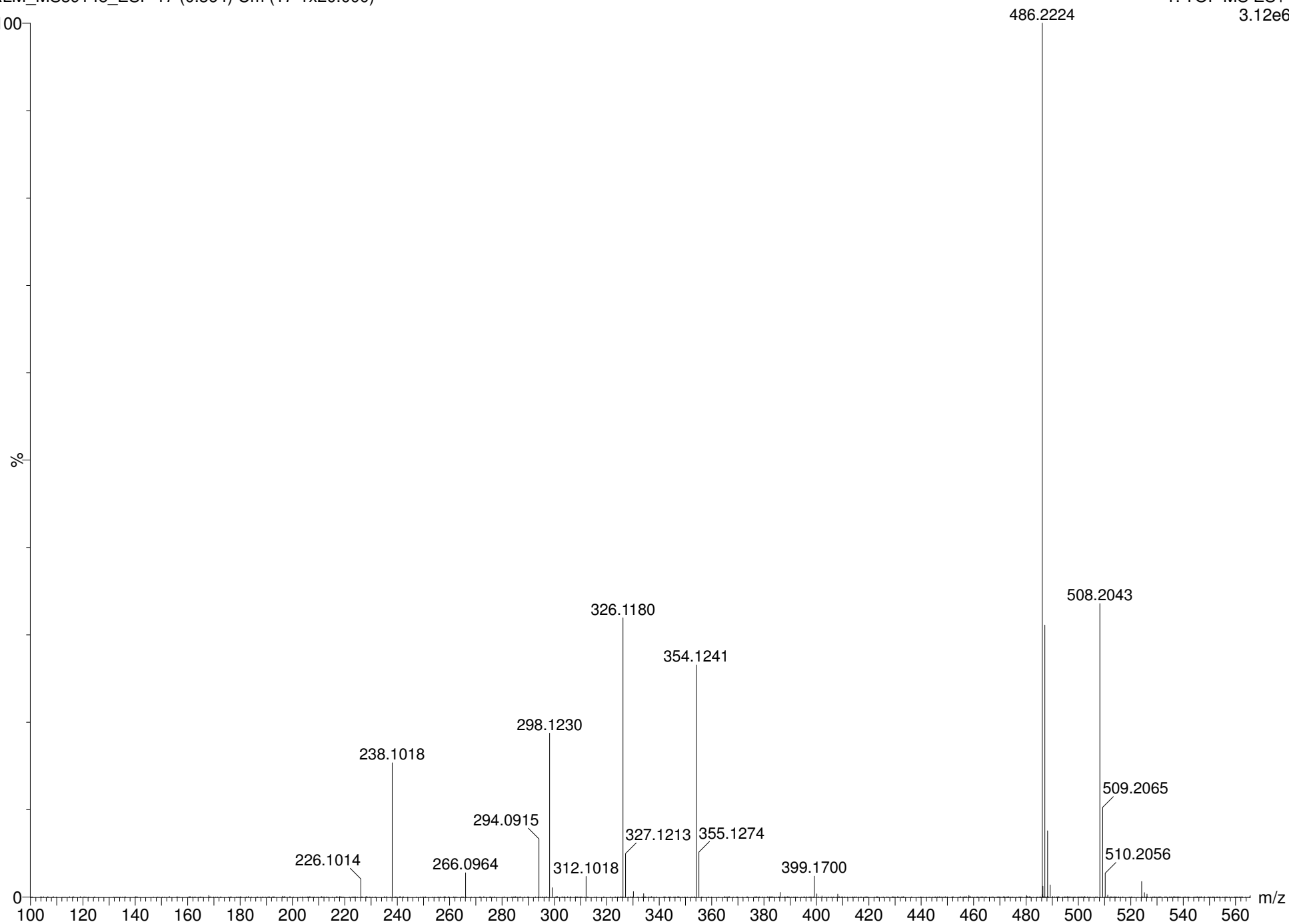

Supplement: Supplementary file 2 — ol2c04198_si_002.zip [file ol2c04198_si_002.zip › HMRS/5i_HR_ES.pdf]

01-Aug-2022

MG314A

XEVO-G2XSQTOF#NotSet  
Cardiff University  
1: TOF MS ES+  
8.78e5

RLM\_MS39142\_ESP 17 (0.364) Cm (17-1x20.000)

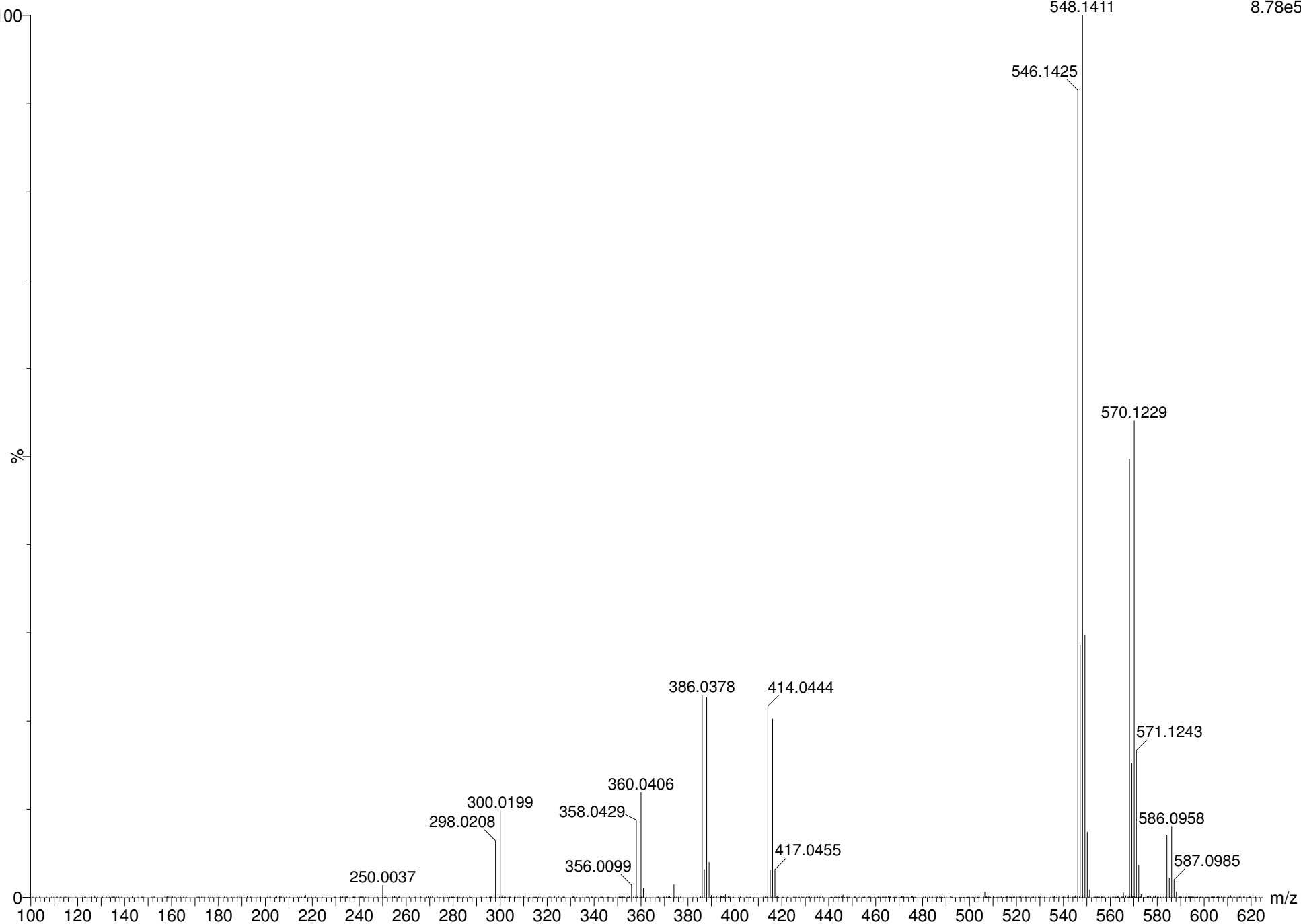

Supplement: Supplementary file 2 — ol2c04198_si_002.zip [file ol2c04198_si_002.zip › HMRS/5j_HR_ES.pdf]

01-Aug-2022

MG313A

XEVO-G2XSQTOF#NotSet  
Cardiff University  
1: TOF MS ES+  
6.36e6

RLM\_MS39141\_ESP 16 (0.327)

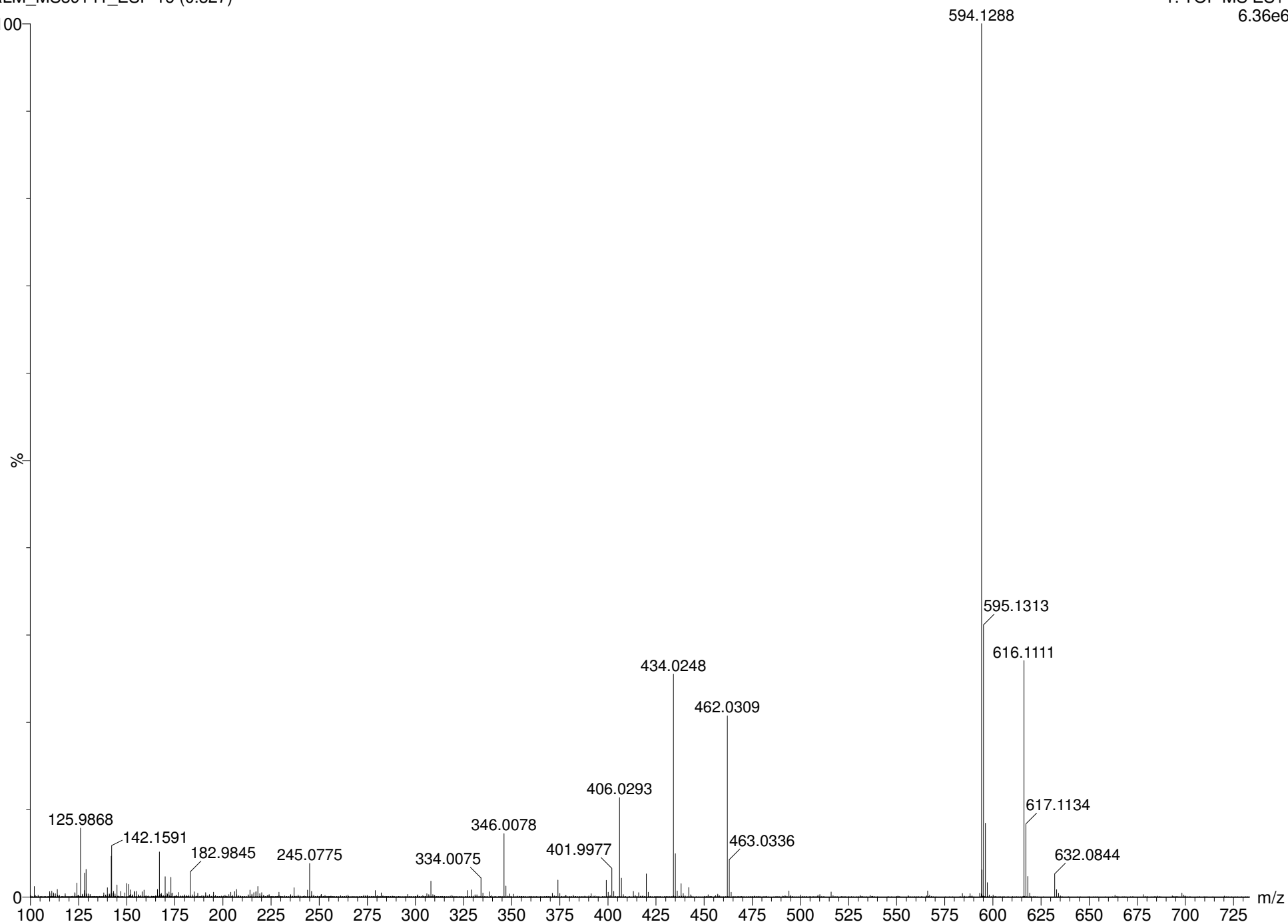

Supplement: Supplementary file 2 — ol2c04198_si_002.zip [file ol2c04198_si_002.zip › HMRS/5k_HR_ES.pdf]

01-Aug-2022

XEVO-G2XSQTOF#NotSet  
Cardiff University  
1: TOF MS ES+  
1.43e7

RLM\_MS39147\_ESP 16 (0.327) Cm (16-1x20.000)

MG260A

486.2222

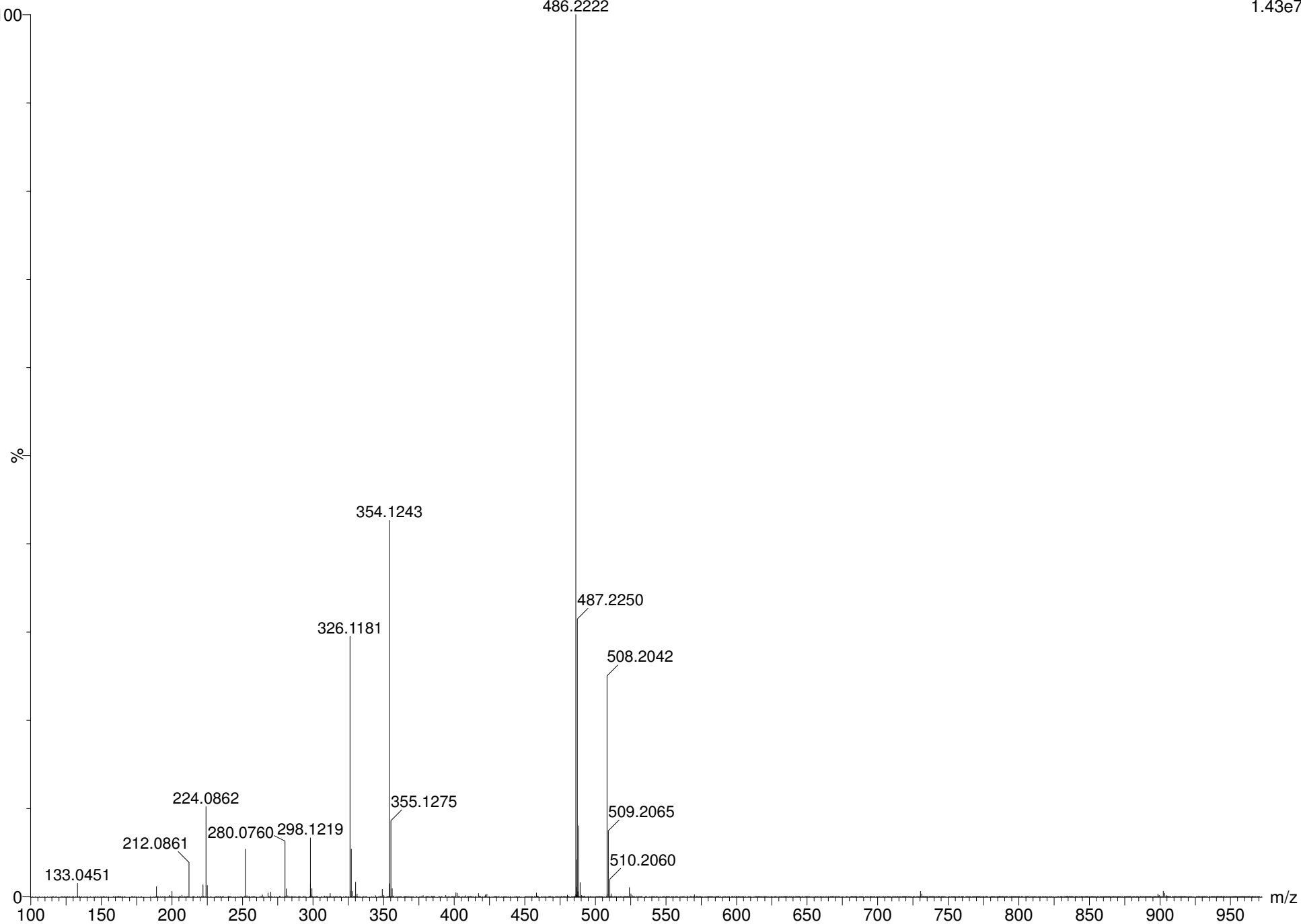

Supplement: Supplementary file 2 — ol2c04198_si_002.zip [file ol2c04198_si_002.zip › HMRS/5l_HR_ESP.pdf]

01-Aug-2022

MG279A4

XEVO-G2XSQTOF#NotSet  
Cardiff University  
1: TOF MS ES+  
1.18e7

RLM\_MS39146\_ESP 8 (0.172) Cm (8-1x20.000)

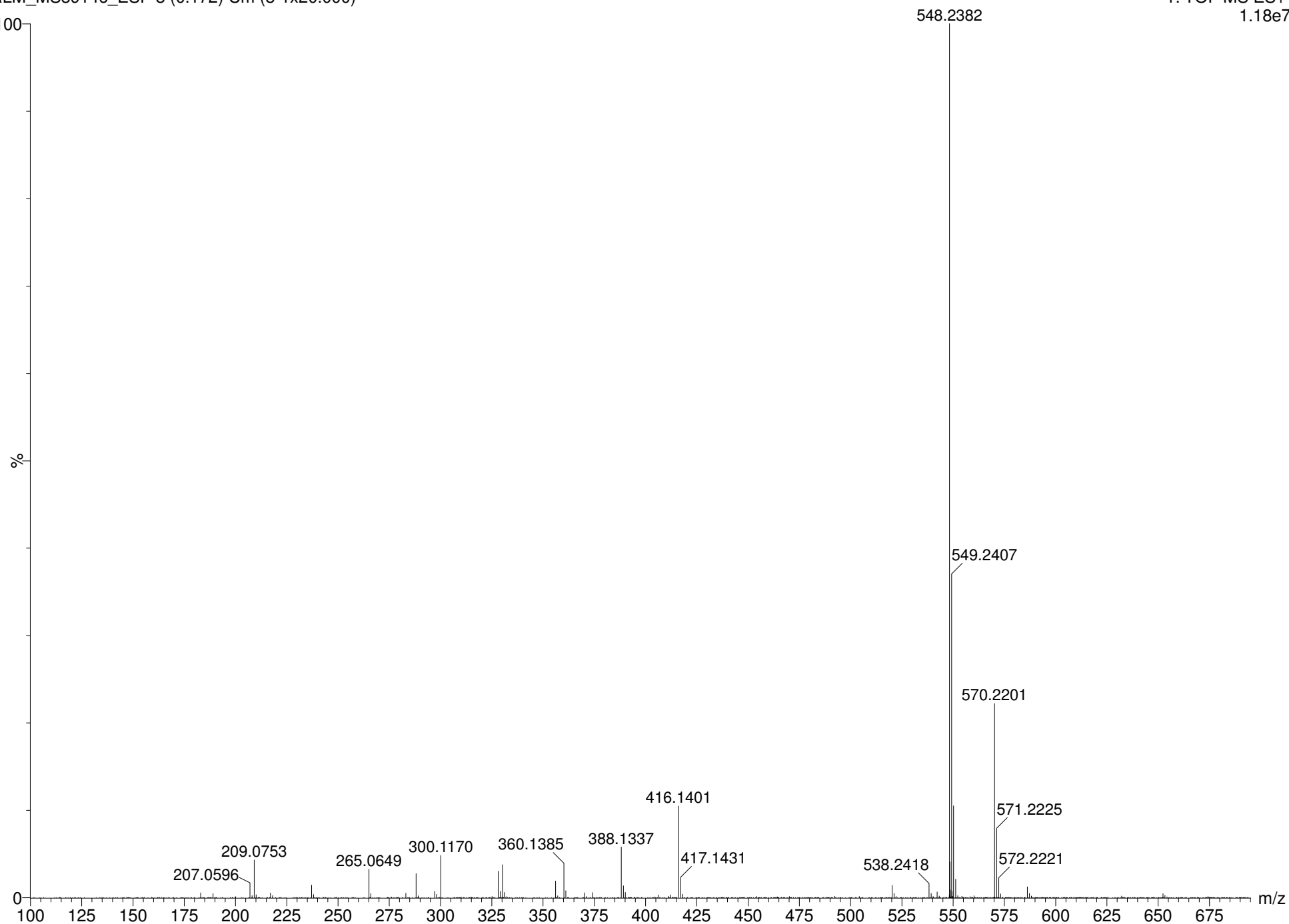

Supplement: Supplementary file 2 — ol2c04198_si_002.zip [file ol2c04198_si_002.zip › HMRS/5m_HR_ES.pdf]

16-Nov-2022

MG362A

XEVO-G2XSQTOF#NotSet  
Cardiff University  
1: TOF MS ES+  
1.29e6

RLM\_MS40202\_ESP 15 (0.293) Cm (15-1)

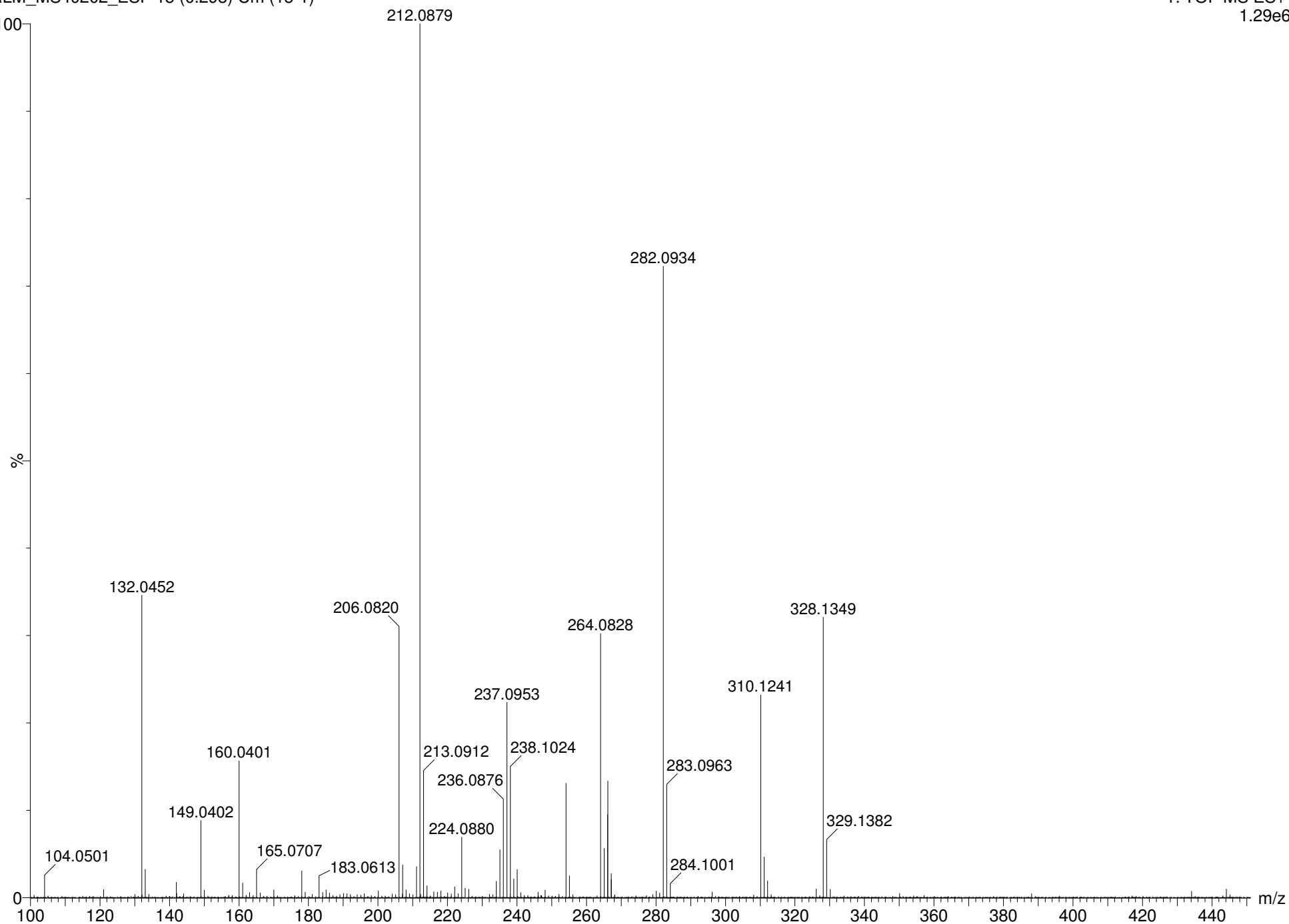

Supplement: Supplementary file 2 — ol2c04198_si_002.zip [file ol2c04198_si_002.zip › HMRS/6a_HR_ESP.pdf]

04-Nov-2022

MG369D

XEVO-G2XSQTOF#NotSet  
Cardiff University  
1: TOF MS ES+  
1.48e7

RLM\_MS40114\_ESP 10 (0.209) Cm (10-1)

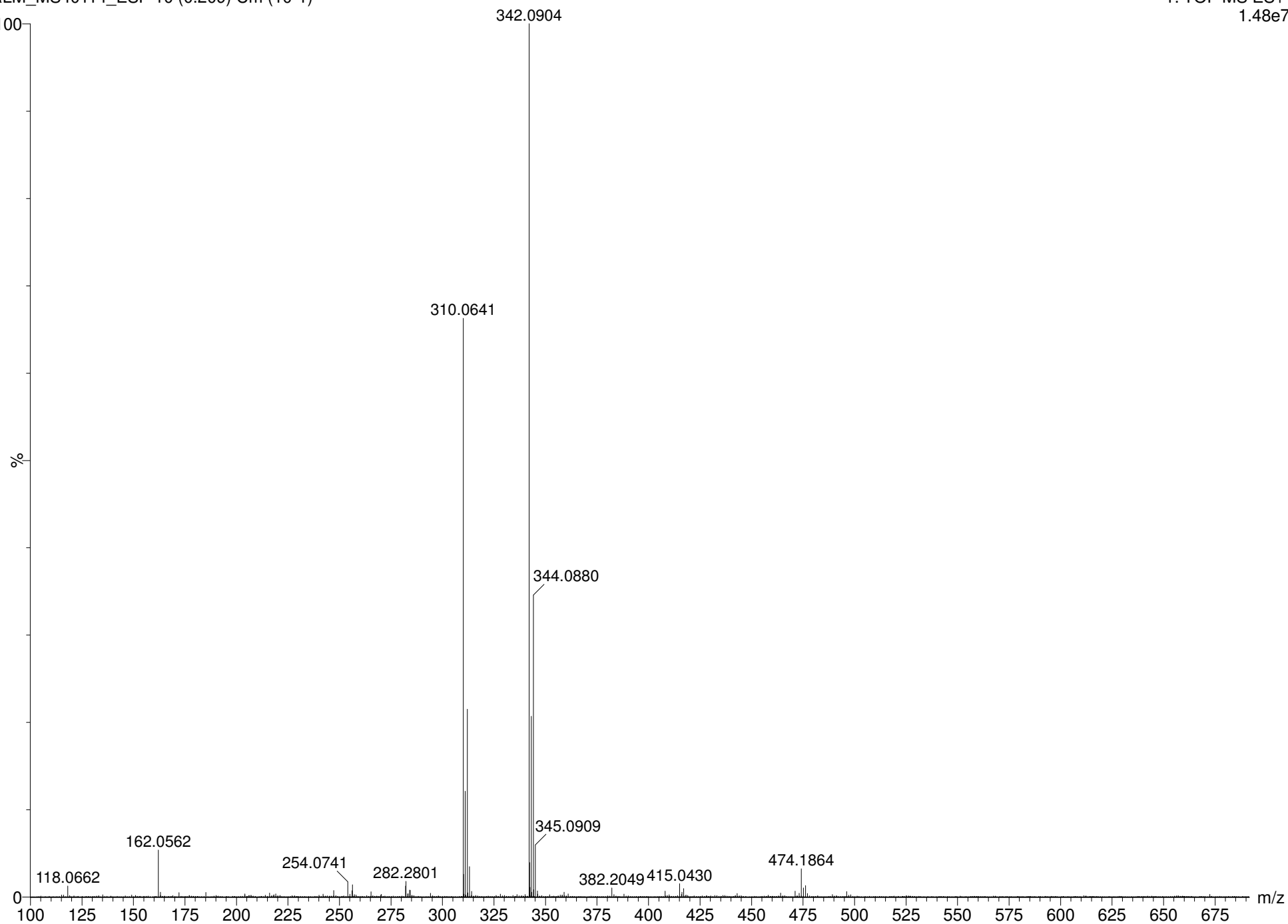

Supplement: Supplementary file 2 — ol2c04198_si_002.zip [file ol2c04198_si_002.zip › HMRS/6b_HR_ESP.pdf]

04-Nov-2022

XEVO-G2XSQTOF#NotSet  
Cardiff University  
1: TOF MS ES+  
4.79e5

RLM\_MS40114\_ESP 10 (0.209) Cm (10-1)

MG369D

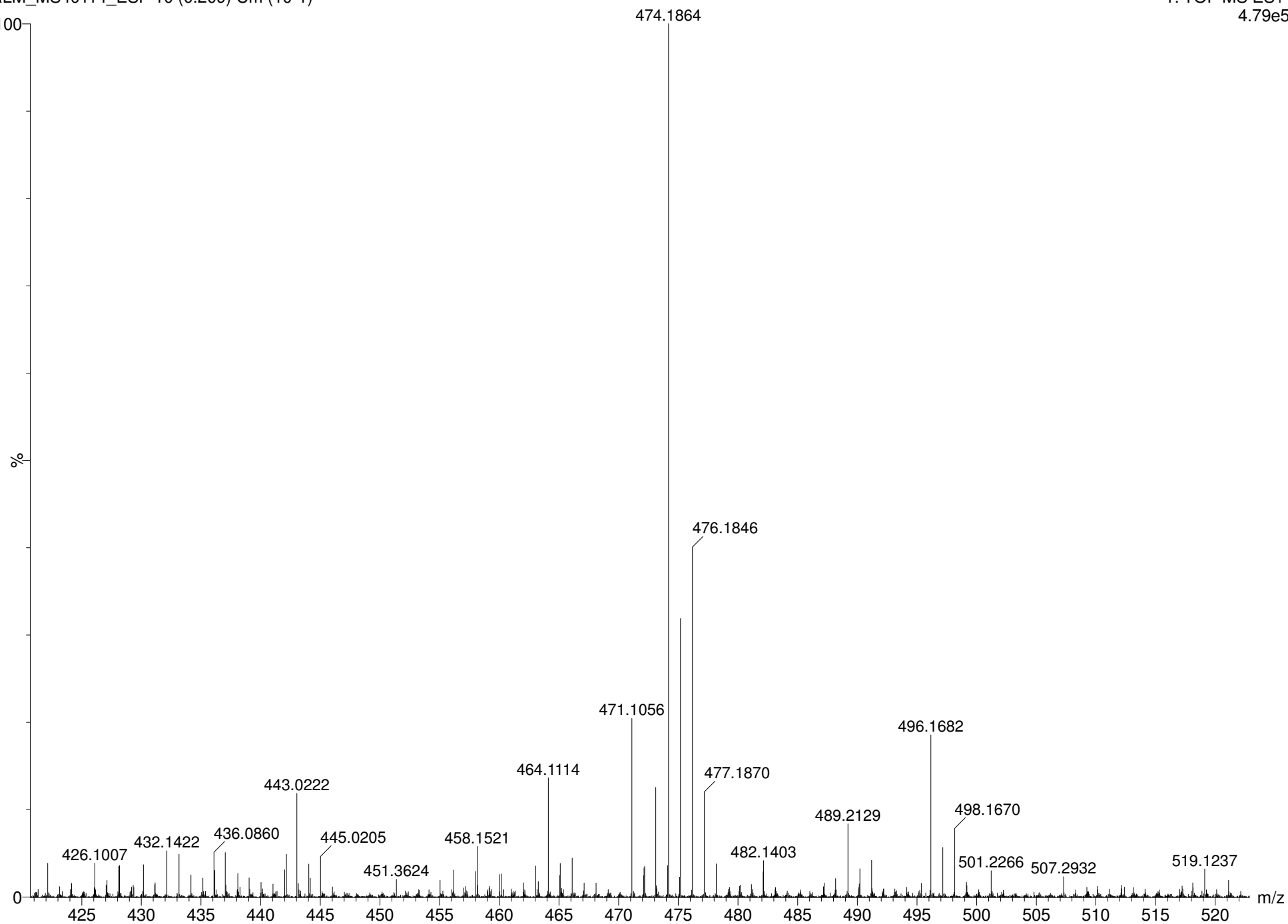

Supplement: Supplementary file 2 — ol2c04198_si_002.zip [file ol2c04198_si_002.zip › HMRS/6b_HR_ESPzoom.pdf]

15-Jul-2022

XEVO-G2XSQTOF#NotSet

Cardiff University

1: TOF MS ES+

5.46e6

RLM\_MS39048\_ESP 7 (0.155) Cm (7-1x20.000)

KS4H4BrB

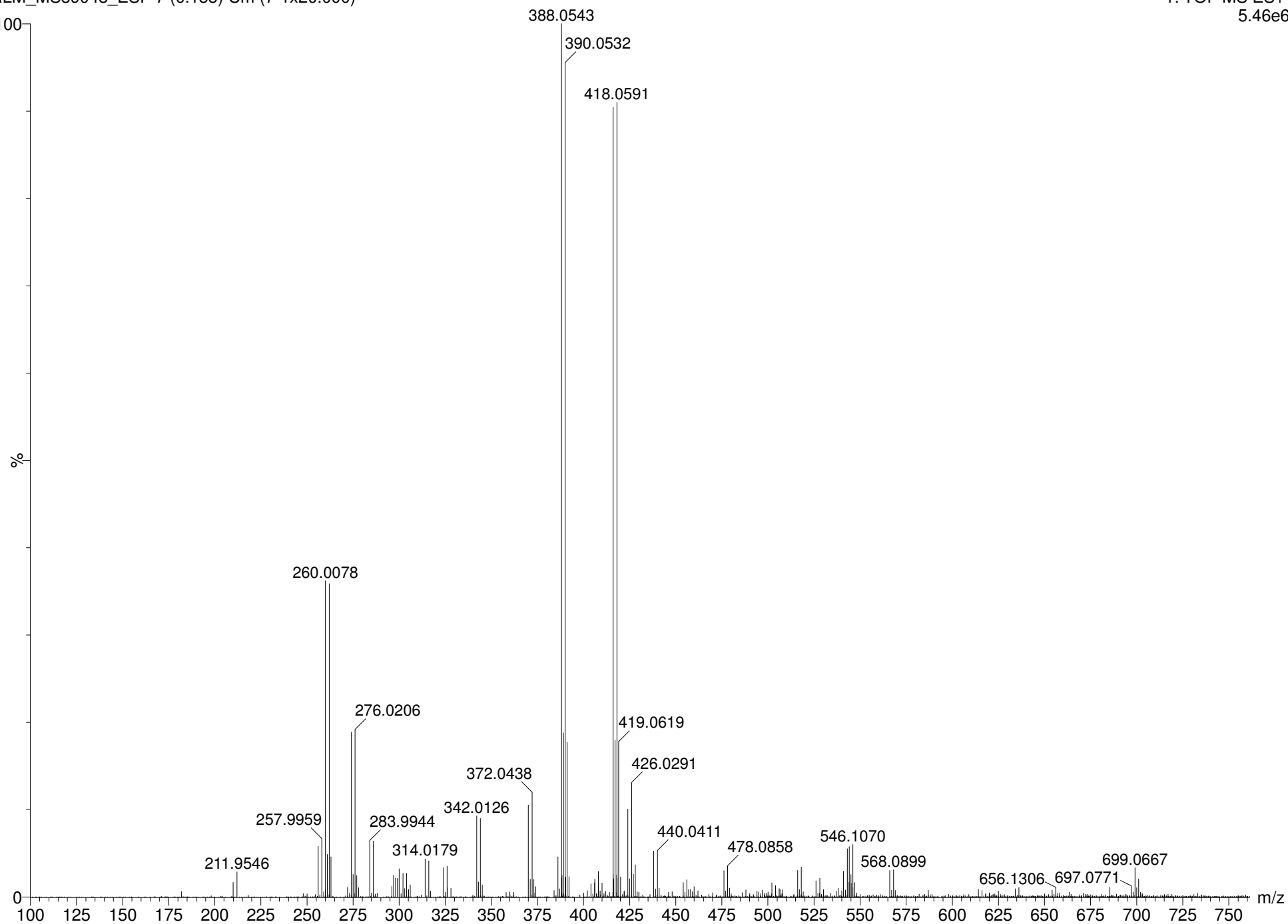

Supplement: Supplementary file 2 — ol2c04198_si_002.zip [file ol2c04198_si_002.zip › HMRS/anti-3n_HR_ESP.pdf]

01-Aug-2022

MG290A

XEVO-G2XSQTOF#NotSet  
Cardiff University  
1: TOF MS ES+  
2.40e7

RLM\_MS39143\_ESP 10 (0.226) Cm (10-1x20.000)

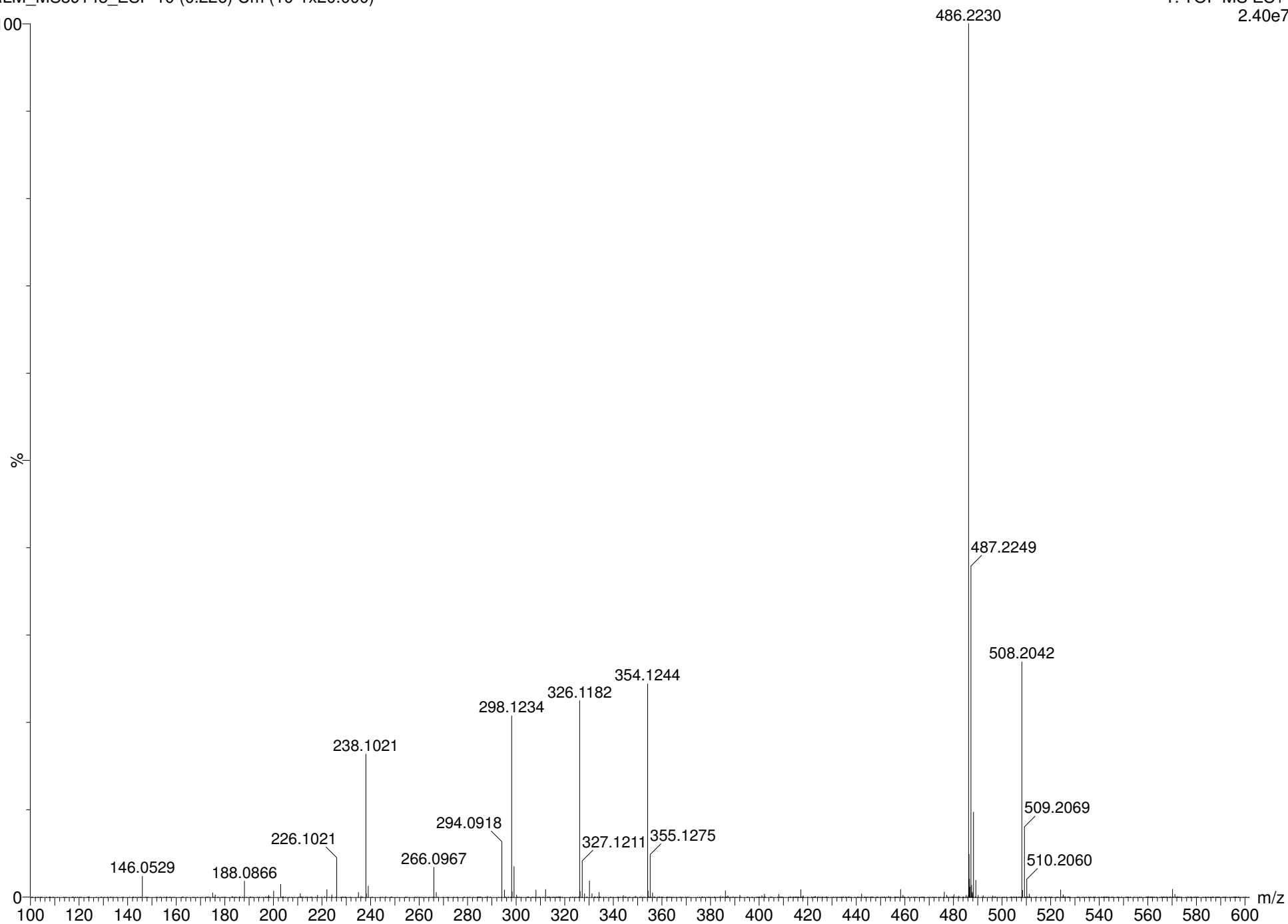

Supplement: Supplementary file 2 — ol2c04198_si_002.zip [file ol2c04198_si_002.zip › HMRS/anti-5d_HR_ES.pdf]

12-Jul-2022

KS4H4Br

XEVO-G2XSQTOF#NotSet  
Cardiff University  
1: TOF MS ES+  
1.77e5

RLM\_MS39021\_ESPrpt 20 (0.414) Cm (20-1)

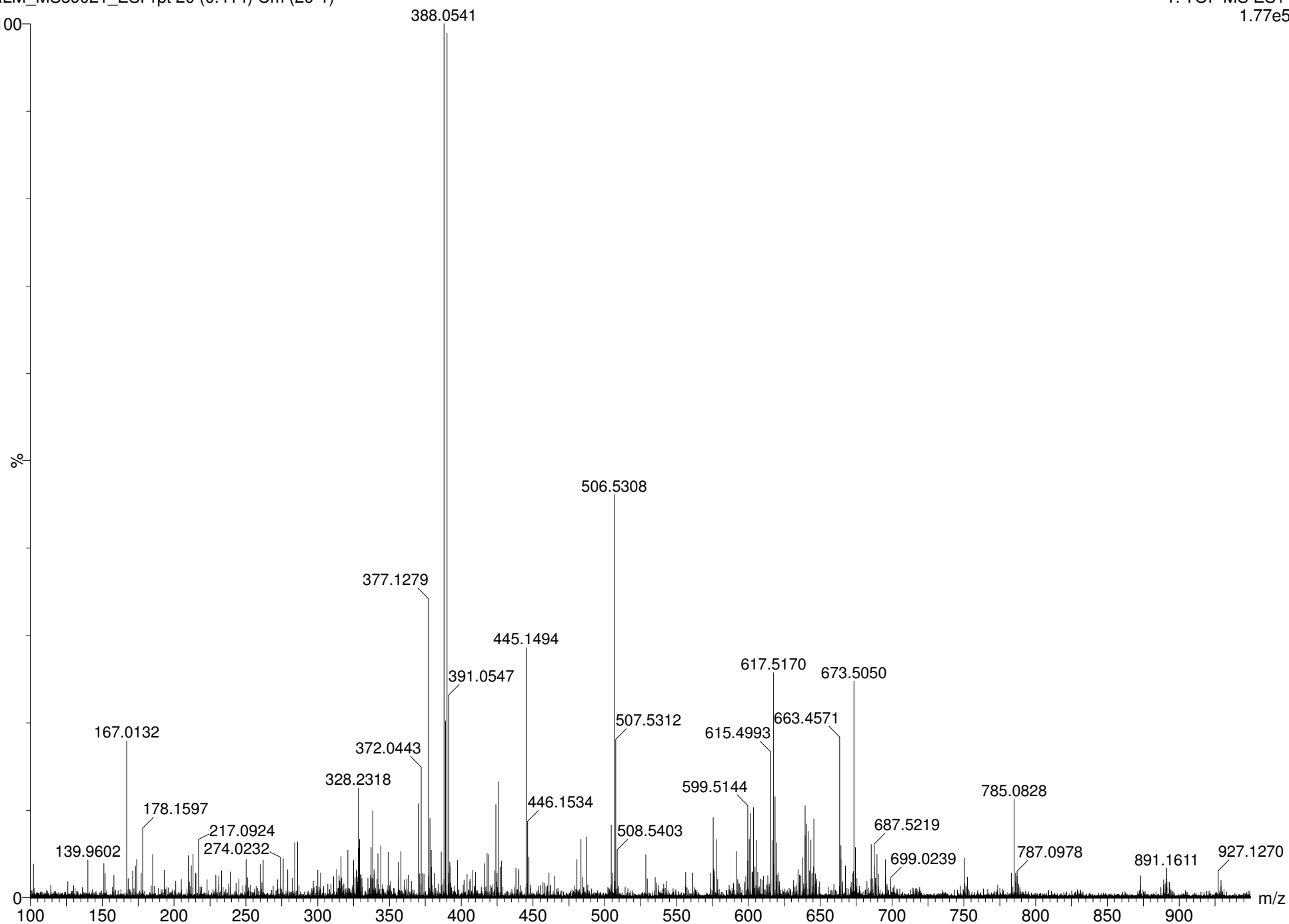

Supplement: Supplementary file 2 — ol2c04198_si_002.zip [file ol2c04198_si_002.zip › HMRS/syn-3n_HR_ESP.pdf]

01-Aug-2022

MG290B

XEVO-G2XSQTOF#NotSet

Cardiff University

1: TOF MS ES+

9.80e6

RLM\_MS39144\_ESP 10 (0.226) Cm (10-1x20.000)

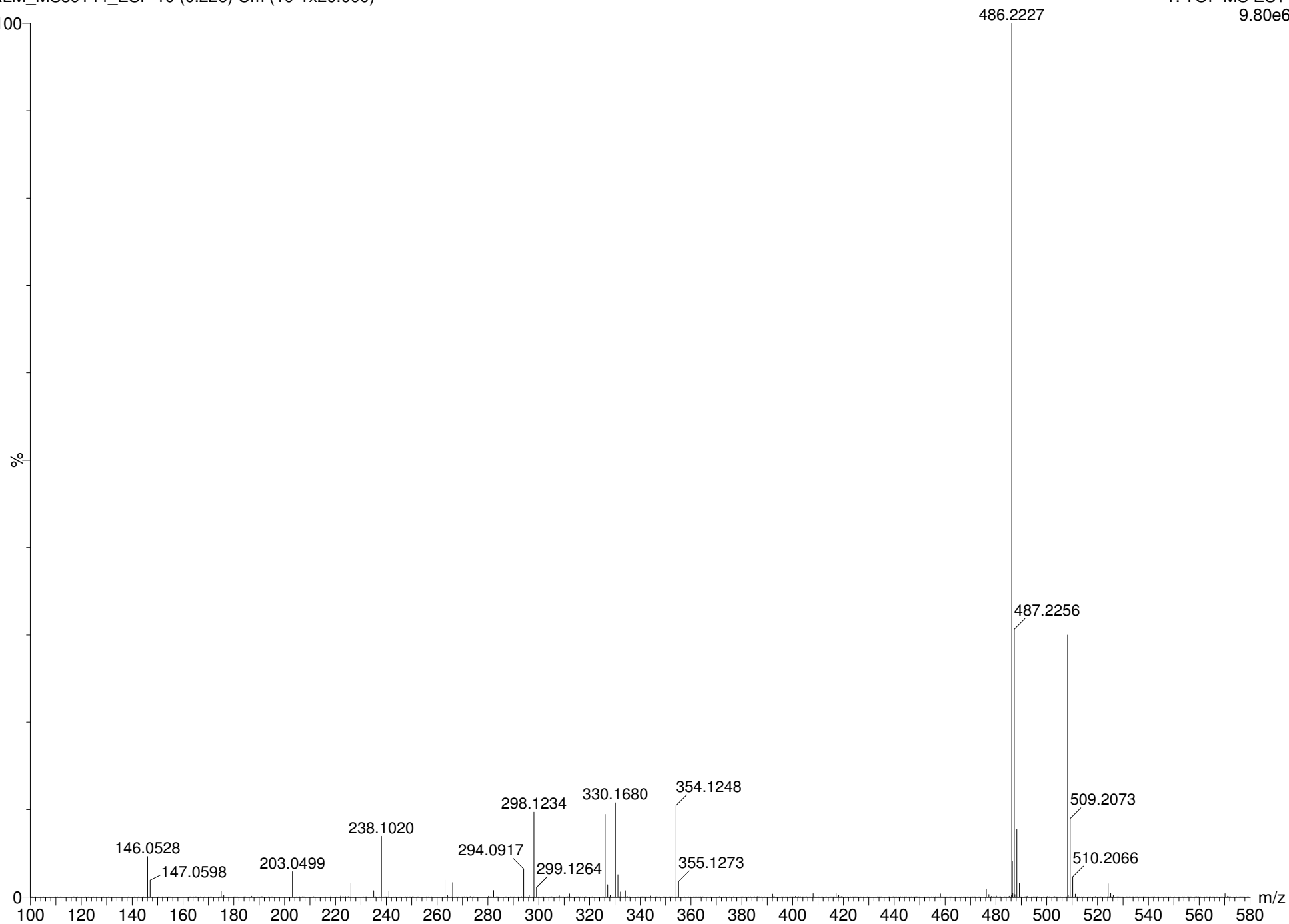

Supplement: Supplementary file 2 — ol2c04198_si_002.zip [file ol2c04198_si_002.zip › HMRS/syn-5d_HR_ES.pdf]
